# Photochemical Organocatalytic Functionalization of Pyridines via Pyridinyl Radicals

**DOI:** 10.1021/jacs.2c12466

**Published:** 2022-12-27

**Authors:** Emilien Le Saux, Eleni Georgiou, Igor A. Dmitriev, Will C. Hartley, Paolo Melchiorre

**Affiliations:** † ICIQ − Institute of Chemical Research of Catalonia, Avinguda Països Catalans 16, 43007 Tarragona, Spain; ‡ URV − Universitat Rovira i Virgili, 43007 Tarragona, Spain; § Department of Industrial Chemistry “Toso Montanari”, University of Bologna, Viale Risorgimento 4, 40136 Bologna, Italy

## Abstract

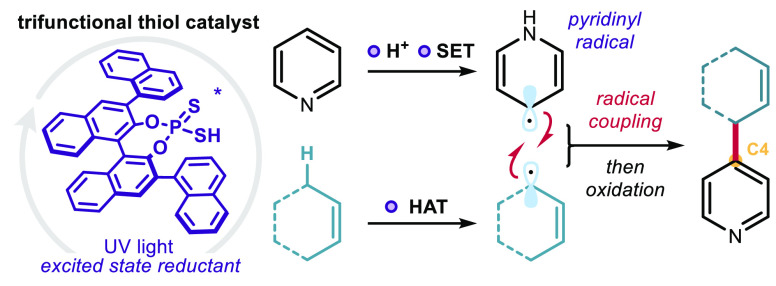

We report a photochemical method for the functionalization of pyridines with radicals derived from allylic C–H bonds. Overall, two substrates undergo C–H functionalization to form a new C(sp^2^)–C(sp^3^) bond. The chemistry harnesses the unique reactivity of pyridinyl radicals, generated upon single-electron reduction of pyridinium ions, which undergo effective coupling with allylic radicals. This novel mechanism enables distinct positional selectivity for pyridine functionalization that diverges from classical Minisci chemistry. Crucial was the identification of a dithiophosphoric acid that masters three catalytic tasks, sequentially acting as a Brønsted acid for pyridine protonation, a single electron transfer (SET) reductant for pyridinium ion reduction, and a hydrogen atom abstractor for the activation of allylic C(sp^3^)–H bonds. The resulting pyridinyl and allylic radicals then couple with high regioselectivity.

Pyridines and related nitrogen heterocycles are ubiquitous in biologically active molecules,^1^ with pyridine being the most abundant heteroaromatic ring in FDA-approved drugs.^2^ Therefore, methods for their regioselective modification are desirable. Contemporary radical chemistry has provided inroads for new pyridine functionalization strategies. The venerable Minisci reaction,^3^ which is based on the addition of nucleophilic carbon radicals to protonated azines, is a broadly used approach (Figure 1a). However, this process is generally characterized by competing C2/C4 positional selectivity.^4^ Strategies to selectively access C4-alkylated pyridines are available. For example, 4-cyano^5^ and 4-triphenylphosphonium^6^ pyridines are prone to react through an *ipso* substitution pathway upon single electron transfer (SET) reduction (Figure 1b, path *i*). Other C4-selective approaches require preinstallation of suitable protecting groups at the pyridine’s nitrogen, followed by a Minisci-type pathway (Figure 1b, path *ii*).^7^

**Figure 1:**
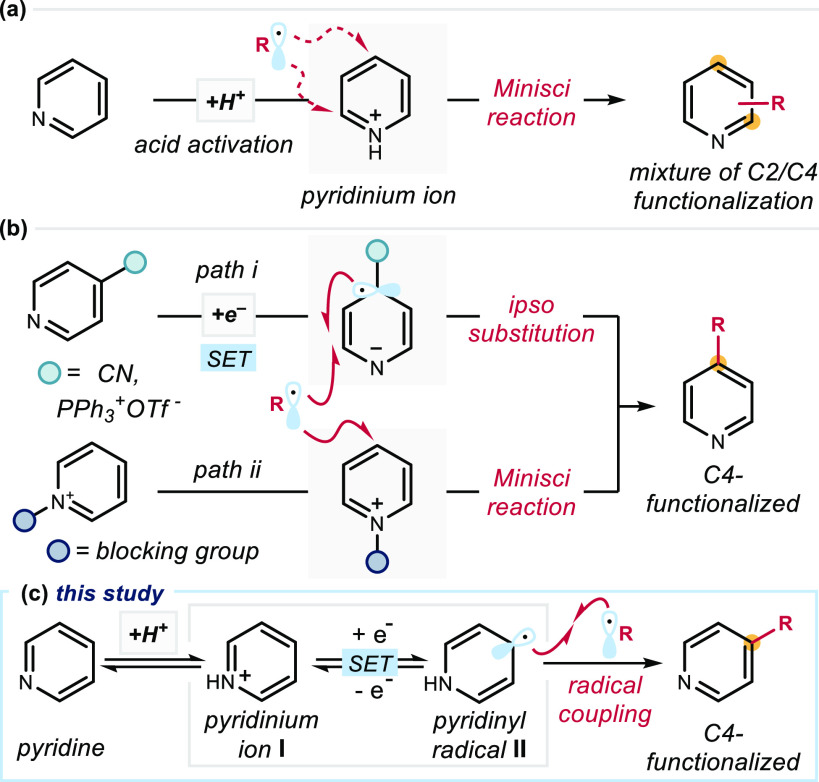
Radical pathways for the functionalization of pyridines: (a) Minisci reaction; (b) C4 functionalization via (path *i*) *ipso* substitution or (path *ii*) a Minisci process using blocking groups at the pyridine’s nitrogen; (c) The new reactivity promoted by pyridinyl radicals **II** generated upon SET reduction of pyridinium ions **I**.

Herein we disclose a new radical-based pathway for pyridine functionalization that mechanistically diverges from classical Minisci chemistry, enabling distinct positional selectivity (Figure 1c). This direct functionalization of simple pyridines is based on the reactivity of pyridinyl radicals **II**, generated upon SET reduction of pyridinium ions **I** formed under acidic conditions. The neutral radical **II** has been characterized in the past by EPR spectroscopy^8^ and exploited as a one-electron shuttle for the catalytic reduction of carbon dioxide.^9^ Surprisingly, pyridinyl radical **II** has been largely neglected in synthetic chemistry, since its use was limited to dimerization reactions.^10^ We demonstrate here that its reactivity can be leveraged to trap radicals derived from allylic C(sp^3^)–H bonds with high C4 regioselectivity. The net process couples two unmodified substrates via functionalization of a C–H bond on each substrate.^11^

This study was motivated by our recent findings on the catalytic reactivity of the dithiophosphoric acid **A** (structure in Figure 2a).^12^ Upon deprotonation, the electron-rich thiolate **A**^–^ can drive the formation of photoactive electron donor–acceptor (EDA) complexes.^13^ Visible-light excitation triggers intracomplex SET, leading to the thiyl radical **A**·, which activates the allylic substrate **2** via a hydrogen atom transfer (HAT) mechanism. We initially sought to translate this photochemical platform to the activation of pyridines **1**. Our original idea was that pyridine protonation by the acid catalyst **A**(14) would form a photoactive ion pair; however, we did not observe the formation of any light-absorbing aggregate (see Supporting Information (SI) section F.2). In contrast, we soon realized (as further detailed below) that the thiolate **A**^–^ could directly absorb light^15^ to generate a highly reducing excited state [**A**^–^]*, which could undergo SET to pyridinium ion **I** to afford the pyridinyl radical **II** (Figure 2a). This redox path also led to the sulfur-centered radical **A**·, which is prone to C–H abstraction from **2** to deliver allylic radical **III**.^12,16^ Radical coupling of **II** and **III** would afford, upon rearomatization, the target functionalized pyridine product **3**.

**Figure 2:**
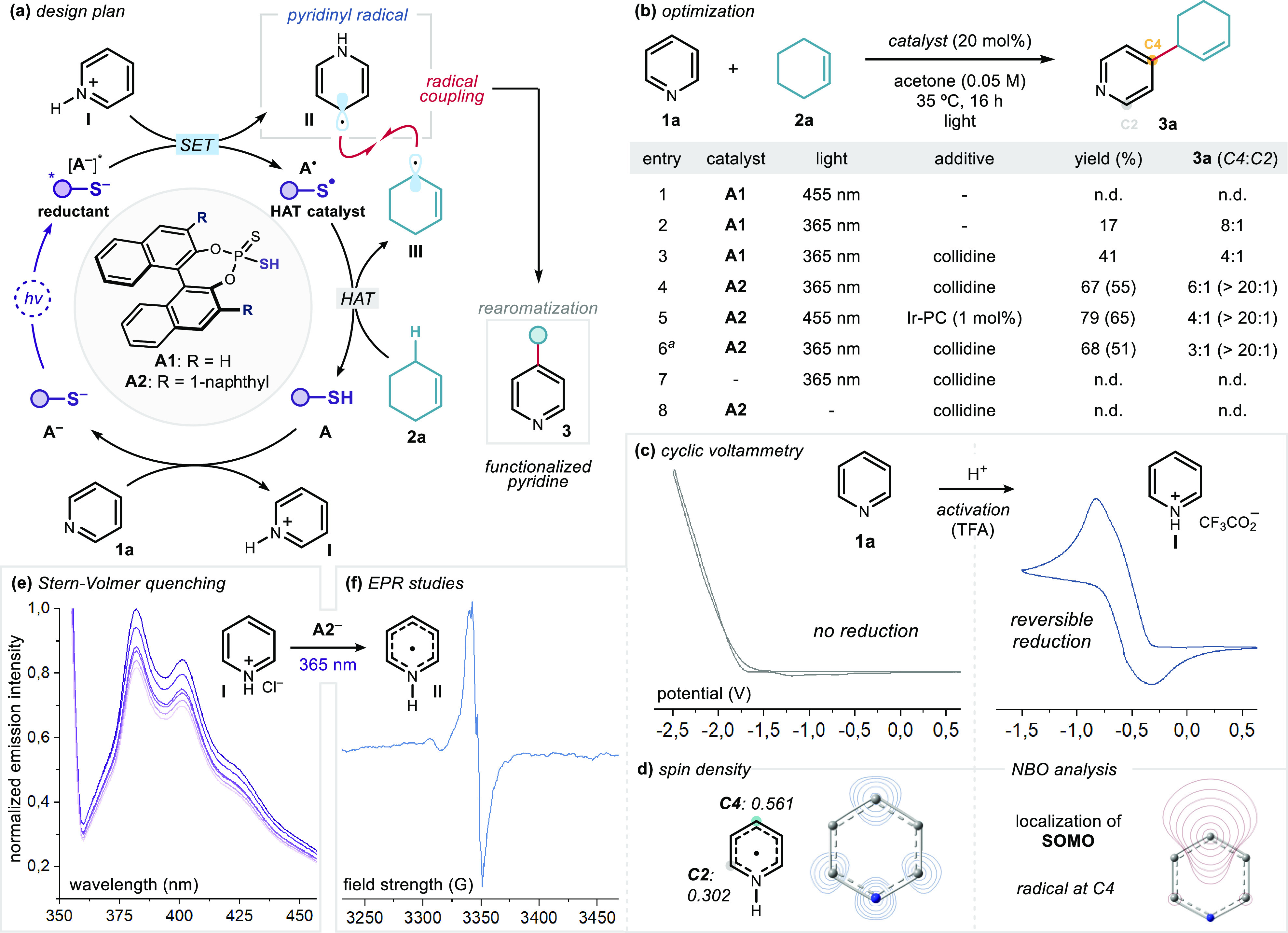
(a) Proposed mechanism of the direct functionalization of pyridine **1a** via the formation of pyridinyl radical **II**. (b) Optimization studies: reactions were performed on a 0.2 mmol scale at 35 °C for 16 h under illumination by a 365 nm EvoluChem LED spotlight using 10 equiv of **2a**. Yields and regioisomeric ratios were determined by ^1^H NMR analysis of the crude mixtures. Numbers in parentheses refer to yields of isolated **3a**. (c) Cyclic voltammetry studies (scan rate = 100 mV·s^–1^). (d) Calculated spin density and NBO analysis of pyridinyl radical **II** (uB3LYP/6-31G+(d) level of theory). (e) Stern–Volmer quenching studies of the excited anion of catalyst **A2** (using **A2**·Et_3_N as the source of **A2**^–^) with increasing amounts of pyridinium·TFA salt **I** ([**A2**^–^] = 5 mM; [**I**] = 0.125 to 0.625 mM; excitation wavelength = 350 nm). (f) EPR spectrum of pyridinyl radical **II** measured after 15 min of irradiation of a 5:1 mixture of **1a** and **A2** at 77 K. Collidine refers to 2,4,6-collidine (50 mol %); n.d. denotes not detected; Ir-PC = [Ir(dtbbpy)(ppy)_2_]PF_6_; TFA = trifluoroacetic acid. ^*a*^The reaction was performed on a 1 mmol scale.

This reasoning, although initially based on an inaccurate mechanistic hypothesis, explains our choice of the model reaction (Figure 2b). Specifically, we reacted simple pyridine (**1a**) and cyclohexene (**2a**) in the presence of the dithiophosphoric acid **A1** as the catalyst (20 mol %). The allylic substrate **2a** was chosen since our previous study^12^ demonstrated its propensity to undergo effective HAT from catalyst **A** to afford cyclohexenyl radical **III**. The first experiment, conducted in acetone as the solvent under irradiation by blue light (λ = 455 nm), afforded no product formation (entry 1). The lack of reactivity was reconciled with the absence of any visible-light-absorbing aggregate, as inferred by UV–vis spectroscopic analysis of a mixture of catalyst **A1** and pyridine **1a**. However, these studies established the ability of the deprotonated dithiophosphoric acid (thiolate **A1**^–^) to absorb in the near-UV region (see SI section F.2 for details). Performing the model reaction using an irradiation wavelength of 365 nm led to allylated pyridine **3a** in 17% yield with an 8:1 regioisomeric ratio in favor of the C4*-*functionalized adduct (entry 2).

An optimization campaign identified 2,4,6-collidine (50 mol %) as an effective additive to increase the overall yield, as **3a** was formed in 41% yield (Figure 2b, entry 3; see SI section F.8 for a discussion of the role of collidine). The use of the naphthyl-substituted dithiophosphoric acid catalyst **A2** improved the results, delivering the allylated pyridine **3a** in 67% yield with 6:1 C4 selectivity (entry 4). After purification by column chromatography, **3a** was obtained as a single positional isomer. Interestingly, reactivity under blue-light irradiation could be achieved by adding an iridium-based photocatalyst, which afforded a slightly improved yield (entry 5). The process was equally efficient on a 1 mmol scale (entry 6). Control experiments established that the absence of catalyst or light completely inhibited the transformation (entries 7 and 8). A tentative explanation of the rearomatization step is given in SI section F.9.

We then performed investigations to gain mechanistic insights. Electrochemical analysis of pyridine **1a** showed no reduction wave when a potential as low as −2.5 V vs Ag^+^/Ag in MeCN was applied (Figure 2c). However, a reversible reduction wave was observed for a preformed pyridinium trifluoroacetate salt, with the reduction occurring at approximately at −0.6 V vs Ag^+^/Ag. The observed well-shaped reversible behavior^17^ hinted at a certain kinetic stability of the delocalized pyridinyl radical **II**. We also evaluated the electronic properties of the key pyridinyl radical **II** with calculations performed at the uB3LYP/6-31G+(d) level. Greater spin density was found at C4 compared to C2 (Figure 2d), which is in agreement with the spin densities inferred from the EPR hyperfine coupling constants of **II**.^8c^ Natural bond orbital (NBO) analysis revealed that the singly occupied molecular orbital (SOMO) is localized preferentially at C4.^18^ These results are coherent with the experimentally observed C4 positional selectivity.

We then focused on the photochemical radical generation pathway.^19^ Specifically, we investigated the ability of the excited thiolate catalyst **A2**^–^ to generate pyridinyl radical **II** through SET reduction. Upon irradiation of the triethylammonium salt of catalyst **A2** (**A2**·Et_3_N, the source of **A2**^–^) at 350 nm, we detected emission centered at 382 nm (Figure 2e; also see SI section F.5). This confirmed that the deprotonated catalyst **A2** could access an electronically excited state. Applying the Rehm–Weller formalism,^20^ the redox potential of the excited thiolate [*E*(**A2**·/[**A2**^–^]*)] was estimated as −2.23 V vs Ag^+^/Ag in CH_3_CN (see SI section F.3). Therefore, the anion of catalyst **A2** becomes a strong reductant in the excited state, and SET to pyridinium ion **I** (*E* = −0.6 V vs Ag^+^/Ag in CH_3_CN) is highly exergonic. Stern–Volmer quenching studies confirmed that protonated pyridine **I** (pyridinium chloride) quenched the excited state of **A2**^–^ (Figure 2e). In addition, upon UV irradiation of a mixture of pyridine and catalyst **A2**, low-temperature EPR analysis detected the formation of a carbon-centered radical,^8c^ consistent with the pyridinyl radical **II** formed through SET quenching of [**A2**]* (Figure 2f). Finally, we used transient absorption spectroscopy (TAS) to measure a half-life of ∼5 μs for [**A2**^–^]* (see SI section F.4). Collectively, these studies support the mechanistic pathway proposed in Figure 2a.

Using the conditions described in Figure 2b, entry 4, the generality of the allylation of pyridines was evaluated (Figure 3). Notably, Minisci reactions with allylic radicals are rare,^21^ and the present radical coupling strategy based on the reactivity of pyridinyl radical **II** can address this gap in synthetic methodology. *Ortho*-substituted pyridines were converted in good yields and isolated exclusively as C4-allylated products **3b** and **3c**. Pyridines bearing *o*-halogen substituents were unsuccessful, instead undergoing dehalogenation (for a list of unsuccessful substrates, see Figure S1). The introduction of *m*-halogen substituents led to the formation of C4-functionalized pyridines **3d** and **3e** with high regioselectivity. Electron-withdrawing CF_3_ and CN substituents at the *meta* position were also tolerated, giving the C4 products **3f** and **3g** in good yields. An alkyl substituent was also accepted (**3h**). The introduction of a bulkier aryl substituent at the *meta* position caused a switch of regioselectivity in favor of C6 (**3i**). Considering the importance of nicotinic acid and its derivatives as lipid-controlling drugs,^22^ we tested the reactivity of niacin (vitamin B3) and nicotinate esters, nicotinamides, and halogenated derivatives. These substrates afforded products **3j**–**s** in good yields with a positional preference for C6 functionalization. A series of amino acid nicotinates proved to be suitable substrates, showcasing this method’s potential for the functionalization of complex pyridines (entries **3t**–**w**). These results suggest that the greater spin density at C4, which accounts for the positional selectivity with simple pyridines, is overridden in pyridines bearing bulky 3-substitutents, such as those derived from nicotinic acid.^10^ Further studies are ongoing to better rationalize the interplay of steric and electronic factors that dictate regioselectivity.

**Figure 3:**
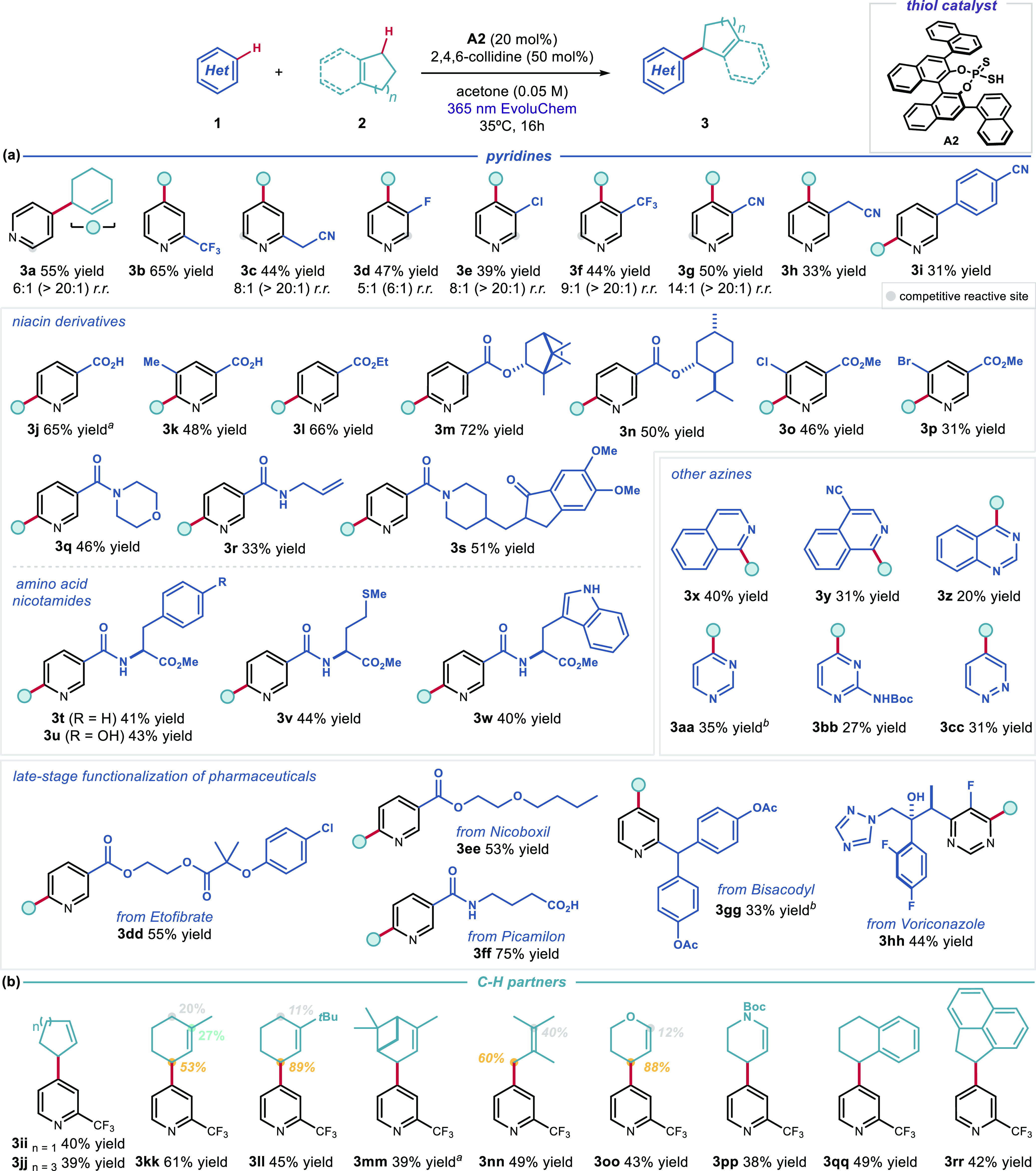
Photochemical organocatalytic allylation of pyridines and derivatives: (a) scope of pyridines **1**; (b) scope of allylic substrates **2**. Reactions were performed on a 0.2 mmol scale using 10 equiv of **2**. Yields refer to isolated products **3** after purification. Products **3** were obtained as single regioisomers (>20:1 *r.r.*), unless otherwise stated. When more than one regioisomer was observed, the minor site of functionalization is highlighted by a gray circle. In these cases, the regioisomeric ratio (*r.r.*) of the crude mixture is specified, and the *r.r.* after isolation is reported in parentheses. When applicable, *d.r.* was ∼1:1. ^*a*^Yield determined by ^1^H NMR analysis. ^*b*^ The conditions described in Figure 2b, entry 5 were used. Boc = *tert*-butoxycarbonyl.

Other azines, including isoquinolines (**3x** and **3y**), pyrimidines (**3z**–**bb**), and pyridazine (**3cc**), also delivered the allylated products with high regioselectivity in modest yields. Next, we turned our attention to the direct functionalization of pharmaceuticals. Three niacin derivatives, i.e., nicoboxil (a rubefacient), etofibrate (a hypolipidemic agent), and picamilon (a dietary supplement), were successfully functionalized in good yields with exclusive C6 regioselectivity (products **3dd**–**ff**), while the *ortho*-substituted pyridine bisacodyl (a stimulant laxative drug) and pyrimidine-based voriconazole (an antifungal) afforded the C4-allylated adducts **3gg** and **3hh**, respectively, in synthetically useful yields.

We then evaluated other C(sp^3^)–H partners that could serve as radical precursors for the functionalization of 2-trifluoromethylpyridine (Figure 3b). Cyclic alkenes with different ring sizes and substitution patterns afforded the desired products with complete C4 positional selectivity. Tetramethylethylene (**3nn**) and heterocycles, including dihydropyran (**3oo**) and a protected piperidine (**3pp**), were also suitable allylic precursors. Finally, the sp^3^ C–H benzylic precursors tetrahydronaphthalene and acenaphthylene were successfully reacted, affording adducts **3qq** and **3rr**, respectively.

Further mechanistic studies were undertaken to probe the formation of the key pyridinyl radical **II** (Figure 4). Both C2- and C4-cyclopropylpyridines **4a** and **4b** underwent ring opening under the standard conditions to give products **5a** and **5b** in 51% and 45% yield, respectively. The formation of these products is consistent with the intermediacy of **II**, which upon ring opening to give highly stabilized benzylic radical **IV**(23) can undergo radical coupling with allylic radical **III**.

**Figure 4:**
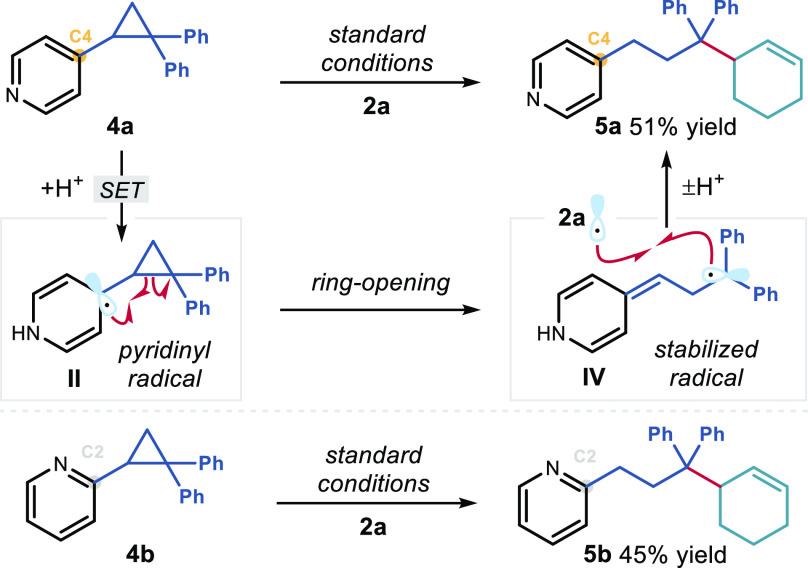
Probing the formation of pyridinyl radical **II**.

In summary, we have developed a new strategy for the radical functionalization of pyridines and related azine hetereoarenes based on the unique reactivity of pyridinyl radicals. These previously neglected intermediates, readily generated upon SET reduction of pyridinium ions, undergo effective radical coupling with allylic radicals. We envisage that this reactivity will offer further opportunities to develop new pyridine functionalization strategies.

## Supporting Information Available

Details of experimental procedures, full characterization data, and copies of NMR spectra (PDF)
