# Supplementary material for: Photochemical Organocatalytic Functionalization of Pyridines via Pyridinyl Radicals
Source: J Am Chem Soc. 2022 Dec 27;145(1):47–52. doi: 10.1021/jacs.2c12466 (PMC9837848; doi:10.1021/jacs.2c12466)
Supplement: Supplementary file 1 — ja2c12466_si_001.pdf [file ja2c12466_si_001.pdf]

Supporting Information

# Photochemical Organocatalytic Functionalization of Pyridines via Pyridinyl Radicals

Emilien Le Saux,[a,b] Eleni Georgiou,[a,b] Igor A. Dmitriev,[a,b] Will C. Hartley,[a]
and Paolo Melchiorre*[c]

[a]*ICIQ - Institute of Chemical Research of Catalonia*
Av. Països Catalans 16, 43007 Tarragona, Spain

[b]*URV – Universitat Rovira I Virgili*
Campus Sescelades, Carretera de Valls, S/N, 43007 Tarragona, Spain

[c] *University of Bologna – Department of Industrial Chemistry 'Toso Montanari'*
Viale Risorgimento 4, 40136 Bologna, Italy

*Correspondence to: pmelchiorre@unibo.it

A. General information ........ 3
B. Supplementary figures ........ 4
C. Synthesis of dithiophosphoric acid catalysts ........ 5
D. Synthesis of the substrates ........ 9
E. Experimental procedures ........ 17
F. Mechanistic studies ........ 37
F.1 Electrochemical studies ........ 37
F.2 Absorption spectroscopy analysis ........ 40
F.3 Evaluation of the excited-state potential of **A2**$^-$ ........ 41
F.4 Transient Absorption Spectroscopy (TAS) ........ 41
F.5 Emission spectrum and Stern-Volmer quenching studies ........ 42
F.6 Electron Paramagnetic Resonance (EPR) ........ 48
F.7 Radical clock experiments ........ 49
F.8 Discussion on the role of collidine ........ 50
F.9 Oxidation of the dihydropyridine intermediate ........ 50
G. Computational studies ........ 53
H. References ........ 61
I. NMR spectra ........ 63
J. UPC$^2$ traces ........ 128

## A. General information

The NMR spectra were recorded at 300 MHz, 400 MHz and 500 MHz for $^{1}H$ or at 75 MHz, 101 MHz and 126 MHz for $^{13}C$, 376 MHz for $^{19}F$, 162 MHz for $^{31}P$, respectively. The chemical shifts (δ) for $^{1}H$ and $^{13}C\{^{1}H\}$ are given in ppm relative to residual signals of the solvents ($CHCl_3$ @ 7.26 ppm $^{1}H$ NMR, 77.00 ppm $^{13}C$ NMR). Coupling constants are given in Hz. The following abbreviations are used to indicate the multiplicity: s, singlet; d, doublet; t, triplet; q, quartet; m, multiplet; br s, broad signal.

High-resolution mass spectra (HRMS) were obtained from the ICIQ High-Resolution Mass Spectrometry Unit on MicroTOF Focus and Maxis Impact (Bruker Daltonics) with electrospray ionization or atmospheric pressure chemical ionization. UV-vis measurements were carried out on a Shimadzu UV-2401PC spectrophotometer equipped with photomultiplier detector, double beam optics and D2 and W light sources.

*The authors are indebted to the team of the Research Support Area at ICIQ, particularly to the NMR, and the High-Resolution Mass Spectrometry Units.*

**General Procedures.** All reactions were set up under an argon atmosphere in oven-dried glassware using standard Schlenk techniques, unless otherwise stated. Synthesis grade solvents were used as purchased. Anhydrous solvents were taken from a commercial SPS solvent dispenser. Chromatographic purification of products was accomplished using flash column chromatography (FC) on silica gel (230-400 mesh). For thin layer chromatography (TLC) analysis throughout this work, Merck precoated TLC plates (silica gel 60 $GF_{254}$, 0.25 mm) were used, using UV light as the visualizing agent and either phosphomolybdic acid in EtOH, dinitrophenylhydrazine in EtOH/$H_2O$, *p*-anisaldehyde or basic aqueous potassium permanganate ($KMnO_4$), and heat as developing agents. Organic solutions were concentrated under reduced pressure on a Büchi rotary evaporator (*in vacuo* at 40 ºC, ~5 mbar).

**Determination of Diastereomeric Ratio.** The diastereomeric ratio was determined by $^{1}H$ NMR analysis of the crude reaction mixture through integration of diagnostic signals, or by $UPC^2$ analysis on chiral stationary phase using a Waters Acquity instrument. The exact conditions for the analyses are specified within the characterization section.

**Materials:** Commercial grade reagents and solvents were purchased at the highest commercial quality from Sigma Aldrich, Fluka, Acros Organics, Fluorochem, or Alfa Aesar and used as received, unless otherwise stated.

## B. Supplementary Figures

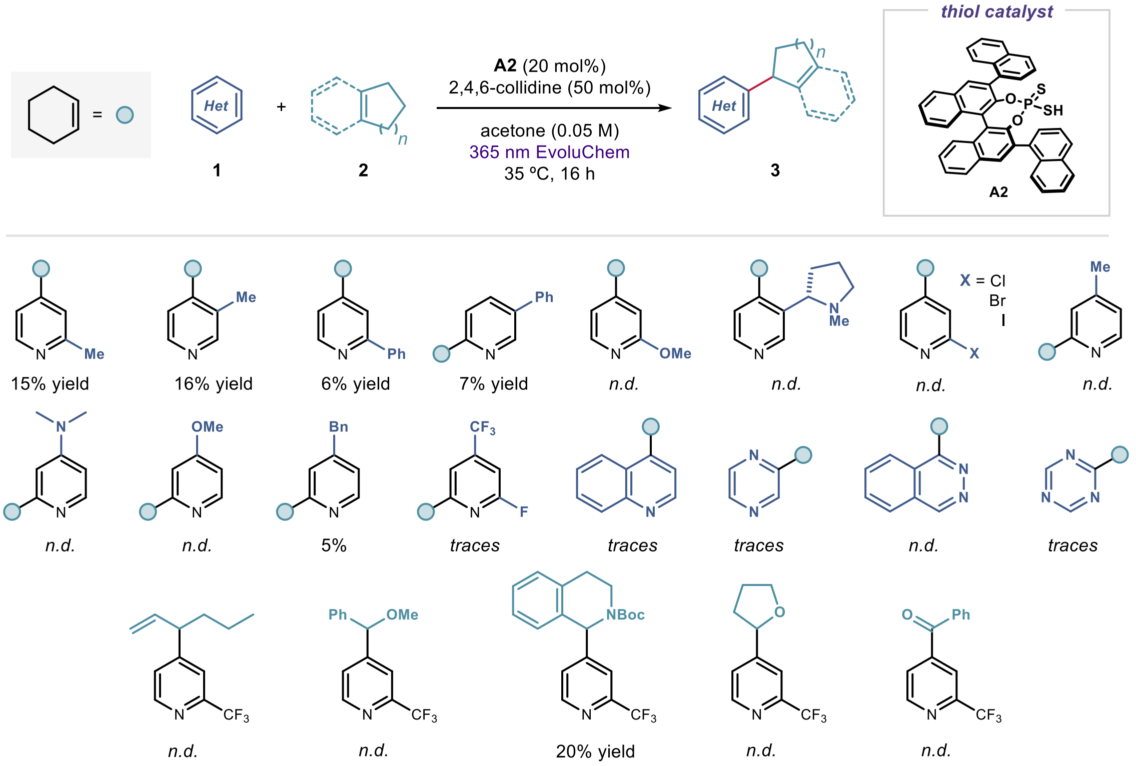


**Figure S1.** Moderately successful and unsuccessful substrates in the C-H allylation of pyridines and derivatives. Yields and ratios determined by $^1$H NMR analysis of the crude mixtures. *n.d.*: not detected.

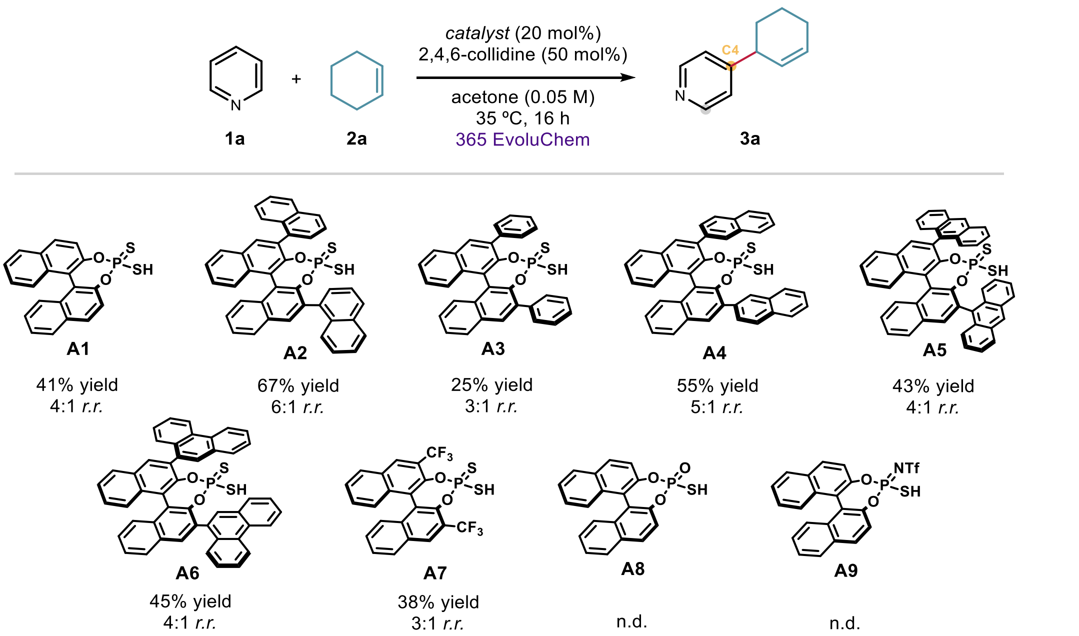


**Figure S2.** Screening of catalysts for the C-H allylation of pyridine. Yields and ratios were determined by $^1$H NMR analysis of the crude mixtures. *r.r.* refers to the C4/C2 ratio in **3a**. *n.d.*: not detected. Catalysts **A1–7** were synthesized as described in section C. Catalysts **A8–9** were prepared according to a reported procedure.[1]

## C. Synthesis of dithiophosphoric acid catalysts

### C.1 Synthesis of binols

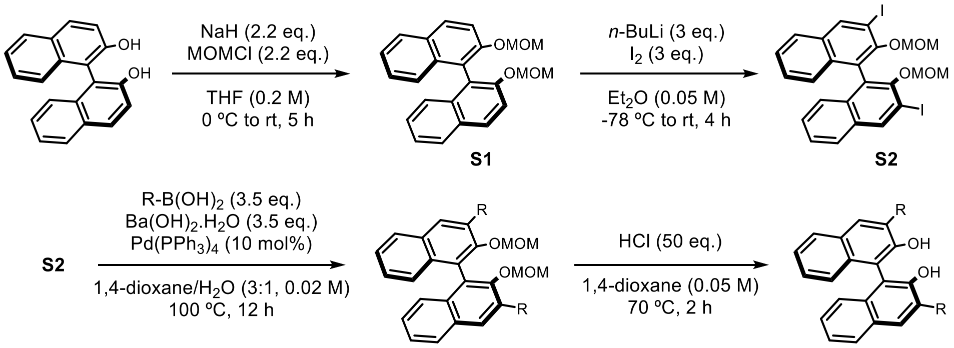


**Scheme S1.** Synthetic route for the preparation of 3,3'-substiuted (*S*)-binol derivatives.

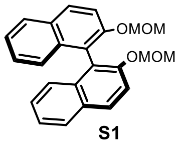

S1

**(*S*)-2,2'-bis(methoxymethoxy)-1,1'-binaphthalene (S1)** NaH (1.8 g, 2.2 equiv., 60% in mineral oil) was suspended in dry THF (90 mL) at 0 ºC under an atmosphere of argon. A solution of (*S*)-2,2'-dihydroxy- 1,1'-binaphthyl (5.73 g, 20.0 mmol) in THF (30 mL) was added dropwise and the mixture stirred at 0 ºC for 1 h and then at room temperature for 30 min. After the mixture was cooled-down to 0 ºC, chloromethyl methyl ether (3.33 mL, 2.2 equiv.) was slowly added and the reaction mixture was warmed-up to room temperature and stirred for 5 h. Saturated $NH_4Cl$ (50 mL) was added to the flask, then the solvent was removed in vacuo. The residue was extracted with $CH_2Cl_2$ (50 mL × 3). The organic layers were combined, washed with brine (50 mL), dried over $MgSO_4$, filtered and concentrated. The crude product was triturated with cold hexanes, filtered and dried under high vacuum to afford **S1** as a white solid (6.82 g, 91% yield).

$^{1}$H NMR (400 MHz, $CDCl_3$) δ 7.97 (d, *J* = 8.7 Hz, 2H), 7.89 (d, *J* = 8.1 Hz, 2H), 7.60 (d, *J* = 9.0 Hz, 2H), 7.36 (ddd, *J* = 8.1, 6.6, 1.3 Hz, 2H), 7.24 (ddd, *J* = 8.1, 6.7, 1.3 Hz, 2H), 7.18 (d, *J* = 8.5 Hz, 2H), 5.10 (d, *J* = 6.8 Hz, 2H), 5.00 (d, *J* = 6.8 Hz, 2H), 3.17 (s, 6H).
$^{13}$C NMR (126 MHz, $CDCl_3$) δ 152.7, 134.0, 129.9, 129.4, 127.9, 126.3, 125.6, 124.1, 121.3, 117.3, 95.2, 55.8, 29.7.

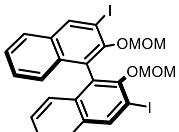

S2

**(*S*)-3,3'-diiodo-2,2'-bis(methoxymethoxy)-1,1'-binaphthalene (S2)** To a solution of MOM protected (*S*)-binol **S1** (6.82 g, 18.2 mmol) in dry $Et_2O$ (350 mL, 0.05 M), was added *n*-BuLi (2.5 M in hexanes, 21.9 mL, 3 equiv.) at 0 ºC. The resulting mixture was warmed-up to room temperature and stirred for 3 h. It was then cooled-down to -78 ºC and $I_2$ (13.9 g, 3 equiv.) was added portionwise. The reaction mixture was allowed to warm-up to room temperature overnight. Saturated $NH_4Cl$ (100 mL) was then added and the biphasic mixture diluted with water (100 mL) and EtOAc (100 mL). The aqueous layer was extracted with EtOAc (100 mL x 2), and the combined organic layers washed with water (100 mL x 2), 10% $Na_2S_2O_3$ (100 mL x 2), brine, dried over $MgSO_4$, filtered and concentrated under reduced pressure. The crude product was purified by column chromatography ($SiO_2$, 5:95 EtOAc/hexanes) to afford **S2** as an off-white foamy solid (6.9 g, 60% yield).

$^{1}$H NMR (500 MHz, $CDCl_3$) δ 8.56 (s, 2H), 7.80 (d, *J* = 7.8 Hz, 2H), 7.45 (ddd, *J* = 8.1, 6.7, 1.2 Hz, 2H), 7.32 (ddd, *J* = 8.2, 6.8, 1.3 Hz, 2H), 7.19 (d, *J* = 8.5 Hz, 2H), 4.83 (d, *J* = 5.7 Hz, 2H), 4.71 (d, *J* = 5.7 Hz, 2H), 2.62 (s, 6H).

The remaining steps for the synthesis of substituted binols are illustrated with the preparation of the 1-naphthyl-subsituted binol.

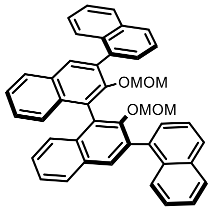


**(*S*)-3,3'-bis(1-naphtyl)-2,2'-bis(methoxymethoxy)-1,1'-binaphthalene** To **S2** (4.0 g, 6.39 mmol), $Ba(OH)_2·8H_2O$ (7.1 g, 3.5 equiv.), and $Pd(PPh_3)_4$ (738 mg, 0.1 equiv.) was added 1,4-dioxane/$H_2O$ (320 mL, 0.02 M, 3:1 mixture) at room temperature, under an atmosphere of argon. Naphthalene-1-boronic acid (3.85 g, 3.5 equiv.) was then added in one portion, and the mixture stirred at 100 ºC for 12 h. 1,4-dioxane was then removed under reduced pressure, and the residue extracted with $CH_2Cl_2$ (100 mL x 3), the combined organic layers washed with brine, dried over $MgSO_4$, filtered and concentrated under reduced pressure. The crude product was purified by column chromatography ($SiO_2$, 5:95 EtOAc/hexanes) to afford the product as a white solid (3.85 g, 96% yield).

NMR analysis of this compound is complicated by the presence of multiple conformers due to slow rotation around the 3,3′-biaryl bonds and the unsymmetrical nature of the 1-naphthyl substituent.

$^1$H NMR (400 MHz, $CDCl_3$) δ 8.05 – 7.35 (m, 24H), 4.53 – 4.18 (m, 4H), 2.20 – 2.12 (m, 6H).

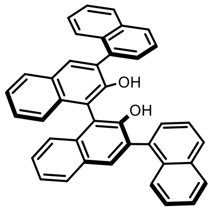


**(*S*)-3,3'-bis(1-naphthyl)-2,2'-dihydroxy-1,1'-binaphtalene** To (*S*)-3,3'-bis(1-naphtyl)-2,2'-bis(methoxymethoxy)-1,1'-binaphthalene (3.85 g, 6.2 mmol) dissolved in 1,4-dioxane (20 mL, 0.3M) was added concentrated HCl (10 mL). The resulting mixture was stirred at 70 ºC for 2h. The solvent was then removed under reduced pressure, the residue dissolved in $CH_2Cl_2$ (100 mL) and the organic layer washed with water (100 mL), saturated $NaHCO_3$ (100 mL), brine, dried over $MgSO_4$, filtered and concentrated under reduced pressure. After drying under high-vacuum for several hours, the product was obtained as a light-yellow solid (3.4 g, quant. yield) that was used in the next step without further purification.

NMR analysis of this compound is complicated by the presence of multiple conformers due to slow rotation around the 3,3′-biaryl bonds and the unsymmetrical nature of the 1-naphthyl substituent.

$^1$H NMR (400 MHz, CDCl3) δ 8.07 – 8.02 (m, 2H), 8.02 – 7.91 (m, 6H), 7.89 – 7.83 (m, 1H), 7.78 – 7.59 (m, 5H), 7.59 – 7.35 (m, 10H), 5.30 – 5.16 (m, 2H).

### C.2 Synthesis of Catalysts A1-7

### *GP1* – General Procedure for the Synthesis of Binol-derived Dithiophosphoric Acids

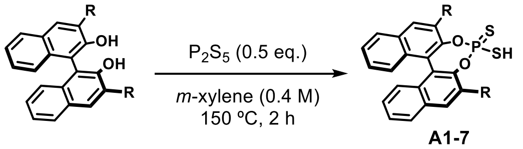


**Scheme S2.** Synthesis of dithiophosphoric acid catalysts **A**.

A flame dried flask was charged with the appropriate (*S*)-binol derivative (1.0 equiv.), $P_2S_5$ (0.5 equiv.), and anhydrous *m*-xylene (0.2 M). The flask was equipped with a condenser and placed in an aluminum heating block preheated to 150 ºC. The progress of the reaction was monitored by disappearance of the phenolic protons, as inferred by $^1$H NMR analysis of the crude mixture.

After 2 h, the reaction was completed and the mixture was cooled to ambient temperature. The solvent was removed in vacuo. The crude product was dissolved in a minimum amount of $CH_2Cl_2$ and treated with hexanes. The resulting fine precipitate was then collected by filtration and the operation repeated until no more precipitate was formed. The pure dithiophosphoric acids **A1-7** were obtained after washing the precipitates with cold hexanes.

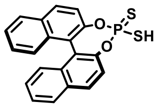

A1

**(11bS)-4-mercaptodinaphtho[2,1-d:1',2'-f][1,3,2]dioxaphosphepine 4-sulfide (A1)** Prepared according to GP1 using (S)-1,1’-bi-2-naphtol (2.23g, 5.86 mmol). **A1** (2.23g, 5.86 mmol, 73% yield) was obtained as a white powder which displayed spectroscopic data consistent with those reported previously.[2]

<u>$^{1}$H NMR</u> (400 MHz, $CDCl_3$) δ 8.13 – 8.07 (m, 2H), 8.00 (dd, *J* = 8.1, 1.2 Hz, 2H), 7.60 (dd, *J* = 8.8, 1.4 Hz, 2H), 7.54 (ddt, *J* = 8.0, 6.8, 1.1 Hz, 2H), 7.48 – 7.42 (m, 2H), 7.36 (ddd, *J* = 8.4, 6.7, 1.3 Hz, 2H).
<u>$^{13}$C NMR</u> (75 MHz, $CDCl_3$) δ 147.2, 132.4, 132.0, 131.1, 128.6, 127.2, 126.9, 126.1, 122.6, 121.1.
<u>$^{31}$P NMR</u> (162 MHz, $CDCl_3$) δ 100.2.

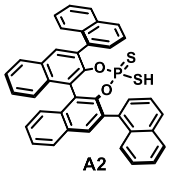

A2

**(11bS)-4-mercapto-2,6-di(naphthalen-1-yl)dinaphtho[2,1-d:1',2'-f][1,3,2]dioxaphosphepine 4-sulfide (A2)** Prepared according to GP1 using (1′S)-[1,3′:1′,1″:3″,1‴-Quaternaphthalene]-2′,2″-diol (1.25 g, 2.32 mmol). **A2** (1.17 g, 1.85 mmol, 80% yield) was obtained as a white powder which displayed spectroscopic data consistent with those reported previously.[3]

NMR analysis of this compound is complicated by the presence of multiple conformers due to slow rotation around the 3,3′-biaryl bonds and the unsymmetrical nature of the 1-naphthyl substituent.

<u>$^{1}$H NMR</u> (400 MHz, $CDCl_3$) δ 8.24 – 7.31 (m, 24H).
<u>$^{31}$P NMR</u> (162 MHz, $CDCl_3$) δ 96.3, 96.2, 96.1.

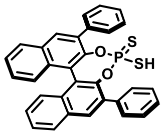

A3

**(11bS)-4-mercapto-2,6-diphenyldinaphtho[2,1-d:1',2'-f][1,3,2]dioxaphosphepine 4-sulfide (A3)** Prepared according to GP1 using (1S)-3,3′-Diphenyl[1,1′-binaphthalene]-2,2′-diol (317 mg, 0.72 mmol). **A3** (256 mg, 0.48 mmol, 67% yield) was obtained as a white powder which displayed spectroscopic data consistent with those reported previously.[2]

<u>$^{1}$H NMR</u> (500 MHz, CDCl3) δ 8.14 (s, 2H), 8.06 – 8.02 (m, 2H), 7.75 – 7.71 (m, 4H), 7.58 (dt, J = 7.0, 1.0 Hz, 2H), 7.52 – 7.42 (m, 8H), 7.38 (ddd, J = 8.5, 6.8, 1.3 Hz, 3H).
<u>$^{13}$C NMR</u> (101 MHz, CDCl3) δ 137.2, 131.4, 130.2, 129.6, 128.9, 128.6, 128.3, 127.8, 127.1, 126.7, 126.3.
<u>$^{31}$P NMR</u> (202 MHz, CDCl3) δ 96.2.

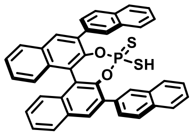

A4

**(11bS)-4-mercapto-2,6-di(naphthalen-2-yl)dinaphtho[2,1-d:1',2'-f][1,3,2]dioxaphosphepine 4-sulfide (A4)** Prepared according to GP1 using (1′S)-[2,3′:1′,1″:3″,2‴-Quaternaphthalene]-2′,2″-diol (590 mg, 1.1 mmol). **A4** (564 mg, 0.89 mmol, 81% yield) was obtained as a white powder.

<u>$^{1}$H NMR</u> (400 MHz, $CDCl_3$) δ 8.24 – 8.18 (m, 4H), 8.06 (d, *J* = 8.2 Hz, 2H), 7.97 – 7.82 (m, 8H), 7.61 – 7.55 (m, 2H), 7.54 – 7.48 (m, 6H), 7.44 – 7.35 (m, 2H).
<u>$^{13}$C NMR</u> (126 MHz, $CDCl_3$) δ 134.7, 133.3, 132.8, 132.0, 131.7, 129.3, 128.6, 128.4, 128.0, 127.8, 127.7, 127.1, 126.8, 126.4, 126.3, 126.2.

$^{31}$P NMR (162 MHz, $CDCl_3$) δ 96.3.

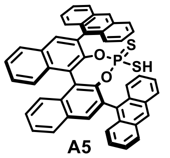

A5

**(11bS)-2,6-di(anthracen-9-yl)-4-mercaptodinaphtho[2,1-d:1',2'-f][1,3,2]dioxaphosphepine 4-sulfide (A5)** Prepared according to GP1 using (1S)-3,3′-Di-9-anthracenyl[1,1′-binaphthalene]-2,2′-diol (585 mg, 0.92 mmol). **A5** (543 mg, 0.74 mmol, 81% yield) was obtained as a white powder.

$^{1}$H NMR (400 MHz, $CDCl_3$) δ 8.54 – 8.49 (m, 2H), 8.17 (s, 2H), 8.08 – 7.95 (m, 8H), 7.74 – 7.61 (m, 6H), 7.57 – 7.50 (m, 2H), 7.46 – 7.37 (m, 4H), 7.36 – 7.26 (m, 4H).

$^{13}$C NMR (126 MHz, $CDCl_3$) δ 134.7, 131.6, 131.0, 131.0, 130.9, 130.3, 128.7, 128.6, 128.4, 128.1, 127.5, 127.2, 126.5, 126.3, 126.2, 125.1, 125.1, 125.0.

$^{31}$P NMR (122 MHz, $CDCl_3$) δ 93.2.

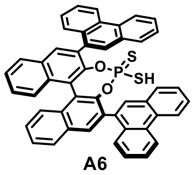

A6

**(11bS)-4-mercapto-2,6-di(phenanthren-9-yl)dinaphtho[2,1-d:1',2'-f][1,3,2]dioxaphosphepine 4-sulfide (A6)** Prepared according to GP1 using (1S)-3,3′-Di-9-phenanthrenyl[1,1′-binaphthalene]-2,2′-diol (540 mg, 0.85 mmol). **A6** (460 mg, 0.63 mmol, 74% yield) was obtained as a white powder.

$^{1}$H NMR (500 MHz, $CDCl_3$) δ 8.84 – 8.71 (m, 4H), 8.25 – 8.02 (m, 5H), 8.00 – 7.89 (m, 3H), 7.75 – 7.58 (m, 12H), 7.58 – 7.43 (m, 4H).

$^{31}$P NMR (202 MHz, $CDCl_3$) δ 96.5.

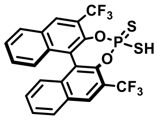

A7

**(11bS)-4-mercapto-2,6-bis(trifluoromethyl)dinaphtho[2,1-d:1',2'-f][1,3,2]dioxaphosphepine 4-sulfide (A7)** Prepared according to GP1 using (1S)-3,3′-Bis(trifluoromethyl)[1,1′-binaphthalene]-2,2′-diol (470 mg, 1.1 mmol). **A7** (487 mg, 0.94 mmol, 85% yield) was obtained as a white powder

$^{1}$H NMR (500 MHz, $CDCl_3$) δ 8.46 (s, 2H), 8.09 (d, $J$ = 8.2 Hz, 2H), 7.63 (t, $J$ = 7.6 Hz, 2H), 7.43 (t, $J$ = 8.4 Hz, 1H), 7.16 (d, $J$ = 8.6 Hz, 2H).

$^{13}$C NMR (126 MHz, $CDCl_3$) δ 143.1, 143.0, 133.9, 130.3, 130.3, 130.1, 129.5, 129.3, 127.4, 126.9, 123.6, 123.6, 121.8, 121.4.

$^{19}$F NMR (471 MHz, $CDCl_3$) δ -59.1.

$^{31}$P NMR (202 MHz, $CDCl_3$) δ 97.7.

# D. Synthesis of the Substrates

## D.1 Synthesis of pyridines 1

### D.1.1 Synthesis of 4-(pyridin-3-yl)benzonitrile 1i

Pyridine **1i** was prepared following a reported procedure (Scheme S3).[4]

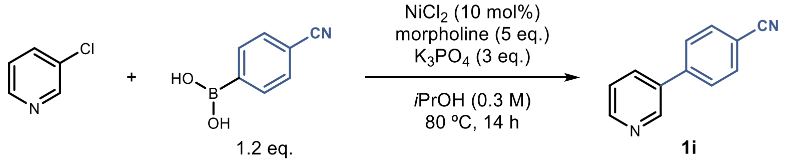

**Scheme S3.** Synthesis of pyridine **1i**.

To a mixture of 3-chloropyridine (95 µL, 1 mmol), $K_3PO_4$ (637 mg, 3 equiv.), $NiCl_2$ (13 mg, 0.1 equiv.), and (4-cyanophenyl)boronic acid (176 mg, 1.2 equiv.) was added morpholine (431 µL, 5 equiv.) followed by 2-propanol (3 mL, 0.3 M). The mixture was stirred at 80 °C under nitrogen. After 14 h, the mixture was diluted with ethyl acetate and $H_2O$. The organic layer was separated and the aqueous layer was extracted with ethyl acetate. The combined organic layers were washed with brine, dried over MgSO , filtered, and concentrated under reduced pressure. The crude mixture was purified by column chromatography ($SiO_2$, 20:80 EtOAc/hexanes) to afford **1i** as a white solid (63 mg, 35% yield) which displayed spectroscopic data consistent with those reported previously.[4]

$^1$H NMR (300 MHz, $CDCl_3$) δ 8.86 (s, 1H), 8.68 (d, *J* = 4.9 Hz, 1H), 7.92 (ddd, *J* = 8.0, 2.4, 1.6 Hz, 1H), 7.82 – 7.76 (m, 2H), 7.73 – 7.67 (m, 2H), 7.45 (dd, *J* = 7.9, 4.8 Hz, 1H).

$^{13}$C NMR (101 MHz, $CDCl_3$) δ 149.7, 148.1, 142.2, 134.6, 134.4, 132.8, 127.7, 123.7, 118.4, 111.8.

### D.1.2 Synthesis of nicotinamides and nicotinates

***GP2* – General procedure for the synthesis of nicotinamides and nicotinates from nicotinoyl chloride**

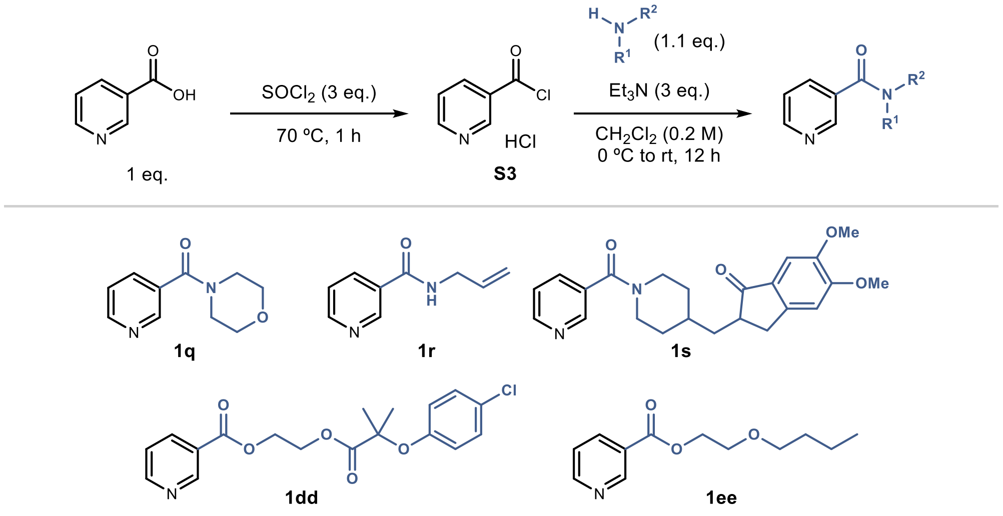

**Figure S3.** Synthesis of nicotinamides and nicotinic esters from nicotinoyl hydrochloride.

A suspension of nicotinic acid (1 equiv.) in thionyl chloride (3 equiv.) was stirred at 70 °C for 1 h. The mixture was cooled-down to room temperature and concentrated under reduced pressure to

afford the crude nicotinoyl chloride hydrochloride **S3** as a white solid which was used in the next step without further purification.

To an ice-cold suspension of **S3** (1 equiv.) in dichloromethane (0.2 M) was added the amine or alcohol (1.1 equiv.) in portions, followed by dropwise addition of triethylamine (3 equiv.). The reaction was allowed to warm-up to room temperature over 12 h. The mixture was washed with water, brine, dried over magnesium sulfate, filtered, and concentrated under reduced pressure. Purification by column chromatography afforded the pure nicotinamides.

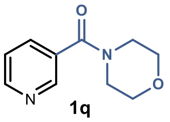

**1q**

**morpholino(pyridin-3-yl)methanone (1q)**

Prepared according to GP2 using nicotinic acid (985 mg, 8.0 mmol), and morpholine (769 µL, 1.1 equiv.). Purification by column chromatography ($SiO_2$, 1:2:97 $Et_3N$/MeOH/$CH_2Cl_2$) afforded product **1q** as a colorless oil (1.1 g, 68% yield), which displayed spectroscopic data consistent with those reported previously.[5]

<u>$^1$H NMR</u> (300 MHz, $CDCl_3$) δ 8.70 – 8.65 (m, 2H), 7.76 (dt, *J* = 7.8, 2.0 Hz, 1H), 7.37 (ddd, *J* = 7.8, 4.9, 0.9 Hz, 1H), 3.89 – 3.39 (m, 8H).
<u>$^{13}$C NMR</u> (101 MHz, $CDCl_3$) δ 167.8, 151.0, 148.0, 135.1, 131.2, 123.5, 66.8, 46.3, 30.9, 8.6.

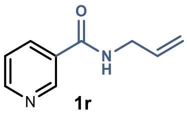

**1r**

***N*-allyl-nicotinamide (1r)**

Prepared according GP2 using nicotinic acid (616 mg, 5 mmol) and allylamine (636 µL, 1.7 equiv.). Purification by column chromatography ($SiO_2$, 30:70 EtOAc/hexanes) afforded product **1r** as a colorless oil (581 mg, 72% yield) which displayed spectroscopic data consistent with those reported previously.[6]

<u>$^1$H NMR</u> (400 MHz, $CDCl_3$) δ 8.98 (d, *J* = 1.4 Hz, 1H), 8.66 (dd, *J* = 4.9, 1.7 Hz, 1H), 8.12 (dt, *J* = 7.9, 2.0 Hz, 1H), 7.35 (ddd, *J* = 7.9, 4.9, 0.9 Hz, 1H), 6.90 (br s, 1H), 5.90 (ddt, *J* = 17.2, 10.2, 5.7 Hz, 1H), 5.23 (dq, *J* = 17.1, 1.6 Hz, 1H), 5.16 (dq, *J* = 10.3, 1.4 Hz, 1H), 4.06 (tt, *J* = 5.7, 1.6 Hz, 2H).
<u>$^{13}$C NMR</u> (101 MHz, $CDCl_3$) δ 165.6, 152.2, 148.0, 135.4, 133.9, 130.3, 123.6, 117.0, 42.6.

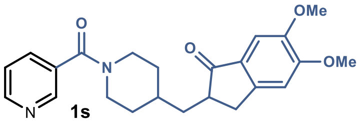

**1s**

**5,6-dimethoxy-2-((1-nicotinoylpiperidin-4-yl)methyl)-2,3-dihydro-1H-inden-1-one (1s)**

Prepared according to GP2 using nicotinic acid (369 mg, 3.0 mmol), and 5,6-dimethoxy-2-(piperidin-4-ylmethyl)-2,3-dihydro-1H-inden-1-one (955 mg, 1.1 equiv.). Purification by column chromatography ($SiO_2$, 1:1:98 $Et_3N$/MeOH/$CH_2Cl_2$) afforded product **1s** as a white solid (794 mg, 67% yield).

<u>$^1$H NMR</u> (400 MHz, $CDCl_3$) δ 8.67 (s, 2H), 7.76 (dt, *J* = 7.8, 1.9 Hz, 1H), 7.37 (dd, *J* = 7.8, 4.9 Hz, 1H), 7.17 (s, 1H), 6.86 (s, 1H), 4.88 – 4.62 (m, 1H), 3.97 (s, 3H), 3.91 (s, 3H), 3.83 – 3.63 (m, 1H), 3.28 (dd, *J* = 17.3, 8.0 Hz, 1H), 3.17 – 2.99 (m, 1H), 2.92 – 2.78 (m, 1H), 2.79 – 2.65 (m, 2H), 2.02 – 1.84 (m, 3H), 1.81 – 1.69 (m, 2H), 1.49 – 1.34 (m, 2H).
<u>$^{13}$C NMR</u> (126 MHz, $CDCl_3$) δ 207.2, 167.6, 155.6, 150.6, 149.6, 148.6, 147.8, 134.9, 132.1, 129.2, 123.5, 107.3, 104.4, 68.5, 56.3, 56.1, 48.1, 45.0, 42.6, 38.6, 34.5, 33.4.
<u>HRMS</u> (ESI$^+$) Calculated for $C_{23}H_{26}N_2NaO_4$ $[M+Na]^+$: 417.1785 found: 417.1786.

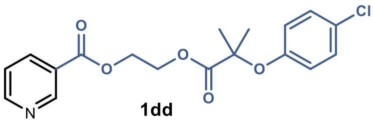

**1dd**

**Etofibrate (1dd)**

Prepared according to GP2 using nicotinic acid (222 mg, 1.8 mmol), and 2-hydroxyethyl 2-(4-chlorophenoxy)-2-

methylpropanoate (512 mg, 1.1 equiv.). Purification by column chromatography ($SiO_2$, 1:10:89 $Et_3N$/EtOAc/hexanes) afforded product **1dd** as a yellow oil (473 mg, 72% yield).

$^1$H NMR (300 MHz, $CDCl_3$) δ 9.17 (dd, *J* = 2.2, 0.9 Hz, 1H), 8.81 (dd, *J* = 4.9, 1.7 Hz, 1H), 8.15 (ddd, *J* = 8.0, 2.2, 1.8 Hz, 1H), 7.40 (ddd, *J* = 8.0, 4.9, 0.9 Hz, 1H), 7.17 – 7.08 (m, 2H), 6.83 – 6.74 (m, 2H), 4.56 (m, 4H), 1.62 (s, 6H).
$^{13}$C NMR (126 MHz, $CDCl_3$) δ 173.9, 164.9, 153.9, 153.7, 150.9, 137.1, 129.1, 127.2, 125.5, 123.4, 120.2, 79.3, 63.0, 62.8, 25.3.

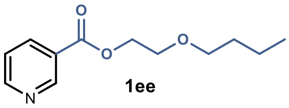
1ee

**Nicoboxil (1ee)**

Prepared according to GP2 using nicotinic acid (222 mg, 1.8 mmol), and 2-butoxyethanol (260 µL, 1.1 equiv.). Purification by column chromatography ($SiO_2$, 1:10:89 $Et_3N$/EtOAc/hexanes) afforded product **1ee** as a yellow oil (199.4 mg, 50% yield).

$^1$H NMR (300 MHz, $CDCl_3$) δ 9.27 (d, *J* = 1.8 Hz, 1H), 8.80 (dd, *J* = 4.9, 1.8 Hz, 1H), 8.34 (dt, *J* = 8.0, 2.0 Hz, 1H), 7.42 (dd, *J* = 8.0, 4.9 Hz, 1H), 4.55 – 4.50 (m, 2H), 3.83 – 3.77 (m, 2H), 3.54 (t, *J* = 6.6 Hz, 2H), 1.66 – 1.55 (m, 2H), 1.48 – 1.34 (m, 2H), 0.94 (t, *J* = 7.3 Hz, 3H).
$^{13}$C NMR (101 MHz, $CDCl_3$) δ 165.3, 153.4, 151.0, 137.2, 126.1, 123.3, 71.3, 68.4, 64.6, 31.7, 19.3, 13.9.
HRMS (ESI$^+$) Calculated for $C_{12}H_{18}NO_3$ $[M+H]^+$: 224.1281 found: 224.1279.

### D.1.3 Synthesis of nicotinates 1m-n

### *GP3* – General procedure for the synthesis of nicotinates using DCC

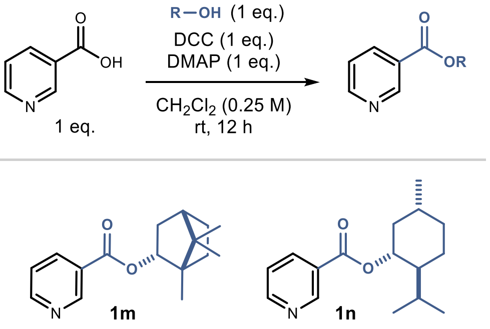


**Figure S4.** Synthesis of nicotinates **1m-n**.

To a mixture of nicotinic acid (1.0 equiv.) and alcohol (1.0 equiv.) in dichloromethane (0.25 M) were added DCC (2.06 g, 10 mmol, 1.0 equiv.), followed by DMAP (1.22 g, 10 mmol, 1.0 equiv.) The resulting mixture was stirred at room temperature for 12 h. The solution was concentrated under reduced pressure. Purification by column chromatography afforded the pure nicotinates.

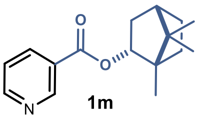
1m

**(1*R*,2*S*,4*R*)-1,7,7-trimethylbicyclo[2.2.1]heptan-2-yl nicotinate (1m)**
Prepared according to GP3 using nicotinic acid (1.23 g, 10.0 mmol), and L-borneol (1.54 g, 1.0 equiv.). Purification by column chromatography ($SiO_2$, 80:20 hexanes/EtOAc) afforded product **1m** as a colorless oil (1.27 g, 78% yield) which displayed spectroscopic data consistent with those reported previously.[7]

$^{1}$H NMR (400 MHz, $CDCl_3$) δ 9.26 (d, J = 2.2 Hz, 1H), 8.78 (dd, J = 4.8, 1.8 Hz, 1H), 8.31 (dt, J = 8.0, 4.8 Hz, 1H), 7.50 – 7.35 (m, 1H), 5.15 (ddd, J = 10.0, 3.4, 2.2 Hz, 1H), 2.59 – 2.39 (m, 1H), 2.03 – 2.17 (m, 1H), 1.87 – 1.73 (m, 2H), 1.49 – 1.38 (m, 1H), 1.36 – 1.28 (m, 1H), 1.14 (dd, J = 13.8, 3.6 Hz, 1H), 0.98 (s, 3H), 0.93 (d, J = 1.2 Hz, 6H).

$^{13}$C NMR (100 MHz, $CDCl_3$) δ 165.6, 153.4, 151.0, 137.1, 126.8, 123.4, 81.3, 49.2, 48.0, 45.0, 37.0, 28.2, 27.5, 19.8, 19.0, 13.7.

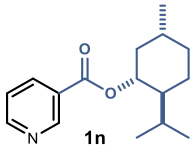


**(1*R*,2*S*,5*R*)-2-Isopropyl-5-methylcyclohexyl nicotinate (1n).**

Prepared according to GP3 using nicotinic acid (1.23 g, 10.0 mmol), and L-menthol (1.56 g, 1.0 equiv.). Purification by column chromatography ($SiO_2$, 80:20 hexanes/EtOAc) afforded product **1n** as a colorless oil (0.95 g, 73% yield) which displayed spectroscopic data consistent with those reported previously.[8]

$^{1}$H NMR (400 MHz, $CDCl_3$) δ 9.20 (d, J = 2.0 Hz, 1H), 8.74 (dd, J = 5.0, 1.8 Hz, 1H), 8.27 (dt, J = 8.0, 2.0 Hz, 1H), 7.37 (dd, J = 7.9, 4.9 Hz, 1H), 4.94 (td, J = 10.8, 4.4 Hz, 1H), 2.14 – 2.07 (m, 1H), 1.99 – 1.86 (m, 1H), 1.80 – 1.67 (m, 2H), 1.65 – 1.30 (m, 3H), 1.16 – 1.03 (m, 2H), 0.94 – 0.89 (m, 6H), 0.80 – 0.74 (m, 3H).

$^{13}$C NMR (100 MHz, $CDCl_3$) δ 164.9, 153.3, 151.0, 137.1, 126.7, 123.3, 75.6, 47.2, 41.0, 34.3, 31.5, 26.6, 23.6, 22.1, 20.8.

### D.1.4 Synthesis of amino acid-deriving nicotinamides 1t-w

### *GP4* – General procedure for the synthesis of amino acid-derived nicotinamides

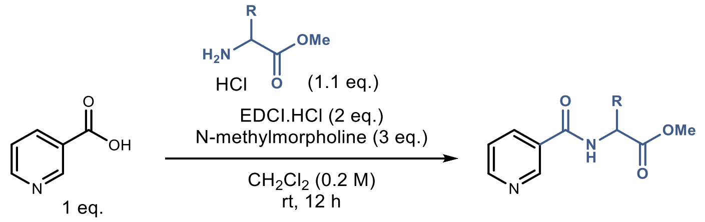


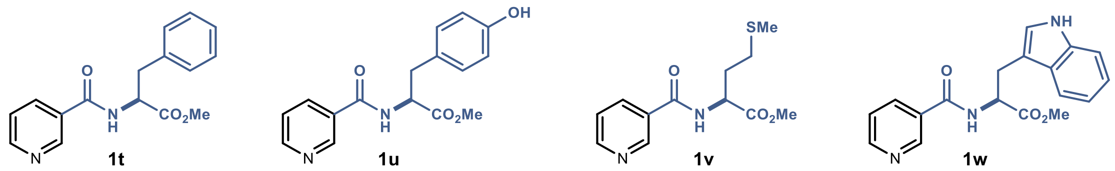


**Figure S5.** Synthesis of amino acids-derived nicotinamides **1t-w**.

To a suspension of nicotinic acid (1 equiv.) in dichloromethane (0.2 M) was added EDCI hydrochloride (2 equiv.), followed by *N*-methylmorpholine (3 equiv.) and the hydrochloride salt of the amino acid methyl ester (1.1 equiv.). The reaction mixture was stirred at room temperature overnight. The mixture was diluted with dichloromethane, washed with water, brine, dried over magnesium sulfate, filtered and concentrated under reduced pressure. Purification by column chromatography afforded the pure nicotinamides.

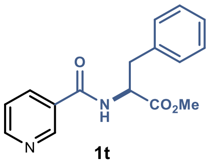

**1t**

### methyl nicotinoyl-L-phenylalaninate (1t)

Prepared according to GP4 using nicotinic acid (1.38 g, 11.2 mmol), and methyl L-phenylalaninate (2.00 g, 1.0 equiv. Purification by column chromatography ($SiO_2$, 20:80 hexanes/EtOAc to 100% EtOAc) afforded product **1t** as a yellow oil (2.41 g, 76% yield) which displayed spectroscopic data consistent with those reported previously.[7]

$^{1}$H NMR (400 MHz, $CDCl_3$) δ 8.92 (d, *J* = 1.9 Hz, 1H), 8.74 (dd, *J* = 4.9, 1.7 Hz, 1H), 8.06 (dt, *J* = 7.9, 2.1 Hz, 1H), 7.39 (ddd, *J* = 7.9, 4.8, 0.9 Hz, 1H), 7.35 – 7.25 (m, 3H), 7.16 – 7.09 (m, 2H), 6.61 (d, *J* = 7.5 Hz, 1H), 5.09 (dt, *J* = 7.6, 5.6 Hz, 1H), 3.79 (s, 3H), 3.27 (qd, *J* = 13.9, 5.6 Hz, 2H).
$^{13}$C NMR (101 MHz, $CDCl_3$) δ 171.9, 165.1, 152.5, 148.0, 135.7, 135.4, 129.4, 128.9, 127.5, 123.7, 53.7, 52.7, 38.0.

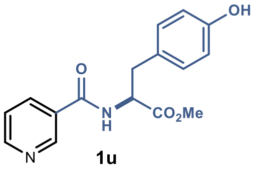

**1u**

### methyl nicotinoyl-L-tyrosinate (1u)

Prepared according to GP4 using nicotinic acid (616 mg, 5.0 mmol), and methyl L-tyrosinate (976 mg, 1.0 equiv.). Purification by column chromatography ($SiO_2$, 20:80 EtOAc /hexanes to 100% EtOAc) afforded product **1u** as a white solid (852 mg, 57% yield) which displayed spectroscopic data consistent with those reported previously.[9]

$^{1}$H NMR (400 MHz, $CDCl_3$) δ 8.91 (s, 1H), 8.74 (d, *J* = 4.9 Hz, 1H), 8.09 (dt, *J* = 7.9, 2.0 Hz, 1H), 7.41 (dd, *J* = 8.0, 4.8 Hz, 1H), 7.02 – 6.95 (m, 2H), 6.80 – 6.73 (m, 2H), 6.60 (d, *J* = 7.6 Hz, 1H), 5.05 (dt, *J* = 7.7, 5.6 Hz, 1H), 3.79 (s, 3H), 3.20 (qd, *J* = 14.1, 5.6 Hz, 2H).
$^{13}$C NMR (101 MHz, MeOD) δ 173.5, 167.9, 157.5, 152.8, 149.2, 137.1, 131.7, 131.2, 128.9, 125.1, 116.3, 56.2, 52.8, 37.4.

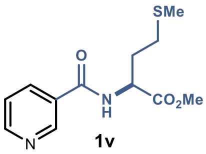

**1v**

### methyl nicotinoyl-L-methioninate (1v)

Prepared according to GP4 using nicotinic acid (616 mg, 5.0 mmol), and methyl L-methioninate (816 mg, 1.0 equiv.). Purification by column chromatography ($SiO_2$, 20:80 EtOAc /hexanes to 100% EtOAc) afforded product **1v** as a colorless oil (593 mg, 44% yield).

$^{1}$H NMR (400 MHz, $CDCl_3$) δ 9.07 – 9.01 (m, 1H), 8.74 (dd, *J* = 5.0, 1.7 Hz, 1H), 8.14 (dt, *J* = 7.9, 2.0 Hz, 1H), 7.40 (ddd, *J* = 8.0, 4.8, 0.9 Hz, 1H), 7.19 (d, *J* = 7.7 Hz, 1H), 4.93 (td, *J* = 7.3, 5.0 Hz, 1H), 3.80 (s, 3H), 2.60 (t, *J* = 7.2 Hz, 2H), 2.29 (dtd, *J* = 14.7, 7.4, 5.1 Hz, 1H), 2.21 – 2.09 (m, 1H), 1.86 (s, 3H).
$^{13}$C NMR (101 MHz, $CDCl_3$) δ 172.4, 165.4, 152.7, 148.3, 135.4, 129.6, 123.7, 52.9, 52.4, 31.4, 30.3, 15.7.
HRMS ($ESI^+$) Calculated for $C_{12}H_{17}N_2O_3S$ $[M+H]^+$: 269.0954 found: 269.0967.

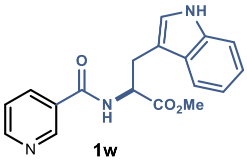

**1w**

### methyl nicotinoyl-L-tryptophanate (1w)

Prepared according to GP4 using nicotinic acid (616 mg, 5.0 mmol), and methyl L-tryptophanate (1.09 g, 1.0 equiv.). Purification by column chromatography ($SiO_2$, 100% EtOAc) afforded product **1w** as a yellow oil (1.26 g, 78% yield) which displayed spectroscopic data consistent with those reported previously.[10]

$^{1}$H NMR (400 MHz, $CDCl_3$) δ 8.84 (d, *J* = 1.5 Hz, 1H), 8.68 (dd, *J* = 4.8, 1.7 Hz, 1H), 8.32 (s, 1H), 7.99 (dt, *J* = 7.9, 2.0 Hz, 1H), 7.53 (dd, *J* = 7.9, 1.1 Hz, 1H), 7.35 (dt, *J* = 8.2, 0.9 Hz, 1H), 7.32 (ddd, *J* = 7.9, 4.9, 0.9 Hz, 1H), 7.18 (ddd, *J* = 8.2, 7.0, 1.2 Hz, 1H), 7.08 (ddd, *J* = 8.0, 7.0,

1.0 Hz, 1H), 6.75 (d, $J$ = 7.7 Hz, 1H), 5.14 (dt, $J$ = 7.7, 5.2 Hz, 1H), 3.75 (s, 3H), 3.52 – 3.40 (m, 2H). $^{13}$C NMR (101 MHz, $CDCl_3$) δ 172.3, 165.2, 152.4, 148.2, 136.3, 135.3, 129.8, 127.7, 123.6, 123.0, 122.6, 120.0, 118.6, 111.6, 109.9, 53.7, 52.7, 27.6.

### D.1.5 Synthesis of the Radical Clock Substrates

Diphenyldiazomethane was prepared following a reported procedure.[11]

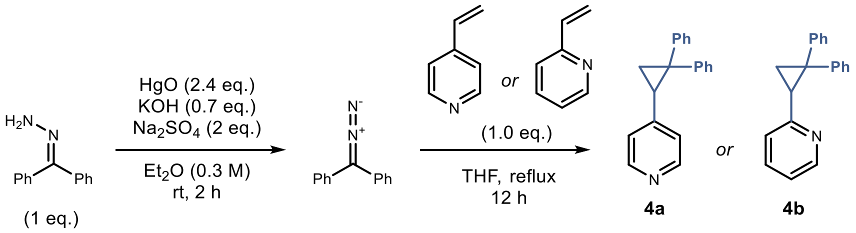


**Scheme S3.** Synthesis of 4-(2,2-diphenylcyclopropyl)pyridines.

**Diphenyldiazomethane.** To benzophenone hydrazone (2.50 g, 12.7 mmol), anhydrous sodium sulfate (3.62 g, 2 equiv.), yellow mercuric oxide (II) (6.62 g, 2.4 equiv.), and potassium hydroxide (500 mg, 0.7 equiv., in 1 mL EtOH), was added 40 mL of diethyl ether. The suspension was stirred at room temperature for 2 hours. The reaction mixture was filtered, and the filtrate concentrated under reduced pressure. The resulting dark red oil was dissolved in hexanes at 50 °C and filtered again. The solvent was evaporated under reduced pressure to obtain a dark red oil that was left to freeze overnight. Dark red crystals (2.47 g, quantitative yield) of diphenyldiazomethane formed when warmed-up to room temperature.

4-(2,2-diphenylcyclopropyl)pyridines **4a** and **4b** were synthesized adapting a procedure from the literature.[12]

### *GP4* – General procedure for the synthesis of (2,2-diphenylcyclopropyl)pyridines

Diphenyldiazomethane (1.17 g, 6.0 mmol) and 4-vinylpyridine (1 equiv.) were dissolved in THF (10 mL, 0.6 M). The solution was stirred under reflux overnight. After cooling down to room temperature, the dark solution was diluted with hexanes and filtered to remove the precipitate. The filtrate was concentrated under reduced pressure and the crude mixture purified by column chromatography to afford the pure (2,2-diphenylcyclopropyl)pyridines.

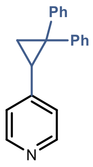


**4a**

**4-(2,2-diphenylcyclopropyl)pyridine (4a)** Prepared according to GP4 using 4-vinylpyridine (643 µL, 6.0 mmol). Purification by column chromatography ($SiO_2$, 1:40:59 $Et_3N$/EtOAc/hexanes) afforded **4a** as a light-yellow wax (1.1 g, 65% yield).

$^{1}$H NMR (400 MHz, $CDCl_3$) δ 8.29 (d, $J$ = 6.2 Hz, 2H), 7.31 (d, $J$ = 4.3 Hz, 4H), 7.25 – 7.10 (m, 6H), 6.74 (d, $J$ = 6.2 Hz, 2H), 2.81 (dd, $J$ = 8.8, 6.4 Hz, 1H), 2.05 (dd, $J$ = 6.4, 5.5 Hz, 1H), 1.93 (dd, $J$ = 8.8, 5.5 Hz, 1H).

$^{13}$C NMR (101 MHz, $CDCl_3$) δ 148.9, 148.5, 146.0, 139.2, 131.0, 128.5, 128.3, 127.3, 126.8, 126.3, 123.0, 40.8, 31.4, 21.5.

HRMS (ESI$^+$) Calculated for $C_{20}H_{18}N$ $[M+H]^+$: 272.1434 found: 272.1434.

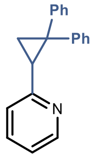

4b

**2-(2,2-diphenylcyclopropyl)pyridine (4b)** Prepared according to GP4 using 2-vinylpyridine (645 µL, 6.0 mmol). Purification by column chromatography ($SiO_2$, 1:40:59 $Et_3N$/EtOAc/hexanes) afforded **4b** as a light-yellow wax (1.0 g, 63% yield).

$^1$H NMR (400 MHz, $CDCl_3$) δ 8.35 (ddd, $J$ = 4.9, 1.9, 0.9 Hz, 1H), 7.41 – 7.34 (m, 3H), 7.32 – 7.26 (m, 2H), 7.22 – 7.16 (m, 1H), 7.16 – 7.06 (m, 5H), 6.95 (ddd, $J$ = 7.5, 4.9, 1.2 Hz, 1H), 6.80 (dt, $J$ = 7.9, 1.1 Hz, 1H), 3.13 (dd, $J$ = 8.7, 6.5 Hz, 1H), 2.34 (dd, $J$ = 6.5, 5.1 Hz, 1H), 1.85 (dd, $J$ = 8.7, 5.1 Hz, 1H).

$^{13}$C NMR (101 MHz, $CDCl_3$) δ 158.4, 148.6, 146.5, 140.2, 135.3, 131.0, 128.3, 127.9, 127.5, 126.2, 126.0, 122.3, 120.6, 40.2, 33.8, 20.3.

HRMS (ESI$^+$) Calculated for $C_{20}H_{18}N$ $[M+H]^+$: 272.1434 found: 272.1433.

### D.2 Synthesis of tert-butyl pyrimidin-2-ylcarbamate 1bb

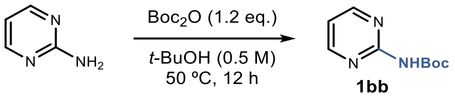


**Scheme S4.** Synthesis of *N*-Boc-2-aminopyrimidine **1bb**.

2-Aminopyrimidine (475 mg, 5.0 mmol) and di-tert-butyl carbonate (1.31 g, 1.2 equiv.) were dissolved in tert-butyl alcohol (10 mL, 0.5 M). The reaction was stirred at 50 ºC overnight. The solvent was then removed under reduced pressure and the residue dissolved in ethyl acetate. The organic layer was washed three times with brine and dried over MgSO4, and the solvent was removed to afford **1bb** as a white solid (486 mg, 50% yield) which displayed spectroscopic data consistent with those reported previously.[13]

$^1$H NMR (300 MHz, $CDCl_3$) δ 8.62 (d, $J$ = 4.9 Hz, 2H), 7.99 (s, 1H), 6.98 (t, $J$ = 4.9 Hz, 1H), 1.57 (s, 9H).

$^{13}$C NMR (101 MHz, $CDCl_3$) δ 158.4, 157.9, 150.5, 115.7, 81.6, 28.2.

### D.3 Synthesis of pyridinium and collidinium salts

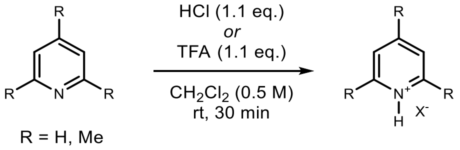


**Scheme S5.** Synthesis of pyridinium and 2,4,6-collidinium salts.

To a solution of the azine (5.5 mmol) in $CH_2Cl_2$ (10 mL, 0.5 M) was added conc. HCl (400 µL, 1.1 equiv.) or trifluoroacetic acid (424 µL, 1.1 equiv.) dropwise. After stirring for 30 min, the solvent was removed under reduced pressure to afford the pure pyridine hydrochloride salt.

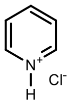

1a.HCl

**Pyridinium hydrochloride (1a.HCl)** Using pyridine (445 µL, 5.5 mmol). **1a.HCl** was obtained as a white powder (460 mg, 72% yield).

$^1$H NMR (400 MHz, $CDCl_3$) δ 8.94 (d, $J$ = 5.5 Hz, 2H), 8.51 (t, $J$ = 7.7 Hz, 1H), 8.04 (t, $J$ = 6.9 Hz, 2H).

$^{13}$C NMR (101 MHz, $CDCl_3$) δ 145.8, 141.2, 127.3.

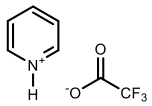

1a.TFA

**Pyridinium trifluoroacetate (1a.TFA)** Using pyridine (445 µL, 5.5 mmol). **1a.TFA** was obtained as a white powder (931 mg, 88% yield).

$^{1}$H NMR (400 MHz, $CDCl_3$) δ 8.87 (br s, 2H), 8.27 (tt, *J* = 7.8, 1.6 Hz, 1H), 7.81 (dd, *J* = 7.8, 6.4 Hz, 2H).

$^{13}$C NMR (101 MHz, $CDCl_3$) δ 143.4, 143.2, 126.3.

$^{19}$F{$^{1}$H} NMR (376 MHz, $CDCl_3$) δ -75.8.

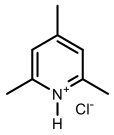

collidine.HCl

**2,4,6-Collidinium hydrochloride (collidine·HCl)** Using 2,4,6-collidine (728 µL, 5.5 mmol). **Collidine·HCl** was obtained as a white powder (620 mg, 71% yield).

$^{1}$H NMR (400 MHz, $CDCl_3$) δ 7.22 (s, 2H), 2.93 (s, 6H), 2.54 (s, 3H).

$^{13}$C NMR (101 MHz, $CDCl_3$) δ 157.7, 153.1, 125.1, 22.0, 19.3.

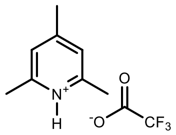

collidine.TFA

**2,4,6-Collidinium trifluoroacetate (collidine·TFA)** Using 2,4,6-collidine (728 µL, 5.5 mmol). **Collidine·TFA** was obtained as a white powder (1.23, 95% yield).

$^{1}$H NMR (400 MHz, $CDCl_3$) δ 7.19 (s, 2H), 2.74 (s, 6H), 2.50 (d, *J* = 0.7 Hz, 3H).

$^{13}$C NMR (101 MHz, $CDCl_3$) δ 157.6, 153.2, 124.9, 21.9, 19.2.

$^{19}$F NMR (376 MHz, $CDCl_3$) δ -75.9.

# E. Experimental Procedures

## E.1 Experimental setups

Two different photochemical setups were used in this study, for UV- or blue light irradiation. Figure S6 depicts the emission spectra of the light sources used in these setups: a 365PF EvoluChem$^{TM}$ LED spotlight from HepatoChem, and a commercial 14 W 455 nm LEDs strip.

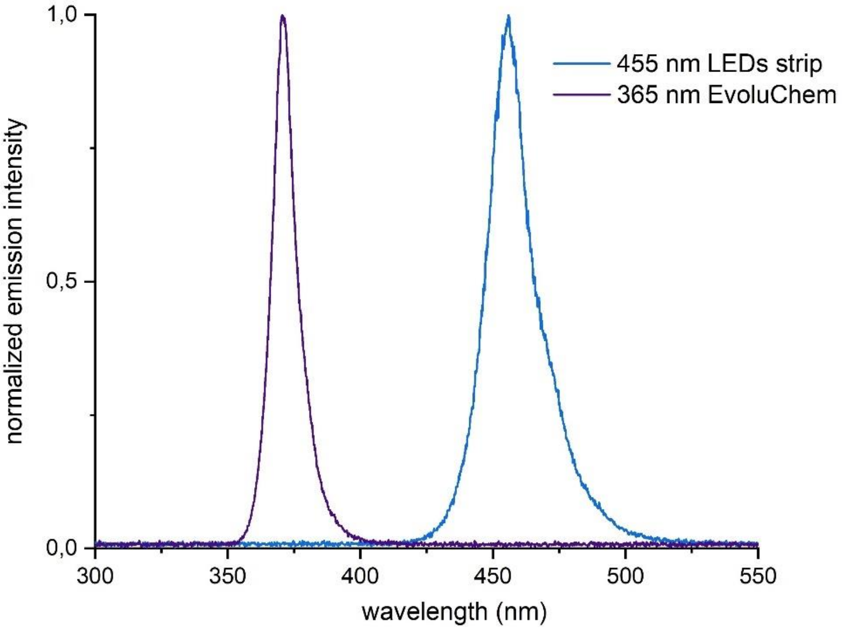


**Figure S6.** Emission spectra of the light sources used in this study.

### E.1.1 365 nm setup

The setup used for the reactions under UV-light consisted of two 365PF EvoluChem$^{TM}$ LED spotlights placed on two opposite sides of a rack. The vials were placed at ~1 cm of the lamps, with a maximum of 3 vials per lamp, allowing 6 reactions to run at the same time. A fan was placed on top of the vials, in order to dissipate the heat, thus maintaining the temperature at ~35 ºC (Figure S7).

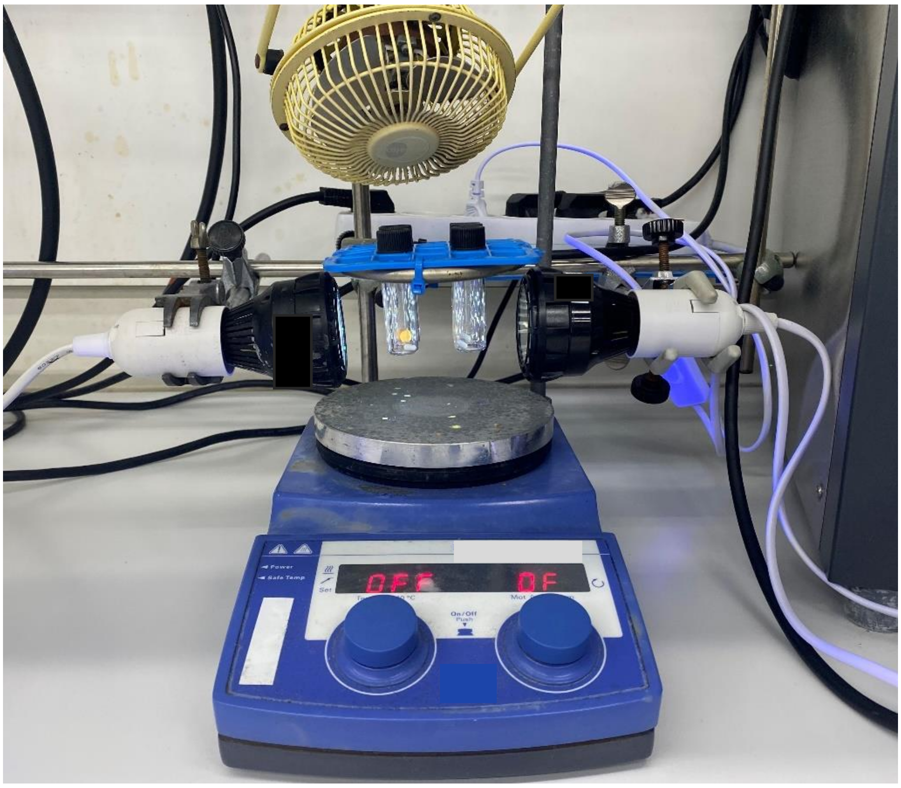

**Figure S7.** 365 nm setup in operation.

### E.1.2 455 nm photoreactor

The photoreactor used for the reactions under blue light consisted of a 12.5 cm diameter jar fitted with 4 standard B29 size quickfit-glass joints arranged around a central B29 size joint (Figure S6). A commercial 1-meter LEDs strip was wrapped around the jar, followed by a layer of aluminium foil and cotton for insulation. An inlet/outlet system provided circulation of liquid (ethylene glycol/water 1:1 mixture) from a Huber Minichiller 300 inside the jar. This setup allowed the performance of reactions at temperatures ranging from –20 ºC to 80 ºC with accurate control of the reaction temperature (± 1ºC, Figure S8).

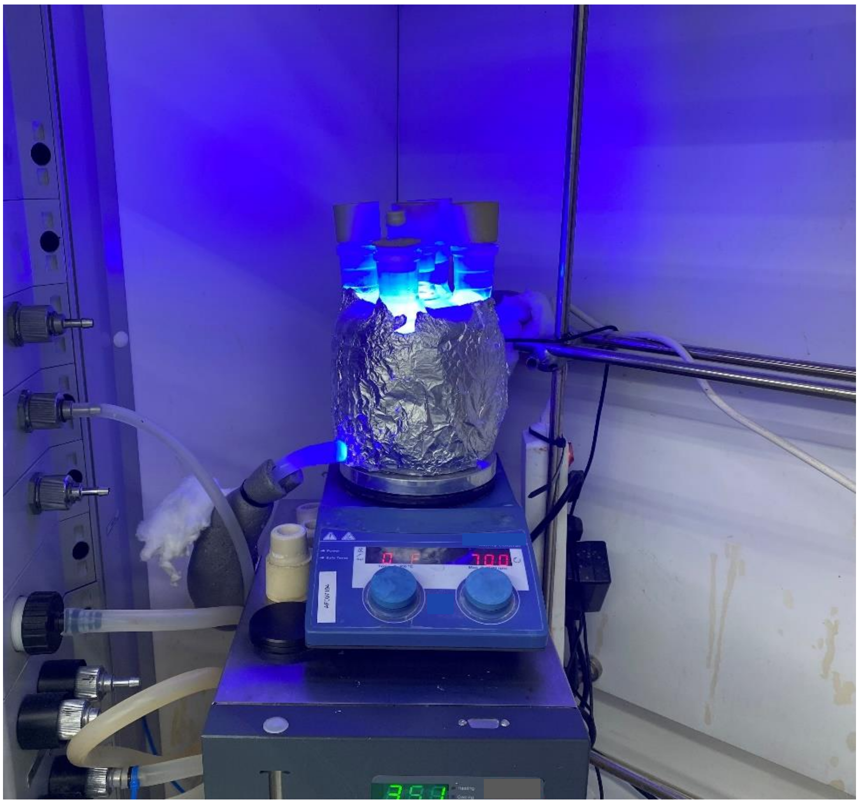

**Figure S8**. Fully assembled temperature-controlled photoreactor in operation.

In order to maintain consistent illumination between different experiments, only the four external positions were used to perform reactions. The central position was used to monitor the temperature using a thermometer inside an inserted Schlenk tube, ensuring that the reaction mixtures were at the desired temperature.

## E.2 Direct allylation of pyridines

### E.2.1 General Procedure A

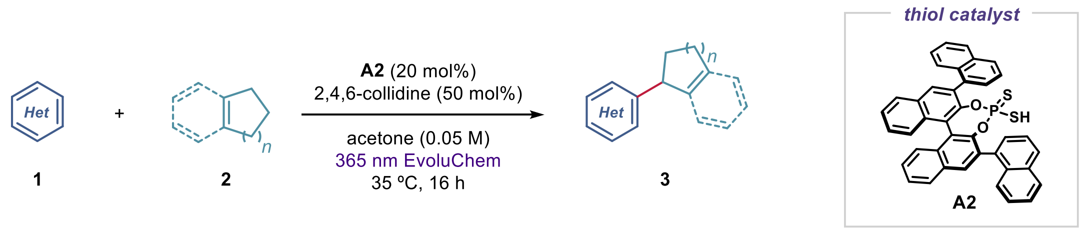


To an argon-purged glass vial, containing the dithiophosphoric acid catalyst **A2** (25.3 mg, 0.04 mmol), and pyridine derivative **1** (0.2 mmol), was added 2,4,6-collidine (13.2 µL, 0.1 mmol), followed by the allylic precursor **2** (2.0 mmol) and argon-sparged HPLC grade acetone (0.05 M). The vial was sealed with Parafilm, and placed in the 365 nm irradiation setup. The reaction was stirred for 16 h, then the solvent was evaporated and the crude mixture purified by flash column chromatography on silica gel to furnish the product **3**.

## E.2.2 General procedure B

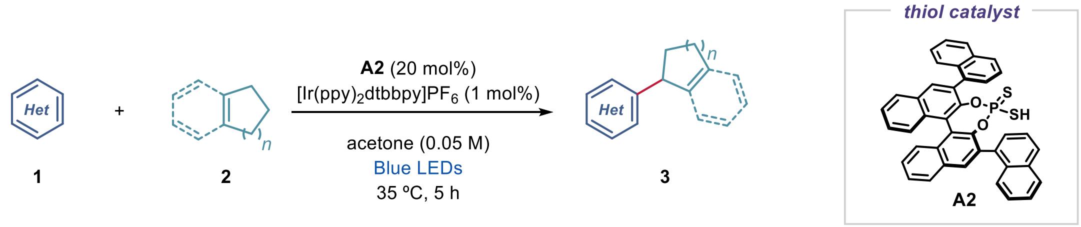


To an argon-purged glass vial, containing the dithiophosphoric acid catalyst **A2** (25.3 mg, 0.04 mmol), [Ir(ppy)$_2$dtbbpy]PF$_6$ (1.8 mg, 2.0 µmol), and pyridine derivative **1** (0.2 mmol), was added the allylic precursor **2** (2.0 mmol) followed by argon-sparged HPLC grade acetone (0.05 M). The vial was sealed with Parafilm, and placed in the 455 nm irradiation setup. The reaction was stirred for 5 h, then the solvent was evaporated and the crude mixture purified by flash column chromatography on silica gel to furnish the product **3**.

## E.2.3 Characterization of Products 3

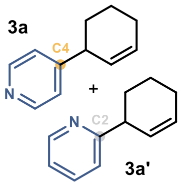


### 4-(cyclohex-2-en-1-yl)pyridine (3a) + 2-(cyclohex-2-en-1-yl)pyridine (3a’)

Prepared according to General Procedure A using pyridine **1a** (16.2 µL, 0.2 mmol) and cyclohexene **2a** (203 µL, 2.0 mmol). The regioisomeric ratio **3a/3a’** (6:1) of the crude mixture was measured by $^1$H NMR analysis. Purification by column chromatography (SiO$_2$, 1:10:89 Et$_3$N/EtOAc/hexanes) afforded product **3a** as a colorless oil (17.6 mg, 55% yield, > 20:1 *r.r.*). The minor regioisomer **3a’** was not isolated after column chromatography. **3a** displayed spectroscopic data consistent with those reported previously.[14]

$^1$H NMR (400 MHz, CDCl$_3$) δ 8.53 – 8.49 (m, 2H), 7.17 – 7.14 (m, 2H), 5.97 (m, 1H), 5.70 – 5.65 (m, 1H), 3.40 (m, 1H), 2.16 – 2.09 (m, 2H), 2.08 – 1.98 (m, 1H), 1.80 – 1.71 (m, 1H), 1.69 – 1.61 (m, 1H), 1.61 – 1.51 (m, 1H).
$^{13}$C NMR (126 MHz, CDCl$_3$) δ 155.3, 149.7, 129.7, 128.2, 123.1, 41.1, 31.7, 24.9, 20.8.

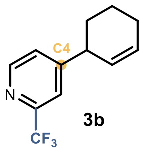


### 4-(cyclohex-2-en-1-yl)-2-(trifluoromethyl)pyridine (3b)

Prepared according to General Procedure A using 2-(trifluoromethyl)pyridine (23.0 µL, 0.2 mmol) and cyclohexene **2a** (203 µL, 2.0 mmol). No other regioisomer was detected in the crude mixture. Purification by column chromatography (SiO$_2$, 1:5:96 Et$_3$N/EtOAc/hexanes) afforded product **3b** as a colorless oil (29.3 mg, 65% yield, > 20:1 *r.r.*).

$^1$H NMR (500 MHz, CDCl$_3$) δ 8.64 (d, *J* = 5.0 Hz, 1H), 7.56 (d, *J* = 1.6 Hz, 1H), 7.36 (dd, *J* = 5.0, 1.6 Hz, 1H), 6.04 (m, 1H), 5.68 (m, 1H), 3.54 – 3.47 (m, 1H), 2.18 – 2.11 (m, 2H), 2.11 – 2.05 (m, 1H), 1.77 – 1.71 (m, 1H), 1.71 – 1.64 (m, 2H), 1.61 – 1.52 (m, 1H).
$^{13}$C NMR (126 MHz, CDCl$_3$) δ 157.5, 149.9, δ 148.3 (q, *J* = 34.0 Hz), 130.6, 127.1, 125.8, 119.8 (q, *J* = 2.9 Hz), 41.2, 31.8, 29.7, 24.8, 20.7.
$^{19}$F NMR (471 MHz, CDCl$_3$) δ -67.9.
HRMS (ESI$^+$) Calculated for $C_{12}H_{13}F_3N$ [M+H]$^+$: 228.0995 found: 228.0988.

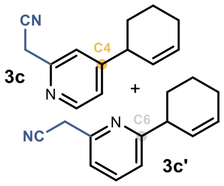


**2-(4-(cyclohex-2-en-1-yl)pyridin-2-yl)acetonitrile (3c) + 2-(6-(cyclohex-2-en-1-yl)pyridin-2-yl)acetonitrile (3c')**

Prepared according to General Procedure A using 2-pyridylacetonitrile (23.6 mg, 0.2 mmol) and cyclohexene **2a** (203 µL, 2.0 mmol). The regioisomeric ratio **3c/3c'** (8:1) of the crude mixture was measured by $^{1}$H NMR analysis. Purification by column chromatography ($SiO_2$, 1:50:49 $Et_3N$/EtOAc/hexanes) afforded product **3c** as a colorless oil (17.4 mg, 44% yield, > 20:1 *r.r.*). The minor regioisomer **3c'** was not isolated after column chromatography.

$^{1}$H NMR (500 MHz, $CDCl_3$) δ 8.48 (d, $J$ = 5.1 Hz, 1H), 7.30 (s, 1H), 7.14 (dd, $J$ = 5.1, 1.6 Hz, 1H), 6.06 – 5.97 (m, 1H), 5.71 – 5.62 (m, 1H), 3.94 (s, 2H), 3.49 – 3.39 (m, 1H), 2.17 – 2.09 (m, 2H), 2.08 – 2.00 (m, 1H), 1.80 – 1.70 (m, 1H), 1.70 – 1.61 (m, 1H), 1.60 – 1.50 (m, 1H).
$^{13}$C NMR (126 MHz, $CDCl_3$) δ 157.3, 150.4, 149.8, 130.2, 127.6, 122.5, 121.6, 117.2, 41.2, 31.7, 26.6, 24.8, 20.8.
HRMS (ESI$^+$) Calculated for $C_{13}H_{15}N_2$ $[M+H]^+$: 199.1230 found: 199.1229.

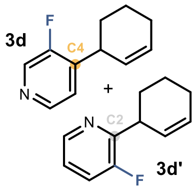


**4-(cyclohex-2-en-1-yl)-3-fluoropyridine (3d) + 2-(cyclohex-2-en-1-yl)-3-fluoropyridine (3d')**

Prepared according to General Procedure A using 3-fluoropyridine (17.2 µL, 0.2 mmol) and cyclohexene **2a** (203 µL, 2.0 mmol). The regioisomeric ratio **3d/3d'** (5:1) of the crude mixture was measured by $^{1}$H NMR analysis. Purification by column chromatography ($SiO_2$, 1:4:95 $Et_3N$/EtOAc/hexanes) afforded a mixture of products **3d** and **3d'** as a colorless oil (16.5 mg, 47% yield, 6:1 *r.r.*). An analytically pure sample of **3d** was obtained by preparative TLC ($SiO_2$, 1:4:95 $Et_3N$/EtOAc/hexanes, >20:1 *r.r.*). Only the major product **3d** is described.

$^{1}$H NMR (400 MHz, $CDCl_3$) δ 8.39 (s, 1H), 8.35 (d, $J$ = 4.9 Hz, 1H), 7.21 (dd, $J$ = 6.5, 4.9 Hz, 1H), 6.06 – 5.98 (m, 1H), 5.68 – 5.57 (m, 1H), 3.85 – 3.74 (m, 1H), 2.18 – 2.10 (m, 2H), 2.09 – 2.03 (m, 1H), 1.76 – 1.66 (m, 2H), 1.63 – 1.53 (m, 1H).
$^{13}$C NMR (126 MHz, $CDCl_3$) δ 145.7 (d, $J$ = 5.1 Hz), 141.7 (d, $J$ = 12.4 Hz), 137.7, 137.5, 130.45, 126.9, 123.8, 33.8, 29.8, 24.8, 20.6.
$^{19}$F{$^{1}$H} NMR (471 MHz, $CDCl_3$) δ -59.03, -134.28 (d, $J$ = 6.4 Hz).
HRMS (APCI$^+$) Calculated for $C_{11}H_{13}FN$ $[M+H]^+$: 178.1027 found: 178.1029.

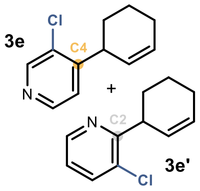


**3-chloro-4-(cyclohex-2-en-1-yl)pyridine (3e) + 3-chloro-2-(cyclohex-2-en-1-yl)pyridine (3e')**

Prepared according to General Procedure A using 3-chloro pyridine (22.7 mg, 0.2 mmol) and cyclohexene **2a** (203 µL, 2.0 mmol). Purification by column chromatography ($SiO_2$, 1:5:94 $Et_3N$/EtOAc/hexanes) afforded products **3e** + **3e'** (14.9 mg, 39% yield, 8:1 *r.r.*) as a colorless oil. Only the major product **3e** is described.

$^{1}$H NMR (400 MHz, $CDCl_3$) δ 8.54 (s, 1H), 8.41 (d, $J$ = 5.0 Hz, 1H), 7.21 (d, $J$ = 5.0 Hz, 1H), 6.09 – 6.02 (m, 1H), 5.67 – 5.58 (m, 1H), 3.91 – 3.83 (m, 1H), 2.17 – 2.06 (m, 3H), 1.73 – 1.65 (m, 2H), 1.58 – 1.44 (m, 1H).
$^{13}$C NMR (101 MHz, $CDCl_3$) δ 152.1, 149.3, 147.7, 131.7, 130.6, 127.2, 123.8, 37.6, 29.1, 24.9, 20.5.
HRMS (ESI$^+$) Calculated for $C_{11}H_{13}ClN$ $[M+H]^+$: 194.0731 found: 194.0732.

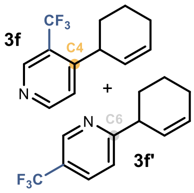


**4-(cyclohex-2-en-1-yl)-3-(trifluoromethyl)pyridine (3f) + 6-(cyclohex-2-en-1-yl)-3-(trifluoromethyl)pyridine (3f')**

Prepared according to General Procedure A using 3-(trifluoromethyl)pyridine (23.0 µL, 0.2 mmol) and cyclohexene **2a** (203 µL, 2.0 mmol). The regioisomeric ratio **3f/3f'** (9:1) of the crude mixture was measured by $^1$H NMR analysis. Purification by column chromatography ($SiO_2$, 1:4:95 $Et_3N$/EtOAc/hexanes) afforded product **3f** as a colorless oil (20.1 mg, 44% yield, > 20:1 *r.r.*). The minor regioisomer **3f'** was not isolated after column chromatography.

$^1$H NMR (500 MHz, $CDCl_3$) δ 8.84 (s, 1H), 8.70 (d, $J$ = 5.2 Hz, 1H), 7.38 (d, $J$ = 5.2 Hz, 1H), 6.08 – 5.95 (m, 1H), 5.62 – 5.51 (m, 1H), 3.87 – 3.80 (m, 1H), 2.20 – 2.13 (m, 2H), 2.13 – 2.08 (m, 1H), 1.88 – 1.77 (m, 1H), 1.77 – 1.65 (m, 1H), 1.51 – 1.43 (m, 1H).
$^{13}$C NMR (126 MHz, $CDCl_3$) δ 155.0, 152.9, 146.8 (q, $J$ = 6.5 Hz), 130.0, 127.9, 124.0, 37.6 (d, $J$ = 1.8 Hz), 31.9, 24.7, 21.2.
$^{19}$F{$^1$H} NMR (376 MHz, $CDCl_3$) δ -59.1, -59.1.
HRMS (ESI$^+$) Calculated for $C_{12}H_{13}F_3N$ [M+H]$^+$: 228.0995 found: 228.0987.

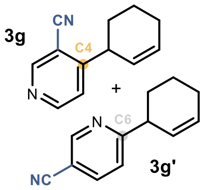


**4-(cyclohex-2-en-1-yl)nicotinonitrile (3g) + 6-(cyclohex-2-en-1-yl)nicotinonitrile (3g')**

Prepared according to General Procedure A using nicotinonitrile (20.8 mg, 0.2 mmol) and cyclohexene **2a** (203 µL, 2.0 mmol). The regioisomeric ratio **3g/3g'** (14:1) of the crude mixture was measured by $^1$H NMR analysis. Purification by column chromatography ($SiO_2$, 1:4:95 $Et_3N$/EtOAc/hexanes) afforded product **3g** as a colorless oil (18.0 mg, 50% yield, > 20:1 *r.r.*). The minor regioisomer **3g'** was not isolated after column chromatography.

$^1$H NMR (400 MHz, $CDCl_3$) δ 8.81 (s, 1H), 8.69 (d, $J$ = 5.3 Hz, 1H), 7.34 (d, $J$ = 5.2 Hz, 1H), 6.10 – 6.05 (m, 1H), 5.63 – 5.58 (m, 1H), 3.86 – 3.80 (m, 1H), 2.20 – 2.11 (m, 3H), 1.76 – 1.67 (m, 2H), 1.58 – 1.49 (m, 1H).
$^{13}$C NMR (101 MHz, $CDCl_3$) δ 159.0, 153.2, 152.9, 131.6, 126.1, 123.0, 116.0, 110.1, 39.9, 30.9, 24.8, 20.7.
HRMS (APCI$^+$) Calculated for $C_{12}H_{13}N_2$ [M+H]$^+$: 185.1073 found: 185.1075.

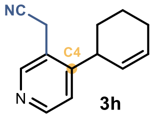


**2-(4-(cyclohex-2-en-1-yl)pyridin-3-yl)acetonitrile (3h)**

Prepared according to General Procedure A using 3-pyridylacetonitrile (21.3 µL, 0.2 mmol) and cyclohexene **2a** (203 µL, 2.0 mmol) prolonging the reaction time to 48 hours. No other regioisomer was detected in the crude mixture. Purification by column chromatography ($SiO_2$, 1:9:90 $Et_3N$/EtOAc/hexanes) afforded product **3h** as a colorless oil (13.0 mg, 33% yield, > 20:1 *r.r.*).

$^1$H NMR (400 MHz, $CDCl_3$) δ 8.50 (d, $J$ = 2.4 Hz, 1H), 7.64 (dd, $J$ = 8.1, 2.4 Hz, 1H), 7.25 (d, $J$ = 8.1 Hz, 1H), 6.00 – 5.93 (m, 1H), 5.81 – 5.75 (m, 1H), 3.74 (s, 2H), 3.64 – 3.57 (m, 1H), 2.17 – 2.04 (m, 3H), 1.79 – 1.63 (m, 3H).
$^{13}$C NMR (101 MHz, $CDCl_3$) δ 153.6, 150.2, 150.1, 130.7, 127.5, 124.2, 123.5, 117.2, 37.8, 30.5, 24.8, 21.0, 18.8.
HRMS (ESI$^+$) Calculated for $C_{13}H_{15}N_2$ [M+H]$^+$: 199.1230 found: 199.1233.

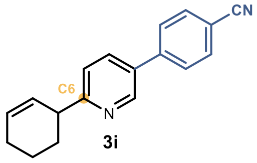

3i

**4-(6-(cyclohex-2-en-1-yl)pyridin-3-yl)benzonitrile (3i)**

Prepared according to General Procedure A using 4-(pyridin-3-yl)benzonitrile **1i** (36.0 mg, 0.2 mmol) and cyclohexene **2a** (203 µL, 2.0 mmol). ). No other regioisomer was detected in the crude mixture. Purification by column chromatography ($SiO_2$, 1:10:89 $Et_3N$/EtOAc/hexanes) afforded product **3i** as a white solid (16.1 mg, 31% yield, > 20:1 *r.r.*).

$^{1}$H NMR (400 MHz, $CDCl_3$) δ 8.81 (dd, $J$ = 2.5, 0.9 Hz, 1H), 7.84 (dd, $J$ = 8.1, 2.5 Hz, 1H), 7.80 – 7.76 (m, 2H), 7.72 – 7.68 (m, 2H), 7.35 (dd, $J$ = 8.1, 0.8 Hz, 1H), 6.04 – 5.97 (m, 1H), 5.89 – 5.81 (m, 1H), 3.72 – 3.64 (m, 1H), 2.20 – 2.09 (m, 3H), 1.88 – 1.63 (m, 3H).

$^{13}$C NMR (101 MHz, $CDCl_3$) δ 165.9, 147.7, 142.5, 134.9, 132.8, 132.3, 129.4, 128.2, 127.6, 122.0, 118.7, 111.6, 43.8, 30.7, 25.0, 21.1.

HRMS (ESI$^+$) Calculated for $C_{18}H_{17}N_2$ $[M+H]^+$: 261.1386 found: 261.1398.

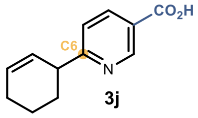

3j

**6-(cyclohex-2-en-1-yl)nicotinic acid (3j)**

Prepared according to General Procedure A using nicotinic acid (24.6 mg, 0.2 mmol) and cyclohexene **2a** (203 µL, 2.0 mmol). No other regioisomer was detected in the crude mixture. The yield (65%, single regioisomer) of **3j** was inferred by $^{1}$H NMR analysis of the crude reaction mixture using trichloroethylene as the internal standard. A 92% pure sample of **3j** (estimated by $^{1}$H NMR integration) was obtained after purification by column chromatography ($SiO_2$, 1:4:95 AcOH/MeOH/$CH_2Cl_2$ as an off-white solid.

$^{1}$H NMR (400 MHz, MeOD) δ 9.10 (d, $J$ = 1.4 Hz, 1H), 8.40 (dd, $J$ = 8.2, 2.2 Hz, 1H), 7.51 (d, $J$ = 8.2 Hz, 1H), 6.06 (dtd, $J$ = 9.9, 3.7, 2.3 Hz, 1H), 5.88 – 5.79 (m, 1H), 3.73 (tq, $J$ = 5.6, 2.8 Hz, 1H), 2.30 – 2.11 (m, 3H), 1.92 – 1.70 (m, 3H).

$^{13}$C NMR (101 MHz, MeOD) δ 169.3, 166.6, 149.6, 138.3, 129.4, 127.1, 125.0, 121.8, 43.7, 30.2, 24.5, 20.7.

HRMS (ESI$^+$) Calculated for $C_{12}H_{14}NO_2$ $[M+H]^+$: 204.1019 found: 204.1028.

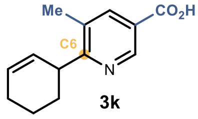

3k

**6-(cyclohex-2-en-1-yl)-5-methylnicotinic acid (3k)**

Prepared according to General Procedure A using 5-methylnicotinic acid (27.4 mg, 0.2 mmol) and cyclohexene **2a** (203 µL, 2.0 mmol). No other regioisomer was detected in the crude mixture. Purification by column chromatography ($SiO_2$, 1:49:50 AcOH/EtOAc/hexanes) afforded product **3k** as a white solid (21.0 mg, 48% yield, > 20:1 *r.r.*).

$^{1}$H NMR (400 MHz, MeOD) δ 8.86 (d, $J$ = 2.1 Hz, 1H), 8.14 (dd, $J$ = 2.1, 0.9 Hz, 1H), 5.97 – 5.90 (m, 1H), 5.70 – 5.63 (m, 1H), 3.95 – 3.88 (m, 1H), 2.45 (s, 3H), 2.17 (ddd, $J$ = 12.7, 6.1, 3.0 Hz, 2H), 2.07 – 1.99 (m, 1H), 1.90 (ddd, $J$ = 11.9, 5.4, 2.9 Hz, 1H), 1.80 – 1.62 (m, 2H).

$^{13}$C NMR (101 MHz, MeOD) δ 168.4, 148.4, 140.9, 133.0, 129.7, 129.1, 41.8, 30.0, 25.8, 22.9, 18.6.

HRMS (ESI$^-$) Calculated for $C_{13}H_{14}NO_2$ $[M-H]^+$: 216.1030 found: 216.1027.

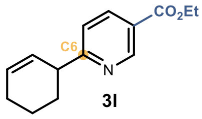

3l

**ethyl 6-(cyclohex-2-en-1-yl)nicotinate (3l)**

Prepared according to General Procedure A using ethyl nicotinate (27.3 µL, 0.2 mmol) and cyclohexene **2a** (203 µL, 2.0 mmol). No other regioisomer was detected in the crude mixture. Purification by column chromatography ($SiO_2$, 1:5:94 $Et_3N$/EtOAc/hexanes) afforded product **3l** as a yellowish oil (30.4 mg, 66% yield, > 20:1 *r.r.*).

$^{1}$H NMR (400 MHz, $CDCl_3$) δ 9.13 (dd, $J$ = 2.2, 0.9 Hz, 1H), 8.19 (dd, $J$ = 8.2, 2.2 Hz, 1H), 7.26 (dd, $J$ = 8.2, 0.9 Hz, 1H), 5.95 (dtd, $J$ = 9.8, 3.7, 2.2 Hz, 1H), 5.80 – 5.75 (m, 1H), 4.38 (q, $J$ = 7.1 Hz, 2H), 3.67 – 3.61 (m, 1H), 2.17 – 2.02 (m, 3H), 1.79 – 1.60 (m, 3H), 1.38 (t, $J$ = 7.1 Hz, 3H).
$^{13}$C NMR (101 MHz, $CDCl_3$) δ 169.9, 165.4, 150.6, 137.4, 129.5, 127.8, 123.9, 121.3, 61.1, 44.1, 30.5, 24.8, 21.0, 14.2.
HRMS: (ESI$^+$) calculated for $C_{14}H_{18}NO_2$ [M+H$^+$]: 232.1332, found 235.1326.

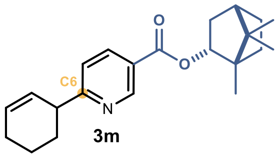

3m

**1,7,7-trimethylbicyclo[2.2.1]heptan-2-yl 6-(cyclohex-2-en-1-yl)nicotinate (3m)**

Prepared according to General Procedure A using 1,7,7-trimethylbicyclo[2.2.1]heptan-2-yl nicotinate **1m** (52.0 mg, 0.2 mmol) and cyclohexene **2a** (203 µL, 2.0 mmol). No other regioisomer was detected in the crude mixture. Purification by column chromatography ($SiO_2$, 2:98 EtOAc/hexanes) afforded product **3m** as a colorless oil (49.0 mg, 72% yield, > 20:1 *r.r.*).

$^{1}$H NMR (500 MHz, $CDCl_3$) δ 9.19 (dd, $J$ = 2.2, 0.8 Hz, 1H), 8.22 (dd, $J$ = 8.2, 2.2 Hz, 1H), 7.29 (dd, $J$ = 8.2, 0.9 Hz, 1H), 5.99 – 5.95 (m, 1H), 5.80 – 5.77 (m, 1H), 5.13 – 5.10 (m, 1H), 3.68 – 3.65 (m, 1H), 2.47 (dddd, $J$ = 13.5, 9.9, 4.7, 3.3 Hz, 1H), 2.15 – 2.04 (m, 4H), 1.84 – 1.63 (m, 5H), 1.44 – 1.36 (m, 1H), 1.32 – 1.24 (m, 1H), 1.10 (ddd, $J$ = 13.8, 3.5, 1.3 Hz, 1H), 0.96 (s, 3H), 0.90 (d, $J$ = 4.8 Hz, 6H).
$^{13}$C NMR (126 MHz, $CDCl_3$) δ 170.0, 165.8, 150.8, 137.6, 129.6, 128.0, 124.5, 121.6, 81.0, 49.3, 48.0, 45.1, 44.3, 37.0, 30.7, 28.2, 27.5, 25.0, 21.2, 19.8, 19.0, 13.7.
HRMS: (ESI$^+$) calculated for $C_{22}H_{30}NO_2$ [M+H$^+$]: 340.2271, found 340.2274.

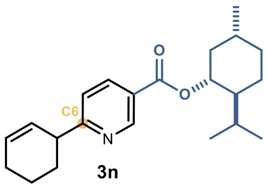

3n

**(1R,2S,5R)-2-isopropyl-5-methylcyclohexyl 6-(cyclohex-2-en-1-yl)nicotinate (3n)**

Prepared according to General Procedure A using (1R,2S,5R)-2-isopropyl-5-methylcyclohexyl nicotinate **1n** (52.3 mg, 0.2 mmol) and cyclohexene **2a** (203 µL, 2.0 mmol). No other regioisomer was detected in the crude mixture. Purification by column chromatography ($SiO_2$, 1:99 EtOAc/hexanes) afforded product **3n** as a colorless oil (34.0 mg, 50% yield, > 20:1 *r.r.*).

$^{1}$H NMR (400 MHz, $CDCl_3$) δ 9.14 (dd, $J$ = 2.2, 0.9 Hz, 1H), 8.20 (dd, $J$ = 8.2, 2.2 Hz, 1H), 7.28 (d, $J$ = 0.8 Hz, 1H), 5.99 – 5.94 (m, 1H), 5.80 – 5.75 (m, 1H), 4.93 (td, $J$ = 10.9, 4.4 Hz, 1H), 3.68 – 3.62 (m, 1H), 2.14 – 2.06 (m, 4H), 1.96 – 1.88 (m, 1H), 1.80 – 1.65 (m, 6H), 1.58 – 1.51 (m, 2H), 1.17 – 1.05 (m, 2H), 0.91 (dd, $J$ = 8.7, 6.8 Hz, 6H), 0.78 (d, $J$ = 6.9 Hz, 3H).
$^{13}$C NMR (101 MHz, $CDCl_3$) δ 170.0, 165.1, 150.7, 137.6 (d1,d2), 129.6 (d1,d2), 128.0, 124.4, 121.6 (d1,d2), 75.4, 47.4, 44.3, 41.1, 34.4, 31.6, 30.7 (d1,d2), 26.7 (d1,d2), 25.0, 23.8(d1,d2), 22.1, 21.2(d1,d2), 20.9, 16.7(d1,d2).
HRMS: (ESI$^+$) calculated for $C_{22}H_{32}NO_2$ [M+H$^+$]: 342.2428, found 342.2439.

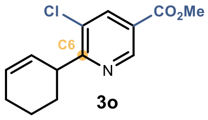

3o

**Methyl 5-chloro-6-(cyclohex-2-en-1-yl)nicotinate (3o)**

Prepared according to General Procedure A using methyl 5-chloronicotinate (34.3 mg, 0.2 mmol) and cyclohexene **2a** (203 µL, 2.0 mmol). No other regioisomer was detected in the crude mixture. Purification by column chromatography ($SiO_2$,

1:2:97 $Et_3N$/EtOAc/hexanes) afforded product **3o** as a colorless oil (23.0 mg, 46% yield, > 20:1 *r.r.*).

$^1$H NMR (400 MHz, $CDCl_3$) δ 9.05 (d, *J* = 1.9 Hz, 1H), 8.23 (d, *J* = 1.9 Hz, 1H), 6.03 – 5.96 (m, 1H), 5.77 – 5.70 (m, 1H), 4.17 – 4.09 (m, 1H), 3.94 (s, 3H), 2.24 – 2.05 (m, 3H), 1.93 – 1.85 (m, 1H), 1.77 – 1.64 (m, 2H).
$^{13}$C NMR (101 MHz, $CDCl_3$) δ 166.5, 165.0, 148.6, 137.9, 130.9, 129.2, 127.5, 125.1, 52.7, 40.6, 28.6, 24.8, 21.8.
HRMS (ESI$^+$) Calculated for C13H15ClNO2 [M+H]$^+$: 252.0786 found: 252.0785.

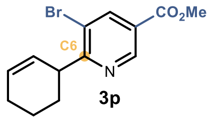

**3p**

**Methyl 5-bromo-6-(cyclohex-2-en-1-yl)nicotinate (3p)**

Prepared according to General Procedure A using methyl 5-bromonicotinate (43.2 mg, 0.2 mmol) and cyclohexene **2a** (203 µL, 2.0 mmol) prolonging the reaction time to 24 hours. No other regioisomer was detected in the crude mixture. Purification by column chromatography ($SiO_2$, 1:2:97 $Et_3N$/EtOAc/hexanes) afforded product **3p** as a colorless oil (18.5 mg, 31% yield, > 20:1 *r.r.*).

$^1$H NMR (400 MHz, $CDCl_3$) δ 9.09 (d, *J* = 1.9 Hz, 1H), 8.41 (d, *J* = 1.9 Hz, 1H), 6.03 – 5.95 (m, 1H), 5.77 – 5.71 (m, 1H), 4.18 – 4.10 (m, 1H), 3.94 (s, 3H), 2.24 – 2.09 (m, 3H), 1.93 – 1.87 (m, 1H), 1.75 – 1.60 (m, 2H).
$^{13}$C NMR (101 MHz, $CDCl_3$) δ 167.6, 164.8, 149.2, 141.3, 129.1, 127.7, 125.2, 120.9, 52., 42.7, 28.8, 24.8, 21.8.
HRMS (ESI$^+$) Calculated for C13H15BrNO2 [M+H]$^+$: 296.0281 found: 296.0283.

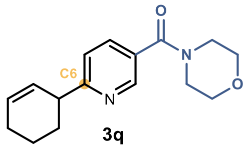

**3q**

**(6-(cyclohex-2-en-1-yl)pyridin-3-yl)(morpholino)methanone (3q)**

Prepared according to General Procedure A using morpholino(pyridin-3-yl)methanone **1q** (38.4 mg, 0.2 mmol) and cyclohexene **2a** (203 µL, 2.0 mmol). No other regioisomer was detected in the crude mixture. Purification by column chromatography ($SiO_2$, 1:99 $Et_3N$/EtOAc) afforded product **3q** as a colorless oil (21.6 mg, 40% yield, > 20:1 *r.r.*).

$^1$H NMR (500 MHz, $CDCl_3$) δ 8.62 (d, *J* = 2.4 Hz, 1H), 7.71 (dd, *J* = 8.0, 2.3 Hz, 1H), 7.29 (d, *J* = 8.0, 1H), 6.03 – 5.94 (m, 1H), 5.82 – 5.76 (m, 1H), 3.95 – 3.40 (m, 9H), 2.17 – 2.05 (m, 3H), 1.82 – 1.74 (m, 1H), 1.73 – 1.64 (m, 2H).
$^{13}$C NMR (126 MHz, $CDCl_3$) δ 168.2, 167.3, 147.6, 135.7, 129.5, 128.6, 128.0, 121.8, 66.8, 44.0, 30.6, 24.9, 21.0.
HRMS (ESI$^+$) Calculated for $C_{16}H_{21}N_2O_2$ [M+H]$^+$: 273.1598 found: 273.1595.

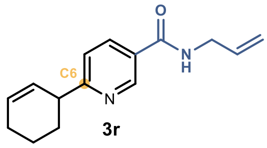

**3r**

**N-allyl-6-(cyclohex-2-en-1-yl)nicotinamide (3r)**

Prepared according to General Procedure A using *N*-allylnicotinamide **1r** (32.4 mg, 0.2 mmol) and cyclohexene **2a** (203 µL, 2.0 mmol). No other regioisomer was detected in the crude mixture. Purification by column chromatography ($SiO_2$, 1:9:90 $Et_3N$/EtOAc/hexanes to 1:24:75 $Et_3N$/EtOAc/hexanes) afforded product **3r** as a colorless oil (16.0 mg, 33% yield, > 20:1 *r.r.*).

$^1$H NMR (400 MHz, $CDCl_3$) δ 8.90 (dd, *J* = 2.4, 0.9 Hz, 1H), 8.05 (dd, *J* = 8.1, 2.4 Hz, 1H), 7.28 (dd, *J* = 8.1, 0.9 Hz, 1H), 6.24 (s, 1H), 5.99 – 5.88 (m, 2H), 5.80 – 5.74 (m, 1H), 5.27 (dq, *J* = 17.1, 1.6 Hz, 1H), 5.20 (dq, *J* = 10.2, 1.4 Hz, 1H), 4.10 (tt, *J* = 5.8, 1.6 Hz, 2H), 3.68 – 3.60 (m, 1H), 2.15 – 2.05 (m, 3H), 1.79 – 1.63 (m, 3H).

$^{13}$C NMR (101 MHz, $CDCl_3$) δ 169.0, 165.8, 147.5, 135.7, 134.0, 129.7, 128.1, 127.9, 121.9, 117.1, 44.2, 42.6, 30.7, 25.1, 21.2.
HRMS (ESI$^+$) Calculated for $C_{15}H_{19}N_2O$ [M+H]$^+$: 243.1492 found: 243.1501.

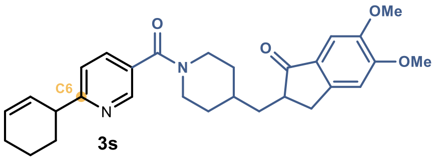

3s

**2-((1-(6-(cyclohex-2-en-1-yl)nicotinoyl)piperidin-4-yl)methyl)-5,6-dimethoxy-2,3-dihydro-1H-inden-1-one (3s)**

Prepared according to General Procedure A using donepezil nicotinate **1s** (78.9 mg, 0.2 mmol) and cyclohexene **2a** (203 µL, 2.0 mmol). No other regioisomer was detected in the crude mixture. Purification by column chromatography ($SiO_2$, 1:1:98 $Et_3N/MeOH/CH_2Cl_2$) afforded product **3s** (48.6 mg, 51% yield, > 20:1 *r.r.*) as a colorless oil.

$^1$H NMR (400 MHz, $CDCl_3$) δ 8.62 (dd, $J$ = 2.3, 0.9 Hz, 1H), 7.71 (dd, $J$ = 8.0, 2.3 Hz, 1H), 7.28 (d, $J$ = 8.1 Hz, 1H), 7.18 (s, 1H), 6.88 (s, 1H), 6.02 – 5.95 (m, 1H), 5.86 – 5.76 (m, 1H), 4.74 (br s, 1H), 3.98 (s, 3H), 3.92 (s, 3H), 3.88 – 3.73 (m, 1H), 3.70 – 3.59 (m, 1H), 3.29 (dd, $J$ = 17.4, 8.0 Hz, 1H), 3.10 (br s, 1H), 2.85 (br s, 1H), 2.77 – 2.68 (m, 2H), 2.17 – 2.06 (m, 3H), 2.02 – 1.82 (m, 3H), 1.82 – 1.64 (m, 2H), 1.48 – 1.37 (m, 2H).
$^{13}$C NMR (101 MHz, $CDCl_3$) δ 207.2, 168.0, 166.8, 155.6, 149.6, 148.6, 147.4, 135.5, 129.5, 129.4, 129.2, 128.1, 121.7, 107.3, 104.4, 56.3, 56.1, 44.0, 30.6, 24.9, 21.1.
HRMS: (ESI$^+$) calculated for $C_{29}H_{34}N_2NaO_4$ [M+Na$^+$]: 497.2411, found 497.2415.

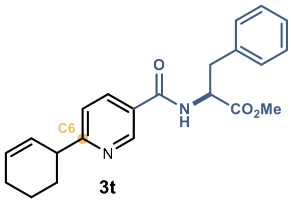

3t

**Methyl (6-(cyclohex-2-en-1-yl)nicotinoyl)-L-phenylalaninate (3t)**

Prepared according to General Procedure A using methyl nicotinoyl-L-phenylalaninate **1t** (26.0 mg, 0.2 mmol) and cyclohexene **2a** (203 µL, 2.0 mmol). No other regioisomer was detected in the crude mixture. Purification by column chromatography ($SiO_2$, 1:9:90 $Et_3N$/EtOAc/hexanes to 1:19:80 $Et_3N$/EtOAc/hexanes) afforded product **3t** as a white solid (30.0 mg, 41% yield, 1:1 *d.r.*, > 20:1 *r.r.*). The diastereomeric ratio was determined by UPC$^2$ analysis on a Daicel Chiralpak ID-3 column (gradient: 1 min 100% $CO_2$; 5 min from 100% $CO_2$ to 60% $CO_2$ - 40% MeOH; flow rate 2.0 mL/min; λ = 268 nm: $\tau_1$ = 5.6 min, $\tau_2$ = 5.9 min).

$^1$H NMR (400 MHz, $CDCl_3$) δ 8.83 (dt, $J$ = 2.2, 1.0 Hz, 1H), 7.95 (dd, $J$ = 8.2, 2.4 Hz, 1H), 7.31 – 7.22 (m, 4H), 7.13 – 7.11 (m, 2H), 6.57 (d, $J$ = 7.6 Hz, 1H), 5.99 – 5.94 (m, 1H), 5.79 – 5.74 (m, 1H), 5.07 (dt, $J$ = 7.6, 5.6 Hz, 1H), 3.77 (s, 3H), 3.65 – 3.60 (m, 1H), 3.24 (qd, $J$ = 13.9, 5.6 Hz, 2H), 2.13 – 2.04 (m, 3H), 1.77 – 1.63 (m, 3H).
$^{13}$C NMR (101 MHz, $CDCl_3$) δ 172.0, 169.2, 165.4, 147.8, 147.8, 135.7, 135.6, 129.6, 129.4, 128.9, 128.0, 127.5, 127.3, 121.8, 53.6, 52.6, 44.2, 37.9, 30.7, 25.0, 21.1.
HRMS: (ESI$^+$) calculated for $C_{22}H_{25}N_2O_3$ [M+H$^+$]: 365.1860, found 365.1864.

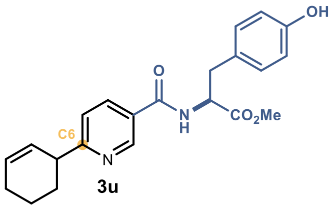

3u

**Methyl (6-(cyclohex-2-en-1-yl)nicotinoyl)-L-tyrosinate (3u)**

Prepared according to General Procedure A using methyl nicotinoyl-L-tyrosinate **1u** (60.0 mg, 0.2 mmol) and cyclohexene **2a** (203 µL, 2.0 mmol). No other regioisomer was detected in the crude mixture. Purification by column chromatography ($SiO_2$, 1:29:70 $Et_3N$/acetone/hexanes) afforded product **3u** as a white

solid (31.8 mg, 42% yield, 1:1 d.r., > 20:1 *r.r.*). The diastereomeric ratio was determined by UPC$^2$ analysis on a Daicel Chiralpak ID-3 column (gradient: 1 min 100% $CO_2$; 5 min from 100% $CO_2$ to 60% $CO_2$ - 40% MeOH; flow rate 2.0 mL/min; $\lambda$ = 268 nm: $\tau_1$ = 10.8 min, $\tau_2$ = 11.3 min).

$^1$H NMR (400 MHz, $CDCl_3$) δ 8.80 (d, *J* = 1.5 Hz, 1H), 8.03 (ddd, *J* = 8.1, 2.4, 1.5 Hz, 1H), 7.29 (d, *J* = 8.1 Hz, 1H), 6.98 – 6.94 (m, 2H), 6.76 – 6.71 (m, 2H), 6.65 (d, *J* = 7.8 Hz, 1H), 5.98 – 5.93 (m, 1H), 5.78 – 5.70 (m, 1H), 5.04 (dt, *J* = 7.7, 5.6 Hz, 1H), 3.78 (s, 3H), 3.69 – 3.60 (m, 1H), 3.16 (qd, *J* = 14.1, 5.7 Hz, 2H), 2.13 – 2.04 (m, 3H), 1.77 – 1.62 (m, 3H).
$^{13}$C NMR (101 MHz, $CDCl_3$) δ 172.2, 169.2, 165.4, 156.0, 147.3, 136.2, 130.5, 129.9, 127.7, 127.6, 126.9, 122.1, 115.9, 53.8, 52.7, 44.0, 37.1, 30.7, 25.0, 21.1.
HRMS: (ESI$^+$) calculated for $C_{22}H_{24}N_2NaO_4$ [M+Na$^+$]: 403.1628, found 403.1627.

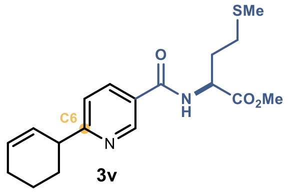

3v

**Methyl (6-(cyclohex-2-en-1-yl)nicotinoyl)-L-methioninate (3v)**

Prepared according to General Procedure A using methyl nicotinoyl-L-methioninate **1v** (53.7 mg, 0.2 mmol) and cyclohexene **2a** (203 µL, 2.0 mmol). No other regioisomer was detected in the crude mixture. Purification by column chromatography ($SiO_2$, 1:9:90 $Et_3N$/EtOAc/hexanes to 1:23:76 $Et_3N$/EtOAc/hexanes) afforded product **3v** as a white solid (31.0 mg, 44% yield, 1:1 *d.r.*, > 20:1 *r.r.*). The diastereomeric ratio was determined by UPC$^2$ analysis on a Daicel Chiralpak ID-3 column (gradient: 1 min 100% $CO_2$; 5 min from 100% $CO_2$ to 60% $CO_2$ - 40% EtOH; flow rate 2.0 mL/min; $\lambda$ = 268 nm: $\tau_1$ = 11.7 min, $\tau_2$ = 12.0 min).

$^1$H NMR (400 MHz, $CDCl_3$) δ 8.96 (d, *J* = 2.4 Hz, 1H), 8.04 (dd, *J* = 8.1, 2.4 Hz, 1H), 7.27 (d, *J* = 8.2 Hz, 1H), 7.14 (d, *J* = 7.6 Hz, 1H), 5.98 – 5.93 (m, 1H), 5.78 – 5.74 (m, 1H), 4.91 (td, *J* = 7.3, 5.0 Hz, 1H), 3.78 (s, 3H), 3.65 – 3.60 (m, 1H), 2.58 (t, *J* = 7.2 Hz, 2H), 2.31 – 2.22 (m, 1H), 2.17-2.04 (m, 4H), 2.10 (s, 3H), 1.77 – 1.63 (m, 3H).
$^{13}$C NMR (101 MHz, $CDCl_3$) δ 172.5, 169.2, 165.6, 148.0, 135.7, 129.6, 128.0, 127.1, 121.8, 52.8, 52.3, 44.1, 31.3, 30.7, 30.3, 25.0, 21.1, 15.7.
HRMS: (ESI$^+$) calculated for $C_{18}H_{23}N_2O_3S$ [M+H$^+$]: 347.1435, found 347.1439.

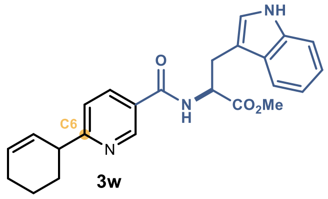

3w

**Methyl (6-(cyclohex-2-en-1-yl)nicotinoyl)-L-tryptophanate (3w)**

Prepared according to General Procedure A using methyl nicotinoyl-L-tryptophanate **1w** (64.7 mg, 0.2 mmol) and cyclohexene **2a** (203 µL, 2.0 mmol). No other regioisomer was detected in the crude mixture. Purification by column chromatography ($SiO_2$, 1:19:80 $Et_3N$/EtOAc/hexanes to 1:59:40 $Et_3N$/EtOAc/hexanes) afforded product **3w** as a white solid (32.5 mg, 40% yield, 1:1 *d.r.*, > 20:1 *r.r.*). The diastereomeric ratio was determined by UPC$^2$ analysis on a Daicel Chiralpak IA-3 column (gradient: 1 min 100% $CO_2$; 5 min from 100% $CO_2$ to 60% $CO_2$ - 40% EtOH; flow rate 2.0 mL/min; $\lambda$ = 268 nm: $\tau_1$ = 6.9 min, $\tau_2$ = 7.2 min).

$^1$H NMR (400 MHz, $CDCl_3$) δ 8.81 (ddd, *J* = 3.3, 2.3, 0.8 Hz, 1H), 8.40 (br s, 1H), 7.90 (dt, *J* = 8.2, 1.9 Hz, 1H), 7.53 (d, *J* = 7.9 Hz, 1H), 7.33 (d, *J* = 8.1 Hz, 1H), 7.20 (d, *J* = 8.2 Hz, 1H), 7.19 – 7.15 (m, 1H), 7.08 (ddd, *J* = 8.0, 7.0, 1.0 Hz, 1H), 6.99 (d, *J* = 2.4 Hz, 1H), 6.69 (d, *J* = 7.7 Hz, 1H), 5.98 – 5.93 (m, 1H), 5.78 – 5.73 (m, 1H), 5.12 (dt, *J* = 7.6, 5.3 Hz, 1H), 3.72 (s, 3H), 3.64 – 3.59 (m, 1H), 3.45 – 3.43 (m, 2H), 2.12 – 2.03 (m, 3H), 1.77 – 1.61 (m, 3H).

$^{13}C$ NMR (101 MHz, $CDCl_3$) δ 172.4, 169.1, 165.5, 147.9, 147.9, 136.3, 135.7, 135.7, 129.7, 129.6, 128.0, 127.7, 127.3, 123.0, 122.5, 121.7, 119.9, 118.6, 111.5, 109.9, 53.5, 52.7, 44.1, 30.7, 30.7, 27.7, 25.0, 21.1.
HRMS: ($ESI^+$) calculated for $C_{24}H_{26}N_3O_3$ [$M+H^+$]: 404.1969, found 404.1979.

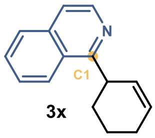

3x

**1-(cyclohex-2-en-1-yl)isoquinoline (3x)**

Prepared according to General Procedure A using isoquinoline (23.5 µL, 0.2 mmol) and cyclohexene **2a** (203 µL, 2.0 mmol). No other regioisomer was detected in the crude mixture. Purification by column chromatography ($SiO_2$, 1:99 $Et_3N$/hexanes to 1:5:94 $Et_3N$/EtOAc/hexanes) afforded product **3x** as a colorless oil (16.6 mg, 40% yield, > 20:1 *r.r.*).

$^1H$ NMR (500 MHz, $CDCl_3$) δ 8.51 (d, *J* = 5.7 Hz, 1H), 8.25 (d, *J* = 8.5 Hz, 1H), 7.82 (dt, *J* = 8.2, 1.0 Hz, 1H), 7.66 (ddd, *J* = 8.2, 6.9, 1.2 Hz, 1H), 7.58 (ddd, *J* = 8.3, 6.9, 1.4 Hz, 1H), 7.50 (dd, *J* = 5.7, 1.0 Hz, 1H), 6.04 – 5.99 (m, 1H), 5.95 (dtdd, *J* = 10.1, 2.5, 1.6, 1.1 Hz, 1H), 4.48 – 4.43 (m, 1H), 2.31 – 2.11 (m, 3H), 1.96 – 1.75 (m, 3H).
$^{13}C$ NMR (126 MHz, $CDCl_3$) δ 164.6, 142.1, 136.5, 129.6, 129.1, 128.2, 127.6, 126.9, 126.5, 125.0, 119.2, 40.3, 30.2, 24.9, 22.1.
HRMS: ($ESI^+$) calculated for $C_{15}H_{16}N$ [$M+H^+$]: 210.1277, found 210.1271.

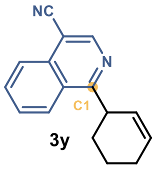

3y

**1-(cyclohex-2-en-1-yl)isoquinoline-4-carbonitrile (3y)**

Prepared according to General Procedure A using isoquinoline-4-carbonitrile (30.8 mg, 0.2 mmol) and cyclohexene **2a** (203 µL, 2.0 mmol). No other regioisomer was detected in the crude mixture. Purification by column chromatography ($SiO_2$, 10:90 EtOAc/hexanes) afforded product **3y** as a white solid (14.6 mg, 31% yield, > 20:1 *r.r.*).

$^1H$ NMR (400 MHz, $CDCl_3$) δ 8.87 (s, 1H), 8.35 (ddt, *J* = 8.5, 1.3, 0.7 Hz, 1H), 8.20 (ddd, *J* = 8.4, 1.3, 0.7 Hz, 1H), 7.89 (ddd, *J* = 8.3, 7.0, 1.3 Hz, 1H), 7.75 (dddd, *J* = 8.6, 7.0, 1.3, 0.3 Hz, 1H), 6.08 – 6.03 (m, 1H), 5.88 (dtdd, *J* = 10.1, 2.4, 1.8, 1.1 Hz, 1H), 4.54 – 4.46 (m, 1H), 2.30 – 2.12 (m, 3H), 1.97 – 1.74 (m, 3H).
$^{13}C$ NMR (101 MHz, $CDCl_3$) δ 169.9, 147.7, 135.0, 132.2, 129.0, 128.8, 127.8, 125.7, 125.6, 125.1, 116.4, 104.3, 40.7, 30.2, 24.8, 21.8.
HRMS: ($ESI^+$) calculated for $C_{16}H_{15}N_2$ [$M+H^+$]: 235.1230, found 235.1234.

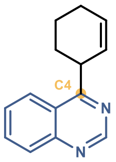

3z

**4-(cyclohex-2-en-1-yl)quinazoline (3z)**

Prepared according to General Procedure A using quinazoline (26.0 mg, 0.2 mmol) and cyclohexene **2a** (203 µL, 2.0 mmol). No other regioisomer was detected in the crude mixture. Purification by column chromatography ($SiO_2$, 1:10:89 $Et_3N$/EtOAc/hexanes) afforded product **3z** (8.3 mg, 20% yield, > 20:1 *r.r.*) as a colorless oil.

$^1H$ NMR (400 MHz, $CDCl_3$) δ 9.28 (s, 1H), 8.22 (d, *J* = 8.4 Hz, 1H), 8.06 (d, *J* = 8.5 Hz, 1H), 7.88 (ddd, *J* = 8.4, 6.9, 1.4 Hz, 1H), 7.64 (ddd, *J* = 8.3, 6.9, 1.3 Hz, 1H), 6.11 – 6.02 (m, 1H), 5.93 – 5.84 (m, 1H), 4.44 (ddp, *J* = 8.4, 5.6, 2.7 Hz, 1H), 2.31 – 2.11 (m, 3H), 2.02 – 1.71 (m, 3H).
$^{13}C$ NMR (101 MHz, $CDCl_3$) δ 174.1, 154.9, 150.2, 133.4, 129.4, 129.3, 127.5, 127.4, 124.4, 123.5, 40.0, 29.9, 24.8, 21.8.
HRMS ($ESI^+$) Calculated for $C_{14}H_{15}N_2$ $[M+H]^+$: 211.1230 found: 211.1236.

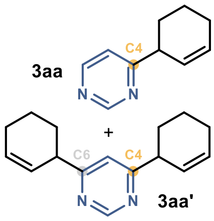


**4-(cyclohex-2-en-1-yl)pyrimidine (3aa) + 4,6-di(cyclohex-2-en-1-yl)pyrimidine (3aa')**

Prepared according to General Procedure B using pyrimidine (15.6 µL, 0.2 mmol) and cyclohexene **2a** (203 µL, 2.0 mmol). Purification by column chromatography ($SiO_2$, 1:5:94 $Et_3N$/EtOAc/hexanes) afforded product **3aa** (8.8 mg, 27% yield, > 20:1 *r.r.*) as a colorless oil.

$^1$H NMR (500 MHz, $CDCl_3$) δ 9.16 (d, *J* = 1.4 Hz, 1H), 8.65 (d, *J* = 5.2 Hz, 1H), 7.25 (dd, *J* = 5.2, 1.4 Hz, 1H), 6.08 – 5.97 (m, 1H), 5.86 – 5.75 (m, 1H), 3.59 – 3.52 (m, 1H), 2.21 – 2.07 (m, 3H), 1.81 – 1.65 (m, 3H).
$^{13}$C NMR (126 MHz, $CDCl_3$) δ 173.7, 158.8, 156.9, 130.2, 126.7, 119.5, 43.5, 29.9, 24.8, 20.8.
HRMS (ESI$^+$) Calculated for $C_{10}H_{13}N_2$ [M+H]$^+$: 161.1073 found: 161.1076.

Product **3aa'** (3.8 mg, 8% yield, > 20:1 *r.r.*) was also isolated as a colorless oil.

$^1$H NMR (500 MHz, $CDCl_3$) δ 9.09 (d, *J* = 1.3 Hz, 1H), 7.12 (d, *J* = 1.3 Hz, 1H), 6.07 – 5.99 (m, 2H), 5.82 – 5.74 (m, 2H), 3.59 – 3.47 (m, 2H), 2.22 – 2.04 (m, 6H), 1.86 – 1.63 (m, 6H).
$^{13}$C NMR (126 MHz, $CDCl_3$) δ 173.7, 158.5, 130.0, 127.1, 117.2, 43.5, 30.0, 24.9, 21.0.
HRMS (ESI$^+$) Calculated for $C_{16}H_{21}N_2$ [M+H]$^+$: 241.1699 found: 241.1699.

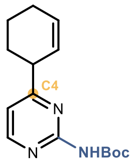


3bb

**tert-butyl (4-(cyclohex-2-en-1-yl)pyrimidin-2-yl)carbamate (3bb)**

Prepared according to General Procedure A using tert-butyl pyrimidin-2-ylcarbamate **1bb** (39.0 mg, 0.2 mmol) and cyclohexene **2a** (203 µL, 2.0 mmol). No other regioisomer was detected in the crude mixture. Purification by column chromatography ($SiO_2$, 1:10:89 $Et_3N$/EtOAc/hexanes) afforded product **3bb** (17.9 mg, 27% yield, > 20:1 *r.r.*) as a colorless oil.

$^1$H NMR (400 MHz, $CDCl_3$) δ 8.53 (d, *J* = 5.1 Hz, 1H), 7.54 (brs, 1H), 6.88 (d, *J* = 5.1 Hz, 1H), 6.04 – 5.93 (m, 1H), 5.80 – 5.72 (m, 1H), 3.54 – 3.40 (m, 1H), 2.17 – 2.04 (m, 3H), 1.82 – 1.63 (m, 3H), 1.55 (s, 9H).
$^{13}$C NMR (101 MHz, $CDCl_3$) δ 175.4, 158.2, 157.6, 150.6, 129.9, 126.9, 113.8, 81.2, 43.3, 29.6, 28.2, 24.8, 20.9.
HRMS (ESI$^+$) Calculated for $C_{15}H_{21}N_3NaO_2$ [M+Na]$^+$: 298.1526 found: 298.1522.

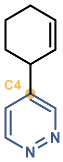


3cc

**4-(cyclohex-2-en-1-yl)pyridazine (3cc)**

Prepared according to General Procedure A using pyridazine (14.5 µL, 0.2 mmol) and cyclohexene **2a** (203 µL, 2.0 mmol). No other regioisomer was detected in the crude mixture. Purification by column chromatography ($SiO_2$, 1:40:59 $Et_3N$/EtOAc/hexanes) afforded product **3cc** (10.0 mg, 31% yield, > 20:1 *r.r.*) as a brown oil.

$^1$H NMR (400 MHz, $CDCl_3$) δ 9.10 (s, 1H), 9.08 (d, *J* = 5.5 Hz, 1H), 7.33 (dd, *J* = 5.3, 2.4 Hz, 1H), 6.05 (dq, *J* = 9.8, 3.4 Hz, 1H), 5.70 – 5.61 (m, 1H), 3.45 (m, 1H), 2.17 – 2.11 (m, 2H), 2.10 – 2.04 (m, 1H), 1.77 – 1.62 (m, 2H), 1.61 – 1.52 (m, 1H).
$^{13}$C NMR (101 MHz, $CDCl_3$) δ 152.3, 151.1, 145.6, 131.1, 126.3, 125.2, 38.8, 31.3, 24.7, 20.4.
HRMS (ESI$^+$) Calculated for $C_{10}H_{13}N_2$ [M+H]$^+$: 161.1073 found: 161.1073.

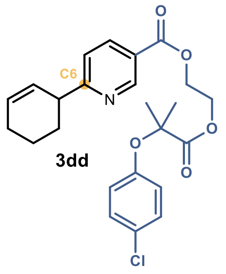


**2-((2-(4-chlorophenoxy)-2-methylpropanoyl)oxy)ethyl 6-(cyclohex-2-en-1-yl)nicotinate (3dd)**

Prepared according to General Procedure A using Etofibrate **1dd** (72.8 mg, 0.2 mmol) and cyclohexene **2a** (203 µL, 2.0 mmol). No other regioisomer was detected in the crude mixture. Purification by column chromatography ($SiO_2$, 1:10:89 $Et_3N$/EtOAc/hexanes) afforded product **3dd** as a colorless oil (47.8 mg, 54% yield, > 20:1 *r.r.*).

$^1$H NMR (400 MHz, $CDCl_3$) δ 9.10 (dd, *J* = 2.3, 0.9 Hz, 1H), 8.07 (dd, *J* = 8.2, 2.2 Hz, 1H), 7.28 (dd, *J* = 8.2, 0.9 Hz, 1H), 7.13 – 7.08 (m, 2H), 6.79 – 6.74 (m, 2H), 6.05 – 5.97 (m, 1H), 5.85 – 5.77 (m, 1H), 4.58 – 4.49 (m, 4H), 3.73 – 3.63 (m, 1H), 2.22 – 2.03 (m, 3H), 1.86 – 1.65 (m, 3H), 1.60 (s, 6H).

$^{13}$C NMR (101 MHz, $CDCl_3$) δ 173.9, 170.5, 165.1, 153.9, 150.7, 137.4, 129.6, 129.1, 127.8, 127.2, 123.1, 121.5, 120.2, 79.4, 63.0, 62.5, 44.2, 30.5, 25.3, 24.9, 21.0.

HRMS (ESI$^+$) Calculated for $C_{24}H_{27}ClNO_5$ $[M+H]^+$: 444.1572 found: 444.1565.

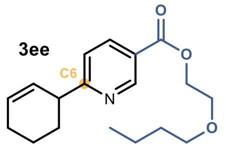


**2-butoxyethyl 6-(cyclohex-2-en-1-yl)nicotinate (3ee)**

Prepared according to General Procedure A using Nicoboxil **1ee** (44.7 mg, 0.2 mmol) and cyclohexene **2a** (203 µL, 2.0 mmol). No other regioisomer was detected in the crude mixture. Purification by column chromatography ($SiO_2$, 1:2:97 $Et_3N$/EtOAc/hexanes) afforded product **3ee** as a light-yellow oil (32.3 mg, 53% yield, > 20:1 *r.r.*).

$^1$H NMR (500 MHz, $CDCl_3$) δ 9.19 (d, *J* = 2.2 Hz, 1H), 8.25 (dd, *J* = 8.1, 2.2 Hz, 1H), 7.30 (d, *J* = 8.2 Hz, 1H), 6.05 – 5.96 (m, 1H), 5.88 – 5.79 (m, 1H), 4.53 – 4.45 (m, 2H), 3.81 – 3.75 (m, 2H), 3.72 – 3.65 (m, 1H), 3.53 (t, *J* = 6.6 Hz, 2H), 2.22 – 2.06 (m, 3H), 1.83 – 1.64 (m, 3H), 1.64 – 1.55 (m, 2H), 1.45 – 1.35 (m, 2H), 0.93 (t, *J* = 7.4 Hz, 3H).

$^{13}$C NMR (126 MHz, $CDCl_3$) δ 170.1, 165.5, 150.8, 137.6, 129.6, 127.9, 123.7, 121.4, 71.3, 68.5, 64.4, 44.2, 31.7, 30.6, 24.9, 21.0, 19.3, 13.9.

HRMS (ESI$^+$) Calculated for $C_{18}H_{26}NO_3$ $[M+H]^+$: 304.1907 found: 304.1904.

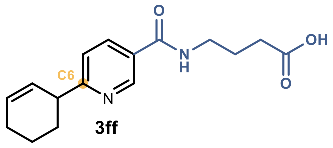


**4-(6-(cyclohex-2-en-1-yl)nicotinamido)butanoic acid (3ff)**

Prepared according to General Procedure A using Picamilon (41.6 mg, 0.2 mmol) and cyclohexene **2a** (203 µL, 2.0 mmol). No other regioisomer was detected in the crude mixture. Purification by column chromatography ($SiO_2$, 1:40:59 AcOH/EtOAc/hexanes to 1:99 AcOH/EtOAc) afforded product **3ff** as a white solid (43.4 mg, 75% yield, > 20:1 *r.r.*).

$^1$H NMR (400 MHz, Acetone) δ 9.05 (d, *J* = 2.7 Hz, 1H), 8.39 (s, 1H), 8.27 (dd, *J* = 8.2, 2.4 Hz, 1H), 7.37 (d, *J* = 8.2 Hz, 1H), 5.95 – 5.89 (m, 1H), 5.82 – 5.75 (m, 1H), 3.71 – 3.61 (m, 1H), 3.52 (q, *J* = 6.7 Hz, 2H), 2.43 (t, *J* = 7.0 Hz, 2H), 2.07 (p, *J* = 2.2 Hz, 4H), 1.93 (p, *J* = 7.1 Hz, 2H), 1.83 – 1.71 (m, 2H), 1.70 – 1.59 (m, 1H).

$^{13}$C NMR (101 MHz, Acetone) δ 147.8, 136.1, 128.8, 128.1, 121.3, 54.1, 43.5, 39.2, 24.6, 20.8.

HRMS (ESI$^+$) Calculated for $C_{16}H_{21}N_2O_3$ $[M+H]^+$: 289.1547 found: 289.1543.

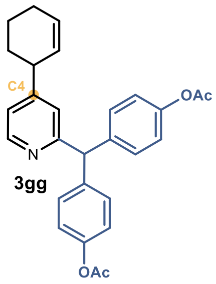
3gg

**((4-(cyclohex-2-en-1-yl)pyridin-2-yl)methylene)bis(4,1-phenylene) diacetate (3gg)**

Prepared according to General Procedure B using bisacodyl (72.3 mg, 0.2 mmol) and cyclohexene **2a** (203 µL, 2.0 mmol). No other regioisomer was detected in the crude mixture. Purification by column ($SiO_2$, 1:9:90 $Et_3N$/EtOAc/hexanes) afforded product **3gg** as a colorless oil (29.0 mg, 33% yield, > 20:1 *r.r.*).

$^{1}$H NMR (400 MHz, $CDCl_3$) δ 8.48 (d, *J* = 5.1 Hz, 1H), 7.21 – 7.17 (m, 4H), 7.03 – 6.98 (m, 6H), 5.94 – 5.89 (m, 1H), 5.63 – 5.59 (m, 2H), 3.35 – 3.30 (m, 1H), 2.27 (s, 6H), 2.09 – 2.04 (m, 2H), 2.00 – 1.94 (m, 1H), 1.73 – 1.63 (m, 2H), 1.62 – 1.59 (m, 1H), 1.52 – 1.42 (m, 1H).
$^{13}$C NMR (101 MHz, $CDCl_3$) δ 169.6, 162.5, 156.4, 149.6, 149.4, 140.4, 140.3, 130.4, 130.4, 129.8, 128.2, 123.5, 121.5, 121.2, 58.2, 41.3, 31.9, 25.0, 21.3, 21.0.
HRMS: (ESI$^+$) calculated for $C_{28}H_{28}NO_4$ [M+H$^+$]: 442.2013, found 442.2031.

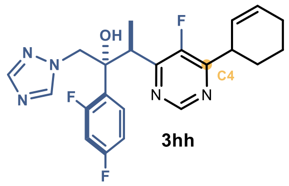
3hh

**3-(6-(cyclohex-2-en-1-yl)-5-fluoropyrimidin-4-yl)-2-(2,4-difluorophenyl)-1-(1H-1,2,4-triazol-1-yl)butan-2-ol (3hh)**

Prepared according to General Procedure A using voriconazole (69.9 mg, 0.2 mmol) and cyclohexene **2a** (203 µL, 2.0 mmol). No other regioisomer was detected in the crude mixture. Purification by column chromatography ($SiO_2$, 1:50:49 $Et_3N$/EtOAc/hexanes) afforded product **3hh** (37.6 mg, 44% yield, 1:1 *d.r.*, > 20:1 *r.r.*) as an off-white solid.

$^{1}$H NMR (400 MHz, $CDCl_3$) *1:1 mixture of diastereoisomers* δ 8.85 (dd, *J* = 7.4, 1.8 Hz, 1H), 7.99 (d, *J* = 4.3 Hz, 1H), 7.67 – 7.58 (m, 1H), 7.54 (d, *J* = 5.2 Hz, 1H), 6.90 – 6.79 (m, 2H), 6.73 (s, 0.5H), 6.68 (s, 0.5H), 6.10 – 6.02 (m, 1H), 5.77 – 5.67 (m, 1H), 4.72 (dd, *J* = 14.2, 3.5 Hz, 1H), 4.32 (dd, *J* = 17.3, 14.1 Hz, 1H), 4.19 – 4.10 (m, 1H), 4.04 – 3.92 (m, 1H), 2.27 – 2.14 (m, 2H), 2.14 – 2.05 (m, 1H), 1.99 – 1.89 (m, 1H), 1.86 – 1.71 (m, 2H), 1.12 – 1.08 (m, 3H).
$^{13}$C NMR (101 MHz, $CDCl_3$) *1:1 mixture of diastereoisomers* δ 164.1, 163.9, 162.1, 162.0, 162.0, 161.9, 161.6, 161.5, 159.8, 159.7, 158.1, 158.0, 157.4, 157.3, 155.1, 153.0, 152.9, 152.9, 152.8, 152.5, 152.5, 150.8, 144.0, 143.9, 130.7, 130.7, 130.7, 130.6, 130.3, 130.2, 125.4, 125.4, 123.6, 123.6, 111.7, 111.5, 104.3, 104.1, 103.8, 57.5, 57.5, 37.3, 37.1, 36.5, 28.2, 28.1, 24.5, 21.3, 21.2, 16.2.
$^{19}$F{$^{1}$H} NMR (376 MHz, $CDCl_3$) *1:1 mixture of diastereoisomers* δ -109.17 (d, *J* = 8.2 Hz), -109.19 (d, *J* = 8.0 Hz), -110.55 (d, *J* = 8.1 Hz), -138.87, -139.00.
HRMS (ESI$^+$) Calculated for $C_{22}H_{23}F_3N_5O$ [M+H]$^+$: 430.1849 found: 430.1854.

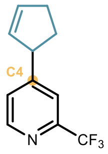
3ii

**4-(cyclopent-2-en-1-yl)-2-(trifluoromethyl)pyridine (3ii)**

Prepared according to General Procedure A using 2-(trifluoromethyl)pyridine (23.0 µL, 0.2 mmol) and cyclopentene (183 µL, 2.0 mmol). No other regioisomer was detected in the crude mixture. Purification by column chromatography ($SiO_2$, 5:95 EtOAc/hexanes) afforded product **3ii** as colorless oil (17.1 mg, 40% yield, > 20:1 *r.r.*).

$^{1}$H NMR (400 MHz, $CDCl_3$) δ 8.60 (d, *J* = 5.0 Hz, 1H), 7.49 (d, *J* = 1.7 Hz, 1H), 7.30 (dd, *J* = 5.0, 1.7 Hz, 1H), 6.09 – 6.05 (m, 1H), 5.76 – 5.72 (m, 1H), 4.00 – 3.93 (m, 1H), 2.60 – 2.40 (m, 3H), 1.77 – 1.67 (m, 1H).

$^{13}$C NMR (101 MHz, $CDCl_3$) δ 157.5, 150.0, 148.4 (q, $J$ = 34.1 Hz), 134.4, 131.6, 125.2, 121.7 (q, $J$ = 274.2 Hz), 119.3 (q, $J$ = 2.8 Hz), 50.6, 33.1, 32.4.
$^{19}$F NMR (376 MHz, $CDCl_3$) δ -68.0.
HRMS: ($ESI^+$) calculated for $C_{11}H_{11}F_3N$ $[M+H]^+$: 214.0838, found 214.0829.

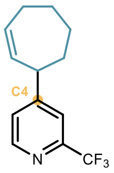

**3jj**

**4-(cyclohept-2-en-1-yl)-2-(trifluoromethyl)pyridine (3jj)**

Prepared according to General Procedure A using 2-(trifluoromethyl)pyridine (23.0 µL, 0.2 mmol) and cycloheptene (233 µL, 2.0 mmol) with a reaction time of 24 hours. No other regioisomer was detected in the crude mixture. Purification by column chromatography ($SiO_2$, 5:95 EtOAc/hexanes) afforded product **3jj** as colorless oil (21.2 mg, 44% yield, > 20:1 *r.r.*).

$^{1}$H NMR (400 MHz, $CDCl_3$) δ 8.62 (d, $J$ = 5.0 Hz, 1H), 7.55 (d, $J$ = 2.2 Hz, 1H), 7.35 (dd, $J$ = 5.0, 1.9 Hz, 1H), 5.96 (dddd, $J$ = 11.3, 6.9, 5.5, 2.3 Hz, 1H), 5.64 (dddt, $J$ = 11.3, 4.2, 1.9, 0.8 Hz, 1H), 3.66 – 3.60 (m, 1H), 2.29 – 2.19 (m, 2H), 1.97 – 1.88 (m, 1H), 1.88 – 1.66 (m, 4H), 1.53 – 1.42 (m, 1H).
$^{13}$C NMR (101 MHz, $CDCl_3$) δ 158.2, 150.1, 148.4 (q, $J$ = 34.1 Hz), 133.9, 133.6, 125.3, 121.7 (q, $J$ = 274.5 Hz), 119.5 (q, $J$ = 2.9 Hz), 46.3, 35.2, 29.7, 28.7, 26.6.
$^{19}$F NMR (376 MHz, $CDCl_3$) δ -68.03.
HRMS: ($ESI^+$) calculated for $C_{13}H_{15}F_3N$ $[M+H]^+$: 242.1151, found 242.1157.

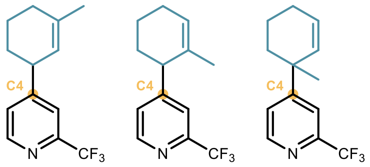

**3kk** **3kk'** **3kk"**

**4-(3-methylcyclohex-2-en-1-yl)-2-(trifluoromethyl)pyridine (3kk) + 4-(2-methylcyclohex-2-en-1-yl)-2-(trifluoromethyl)pyridine (3kk’) + 4-(1-methylcyclohex-2-en-1-yl)-2-(trifluoromethyl)pyridine (3kk’’)**

Prepared according to General Procedure A using 2-(trifluoromethyl)pyridine (23.0 µL, 0.2 mmol) and 1-methylcyclohex-1-ene (237 µL, 2.0 mmol). Purification by column chromatography ($SiO_2$, 1:99 $Et_3N$/hexanes) afforded a mixture of products **3kk, 3kk’, and 3kk’’** as a colorless oil (29.7 mg, 61% yield, 2:0.75:1 allylic *r.r.*). For NMR characterization, when possible, the signals are assigned to the corresponding isomer (COSY and HSQC spectra used for assignment).

$^{1}$H NMR (400 MHz, $CDCl_3$) δ 8.61 (dm, $J$ = 5.0 Hz, 1H, **3kk’**), 8.61 (dm, $J$ = 5.0 Hz, 1H, **3kk’’**), 8.60 (dp, $J$ = 5.0 Hz, 0.7 Hz, 1H, **3kk**), 7.65 (dd, $J$ = 1.8, 0.8 Hz, 1H, **3kk’’**), 7.52 – 7.51 (m, 1H, **3kk**), 7.51 – 7.50 (m, 1H, **3kk’**), 7.46 (ddq, $J$ = 5.2, 1.8, 0.6 Hz, 1H, **3kk’’**), 7.32 (ddt, $J$ = 5.0, 1.7, 0.6 Hz, 1H, **3kk**), 7.30 (ddt, $J$ = 5.0 Hz, 1.6, 0.5 Hz, 1H, **3kk’**), 5.96 (dtd, $J$ = 10.1, 3.7, 0.6 Hz, 1H, **3kk’’**), 5.79 (tp, $J$ = 3.9, 1.4 Hz, 1H, **3kk’**), 5.64 (dtd, $J$ = 10.1, 2.2, 1.1 Hz, 1H, **3kk’’**), 5.36 (ddtd, $J$ = 2.9, 2.2, 1.5, 0.7 Hz, 1H, **3kk**), 3.45 (ddp, $J$ = 8.0, 5.5, 2.5 Hz, 1H, **3kk**), 3.32 (t, $J$ = 5.9 Hz, 1H, **3kk’**), 2.15 – 1.26 (m, 6H, $CH_2$) 1.77 – 1.76 (m, 3H, **3kk**), 1.50 – 1.49 (m, 3H, **3kk’**), 1.40 (s, 3H, **3kk’’**)
$^{13}$C NMR (101 MHz, $CDCl_3$) δ 161.0, 158.2, 156.7, 149.8, 149.8, 149.7, 148.2 (q, $J$ = 34.0 Hz), 148.2 (q, $J$ = 34.0 Hz, **3kk**), 148.1 (q, $J$ = 33.6 Hz), 137.9, 132.5 (**3kk’’**), 131.9, 128.9 (**3kk’’**), 126.4 (**3kk’**), 125.8 (q, $J$ = 1.0 Hz), 124.6 (q, $J$ = 1.1 Hz, **3kk’’**), 121.8 (q, $J$ = 273.9 Hz, **3kk’’**), 121.7 (q, $J$ = 274.2 Hz, **3kk**), 121.7 (q, $J$ = 274.1 Hz, **3kk’**), 121.4 (**3kk**), 120.4 (q, $J$ = 2.5 Hz), 119.8 (q, $J$ = 2.5 Hz), 118.6 (q, $J$ = 2.8 Hz), 45.4 (**3kk’**), 41.6 (**3kk**), 39.7 (**3kk’’**), 38.3, 31.9, 31.5 (**3kk**), 29.7, 28.5 (**3kk’’**), 25.1, 24.8, 23.9 (**3kk**), 22.5 (**3kk’**), 21.1 (**3kk**), 18.8, 18.3 (**3kk’**).
$^{19}$F NMR (376 MHz, $CDCl_3$) δ 67.9 (**3kk’’**), 68.0 (**3kk’**), 68.0 (**3kk**)

HRMS: (ESI$^+$) calculated for $C_{13}H_{15}F_3N$ [M+H]$^+$: 242.1151, found 242.1152.

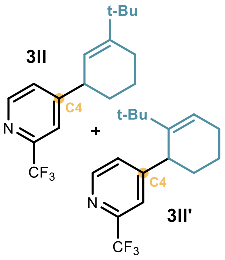


**4-(3-(tert-butyl)cyclohex-2-en-1-yl)-2-(trifluoromethyl)pyridine (3ll) and 4-(2-(tert-butyl)cyclohex-2-en-1-yl)-2-(trifluoromethyl)pyridine (3ll')**

Prepared according to General Procedure A using 2-(trifluoromethyl)pyridine (23.0 µL, 0.2 mmol) and 1-(tert-butyl)cyclohex-1-ene (330 µL, 2.0 mmol). Purification by column chromatography ($SiO_2$, 5:95 EtOAc/hexanes) afforded products **3ll** + **3ll'** as colorless oil (25.8 mg, 46% yield, 8:1 allylic *r.r.*). Only characterization data for the major product **3ll** is described.

$^1$H NMR (400 MHz, $CDCl_3$) δ 8.61 (dt, $J$ = 5.0, 0.7 Hz, 1H), 7.50 (dt, $J$ = 1.5, 0.7 Hz, 1H), 7.31 (ddt, $J$ = 5.0, 1.7, 0.7 Hz, 1H), 5.44 – 5.42 (m, 1H), 3.49 (ddq, $J$ = 8.4, 5.5, 2.7 Hz, 1H), 2.16 – 2.09 (m, 2H), 2.00 (dtd, $J$ = 13.1, 6.6, 2.8 Hz, 1H), 1.79 – 1.69 (m, 1H), 1.65 – 1.55 (m, 1H), 1.44 (dddd, $J$ = 13.1, 10.6, 8.2, 3.0 Hz, 1H), 1.09 (s, 9H).
$^{13}$C NMR (101 MHz, $CDCl_3$) δ 158.5, 149.8, 149.5, 148.2 (q, $J$ = 34.0 Hz), 125.7, 121.7 (q, $J$ = 273.9 Hz), 119.9 (q, $J$ = 2.8 Hz), 41.6, 35.6, 31.6, 30.0, 29.1, 24.3, 21.5.
$^{19}$F NMR (376 MHz, $CDCl_3$) δ -68.0.
HRMS: (ESI$^+$) calculated for $C_{16}H_{21}F_3N$ [M+H]$^+$: 284.1621, found 284.1623.

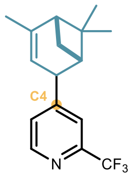

3mm

**2-(trifluoromethyl)-4-(4,6,6-trimethylbicyclo[3.1.1]hept-3-en-2-yl)pyridine (3mm)**

Prepared according to General Procedure A using 2-(trifluoromethyl)pyridine (23.0 µL, 0.2 mmol) and alpha-Pinene (318 µL, 2.0 mmol) with a reaction time of 60 hours. The yield (39%, single regioisomer) of **3mm** was inferred by $^1$H NMR analysis of the crude reaction mixture using trichloroethylene as the internal standard. A 90% pure sample of **3mm** (estimated by $^1$H NMR integration) was obtained after purification by column chromatography ($SiO_2$, 3:97 $Et_2O$/hexanes) as a colorless oil.

$^1$H NMR (400 MHz, $CDCl_3$) δ 8.60 (dt, $J$ = 5.0, 0.7 Hz, 1H), 7.54 (dt, $J$ = 1.6, 0.7 Hz, 1H), 7.35 (ddt, $J$ = 5.1, 1.7, 0.7 Hz, 1H), 5.32 (td, $J$ = 2.9, 1.6 Hz, 1H), 3.62 (h, $J$ = 2.3 Hz, 1H), 2.17 – 2.13 (m, 1H), 1.81 (t, $J$ = 1.9 Hz, 3H), 1.35 (s, 3H), 1.21 – 1.14 (m, 1H), 1.01 (s, 3H).
$^{13}$C NMR (101 MHz, $CDCl_3$) δ 156.3, 149.6, 148.6, 148.0 (q, $J$ = 34.3 Hz), 126.4, 121.8 (q, $J$ = 274.1 Hz), 120.5 (q, $J$ = 2.6 Hz), 115.6, 47.6, 46.8, 45.1, 42.1, 26.3, 26.2, 23.2, 20.5.
$^{19}$F NMR (376 MHz, $CDCl_3$) δ -67.5.
HRMS: (ESI$^+$) calculated for $C_{16}H_{19}F_3N$ [M+H]$^+$: 282.1464, found 282.1473.

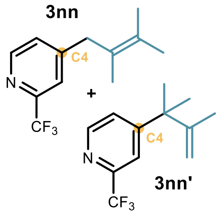


**4-(2,3-dimethylbut-2-en-1-yl)-2-(trifluoromethyl)pyridine (3nn) and 4-(2,3-dimethylbut-3-en-2-yl)-2-(trifluoromethyl)pyridine (3nn')**

Prepared according to General Procedure A using 2-(trifluoromethyl)pyridine (23.0 µL, 0.2 mmol) and tetramethylethylene (238 µL, 2.0 mmol) with a reaction time of 60 hours. Purification by column chromatography ($SiO_2$, 5:95 EtOAc) afforded the mixture of products **3nn** + **3nn'** as a colorless oil (22.4 mg, 43% yield, 3:2 allylic *r.r.*).

$^1$H NMR of **3nn** (400 MHz, $CDCl_3$) δ 8.58 (dt, $J$ = 5.0, 0.7 Hz, 1H), 7.47 – 7.42 (m, 1H), 7.27 – 7.24 (m, 1H), 3.47 – 3.45 (m, 2H), 1.76 – 1.75 (m, 6H), 1.63 – 1.56 (m, 3H).

$^{13}$C NMR of **3nn** (101 MHz, $CDCl_3$) δ 152.4, 149.8, 148.2 (q, *J* = 34.2 Hz), 128.5, 126.3 (q, *J* = 1.1 Hz), 123.6, 121.7 (q, *J* = 274.5 Hz), 120.5 (q, *J* = 2.7 Hz), 39.6, 20.7, 20.6, 18.5.
$^{19}$F NMR of **3nn** (376 MHz, $CDCl_3$) δ -68.1.

$^{1}$H NMR of **3nn'** (400 MHz, $CDCl_3$) δ 8.62 (dp, *J* = 5.1, 0.6 Hz, 1H), 7.59 (dd, *J* = 1.8, 0.7 Hz, 1H), 7.41 (ddq, *J* = 5.1, 1.8, 0.6 Hz, 1H), 5.03 (dq, *J* = 1.4, 0.7 Hz, 1H), 5.00 (p, *J* = 1.4 Hz, 1H), 1.52 (dd, *J* = 1.4, 0.7 Hz, 3H), 1.44 (s, 6H).
$^{13}$C NMR of **3nn'** (101 MHz, $CDCl_3$) δ 159.9, 149.9, 149.8, 148.3 (q, *J* = 34.0 Hz), 124.1 (q, *J* = 1.1 Hz), 121.7 (q, *J* = 274.1 Hz), 118.2 (q, *J* = 2.7 Hz), 111.7, 44.2, 27.6, 20.0.
$^{19}$F NMR of **3nn'** (376 MHz, $CDCl_3$) δ -67.9.
HRMS of **3nn** + **3nn'**: (ESI$^+$) calculated for $C_{12}H_{15}F_3N$ [M+H]$^+$: 230.1151, found 230.1161.

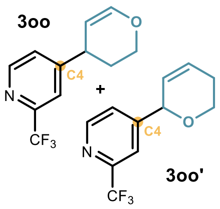


**4-(3,4-dihydro-2H-pyran-4-yl)-2-(trifluoromethyl)pyridine (3oo) + 4-(5,6-dihydro-2H-pyran-2-yl)-2-(trifluoromethyl)pyridine (3oo')**

Prepared according to General Procedure A using 2-(trifluoromethyl)pyridine (23.0 µL, 0.2 mmol) and 3,4-dihydropyran (182 µL, 2.0 mmol). Purification by column chromatography ($SiO_2$, 10:90 EtOAc/hexanes) afforded products **3oo** + **3oo'** as a colorless oil (19.8 mg, 43% yield, 7:1 allylic *r.r.*). Only the major product **3oo** is described.

$^{1}$H NMR (400 MHz, $CDCl_3$) δ 8.65 – 8.63 (m, 1H), 7.60 – 7.58 (m, 1H), 7.40 (m, 1H), 6.64 (dd, *J* = 6.3, 1.9 Hz, 1H), 4.72 (dddd, *J* = 6.3, 3.3, 1.0, 0.7 Hz, 1H), 4.03 (dddt, *J* = 11.0, 8.0, 3.0, 0.4 Hz, 1H), 3.96 (ddd, *J* = 11.1, 6.9, 3.2 Hz, 1H), 2.25 (dddddd, *J* = 13.8, 6.9, 6.3, 3.0, 1.1, 0.5 Hz, 1H), 1.84 (m, 1H).
$^{13}$C NMR (101 MHz, $CDCl_3$) δ 156.6, 150.0, 148.4 (q, *J* = 34.3 Hz), 146.3, 125.6, 121.6 (q, *J* = 274.2 Hz), 119.7 (q, *J* = 2.7 Hz), 100.7, 63.4, 35.8, 31.1.
$^{19}$F NMR (376 MHz, $CDCl_3$) δ -68.0.
HRMS: (ESI$^+$) calculated for $C_{11}H_{11}F_3NO$ [M+H]$^+$: 230.0787, found 230.0788.

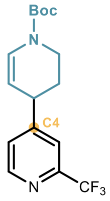

3pp

**tert-butyl 2'-(trifluoromethyl)-3,4-dihydro-[4,4'-bipyridine]-1(2H)-carboxylate (3pp)**

Prepared according to General Procedure A using 2-(trifluoromethyl)pyridine 23.0 µL, 0.2 mmol) and tert-butyl 3,4-dihydropyridine-1(2H)-carboxylate (367 mg, 2.0 mmol). Purification by column chromatography ($SiO_2$, 5:95 to 12:88 EtOAc/hexanes) afforded product **3pp** as colorless oil (24.8 mg, 38% yield, 20:1 allylic *r.r.*) and recovered olefin (311 mg, 1.7 mmol). Due to presence of N-Boc rotamers the $^{1}$H NMR and $^{13}$C NMR spectra were recorded at 328K which produced singular broad singlets for these signals.

$^{1}$H NMR (500 MHz, $CDCl_3$, 328K) δ 8.63 (d, *J* = 5.0 Hz, 1H), 7.55 (d, *J* = 2.0 Hz, 1H), 7.36 (dd, *J* = 5.0, 1.9 Hz, 1H), 7.08 (br. s, 1H), 4.82 (br. s, 1H), 3.67 – 3.51 (m, 3H), 2.19 (dtd, *J* = 13.4, 6.2, 3.6 Hz, 1H), 1.82 (dtd, *J* = 13.5, 7.4, 4.0 Hz, 1H), 1.52 (s, 9H).
$^{13}$C NMR (126 MHz, $CDCl_3$, 328K) δ 156.3, 152.2, 150.1, 148.8 (q, *J* = 34.5 Hz), 128.1, 125.5, 121.7 (q, *J* = 274.3 Hz), 119.7 (q, *J* = 2.7 Hz), 104.5, 81.3, 39.7, 37.8, 30.4, 28.3.
$^{19}$F NMR (376 MHz, $CDCl_3$) δ -68.0.
HRMS: (ESI$^+$) calculated for $C_{16}H_{20}F_3N_2O_2$ [M+H]$^+$: 329.1471, found 329.1471.

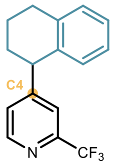

3qq

**4-(1,2,3,4-tetrahydronaphthalen-1-yl)-2-(trifluoromethyl)pyridine (3qq)**

Prepared according to General Procedure A using 2-(trifluoromethyl)pyridine (23.0 µL, 0.2 mmol) and 1,2,3,4-tetrahydronaphthalene (273 µL, 2.0 mmol). No other regioisomer was detected in the crude mixture. Purification by column chromatography ($SiO_2$, 1:100 $Et_3N$/hexanes to 1:3:96 $Et_3N$/EtOAc/hexanes) afforded product **3qq** as pale-yellow oil (27.3 mg, 49% yield, > 20:1 *r.r.*).

$^{1}$H NMR (400 MHz, $CDCl_3$) δ 8.60 (d, *J* = 5.0 Hz, 1H), 7.46 (d, *J* = 1.7 Hz, 1H), 7.21 – 7.17 (m, 3H), 7.12 – 7.05 (dt, *J* = 7.7, 4.2 Hz, 1H), 6.75 (dd, *J* = 7.6, 1.0 Hz, 1H), 4.23 (t, *J* = 6.4 Hz, 1H), 3.00 – 2.82 (m, 2H), 2.27 – 2.17 (m, 1H), 1.90 – 1.77 (m, 3H).

$^{13}$C NMR (101 MHz, $CDCl_3$) δ 158.5, 149.9, 148.3 (q, *J* = 34.1 Hz), 137.6, 136.2, 129.8, 129.5, 126.9, 126.7, 126.1, 121.6 (q, *J* = 274.3 Hz), 120.6 (q, *J* = 2.7 Hz), 45.1, 32.6, 29.4, 20.4.

$^{19}$F NMR (376 MHz, $CDCl_3$) δ -68.0.

HRMS: ($ESI^+$) calculated for $C_{16}H_{15}F_3N$ $[M+H]^+$: 278.1151, found 278.1152.

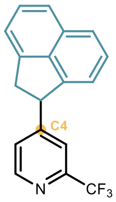

3rr

**4-(1,2-dihydroacenaphthylen-1-yl)-2-(trifluoromethyl)pyridine (3rr)**

Prepared according to General Procedure A using 2-(trifluoromethyl)pyridine (23.0 µL, 0.2 mmol) and 1,2-dihydroacenaphthylene (308 mg, 2.0 mmol). No other regioisomer was detected in the crude mixture. Purification by column chromatography ($SiO_2$, 5:95 EtOAc/hexanes) afforded product **3rr** as colorless oil (25.4 mg, 42% yield, > 20:1 *r.r.*).

$^{1}$H NMR (400 MHz, $CDCl_3$) δ 8.66 – 8.62 (m, 1H), 7.75 (t, *J* = 7.2 Hz, 2H), 7.60 – 7.54 (m, 2H), 7.51 (t, *J* = 7.6 Hz, 1H), 7.37 (d, *J* = 7.1 Hz, 1H), 7.31 – 7.27 (m, 1H), 7.12 (d, *J* = 7.1 Hz, 1H), 5.00 – 4.94 (m, 1H), 4.04 (dd, *J* = 17.6, 8.8 Hz, 1H), 3.39 (d, *J* = 17.3 Hz, 1H).

$^{13}$C NMR (101 MHz, $CDCl_3$) δ 156.5, 150.3, 148.6 (q, *J* = 34.3 Hz), 145.8, 142.5, 138.6, 131.6, 128.4, 128.1, 125.5, 123.9, 123.0, 121.5 (q, *J* = 274.3 Hz), 120.2, 119.8, 119.7 (q, *J* = 2.8 Hz), 48.7, 41.0.

$^{19}$F NMR (376 MHz, $CDCl_3$) δ -67.6.

HRMS: ($ESI^+$) calculated for $C_{18}H_{13}F_3N$ $[M+H]^+$: 300.0995, found 300.0997.

## E.3 Reaction scale-up

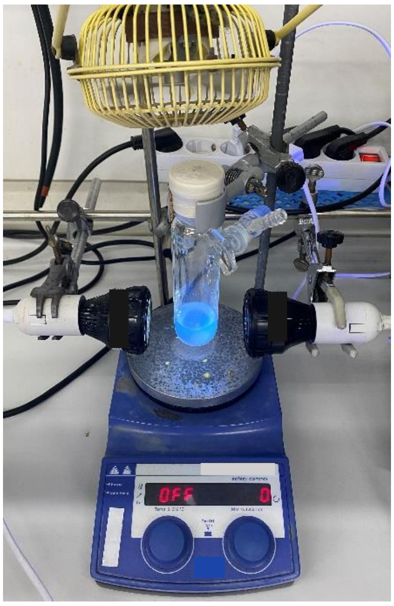

**Figure S9**. 1 mmol scale reaction using two 365 nm EvoluChem lamps.

To an argon-purged glass vial, containing the dithiophosphoric acid catalyst **A2** (127 mg, 0.2 mmol), and pyridine **1a** (81 µL, 1 mmol), was added 2,4,6-collidine (66 µL, 0.5 mmol), followed by cyclohexene **2a** (1 mL, 10 mmol) and argon-sparged HPLC grade acetone (20 mL, 0.05 M). The vial was sealed with Parafilm, and placed in the 365 nm irradiation setup as shown on Figure S9. The reaction was stirred for 16h, then the solvent was evaporated. The regioisomeric ratio **3a/3a'** (2.6:1) of the crude mixture was measured by $^{1}$H NMR analysis. Purification by column chromatography ($SiO_2$, 1:10:89 $Et_3N$/EtOAc/hexanes) afforded product **3a** as a light-yellow oil (81.2 mg, 51% yield, > 20:1 *r.r.*). The minor regioisomer **3a'** was not isolated after column chromatography.

## F. Mechanistic Studies

### F.1 Electrochemical Studies

For all cyclic voltammetry (CV) measurements, a platinum disk electrode (diameter 3 mm) was used as working electrode. A silver wire coated with AgCl immersed in a 3 M aqueous solution of NaCl and separated from the analyte by a fritted glass disk was employed as the reference electrode. A Pt wire counter-electrode completed the electrochemical setup. The scan rate used in each CV experiment is indicated case by case. Potentials are quoted with the following notation: $E_p^C$ refers to the cathodic peak potential, $E_p^A$ refers to the anodic peak potential

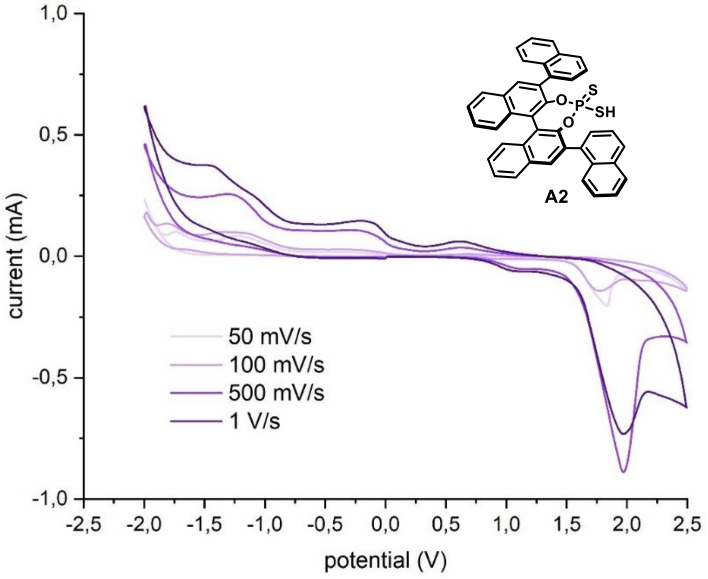


**Figure S10.** Cyclic voltammogram for catalyst **A2** [0.02M] in [0.1 M] $TBAPF_6$ in $CH_3CN$. Measurement started by oxidation from 0 to +2.0 V and finishing at 0 V. Platinum disk working electrode, Ag/AgCl (NaCl 3 M) reference electrode, Pt wire auxiliary electrode. One irreversible oxidation observed at +1.12 V.

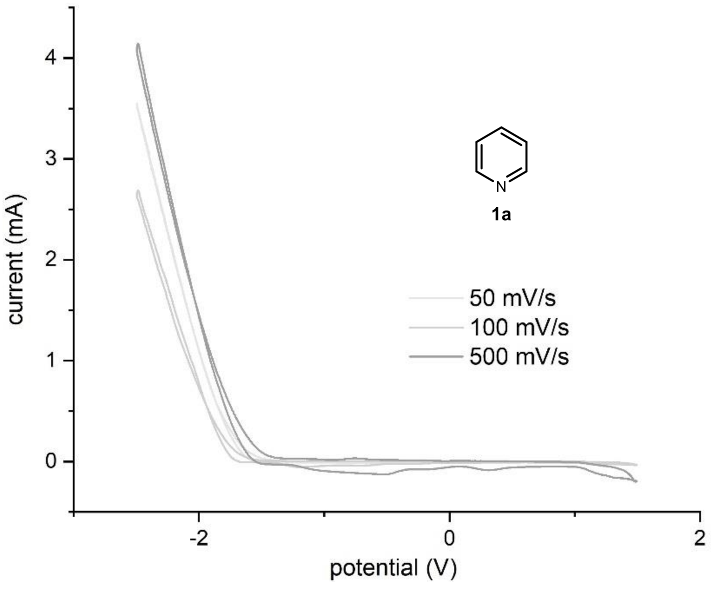


**Figure S11.** Cyclic voltammogram for pyridine **1a** [0.025M] in [0.1 M] $TBAPF_6$ in $CH_3CN$. Measurement started by reduction from 0 to -2.5 V and finishing at 0 V. Platinum disk working electrode, Ag/AgCl (NaCl 3 M) reference electrode, Pt wire auxiliary electrode. No reduction wave was observed.

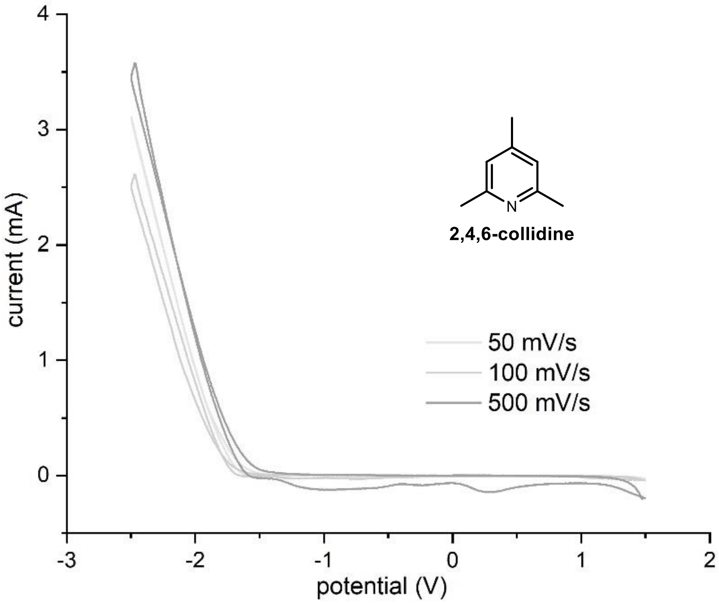


**Figure S12.** Cyclic voltammogram for 2,4,6-collidine [0.025M] in [0.1 M] $TBAPF_6$ in $CH_3CN$. Measurement started by reduction from 0 to -2.5 V and finishing at 0 V. Platinum disk working electrode, Ag/AgCl (NaCl 3 M) reference electrode, Pt wire auxiliary electrode. No reduction wave was observed.

The reduction potential of pyridinium **I** was first measured using pyridinium hydrochloride **1a.HCl** (Figure S13). While a reversible reduction wave attributed to **I** was observed at -0.6V, another wave attributed to the oxidation of the chloride counterion was also detected at +1.3 V.[15]

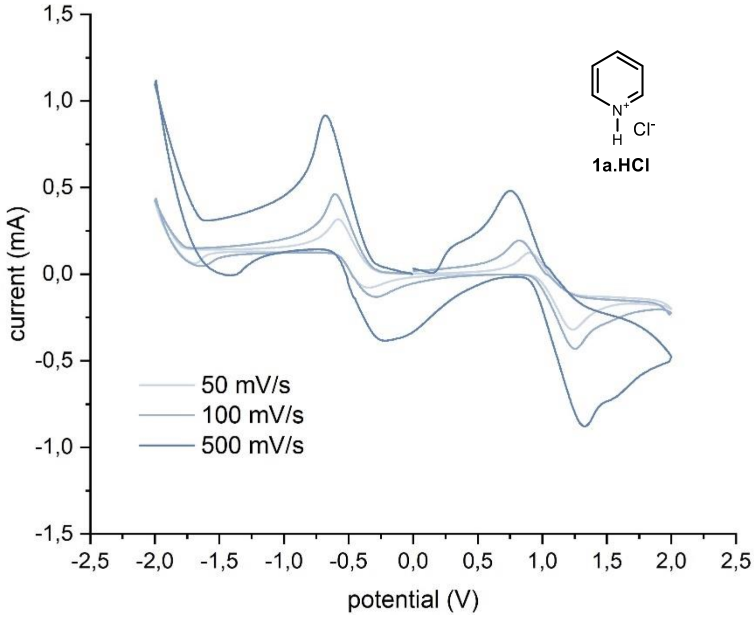


**Figure S13.** Cyclic voltammogram for **1a·HCl** [0.025M] in [0.1 M] $TBAPF_6$ in $CH_3CN$. Measurement started by reduction from 0 to -2.0 V and finishing at 0 V. Platinum disk working electrode, Ag/AgCl (NaCl 3 M) reference electrode, Pt wire auxiliary electrode. One reversible reduction wave observed at -0.6 V, and one oxidation wave observed at +1.3 V.

To avoid this additional oxidation wave, we decided to use the trifluoroacetate salts of both pyridine and 2,4,6-collidine instead (for $TFA^-$ $E_p^{ox} > +2.4$ V vs SCE).[16] As shown in Figures S15 and S16, reversible reduction waves are observed for both **I** and 2,4,6-collidinium.

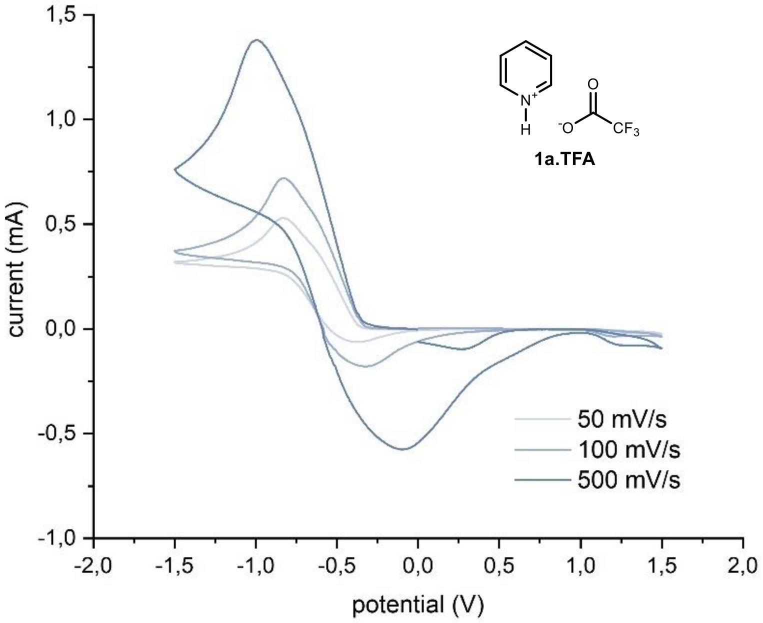


**Figure S14.** Cyclic voltammogram for **1a·TFA** [0.025M] in [0.1 M] $TBAPF_6$ in $CH_3CN$. Measurement started by reduction from 0 to -1.5 V and finishing at 0 V. Platinum disk working electrode, Ag/AgCl (NaCl 3 M) reference electrode, Pt wire auxiliary electrode. One reversible reduction wave observed at -0.6 V.

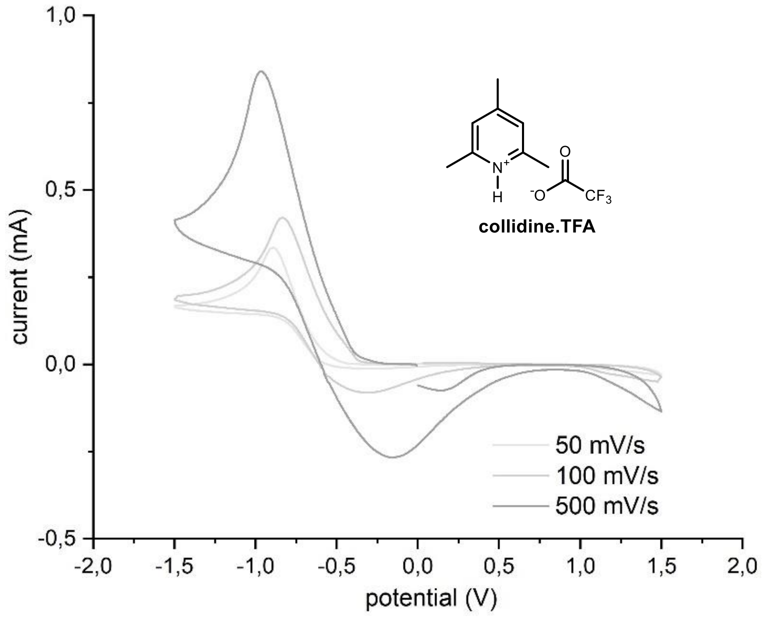


**Figure S15.** Cyclic voltammogram for collidine·TFA [0.025M] in [0.1 M] $TBAPF_6$ in $CH_3CN$. Measurement started by reduction from 0 to -1.5 V and finishing at 0 V. Platinum disk working electrode, Ag/AgCl (NaCl 3 M) reference electrode, Pt wire auxiliary electrode. One reversible reduction wave observed at -0.6 V.

### F.2 Absorption Spectroscopy Analysis

The absorption profiles of the different reaction component were recorded in order to identify the photoactive species (Figure S16). A 1:1 mixture of **A1** + **1a** (green line) displayed the same absorption as **A1**$^{-}$ (light blue line) thus excluding the formation of EDA complexes between **A1** and pyridine **1a**.

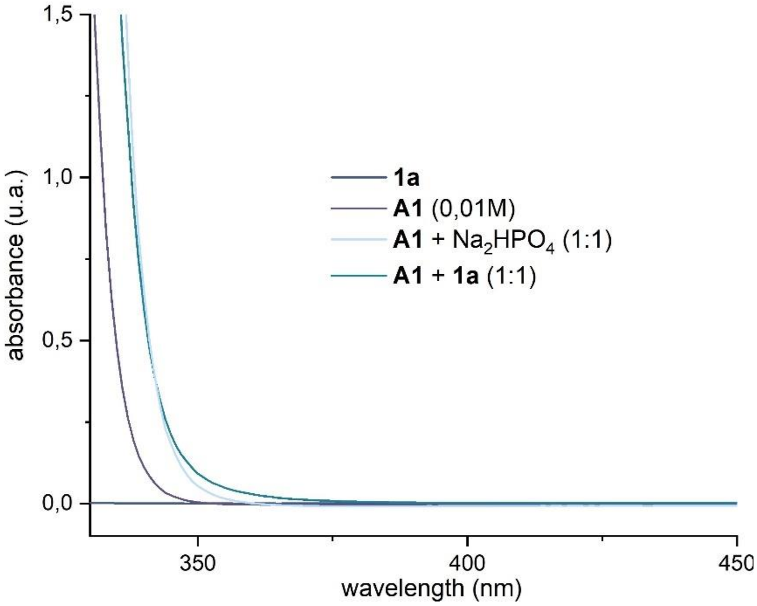


**Figure S16**. Optical absorption spectra of the initial reaction components, recorded in acetone in 1 mm path quartz cuvettes using a Shimadzu 2401PC UV−vis spectrophotometer.

UV-vis analysis of the reaction components from the optimized conditions did not show the formation of an EDA complex either (Figure S17).

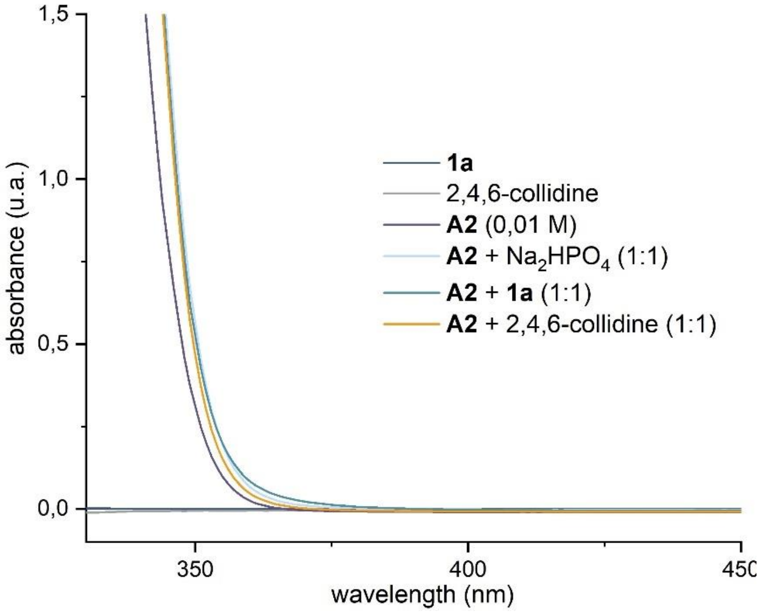


**Figure S17**. Optical absorption spectra of the reaction components under optimized conditions, recorded in acetone in 1 mm path quartz cuvettes using a Shimadzu 2401PC UV−vis spectrophotometer.

### F.3 Evaluation of the excited-state potential of A2$^-$

Using the data collected from the cyclic voltammetry (CV) studies (Section F.1) and from the absorption spectra (Section F.2) of catalyst **A2**, we could estimate the redox potential relative to its excited state **[A2$^-$]$^*$**, by means of the following Equation 1:[17]

$$E(\mathbf{A2}^{\bullet}/[\mathbf{A2}^{-}]^{*})) = E(\mathbf{A2}^{\bullet}/\mathbf{A2}^{-}) - E_{0\text{-}0}([\mathbf{A2}^{-}]^{*})\ /\mathbf{A2}^{-})\ \text{[Eq. 1]}$$

Since the electrochemical oxidation of **A2$^-$** 1a is irreversible (Figure S10), the irreversible peak potential $E_p^{anode}$ was used for E(**A2$^•$/A2$^-$**). $E_{0\text{-}0}$([**A2$^-$]*/A2$^-$**), which is the excited state energy of the anion of the catalyst **A2**, was estimated spectroscopically from the position of the long wavelength tail of the absorption spectrum recorded in acetone (370 nm, Figures S15).

For the catalyst **A2**, the $E_p^{anode}$, which provides the E(**A2$^•$/A2$^-$**), is *1.12 V* (Figures S10), while the position of the long wavelength tail of the absorption spectrum corresponds to *370 nm* (Figure S10), which translates into an $E_{0\text{-}0}$(**[A2$^-$]$^*$**/**A2$^-$**) of *3.350 eV.*

$$E(\mathbf{A2}^{\bullet}/[\mathbf{A2}^{-}]^{*}) = 1.12 - 3.35 = \mathbf{-2.23\ V}\ \text{(vs Ag/AgCl)}$$

### F.4 Transient Absorption Spectroscopy (TAS)

Studies with microsecond transient absorption spectroscopy (TAS) were performed using an excitation source of NdYAG (neodymium-doped yttrium aluminium garnet) Opolette laser with an optical parametric oscillator (OPO) system that allows variable wavelength excitation from 400 -1800 nm, pulse width of 6 ns, up to 2 mJ of energy from OPO output with fiber optic coupled, and high energy output from direct NdYAG harmonics 355 (20 mJ, 5 ns) and 532 (45mJ, 6 ns). The system is completed with 150 W tungsten lamp as probe; 2 monochromators Minuteman MM151; Si amplified photodetector module for VIS; DSPDAU high speed data rate recorder and interface software from RAMDSP. Laser intensity for the chosen wavelength was 355 nm – 1.30 mJ.

In a typical transient absorption spectroscopy experiment, a solution of catalyst **A2** in acetone was prepared under an argon atmosphere and transferred into a screw-top 3.0 mL quartz cuvette for measurement. Upon irradiation with the appropriated wavelength, the decay of absorption at 625 nm of the transient excited state [**A2$^-$]$^*$** was recorded. The same signal was observed upon irradiation from 400 nm to 800 nm but in lower intensity and higher noise.

As shown on Figure S18, **[A2$^-$]$^*$** displayed a first order exponential decay with a half-lifetime of ~5 µs.

The same TAS experiment performed on catalyst **A1** did not show any detectable transient, suggesting that the 3,3’-substitution pattern may play a role in increasing the excited-state lifetime of the dithiophosphoric acid catalyst.

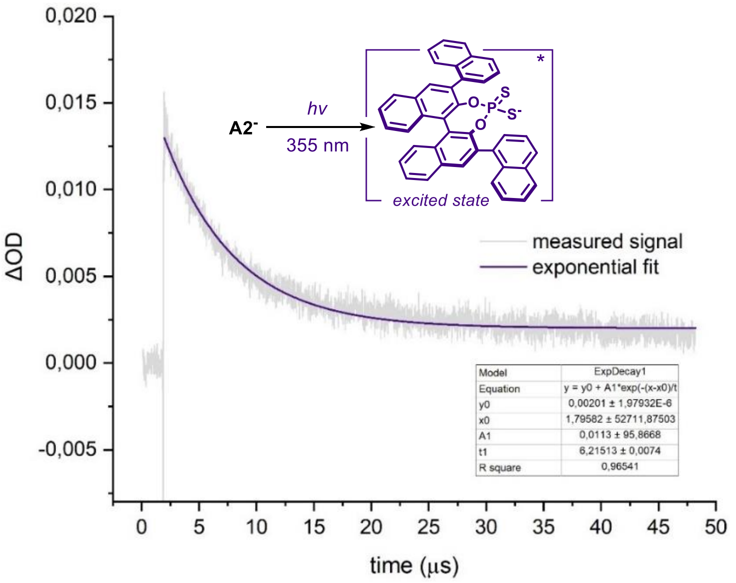


**Figure S18.** Absorption at 625 nm of the transient excited state of **A2**$^-$ generated upon 355 nm laser excitation. [**A2**] = 0.01 M in acetone. A first order exponential fit (purple line) was applied to the signal to facilitate the lifetime measurement. ΔOD: optical density variation.

## F.5 Emission Spectrum and Stern-Volmer Quenching Studies

### F.5.1 Emission spectrum of [A2$^-$]$^*$

The emission spectra were recorded in a Fluorolog Horiba Jobin Yvon spectrofluorimeter equipped with a photomultiplier detector, a double monochromator, and a 350W xenon light source. 2 mL of a 5 mM solution of **A2·Et$_3$N** (obtained by dissolving **A2** and $Et_3N$ in $CH_2Cl_2$ in a 1:1 ratio, followed by solvent removal) in thoroughly degassed acetone were placed in a 10 x10 mm light path quartz fluorescence cuvette equipped with Silicone/PTFE 3.2 mm septum under an argon atmosphere. The excitation wavelength was fixed at 350 nm (incident light slit regulated to 5 mm), while the emission light was acquired from 355 nm to 500 nm (emission light slit regulated to 5 mm). A solvent blank was subtracted from all the measurements.

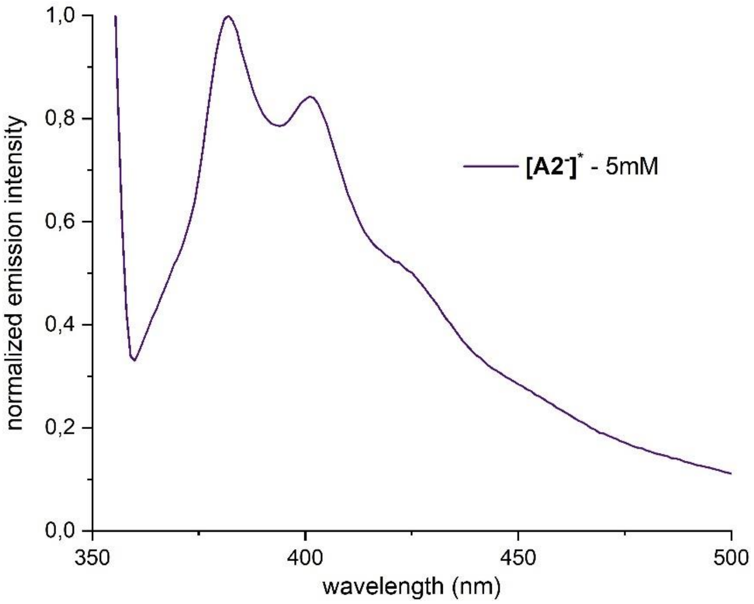


**Figure S19.** Normalized emission of [**A2**$^-$]$^*$ upon 350 nm irradiation in acetone.

### F.5.1.1 Stern-Volmer Quenching Studies using 1a.HCl as the quencher

A $5.10^{-2}$ M solution of **1a·HCl** in acetone was prepared, and 5 μL of this stock solution were added to the solution of catalyst salt **A2·Et$_3$N**, prepared as described above. The addition of this solution of **1a·HCl** was repeated five consecutive times. After each addition, an absorption spectrum and an emission spectrum of the solution were recorded. The excitation wavelength was fixed at 350 nm (incident light slit regulated to 5 mm); the emission light was acquired from 355 nm to 500 nm (emission light slit regulated to 5 mm). A solvent blank was subtracted from all the measurements. The excitation wavelength was chosen in order to avoid saturation of the emission detector. The results shown in Figure S20 indicate that pyridinium **I** quenches the excited state of **A2$^-$** and its emission. No change in the absorption spectra of the solution was observed during the addition of **1a·HCl**.

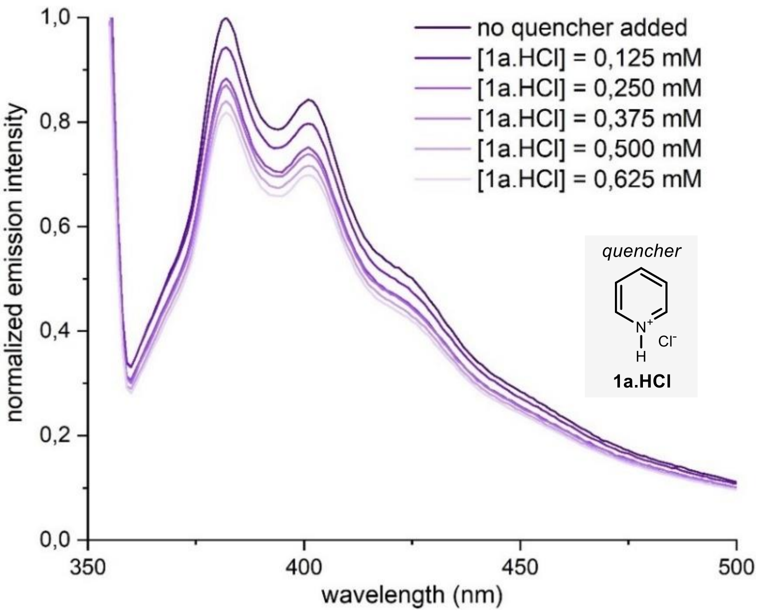


**Figure S20.** Quenching of the emission of $[A2^-]^*$ (5 mM in acetone) in the presence of increasing amounts of 1a.HCl.

The Stern-Volmer plot, reported in Figure S21, shows a linear correlation between the amounts of **1a·HCl** and the ratio $I^0/I$. On the basis of the following Equation 1, it is possible to calculate the Stern-Volmer constant $K_{SV}$.[18]

$$I^0/I = 1 + K_{SV}[Q] \quad [Eq. 1]$$

We calculated a Stern-Volmer quenching constant of **346.4 M$^{-1}$**.

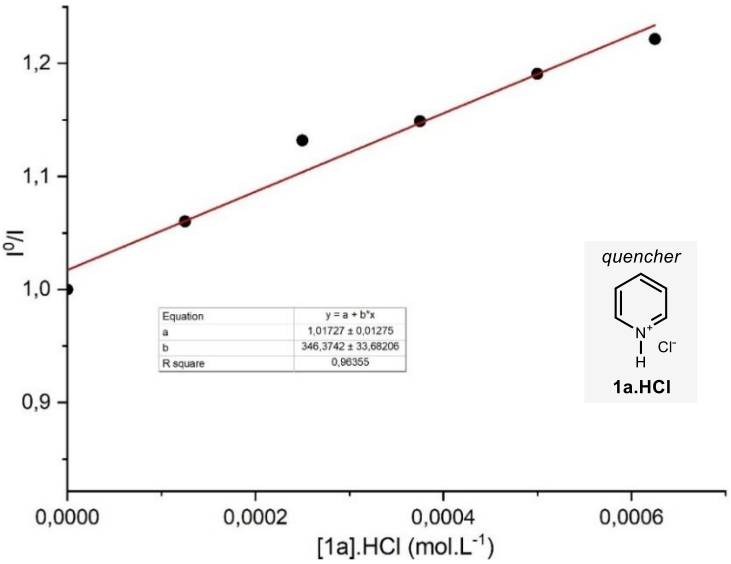


**Figure S21.** Stern-Volmer quenching plot using **1a·HCl** as a quencher.

### F.5.1.2 Stern-Volmer Quenching Studies using collidine·HCl as the quencher

A 2.10$^{-2}$ M solution of 2,4,6-collidine hydrochloride in acetone was prepared, and 10 μL of this stock solution were added to the solution of catalyst salt **A2·Et$_3$N**, prepared as described above. The addition of this 2,4,6-collidine hydrochloride solution was repeated five consecutive times. After each addition, an absorption spectrum and an emission spectrum of the solution were recorded. The excitation wavelength was fixed at 350 nm (incident light slit regulated to 5 mm); the emission light was acquired from 355 nm to 500 nm (emission light slit regulated to 5 mm). A solvent blank was subtracted from all the measurements. The excitation wavelength was chosen in order to avoid saturation of the emission detector. The results shown in Figure S22 indicate that 2,4,6-collidinium quenches the excited state of **A2$^-$** and its emission. No change in the absorption spectra of the solution was observed during the addition of 2,4,6-collidine hydrochloride.

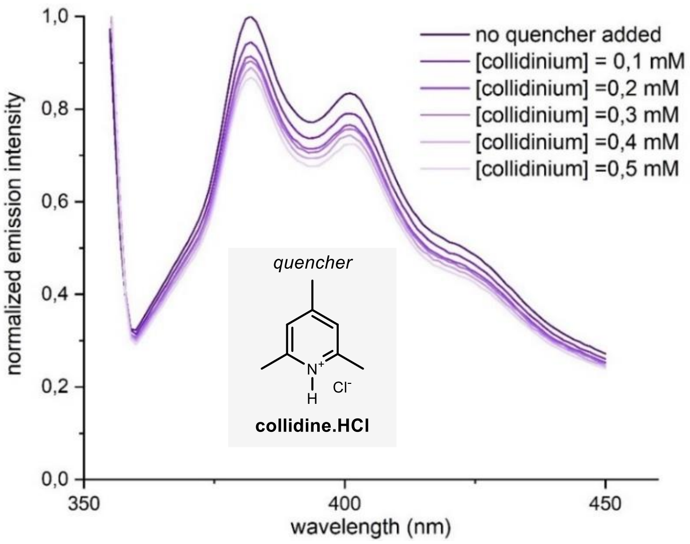


**Figure S22.** Quenching of the emission of [A2$^-$]$^*$ (5 mM in acetone) in the presence of increasing amounts of collidine·HCl.

The Stern-Volmer plot, reported in Figure S23, shows a linear correlation between the amounts of 2,4,6-collidine hydrochloride and the ratio $I^0/I$. On the basis of the following Equation 1, it is possible to calculate the Stern-Volmer constant $K_{SV}$.[18]

$$I^0/I = 1 + K_{SV}[Q] \quad [Eq. 1]$$

We calculated a Stern-Volmer quenching constant of **283.4 $M^{-1}$**.

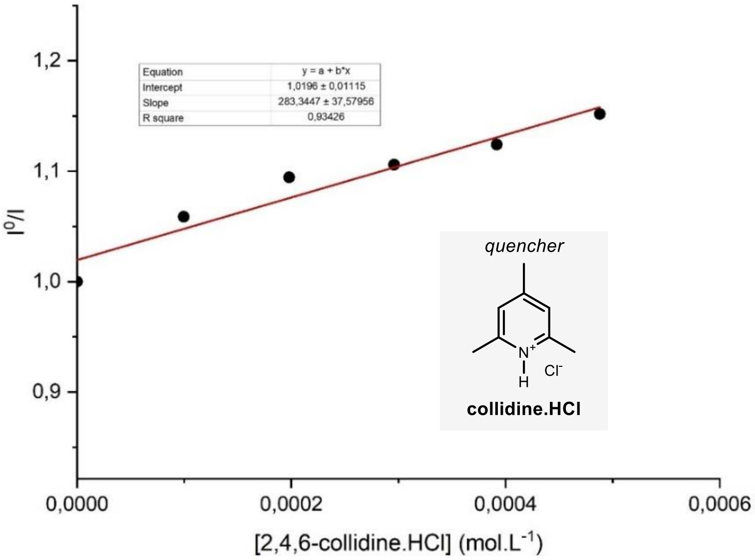


**Figure S23.** Stern-Volmer quenching plot using collidine·HCl as a quencher.

## F.5.2 Emission Spectrum of $[Ir(ppy)_2dtbbpy]PF_6$

The emission spectra were recorded in a Fluorolog Horiba Jobin Yvon spectrofluorimeter equipped with a photomultiplier detector, a double monochromator, and a 350W xenon light source. 10 µL of a 1 mM solution of $[Ir(ppy)_2dtbbpy]PF_6$ in thoroughly degassed acetone were placed in a 10 x10 mm light path quartz fluorescence cuvette equipped with Silicone/PTFE 3.2 mm septum under an argon atmosphere. 2 mL acetone were added to this solution to reach a final concentration of 5 µM. The excitation wavelength was fixed at 390 nm (incident light slit regulated to 5 mm), while the emission light was acquired from 450 nm to 680 nm (emission light slit regulated to 5 mm). A solvent blank was subtracted from all the measurements.

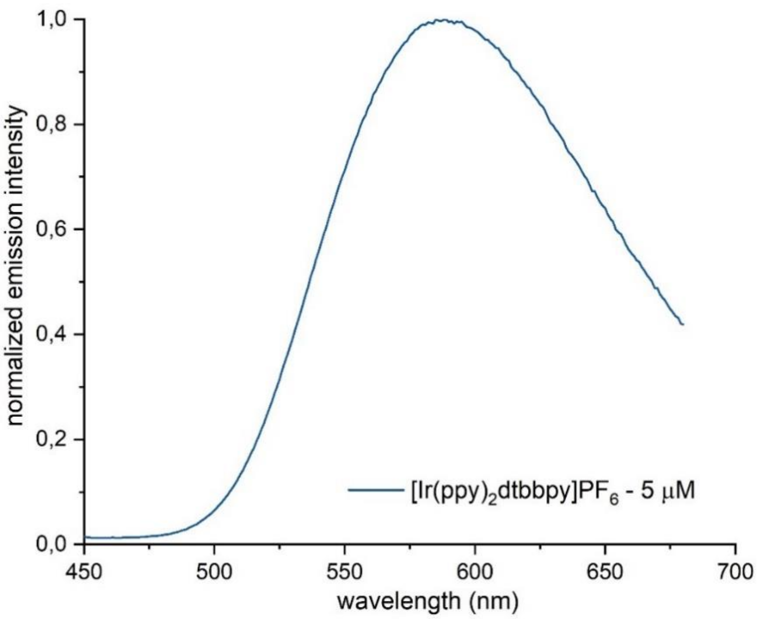


**Figure S24.** Normalized emission of [Ir(ppy)$_2$dtbbpy]$PF_6$ upon 390 nm irradiation in acetone.

### F.5.2.1 Stern-Volmer Quenching Studies using 1a·HCl as the quencher

A 0.1 M solution of **1a·HCl** in acetone was prepared, and 5 μL of this stock solution were added to the solution of [Ir(ppy)$_2$dtbbpy]$PF_6$, prepared as described above. The addition of this solution of **1a·HCl** was repeated five consecutive times. After each addition, an absorption spectrum and an emission spectrum of the solution were recorded. The excitation wavelength was fixed at 390 nm (incident light slit regulated to 5 mm); the emission light was acquired from 450 nm to 680 nm (emission light slit regulated to 5 mm). A solvent blank was subtracted from all the measurements. The excitation wavelength was chosen in order to avoid saturation of the emission detector. The results shown in Figure S25 indicate that pyridinium **I** quenches the excited state of [Ir(ppy)$_2$dtbbpy]$PF_6$ and its emission. No change in the absorption spectra of the solution was observed during the addition of **1a·HCl**.

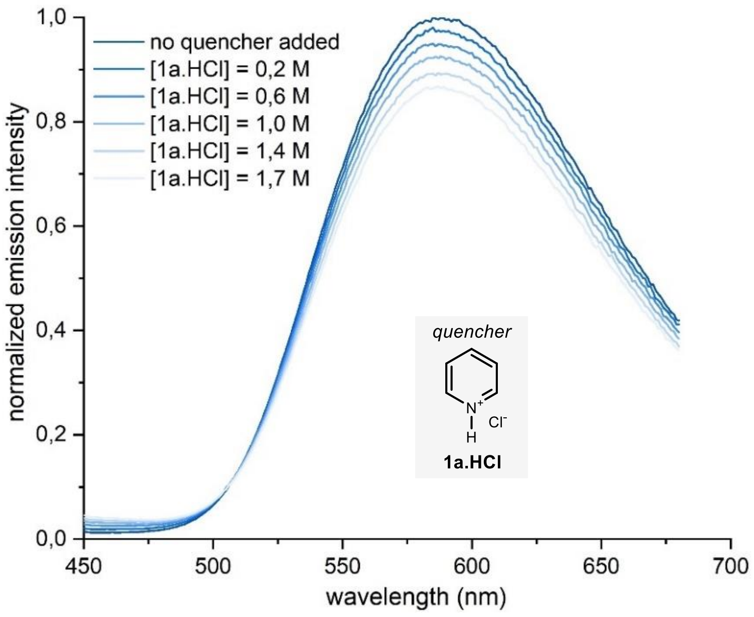


**Figure S25.** Quenching of the emission of $[Ir(ppy)_2dtbbpy]PF_6$ (5 µM in acetone) in the presence of increasing amounts of **1a·HCl**.

The Stern-Volmer plot, reported in Figure S26, shows a linear correlation between the amounts of **1a.HCl** and the ratio $I^0/I$. On the basis of the following Equation 1, it is possible to calculate the Stern-Volmer constant $K_{SV}$.[18]

$$I^0/I = 1 + K_{SV}[Q] \quad [Eq. 1]$$

We calculated a Stern-Volmer quenching constant of **90.0 M$^{-1}$**.

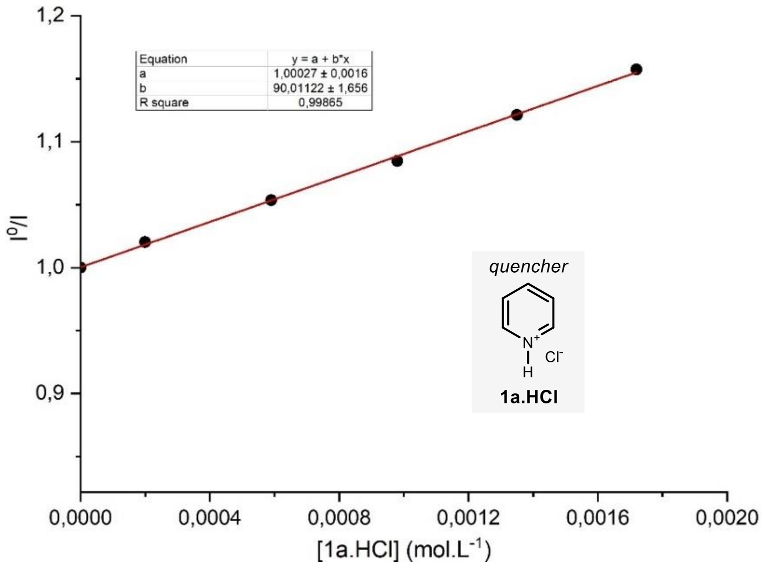


**Figure S26.** Stern-Volmer quenching plot using **1a·HCl** as a quencher.

## F.6 Electron Paramagnetic Resonance (EPR)

Continuous wave (CW) EPR spectra were acquired on a Bruker EMX Micro X-band spectrometer operating at 9.385e$^9$ using a Bruker ER 1164 HS resonator. A 150 mL Suprasil offset liquid nitrogen dewar flask (Wilmad-LabGlass) was used for low-temperature measurements. Individual EPR tubes were filled with ~0.7 mL of the solution and were placed at the same position of the resonant cavity for EPR spectral acquisition. The spectral data were collected at 77 K with the following spectrometer settings: microwave power = 0.5305 mW; center field = 3354.2 G, sweep width = 300 G, sweep time = 22.5 s, modulation frequency = 100 KHz, modulation amplitude = 1 G, power attenuation = 25 dB, time constant = 0.01 ms.

The pyridinyl radical **II** was generated by premixing pyridine **1a** (8 µL, 0.100 mmol) with catalyst **A2** (12.7 mg, 0.020 mmol) in 0.7 mL methanol in an EPR tube under inert atmosphere. The EPR sample was measured first in the absence of light at 77K using liquid $N_2$. As expected, no signal was observed. The same sample was allowed to reach room temperature and was irradiated for 15 min using a 365PF EvoluChem™ LED spotlight. The sample was immediately frozen using liquid $N_2$ and was inserted into the EPR cavity. A new signal at 3347.4 G was observed with a g-value of 2.00324 (Figure S27).

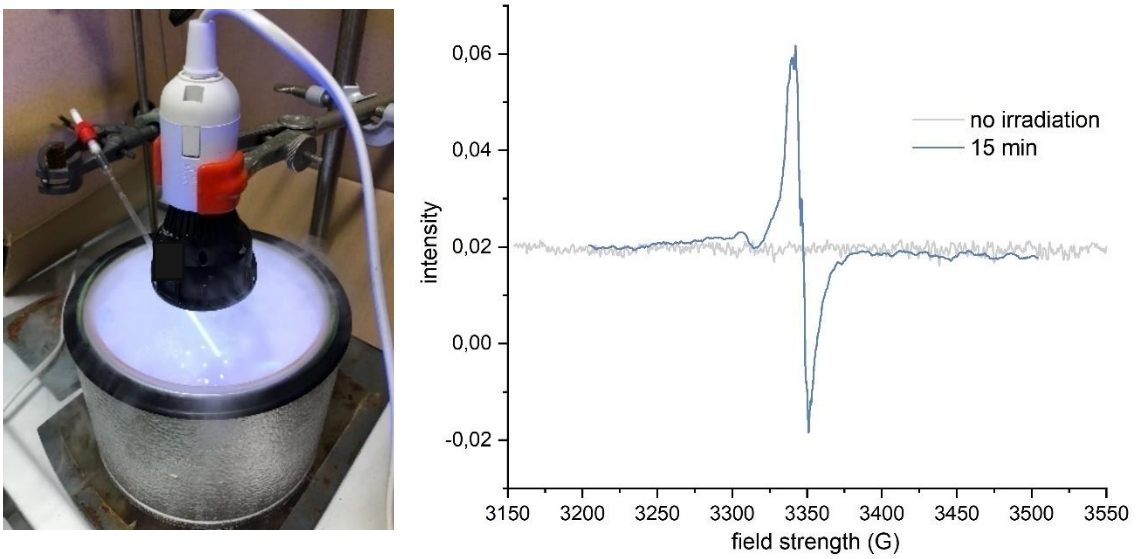


**Figure S27.** Experimental setup for the EPR experiment (*left*) and measured EPR spectra of a 5:1 mixture of **1a** and **A2** in methanol in the absence and after 15 min of irradiation (*right*).

The measured g-value of 2.00324 is in accordance with reports from the literature for the same radical.[19] The hyperfine coupling constants could not be calculated due to the poor resolution of the spectrum.

## F.7 Radical Clock Experiments

### *GP5* – General procedure for the radical clock experiments

To an argon-purged glass vial, containing the dithiophosphoric acid catalyst **A2** (12.7 mg, 0.02 mmol), and the appropriate (2,2-diphenylcyclopropyl)pyridine **4** (0.1 mmol), was added 2,4,6-collidine (6.6 µL, 0.05 mmol), followed by cyclohexene **2a** (101 µL, 1.0 mmol) and argon-sparged HPLC grade acetone (2 mL, 0.05M). The vial was sealed with Parafilm, and placed in the 365 nm irradiation setup. The reaction was stirred for 16h, then the solvent was evaporated and the crude mixture purified by flash column chromatography on silica gel.

The formation of products **5a** and **5b** supports the formation of the delocalized pyridinyl radical **II** which then undergoes a rapid radical ring-opening to generate the highly stabilized bis-benzylic radical **IV**. The latter is then able to couple with a cyclohexene radical **III** generated in the meantime (Figure S28).

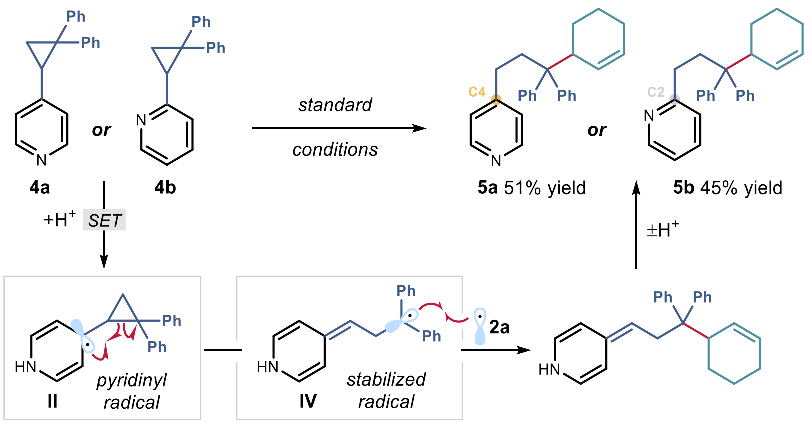


**Figure S28.** Radical clock experiments to probe the formation of the key pyridinyl radical II.

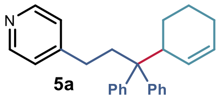


**4-(3-(cyclohex-2-en-1-yl)-3,3-diphenylpropyl)pyridine (5a)** Prepared according to GP5 using 4-(2,2-diphenylcyclopropyl)pyridine **4a** (27.1 mg, 0.1 mmol). Purification by column chromatography ($SiO_2$, 1:10:89 $Et_3N$/EtOAc/hexanes) afforded product **5a** (18.0 mg, 51% yield) as a colorless oil.

$^1$H NMR (400 MHz, $CDCl_3$) δ 8.44 – 8.42 (m, 2H), 7.33 – 7.20 (m, 10H), 6.98 – 6.95 (m, 2H), 5.84 (dp, *J* = 10.4, 2.1 Hz, 1H), 5.61 (ddt, *J* = 10.2, 5.0, 2.5 Hz, 1H), 3.26 (dtq, *J* = 9.8, 4.8, 2.4 Hz, 1H), 2.47 – 2.33 (m, 2H), 2.31 – 2.15 (m, 2H), 1.93 – 1.82 (m, 2H), 1.73 – 1.48 (m, 4H).
$^{13}$C NMR (101 MHz, $CDCl_3$) δ 151.9, 149.6, 145.6, 143.3, 129.7, 129.4, 129.0, 129.0, 127.7, 127.1, 126.1, 126.0, 123.7, 54.4, 41.1, 40.5, 30.5, 25.0, 24.8, 22.4.
HRMS (ESI$^+$) Calculated for $C_{26}H_{28}N$ $[M+H]^+$: 354.2216, found: 354.2216.

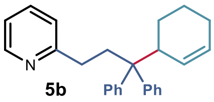


**2-(3-(cyclohex-2-en-1-yl)-3,3-diphenylpropyl)pyridine (5b)** Prepared according to GP5 using 2-(2,2-diphenylcyclopropyl)pyridine **4b** (27.1 mg, 0.1 mmol). Purification by column chromatography ($SiO_2$, 1:10:89 $Et_3N$/EtOAc/hexanes) afforded product **5b** (15.8 mg, 45% yield) as a colorless oil.

$^1$H NMR (400 MHz, $CDCl_3$) δ 8.53 (d, *J* = 4.0 Hz, 1H), 7.54 (td, *J* = 7.7, 1.9 Hz, 1H), 7.35 – 7.28 (m, 8H), 7.27 – 7.20 (m, 2H), 7.09 (dd, *J* = 6.4, 4.9 Hz, 1H), 6.94 (d, *J* = 7.8 Hz, 1H), 5.92 – 5.84 (m, 1H), 5.66 – 5.58 (m, 1H), 3.36 – 3.26 (m, 1H), 2.66 – 2.36 (m, 4H), 2.03 – 1.94 (m, 1H), 1.92 – 1.82 (m, 1H), 1.76 – 1.61 (m, 2H), 1.62 – 1.47 (m, 2H).

$^{13}$C NMR (101 MHz, $CDCl_3$) δ 162.6, 149.1, 145.9, 143.6, 136.3, 129.9, 129.5, 129.4, 128.7, 127.7, 127.0, 125.9, 125.8, 122.7, 120.9, 54.4, 41.4, 39.5, 33.6, 29.7, 25.1, 24.8, 22.5.
HRMS ($ESI^+$) Calculated for $C_{26}H_{28}N$ $[M+H]^+$: 354.2216, found: 354.2218.

### F.8 Discussion on the role of collidine

2,4,6-Collidine has a positive effect on the model reaction. In contrast, acidic additives, including acetic acid and trifluoroacetic acid, were detrimental.

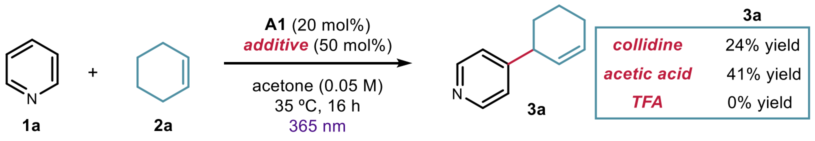


Since collidine is slightly more basic than most of the pyridine substrates and products **3**, it is likely that a dynamic equilibrium between these partially protonated bases allows the reaction to proceed. To better elucidate the role of collidine, we performed cyclic voltammetry studies (see section F1) and quenching experiments (see section F5), which showed that collidine behaves similarly to pyridine: collidinium **V** can be reversibly reduced at -0.6 V and quenches the excited state of catalyst **A2** to generate a collidinyl radical **VI**, although at a lower rate than pyridinium ($K_{SV}$ = 283.4 $M^{-1}$ for **V** against $K_{SV}$ = 346.4 $M^{-1}$ for **I**). However, radical coupling of **VI** with an allylic radical is likely hampered by the methyl groups at *ortho* and *para* positions, thus avoiding any by-product formation. This led us to believe that collidine may act as an electron reservoir and redox mediator in the reaction (Figure S29). Accordingly, if **VI** was to be formed, it could not couple with an allylic radical **III** because of steric hindrance, but it could reduce pyridinium **I** to the desired pyridinyl radical **II**. Similar redox mediation by collidine has been observed previously.[20]

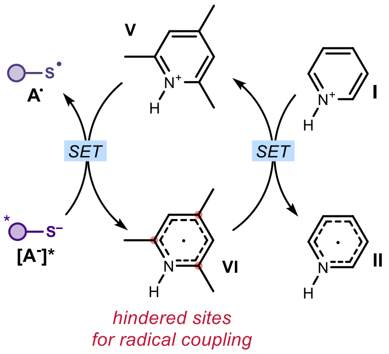


**Figure S29.** Possible role of collidine as a redox mediator.

### F.9 The aromatization step: Oxidation of the dihydropyridine intermediate

As depicted in Figure S30, the proposed radical coupling between **II** and **III** should afford the dihydropyridine **VII** as the formal product of the reaction. However, our attempts of detecting the proposed dihydropyridine intermediate **VII** in the reaction mixture remained unsuccessful, and the aromatized pyridine products **3** were detected instead.

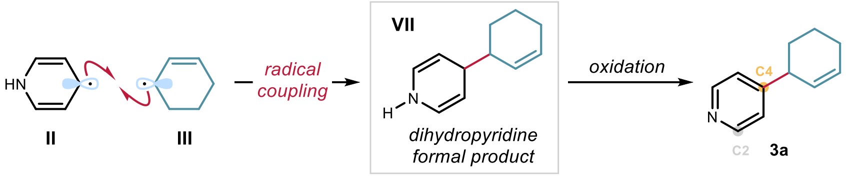


**Figure S30.** Radical coupling between **II** and **III** leads to the formation of a dihydropyridine intermediate **VII**.

During our studies, we did observe the formation of isopropanol via $^{1}H$ NMR analysis of the crude reaction. We surmised that isopropanol most likely came from the reduction of acetone, the solvent of the reaction. This observation is congruent with acetone acting as the oxidant (Scheme S6), but the process through which dihydropyridine **VII** was oxidizes needed clarification.

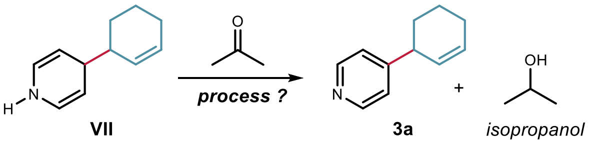


**Scheme S6.** Oxidation of **VII** by acetone and formation of isopropanol.

While the direct reduction of acetone by the catalyst's excited state **[A2$^{-}$]$^{*}$** is thermodynamically unfavorable (for cyclohexanone, $E^{ox}$ = -2.33 V vs SCE),[21] the SET event leading to the formation of the ketyl radical **IX** is feasible for protonated acetone **VIII**[22] (formed under the action of the acidic catalyst). The resulting thyil radical could then abstract the C4 hydrogen atom from the dihydropyridine intermediate **VII** ((BDE (S-H) = 83.3 kcal mol$^{-1}$ for **A1**,[2] and for NADH analogues BDE(C4-H) ~ 69 kcal mol$^{-1}$).[23] After HAT, the ketyl (**IX**) and pyridinyl (**X**) radicals would ultimately afford isopropanol and the final aromatized product **3** (Figure S31).

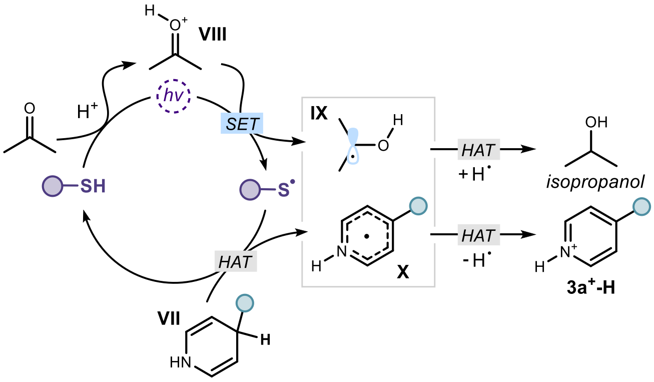


**Figure S31.** Tentative explanation for the re-aromatization of **VII**.

This scenario was tested by submitting a stable dihydropyridine derivative, 9,10-dihydroacridine, to the reaction conditions (Scheme S7). Full consumption of the starting 9,10-dihydroacridine was observed, along with the formation of acridine in 60% yield and isopropanol in 40% yield. In the absence of light or catalyst, no conversion of the dihydroacridine was observed.

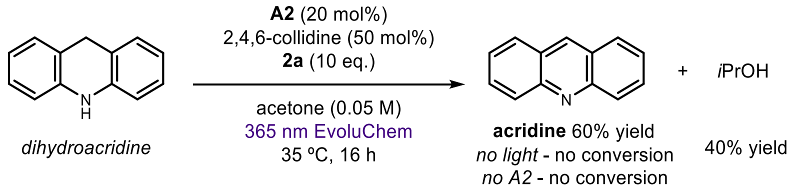


**Scheme S7.** Oxidation of 9,10-dihydroacridine and reduction of acetone under the reaction conditions.

To an argon-purged glass vial, containing the dithiophosphoric acid catalyst **A2** (12.7 mg, 0.02 mmol), and 9,10-dihydroacridine (18.1 mg, 0.1 mmol) was added 2,4,6-collidine (6.6 µL, 0.05 mmol), followed by cyclohexene **2a** (101 µL, 1.0 mmol) followed argon-sparged HPLC grade acetone (2 mL, 0.05 M). The vial was sealed with Parafilm, and placed in the 365 nm irradiation setup. The reaction was stirred for 16 h, before being diluted with 1 mL $CDCl_3$. The crude mixture was then analyzed by $^1$H NMR using trichloroethylene as an internal standard.

To confirm that the excited catalyst can reduce acetone, a last experiment was conducted, excluding both 9,10-dihydroacridine and 2,4,6-collidine (Scheme S8). 7% of dimer of **2a** and 17% of *i*PrOH were formed during the reaction. The formation of isopropanol under these conditions supports the ability of the excited **A2**$^-$ to reduce acetone.

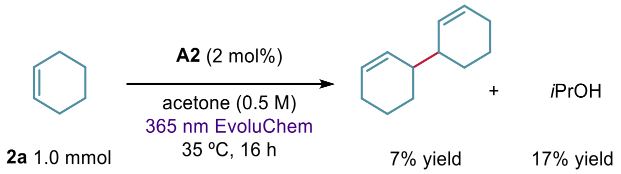


**Scheme S8.** Reduction of acetone to isopropanol by [**A2**$^-$]$^*$.

To an argon-purged glass vial, containing the dithiophosphoric acid catalyst **A2** (12.7 mg, 0.02 mmol), and cyclohexene **2a** (101 µL, 1.0 mmol) was added argon-sparged HPLC grade acetone (2 mL, 0.5 M). The vial was sealed with Parafilm, and placed in the 365 nm irradiation setup. The reaction was stirred for 16h, before being diluted with 1 mL $CDCl_3$. The crude mixture was then analyzed by $^1$H NMR using trichloroethylene as an internal standard.

Overall, these results support the existence of the second catalytic cycle depicted in Figure S31.

## G. Computational Studies

All calculations were performed using Gaussian 09W (version 7.0).[24] Gaussview 5.0.9[25] was used to generate input files for geometry and energy optimizations, and to visualize the output files. Spin density contour maps were generated from output (.chk) files. Mulliken spin densities were obtained directly from the output (.chk) files. NBO analysis was performed after full geometry and energy optimization. All calculations were performed without geometry restrictions and using unrestricted B3LYP[26] functional unless otherwise stated. Energy values are given in atomic units (a.u.). *A*(X) values are literature values for the experimentally verified equilibrium constant between equatorial and axial conformations of a substituted cyclohexane.

| **MO of II** | **MO visualization** | **Electron density contour map**<br>(0.15 Å above plane) |
|---|---|---|
| uB3LYP/6-311G+(d)<br>**SOMO** (orbital 22)<br>Energy = -0.11812 | 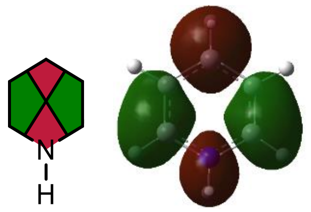 | 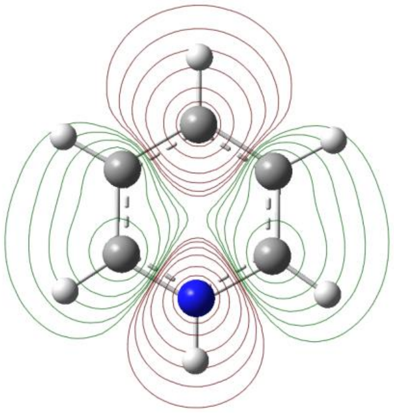 |
| uB3LYP/6-311G+(d)<br>**SOMO–1** (orbital 21)<br>Energy = -0.27560 | 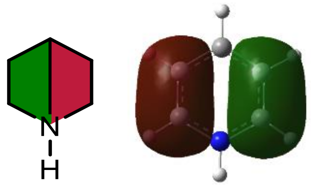 | 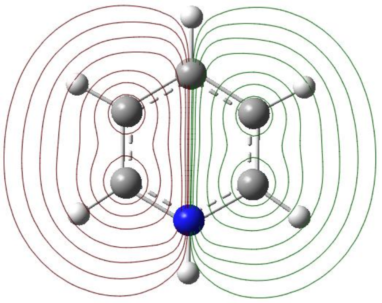 |

**Table S1.** Calculated molecular orbitals of the pyridinyl radical II.

### G.1 NBO analysis

A full NBO analysis was performed on the pyridinyl radical **II**. Using this approach, the wavefunction is analysed in terms of localised orbitals. Since the system is open-shell with doublet multiplicity, the α and β spins are treated separately. Both α and β orbitals indicate localised density about the C4 atom in the model pyridinyl radical, with minimal denisty located at the C2 position.

| Substrate | NBO analysis of the SOMO | **Electron density contour map** (positive values only, 0.06 Å above plane) |
|---|---|---|
| **1a**<br>NBO 22α occupancy = 0.94357<br>Energy = -0.14039 | 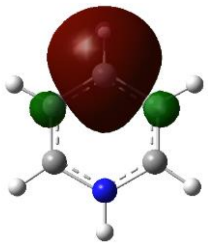 | 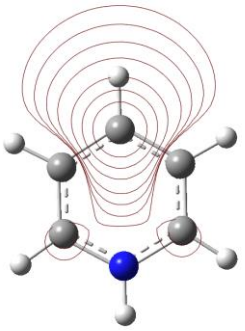 |
| **1a**<br>NBO 22β occupancy = 0.35099<br>Energy = -0.05085 | 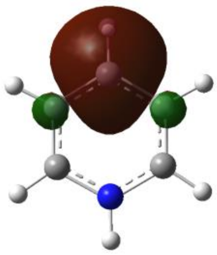 | 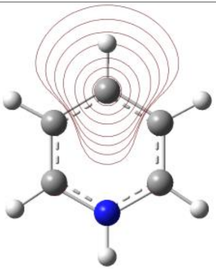 |

**Table S2.** NBO analysis of the pyridinyl radical I.

## G.2 Spin density calculations

| Substrate | Mulliken spin densities | Contour map of spin density (positive values only, 0.15 Å above plane) | Total Energy / a.u. | Dipole moment/ debye |
|---|---|---|---|---|
| **3a** 5.6 : 1 rr C4/C2 | 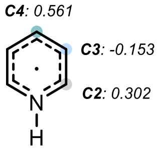  | 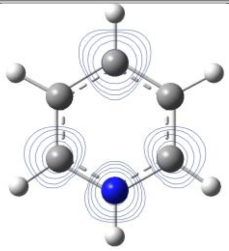 | -248.84962 | 2.0395 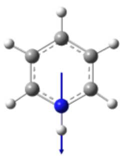 |
| **3a** 5.6 : 1 rr C4/C2 | 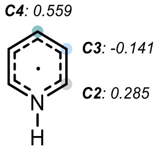  SCI-PCM(acetone) solvation model | 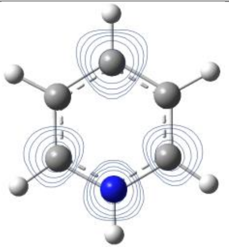 | -248.85550 | 2.7639 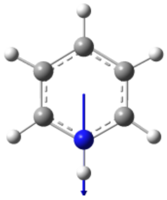 |
| **3a** 5.6 : 1 rr C4(major)/ C2(minor) | 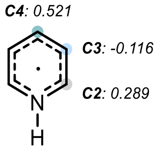  uB3LYP/6-311G+(d) | 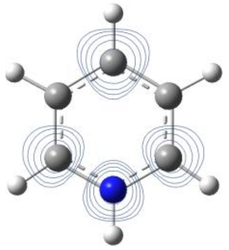 | -248.89864 | 2.0144 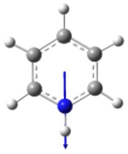 |
| **3a** 5.6 : 1 rr C4(major)/ C2(minor) | 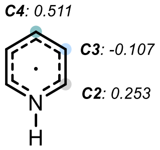  uB3LYP/cc-pVDZ[27] | 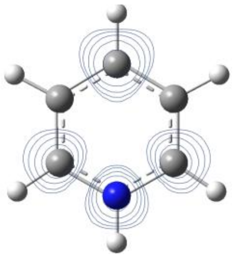 | -248.85512 | 2.0490 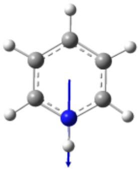 |
| **3a** 5.6 : 1 rr C4(major)/ C2(minor) | 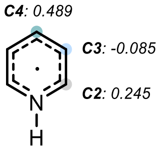  uB3LYP/cc-pVTZ [27] | 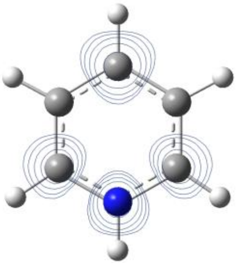 | -248.93286 | 1.9879 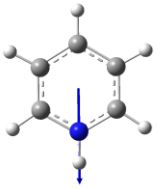 |
| **3d** 5 : 1 r.r. C4(major)/ C2(minor) | 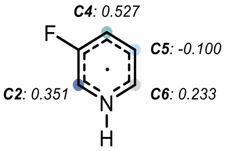  steric factor: *A*(F) = 0.15 | 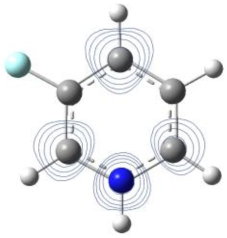 | -348.08990 | 3.2951 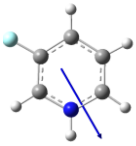 |

| | | | | |
|---|---|---|---|---|
| **3e**<br>8:1 r.r.<br>C4(major)/<br>C2(minor) | 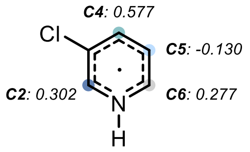<br>steric factor: *A*(Cl) = 0.43 | 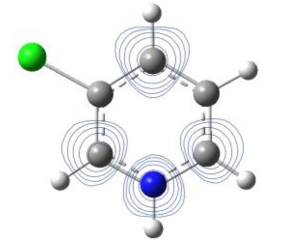 | -708.44483 | 3.4003<br>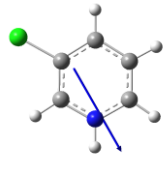 |
| **3f**<br>9:1 r.r.<br>C4(major)/<br>C6(minor) | 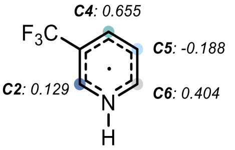<br>steric factor: *A*($CF_3$) = 2.1 | 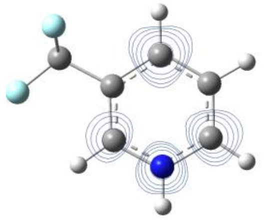 | -585.91210 | 4.5760<br>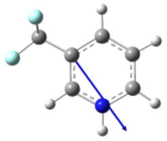 |
| **3b**<br>C4 only | 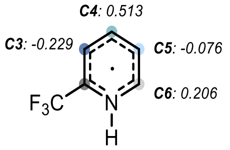<br>steric factor: *A*($CF_3$) = 2.1 | 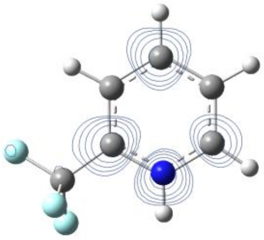 | -585.91327 | 2.8984<br>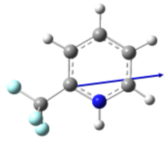 |
| **3g**<br>14 : 1<br>C4(major)/<br>C6(minor) | 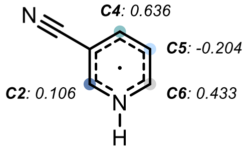<br>steric factor: *A*(CN) = 0.17 | 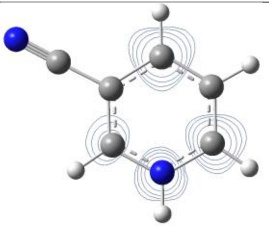 | -341.09787 | 6.2084<br>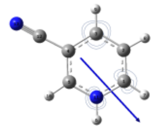 |
| **3j**<br>C6 only | 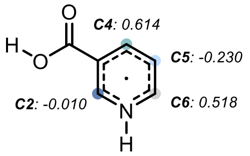<br>steric factor: *A*($CO_2^-$) = 1.92<br>*A*($CO_2H$) = 1.35 | 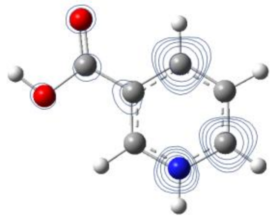 | -437.55155 | 4.8603<br>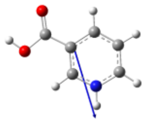 |
| **3l** (Me<br>instead of<br>Et)<br><br>C6 only | 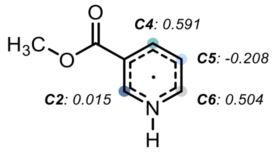<br>steric factor: *A*($CO_2CH_3$) =<br>1.27 | 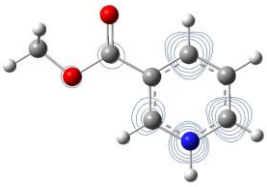 | -476.71603 | 4.3854<br>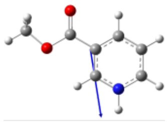 |
| **3o**<br>(Me<br>instead of<br>Et)<br><br>C2 only | 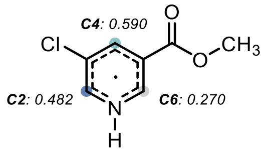<br>steric factor: *A*(Cl) = 0.43<br>*A*($CO_2CH_3$) = 1.27 | 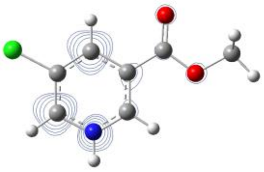 | -936.33518 | 5.5148<br>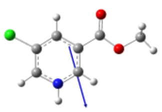 |

| | | | | |
|---|---|---|---|---|
| **3aa**<br>C4/6 only | 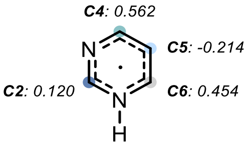 | 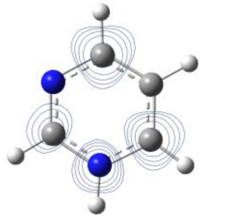 | -264.89105 | 3.5256<br>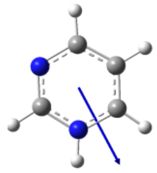 |
| **3cc**<br>C4/5 only | 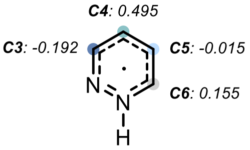 | 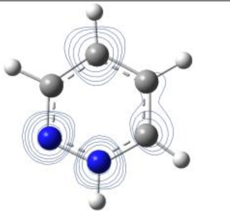 | -264.87491 | 2.1416<br>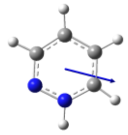 |
| **3x**<br>C1 only | 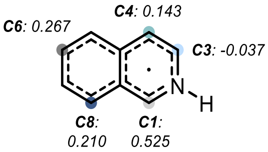 | 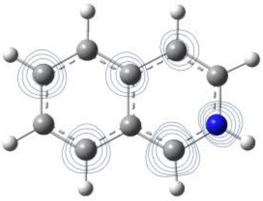 | -402.50603 | 3.1094<br>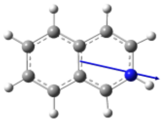 |

**Table S3.** Calculates spin densities on the pyridinyl-type radicals for different classes of substrates.

## G.3 Input and optimized geometries of the computed structures

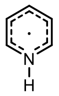

uB3LYP/6-31G+(d)

Input orientation:

| Center Number | Atomic Number | Atomic Type | Coordinates (Angstroms) X | Y | Z |
|---|---|---|---|---|---|
| 1 | 6 | 0 | 1.064257 | 0.100402 | 0.000000 |
| 2 | 6 | 0 | 2.459417 | 0.100402 | 0.000000 |
| 3 | 6 | 0 | 3.156955 | 1.308153 | 0.000000 |
| 4 | 6 | 0 | 2.459301 | 2.516662 | -0.001199 |
| 5 | 6 | 0 | 1.064476 | 2.516584 | -0.001678 |
| 6 | 1 | 0 | 0.514498 | -0.851915 | 0.000450 |
| 7 | 1 | 0 | 3.008925 | -0.852111 | 0.001315 |
| 8 | 1 | 0 | 4.256635 | 1.308233 | 0.000634 |
| 9 | 1 | 0 | 3.009501 | 3.468805 | -0.001258 |
| 10 | 1 | 0 | 0.514354 | 3.468865 | -0.002631 |
| 11 | 1 | 0 | -0.732729 | 1.308561 | -0.000862 |
| 12 | 7 | 0 | 0.366875 | 1.308378 | -0.000682 |

Output orientation:

| Center Number | Atomic Number | Atomic Type | Coordinates (Angstroms) X | Y | Z |
|---|---|---|---|---|---|
| 1 | 6 | 0 | 1.080128 | 0.093151 | 0.000076 |
| 2 | 6 | 0 | 2.447733 | 0.090813 | 0.000506 |
| 3 | 6 | 0 | 3.186109 | 1.308319 | 0.000030 |
| 4 | 6 | 0 | 2.447831 | 2.525981 | -0.001049 |
| 5 | 6 | 0 | 1.080261 | 2.523744 | -0.001481 |
| 6 | 1 | 0 | 0.472873 | -0.804016 | 0.000380 |
| 7 | 1 | 0 | 2.961049 | -0.866796 | 0.001215 |
| 8 | 1 | 0 | 4.269771 | 1.308304 | 0.000361 |
| 9 | 1 | 0 | 2.961289 | 3.483508 | -0.001562 |
| 10 | 1 | 0 | 0.473068 | 3.420958 | -0.002326 |
| 11 | 1 | 0 | -0.622646 | 1.308558 | -0.001276 |
| 12 | 7 | 0 | 0.385000 | 1.308491 | -0.000783 |

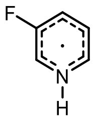

uB3LYP/6-31G+(d)

Input orientation:

| Center Number | Atomic Number | Atomic Type | Coordinates (Angstroms) X | Y | Z |
|---|---|---|---|---|---|
| 1 | 6 | 0 | -0.053548 | 0.381526 | 0.000000 |
| 2 | 6 | 0 | 1.341612 | 0.381526 | 0.000000 |
| 3 | 6 | 0 | 2.039150 | 1.589277 | 0.000000 |
| 4 | 6 | 0 | 1.341496 | 2.797786 | -0.001199 |
| 5 | 6 | 0 | -0.053329 | 2.797708 | -0.001678 |
| 6 | 1 | 0 | -0.603307 | -0.570791 | 0.000450 |
| 7 | 1 | 0 | 3.138830 | 1.589357 | 0.000634 |
| 8 | 1 | 0 | 1.891696 | 3.749929 | -0.001258 |
| 9 | 1 | 0 | -0.603451 | 3.749989 | -0.002631 |
| 10 | 1 | 0 | -1.850534 | 1.589685 | -0.000862 |
| 11 | 7 | 0 | -0.750930 | 1.589502 | -0.000682 |
| 12 | 9 | 0 | 2.016220 | -0.787834 | 0.001614 |

Output orientation:

| Center Number | Atomic Number | Atomic Type | Coordinates (Angstroms) X | Y | Z |
|---|---|---|---|---|---|
| 1 | 6 | 0 | -0.283536 | -1.186974 | 0.000014 |
| 2 | 6 | 0 | -0.947190 | 0.009590 | -0.000006 |
| 3 | 6 | 0 | -0.306335 | 1.260773 | -0.000037 |
| 4 | 6 | 0 | 1.122714 | 1.232024 | 0.000017 |
| 5 | 6 | 0 | 1.809714 | 0.051880 | 0.000028 |
| 6 | 1 | 0 | -0.777228 | -2.148581 | 0.000058 |
| 7 | 1 | 0 | -0.876751 | 2.180463 | -0.000050 |
| 8 | 1 | 0 | 1.684899 | 2.160694 | 0.000061 |
| 9 | 1 | 0 | 2.890693 | -0.015836 | 0.000072 |
| 10 | 1 | 0 | 1.629579 | -2.029428 | 0.000138 |
| 11 | 7 | 0 | 1.120467 | -1.159622 | -0.000078 |
| 12 | 9 | 0 | -2.307406 | -0.025968 | 0.000020 |

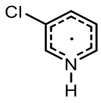

uB3LYP/6-31G+(d)

Input orientation:

| Center Number | Atomic Number | Atomic Type | Coordinates (Angstroms) X | Y | Z |
|---|---|---|---|---|---|
| 1 | 6 | 0 | 2.171732 | -0.537123 | 0.000000 |
| 2 | 6 | 0 | 2.869270 | 0.670628 | 0.000000 |
| 3 | 6 | 0 | 2.171616 | 1.879137 | -0.001199 |
| 4 | 6 | 0 | 0.776791 | 1.879059 | -0.001678 |
| 5 | 6 | 0 | 0.079190 | 0.670853 | -0.000682 |
| 6 | 1 | 0 | 0.226813 | -1.489440 | 0.000450 |
| 7 | 1 | 0 | 2.721240 | -1.489636 | 0.001315 |
| 8 | 1 | 0 | 2.721816 | 2.831280 | -0.001258 |
| 9 | 1 | 0 | 0.226669 | 2.831340 | -0.002631 |
| 10 | 1 | 0 | -1.020414 | 0.671036 | -0.000862 |
| 11 | 7 | 0 | 0.776572 | -0.537123 | 0.000000 |
| 12 | 17 | 0 | 4.629270 | 0.670756 | 0.001015 |

Output orientation:

| Center Number | Atomic Number | Atomic Type | Coordinates (Angstroms) X | Y | Z |
|---|---|---|---|---|---|
| 1 | 6 | 0 | 0.137087 | -1.175938 | 0.000009 |
| 2 | 6 | 0 | -0.529869 | 0.018652 | 0.000005 |
| 3 | 6 | 0 | 0.142674 | 1.266263 | 0.000015 |
| 4 | 6 | 0 | 1.568040 | 1.222683 | 0.000002 |
| 5 | 6 | 0 | 2.245256 | 0.036851 | -0.000008 |
| 6 | 1 | 0 | 2.034359 | -2.044033 | 0.000145 |
| 7 | 1 | 0 | -0.351691 | -2.140787 | -0.000044 |
| 8 | 1 | 0 | -0.405517 | 2.199149 | -0.000037 |
| 9 | 1 | 0 | 2.138570 | 2.146636 | 0.000002 |
| 10 | 1 | 0 | 3.325175 | -0.044337 | -0.000006 |
| 11 | 7 | 0 | 1.537166 | -1.167340 | -0.000026 |
| 12 | 17 | 0 | -2.287070 | -0.009194 | -0.000001 |

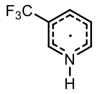


uB3LYP/6-31G+(d)

Input orientation:

| Center Number | Atomic Number | Atomic Type | X | Y | Z |
|---|---|---|---|---|---|
| 1 | 6 | 0 | -1.572959 | 1.077644 | 0.000000 |
| 2 | 6 | 0 | -0.177799 | 1.077644 | 0.000000 |
| 3 | 6 | 0 | 0.519739 | 2.285395 | 0.000000 |
| 4 | 6 | 0 | -1.572740 | 3.493826 | -0.001678 |
| 5 | 6 | 0 | -2.270341 | 2.285620 | -0.000682 |
| 6 | 1 | 0 | -2.122718 | 0.125327 | 0.000450 |
| 7 | 1 | 0 | 0.371709 | 0.125131 | 0.001315 |
| 8 | 1 | 0 | 1.619419 | 2.285475 | 0.000634 |
| 9 | 1 | 0 | 0.372285 | 4.446047 | -0.001258 |
| 10 | 1 | 0 | -2.122862 | 4.446107 | -0.002631 |
| 11 | 6 | 0 | -3.810341 | 2.285876 | -0.000934 |
| 12 | 7 | 0 | -0.177915 | 3.493904 | -0.001199 |
| 13 | 9 | 0 | -4.260264 | 3.381702 | 0.646550 |
| 14 | 9 | 0 | -4.260130 | 2.298877 | -1.273734 |
| 15 | 9 | 0 | -4.260628 | 1.177275 | 0.624162 |

Coordinates (Angstroms)

Output orientation:

| Center Number | Atomic Number | Atomic Type | X | Y | Z |
|---|---|---|---|---|---|
| 1 | 6 | 0 | -0.717641 | -1.265327 | 0.000203 |
| 2 | 6 | 0 | -2.130730 | -1.225039 | -0.000111 |
| 3 | 6 | 0 | -2.814249 | -0.036887 | -0.000204 |
| 4 | 6 | 0 | -0.709081 | 1.172423 | -0.000082 |
| 5 | 6 | 0 | -0.023423 | -0.006431 | 0.000041 |
| 6 | 1 | 0 | -0.174242 | -2.201587 | 0.000264 |
| 7 | 1 | 0 | -2.701866 | -2.148821 | -0.000323 |
| 8 | 1 | 0 | -3.892869 | 0.050245 | -0.000062 |
| 9 | 1 | 0 | -2.587776 | 2.049329 | -0.003265 |
| 10 | 1 | 0 | -0.227267 | 2.141317 | -0.000294 |
| 11 | 6 | 0 | 1.473882 | 0.008186 | 0.000038 |
| 12 | 7 | 0 | -2.093052 | 1.170782 | 0.000850 |
| 13 | 9 | 0 | 1.995157 | 1.262524 | -0.000567 |
| 14 | 9 | 0 | 1.989316 | -0.628937 | 1.086967 |
| 15 | 9 | 0 | 1.989176 | -0.629976 | -1.086575 |

Coordinates (Angstroms)

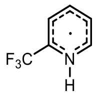


uB3LYP/6-31G+(d)

Input orientation:

| Center Number | Atomic Number | Atomic Type | X | Y | Z |
|---|---|---|---|---|---|
| 1 | 6 | 0 | -3.291775 | -0.925402 | 0.000000 |
| 2 | 6 | 0 | -1.884897 | -0.925402 | 0.000000 |
| 3 | 6 | 0 | -1.218206 | 0.301205 | 0.000000 |
| 4 | 6 | 0 | -1.972279 | 1.475992 | -0.000241 |
| 5 | 6 | 0 | -3.375481 | 1.373187 | -0.000382 |
| 6 | 1 | 0 | -0.119662 | 0.340981 | -0.000950 |
| 7 | 1 | 0 | -1.330165 | -1.872303 | 0.000264 |
| 8 | 1 | 0 | -1.488243 | 2.460916 | -0.000342 |
| 9 | 1 | 0 | -4.008830 | 2.278250 | -0.000463 |
| 10 | 6 | 0 | -4.080255 | -2.248240 | 0.000170 |
| 11 | 9 | 0 | -3.761881 | -2.961740 | 1.101104 |
| 12 | 9 | 0 | -5.403963 | -1.983115 | 0.002987 |
| 13 | 9 | 0 | -3.766123 | -2.959494 | - 1.103432 |
| 14 | 1 | 0 | -5.104747 | 0.221998 | -0.000239 |
| 15 | 7 | 0 | -4.035011 | 0.198236 | -0.000197 |

Coordinates (Angstroms)

Standard orientation:

| Center Number | Atomic Number | Atomic Type | X | Y | Z |
|---|---|---|---|---|---|
| 1 | 6 | 0 | 0.033837 | 0.093336 | -0.041109 |
| 2 | 6 | 0 | 0.770766 | 1.261582 | -0.025599 |
| 3 | 6 | 0 | 2.171473 | 1.230159 | 0.006485 |
| 4 | 6 | 0 | 2.820733 | -0.047183 | 0.015425 |
| 5 | 6 | 0 | 2.085975 | -1.194840 | 0.001467 |
| 6 | 1 | 0 | 2.747272 | 2.148145 | 0.007951 |
| 7 | 1 | 0 | 0.238324 | 2.206782 | -0.040334 |
| 8 | 1 | 0 | 3.903587 | -0.118800 | 0.030185 |
| 9 | 1 | 0 | 2.520898 | -2.187467 | 0.001618 |
| 10 | 6 | 0 | -1.446482 | 0.033845 | -0.000841 |
| 11 | 9 | 0 | -2.020090 | 1.223760 | -0.273400 |
| 12 | 9 | 0 | -1.938659 | -0.879232 | -0.900888 |
| 13 | 9 | 0 | -1.938534 | -0.376430 | 1.211105 |
| 14 | 1 | 0 | 0.174978 | -1.984492 | -0.206886 |
| 15 | 7 | 0 | 0.696095 | -1.148350 | 0.020162 |

Coordinates (Angstroms)

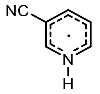


uB3LYP/6-31G+(d)

Input orientation:

| Center Number | Atomic Number | Atomic Type | X | Y | Z |
|---|---|---|---|---|---|
| 1 | 6 | 0 | 1.328226 | 0.368139 | 0.000000 |
| 2 | 6 | 0 | 2.025764 | 1.575890 | 0.000000 |
| 3 | 6 | 0 | 1.328110 | 2.784399 | -0.001199 |
| 4 | 6 | 0 | -0.066715 | 2.784321 | -0.001678 |
| 5 | 6 | 0 | -0.764316 | 1.576115 | -0.000682 |
| 6 | 1 | 0 | -0.616693 | -0.584178 | 0.000450 |
| 7 | 1 | 0 | 1.877734 | -0.584374 | 0.001315 |
| 8 | 1 | 0 | 3.125444 | 1.575970 | 0.000634 |
| 9 | 1 | 0 | 1.878310 | 3.736542 | -0.001258 |
| 10 | 1 | 0 | -1.863920 | 1.576298 | -0.000862 |
| 11 | 7 | 0 | -0.066934 | 0.368139 | 0.000000 |
| 12 | 6 | 0 | -0.837054 | 4.117804 | -0.003012 |
| 13 | 7 | 0 | -1.410605 | 5.110643 | -0.004006 |

Coordinates (Angstroms)

Output orientation:

| Center Number | Atomic Number | Atomic Type | X | Y | Z |
|---|---|---|---|---|---|
| 1 | 6 | 0 | -2.160826 | 0.018270 | -0.000003 |
| 2 | 6 | 0 | -1.490305 | 1.216739 | 0.000005 |
| 3 | 6 | 0 | -0.082895 | 1.280550 | -0.000005 |
| 4 | 6 | 0 | 0.639720 | 0.024941 | 0.000000 |
| 5 | 6 | 0 | -0.043011 | -1.164778 | 0.000006 |
| 6 | 1 | 0 | -1.900503 | -2.065995 | 0.000092 |
| 7 | 1 | 0 | -3.237246 | -0.087854 | 0.000019 |
| 8 | 1 | 0 | -2.074038 | 2.132775 | 0.000013 |
| 9 | 1 | 0 | 0.451855 | 2.221246 | 0.000009 |
| 10 | 1 | 0 | 0.454751 | -2.127197 | 0.000014 |
| 11 | 7 | 0 | -1.417309 | -1.180287 | -0.000026 |
| 12 | 6 | 0 | 2.068614 | -0.005003 | 0.000000 |
| 13 | 7 | 0 | 3.234081 | -0.005040 | 0.000001 |

Coordinates (Angstroms)

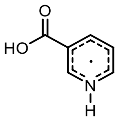

uB3LYP/6-31G+(d)

Input orientation:

| Center | Atomic | Atomic | Cordinates (Angstroms) | | |
|---|---|---|---|---|---|
| Number | Number | Type | X | Y | Z |
| 1 | 6 | 0 | -1.957447 | 0.687943 | 0.000000 |
| 2 | 6 | 0 | -0.562287 | 0.687943 | 0.000000 |
| 3 | 6 | 0 | 0.135251 | 1.895694 | 0.000000 |
| 4 | 6 | 0 | -0.562403 | 3.104203 | -0.001199 |
| 5 | 6 | 0 | -2.654829 | 1.895919 | -0.000682 |
| 6 | 1 | 0 | -2.507206 | -0.264374 | 0.000450 |
| 7 | 1 | 0 | -0.012779 | -0.264570 | 0.001315 |
| 8 | 1 | 0 | -0.012203 | 4.056346 | -0.001258 |
| 9 | 1 | 0 | -2.507350 | 4.056406 | -0.002631 |
| 10 | 1 | 0 | -3.754433 | 1.896102 | -0.000862 |
| 11 | 6 | 0 | 1.675251 | 1.895806 | 0.000888 |
| 12 | 8 | 0 | 2.323586 | 3.024099 | 0.000886 |
| 13 | 8 | 0 | 2.323750 | 0.767608 | 0.001637 |
| 14 | 1 | 0 | 3.268006 | 0.940753 | 0.001975 |
| 15 | 7 | 0 | -1.957228 | 3.104125 | -0.001678 |

Output orientation:

| Center | Atomic | Atomic | Coordinates (Angstroms) | | |
|---|---|---|---|---|---|
| Number | Number | Type | X | Y | Z |
| 1 | 6 | 0 | -1.846517 | 1.235758 | -0.000009 |
| 2 | 6 | 0 | -0.451769 | 1.268688 | -0.000015 |
| 3 | 6 | 0 | 0.258534 | 0.002882 | -0.000003 |
| 4 | 6 | 0 | -0.457573 | -1.162464 | 0.000011 |
| 5 | 6 | 0 | -2.547568 | 0.049417 | 0.000003 |
| 6 | 1 | 0 | -2.411275 | 2.160866 | -0.000014 |
| 7 | 1 | 0 | 0.094889 | 2.198772 | -0.000023 |
| 8 | 1 | 0 | 0.036220 | -2.125354 | 0.000020 |
| 9 | 1 | 0 | -2.310211 | -2.033397 | 0.000023 |
| 10 | 1 | 0 | -3.622328 | -0.038086 | 0.000002 |
| 11 | 6 | 0 | 1.720499 | -0.122840 | -0.000005 |
| 12 | 8 | 0 | 2.351904 | -1.163205 | -0.000025 |
| 13 | 8 | 0 | 2.345118 | 1.087968 | 0.000027 |
| 14 | 1 | 0 | 3.295374 | 0.903602 | 0.000025 |
| 15 | 7 | 0 | -1.816069 | -1.156164 | 0.000008 |

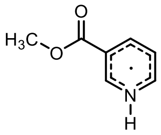

uB3LYP/6-31G+(d)

Input orientation:

| Center | Atomic | Atomic | Coordinates (Angstroms) | | |
|---|---|---|---|---|---|
| Number | Number | Type | X | Y | Z |
| 1 | 6 | 0 | -1.957447 | 0.687943 | 0.000000 |
| 2 | 6 | 0 | -0.562287 | 0.687943 | 0.000000 |
| 3 | 6 | 0 | 0.135251 | 1.895694 | 0.000000 |
| 4 | 6 | 0 | -0.562403 | 3.104203 | -0.001199 |
| 5 | 6 | 0 | -2.654829 | 1.895919 | -0.000682 |
| 6 | 1 | 0 | -2.507206 | -0.264374 | 0.000450 |
| 7 | 1 | 0 | -0.012779 | -0.264570 | 0.001315 |
| 8 | 1 | 0 | -0.012203 | 4.056346 | -0.001258 |
| 9 | 1 | 0 | -2.507350 | 4.056406 | -0.002631 |
| 10 | 1 | 0 | -3.754433 | 1.896102 | -0.000862 |
| 11 | 6 | 0 | 1.675251 | 1.895806 | 0.000888 |
| 12 | 8 | 0 | 2.323586 | 3.024099 | 0.000886 |
| 13 | 8 | 0 | 2.323750 | 0.767608 | 0.001637 |
| 14 | 7 | 0 | -1.957228 | 3.104125 | -0.001678 |
| 15 | 6 | 0 | 3.730299 | 1.025522 | 0.002140 |
| 16 | 1 | 0 | 3.989774 | 1.586288 | 0.875704 |
| 17 | 1 | 0 | 4.263063 | 0.097588 | 0.002689 |
| 18 | 1 | 0 | 3.990511 | 1.585675 | -0.871598 |

Output orientation:

| Center | Atomic | Atomic | Coordinates (Angstroms) | | |
|---|---|---|---|---|---|
| Number | Number | Type | X | Y | Z |
| 1 | 6 | 0 | -2.138094 | -1.380207 | -0.000002 |
| 2 | 6 | 0 | -0.744762 | -1.250449 | -0.000007 |
| 3 | 6 | 0 | -0.185310 | 0.091579 | -0.000004 |
| 4 | 6 | 0 | -1.034638 | 1.167651 | 0.000000 |
| 5 | 6 | 0 | -2.974550 | -0.280520 | 0.000005 |
| 6 | 1 | 0 | -2.592131 | -2.367549 | -0.000002 |
| 7 | 1 | 0 | -0.092714 | -2.113141 | -0.000010 |
| 8 | 1 | 0 | -0.659430 | 2.184914 | 0.000003 |
| 9 | 1 | 0 | -2.981746 | 1.818899 | 0.000009 |
| 10 | 1 | 0 | -4.055100 | -0.316860 | 0.000009 |
| 11 | 6 | 0 | 1.256636 | 0.389958 | -0.000006 |
| 12 | 8 | 0 | 1.752436 | 1.509939 | -0.000006 |
| 13 | 8 | 0 | 2.008487 | -0.741379 | 0.000003 |
| 14 | 7 | 0 | -2.387534 | 1.002855 | 0.000005 |
| 15 | 6 | 0 | 3.431092 | -0.545118 | 0.000007 |
| 16 | 1 | 0 | 3.741627 | 0.007801 | 0.891015 |
| 17 | 1 | 0 | 3.860972 | -1.547706 | 0.000008 |
| 18 | 1 | 0 | 3.741632 | 0.007801 | -0.890998 |

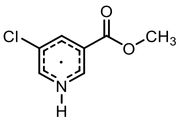

uB3LYP/6-31G+(d)

Input orientation:

| Center | Atomic | Atomic | Coordinates | | |
|---|---|---|---|---|---|
| Number | Number | Type | X | Y | Z |
| 1 | 6 | 0 | -1.770117 | 0.175950 | 0.000000 |
| 2 | 6 | 0 | -0.374957 | 0.175950 | 0.000000 |
| 3 | 6 | 0 | 0.322581 | 1.383701 | 0.000000 |
| 4 | 6 | 0 | -0.375073 | 2.592210 | -0.001199 |
| 5 | 6 | 0 | -1.769898 | 2.592132 | -0.001678 |
| 6 | 1 | 0 | -2.319876 | -0.776367 | 0.000450 |
| 7 | 1 | 0 | 1.422261 | 1.383781 | 0.000634 |
| 8 | 1 | 0 | -2.320020 | 3.544413 | -0.002631 |
| 9 | 1 | 0 | -3.567103 | 1.384109 | -0.000862 |
| 10 | 7 | 0 | -2.467499 | 1.383926 | -0.000682 |
| 11 | 6 | 0 | 0.395431 | 3.925598 | -0.001282 |
| 12 | 8 | 0 | -0.023430 | 5.082368 | -0.002229 |
| 13 | 8 | 0 | 1.740095 | 3.743149 | -0.000171 |
| 14 | 6 | 0 | 2.418807 | 5.001819 | -0.000296 |
| 15 | 1 | 0 | 2.143449 | 5.555602 | 0.872861 |
| 16 | 1 | 0 | 3.476028 | 4.836949 | 0.000599 |
| 17 | 1 | 0 | 2.144790 | 5.554706 | -0.874441 |
| 18 | 17 | 0 | 0.504531 | -1.348548 | 0.002105 |

Standard orientation:

| Center | Atomic | Atomic | Coordinates (Angstroms) | | |
|---|---|---|---|---|---|
| Number | Number | Type | X | Y | Z |
| 1 | 6 | 0 | 2.204339 | 1.126388 | -0.000011 |
| 2 | 6 | 0 | 1.794309 | -0.196259 | -0.000013 |
| 3 | 6 | 0 | 0.457391 | -0.581541 | -0.000003 |
| 4 | 6 | 0 | -0.539362 | 0.481035 | -0.000007 |
| 5 | 6 | 0 | -0.117231 | 1.791214 | -0.000028 |
| 6 | 1 | 0 | 3.228673 | 1.464149 | -0.000004 |
| 7 | 1 | 0 | 0.164566 | -1.621059 | 0.000012 |
| 8 | 1 | 0 | -0.817758 | 2.616874 | -0.000025 |
| 9 | 1 | 0 | 1.469753 | 3.079556 | -0.000012 |
| 10 | 7 | 0 | 1.197652 | 2.105876 | -0.000018 |
| 11 | 6 | 0 | -1.984003 | 0.236485 | 0.000010 |
| 12 | 8 | 0 | -2.856220 | 1.106130 | 0.000074 |
| 13 | 8 | 0 | -2.276439 | -1.086570 | -0.000036 |
| 14 | 6 | 0 | -3.674974 | -1.435722 | -0.000019 |
| 15 | 1 | 0 | -4.166777 | -1.041903 | -0.892301 |
| 16 | 1 | 0 | -3.700611 | -2.524649 | -0.000064 |
| 17 | 1 | 0 | -4.166735 | -1.041979 | 0.892320 |
| 18 | 17 | 0 | 3.048459 | -1.432836 | 0.000019 |

**Table S4.** Input and optimized geometries for different classes of pyridines used in this study.

## H. References


(1) Tanabe, S.; Mitsunuma, H.; Kanai, M. Catalytic Allylation of Aldehydes Using Unactivated Alkenes. *J. Am. Chem. Soc.* **2020**, *142*, 12374–12381.

(2) Le Saux, E.; Zanini, M.; Melchiorre, P. Photochemical Organocatalytic Benzylation of Allylic C-H Bonds. *J. Am. Chem. Soc*. **2022**, *144*, 1113–1118.

(3) Shapiro, N. D.; Rauniyar, V.; Hamilton, G. L.; Wu, J.; Toste, F. D. Asymmetric Additions to Dienes Catalyzed by a Dithiophosphoric Acid. *Nature* **2011**, *470*, 245–249.

(4) Abe, T.; Mino, T.; Watanabe, K.; Sakamoto, M. Suzuki–Miyaura Coupling of Aryl Chlorides with Arylboronic Acids Using the Morpholine–NiCl2 Catalyst System. *Eur. J. Org. Chem*. **2014**, *31*, 6983–6991.

(5) Deguest, G.; Devineau, A.; Bischoff, L.; Fruit, C.; Marsais, F. One-Pot Synthesis of 2,3-Dihydro-pyrrolopyridinones Using in Situ Generated Formimines. *Org. Lett*. **2006**, *25*, 5889–5892.

(6) Azizi, M. S.; Edder, Y.; Karim, A.; Sauthier, M. Nickel(0)-Catalyzed N-Allylation of Amides and p-Toluenesulfonamide with Allylic Alcohols under Neat and Neutral Conditions. *Eur. J. Org. Chem*. **2016**, *22*, 3796–3803.

(7) Saito, R.; Naruse, S.; Takano, K.; Fukuda, K.; Katoh, A.; Inoue, Y. Unusual Temperature Dependence of Enantioselectivity in Asymmetric Reductions by Chiral NADH Models. *Org. Lett*. **2006**, 10, 2067–2070.

(8) Zhang, T.; Luan, Y-X.; Lam, N. Y. S.; Li, J-F.; Li, Y.; Ye, M.; Yu, J-Q. A Directive Ni Catalyst Overrides Conventional Site Selectivity in Pyridine C–H Alkenylation. *Nat. Chem.* **2021**, *13*, 1207–1213.

(9) Yamaguchi, T.; Matsumura, Y.; Ishii, T.; Tokuoka, Y.; Kurita, K. Synthesis of Nicotinamide Derivatives Having a Hydroxy Substituted Benzene Ring and the Influence of Their Structures on the Apoptosis-Inducing Activity Against Leukemia Cells. *Drug Dev. Res*. **2011**, *72*, 289–297.

(10) Lu, J.; Yin, L.; Liu, T.; Wang, Y. Synthesis of Pseudopeptides Based L-Tryptophan as a Potential Antimicrobial Agent. *Bioorg. Med. Chem. Lett.* **2007**, *17*, 1601–1607.

(11) Fontesa, A.; Prata, M. I. M.; Geraldes, C. F. G. C.; André, J. P. Ga(III) Chelates of Amphiphilic DOTA-Based Ligands: Synthetic Route and *in vitro* and *in vivo* Studies. *Nuclear Medicine and Biology*, **2011**, *38*, 363–370.

(12) Gray, A. P.; Kraus, H.; Heitmeier, D. E.; Shiley, R. H. Cyclopropylpyridines. Interaction with Acid and Hydrogen. The Synthesis of Cyclopropane Ring-Opened Analogs. *J. Org. Chem*. **1968**, *33*, 3007–3015.

(13) Deetz, M. J.; Forbes, C. C.; Jonas, M.; Malerich, J. P.; Smith, B. D.; Wiest, O. Unusually Low Barrier to Carbamate C−N Rotation. *J. Org. Chem*. **2002**, *67*, 3949–3952.

(14) Cuthbertson, J. D.; MacMillan, D. W. C. The Direct Arylation of Allylic $sp^3$ C-H Bonds via Organic and Photoredox Catalysis. *Nature* **2015**, *519*,74−77.

(15) Huang, C-Y.; Li, J. ; Li, C-J. A Cross-Dehydrogenative C($sp^3$)−H Heteroarylation via Photo-Induced Catalytic Chlorine Radical Generation. *Nat. Comm.* **2021**, *12*, 4010.

(16) Beatty, J. W.; Douglas, J. J.; Cole, K. P.; Stephenson, C. R. J. A Scalable and Operationally Simple Radical Trifluoromethylation. *Nat. Comm.* **2015**, *6*, 7919.

(17) Kavarnos, G. J. Energetics of photoinduced electron transfer. In *Fundamentals of Photoinduced Electron Transfer*; VCH: New-York, Weinheim, 1993, pp 29–37.

(18) J. R. Lakowicz, Ed. *Principles of Fluorescence Spectroscopy*, Plenum Press, **1983**, 52–93.

(19) Fessenden, R. P.; Neta, P. ESR Spectra of Radicals Produced by Reduction of Pyridine and Pyrazine. *Chem. Phys. Lett*. **1973**, *18*, 14–17.

(20) Bieszczad, B.; Perego, L. A.; Melchiorre, P. *Angew. Chem. Int. Ed*. **2019**, *58*, 16878–16883.

(21) Roth, H. G.; Romero, N. A.; Nicewicz, D. A. Experimental and Calculated Electrochemical Potentials of Common Organic Molecules for Applications to Single-Electron Redox Chemistry. *Synlett*, **2016**, *27*, 714–723.

(22) Tarantino, K. T.; Liu, P.; Knowles, R. R. Catalytic Ketyl-Olefin Cyclizations Enabled by Proton-Coupled Electron Transfer. *J. Am. Chem. Soc*. **2013**, *135*, 10022−10025.

(23) Zhu, X-Q.; Li, H-R.; Li, Q.; Ai, T.; Lu, J-Y.; Yang, Y.; Cheng, J-P. Determination of the C4-H Bond Dissociation Energies of NADH Models and Their Radical Cations in Acetonitrile. *Chem. Eur. J*. **2003**, *9*, 871−880.

(24) Gaussian 09, Revision A.02, M. J. Frisch, G. W. Trucks, H. B. Schlegel, G. E. Scuseria, M. A. Robb, J. R. Cheeseman, G. Scalmani, V. Barone, G. A. Petersson, H. Nakatsuji, X. Li, M. Caricato, A. Marenich, J. Bloino, B. G. Janesko, R. Gomperts, B. Mennucci, H. P. Hratchian, J. V. Ortiz, A. F. Izmaylov, J. L. Sonnenberg, D. Williams-Young, F. Ding, F. Lipparini, F. Egidi, J. Goings, B. Peng, A. Petrone, T. Henderson, D. Ranasinghe, V. G. Zakrzewski, J. Gao, N. Rega, G. Zheng, W. Liang, M. Hada, M. Ehara, K. Toyota, R. Fukuda, J. Hasegawa, M. Ishida, T. Nakajima, Y. Honda, O. Kitao, H. Nakai, T. Vreven, K. Throssell, J. A. Montgomery, Jr., J. E. Peralta, F. Ogliaro, M. Bearpark, J. J. Heyd, E. Brothers, K. N. Kudin, V. N. Staroverov, T. Keith, R. Kobayashi, J. Normand, K. Raghavachari, A. Rendell, J. C. Burant, S. S. Iyengar, J. Tomasi, M. Cossi, J. M. Millam, M. Klene, C. Adamo, R. Cammi, J. W. Ochterski, R. L. Martin, K. Morokuma, O. Farkas, J. B. Foresman, and D. J. Fox, Gaussian, Inc., Wallingford CT, 2016.

(25) R. Denningtom, T. Keith and J. Millam, GaussView, 2009, version 5; Semichem Inc.: Shawnee Mission, KS, 2009.

(26) (a) A. D. Becke, *J. Chem. Phys*. **1993**, *98*, 13, 72; (b) C. Lee, W. Yang, R. G. Parr, *Phys.Rev.B*. **1988**, *37*, 785; (c) P. C. Hariharan, J. A. Pople, Theor. Chim. Acta. **1973**, *28*, 213; (d) M. M. Francl, W. J. Petro, W. J. Hehre, J. S. Binkley, M. S. Gordon, D. J. DeFrees, J. A. Pople, *J. Chem. Phys*. **1982**, *77*, 3654.

(27) Dunning Jr., T. H. *J. Chem. Phys*. **1989**, *90*, 1007−1023.

# I. NMR spectra

$^1$H NMR (400 MHz, $CDCl_3$) of **4a**

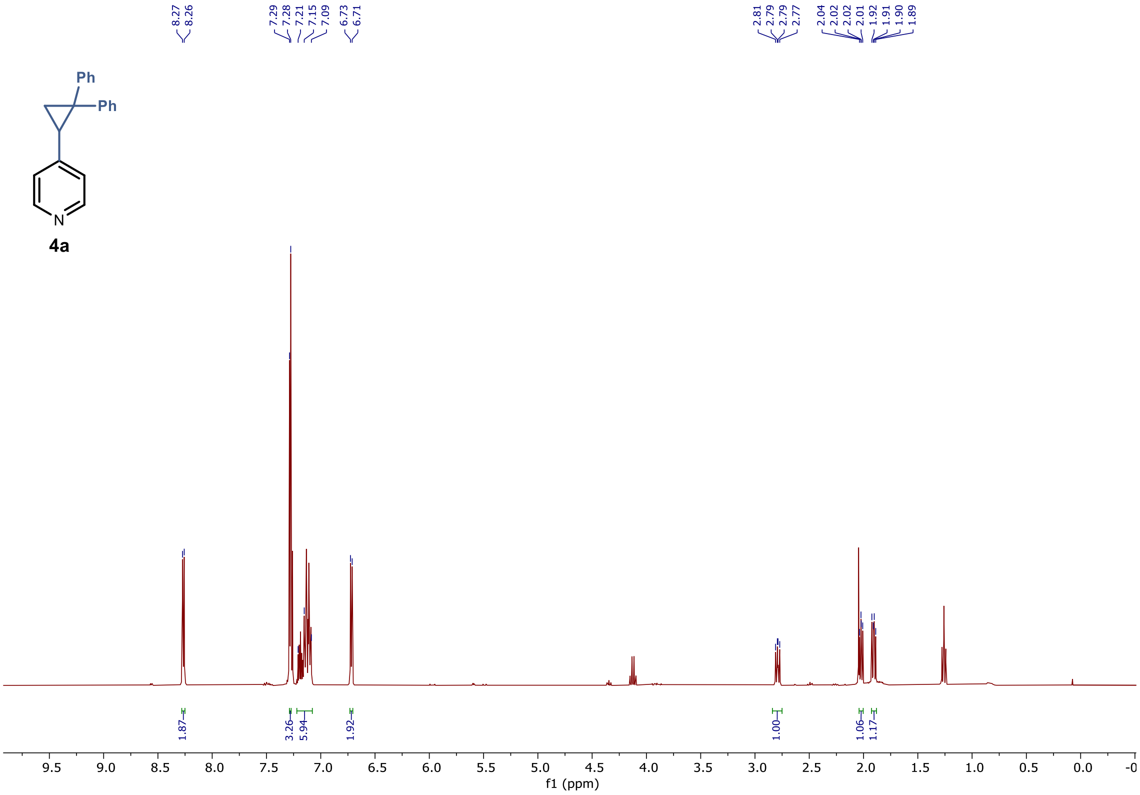


$^{13}$C NMR (101 MHz, $CDCl_3$) of **4a**

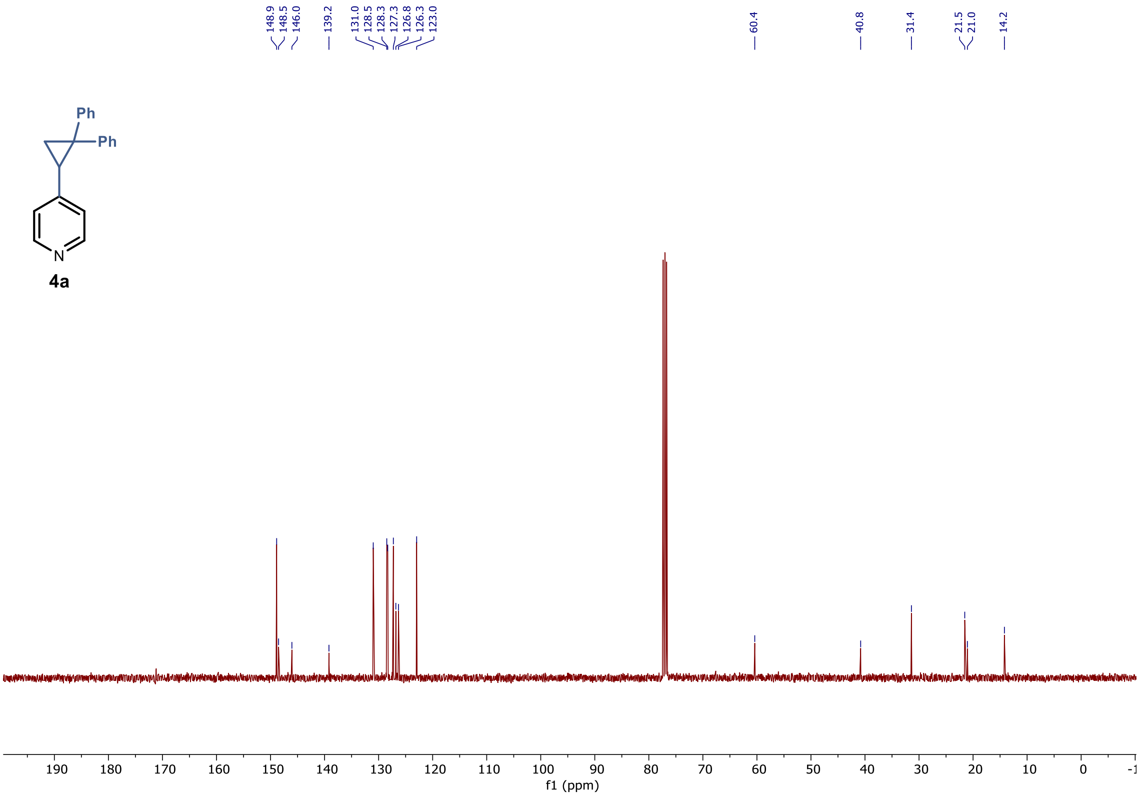

$^{1}H$ NMR (400 MHz, $CDCl_3$) of **4b**

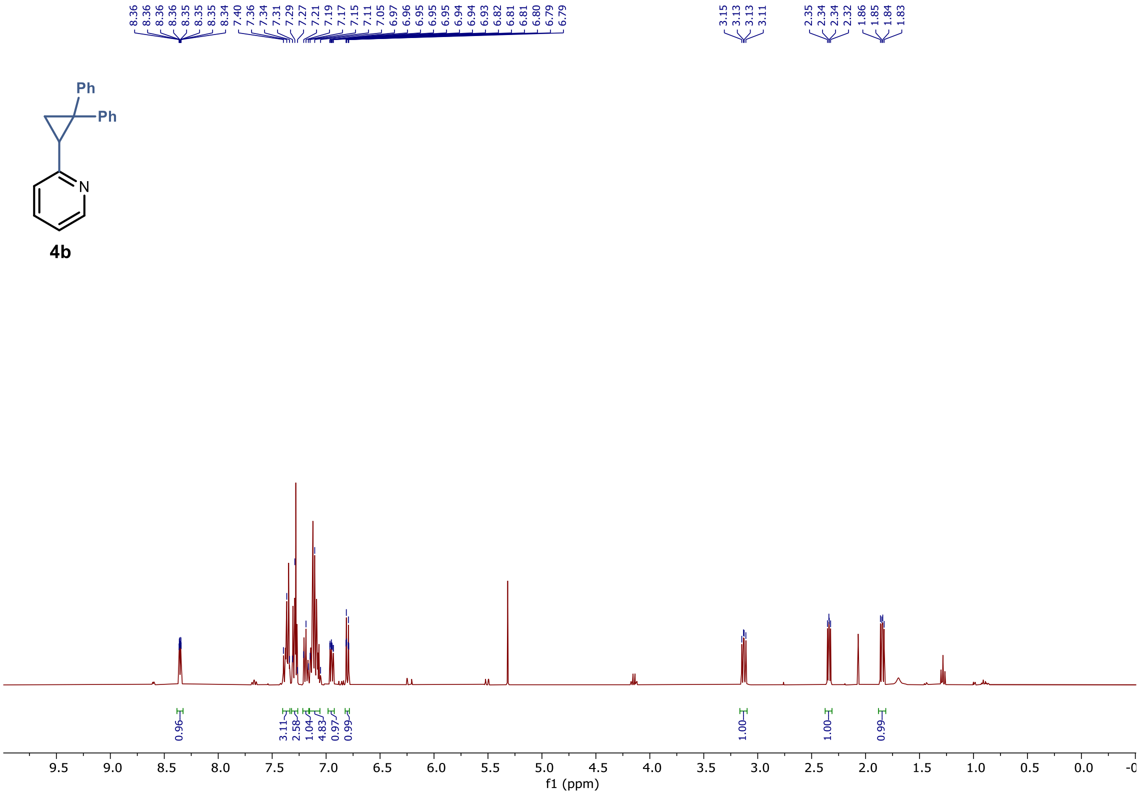


$^{13}C$ NMR (101 MHz, $CDCl_3$) of **4b**

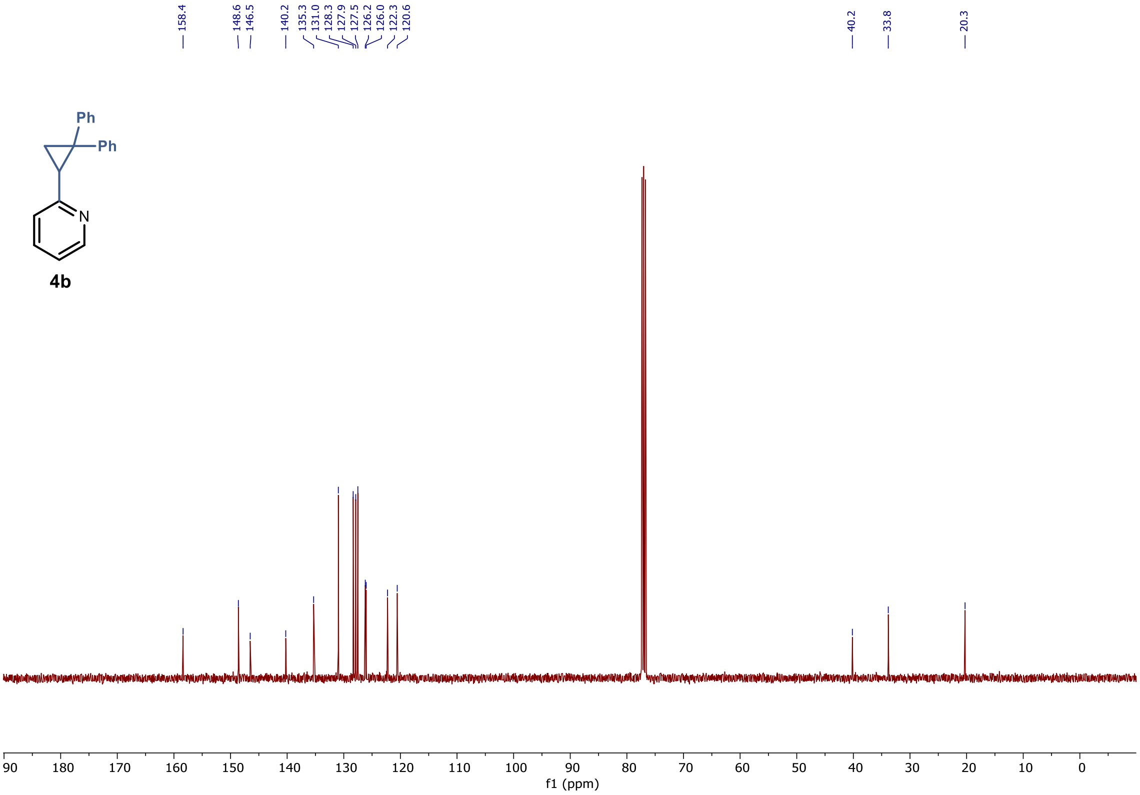

$^{1}$H NMR (400 MHz, $CDCl_3$) of **3a**

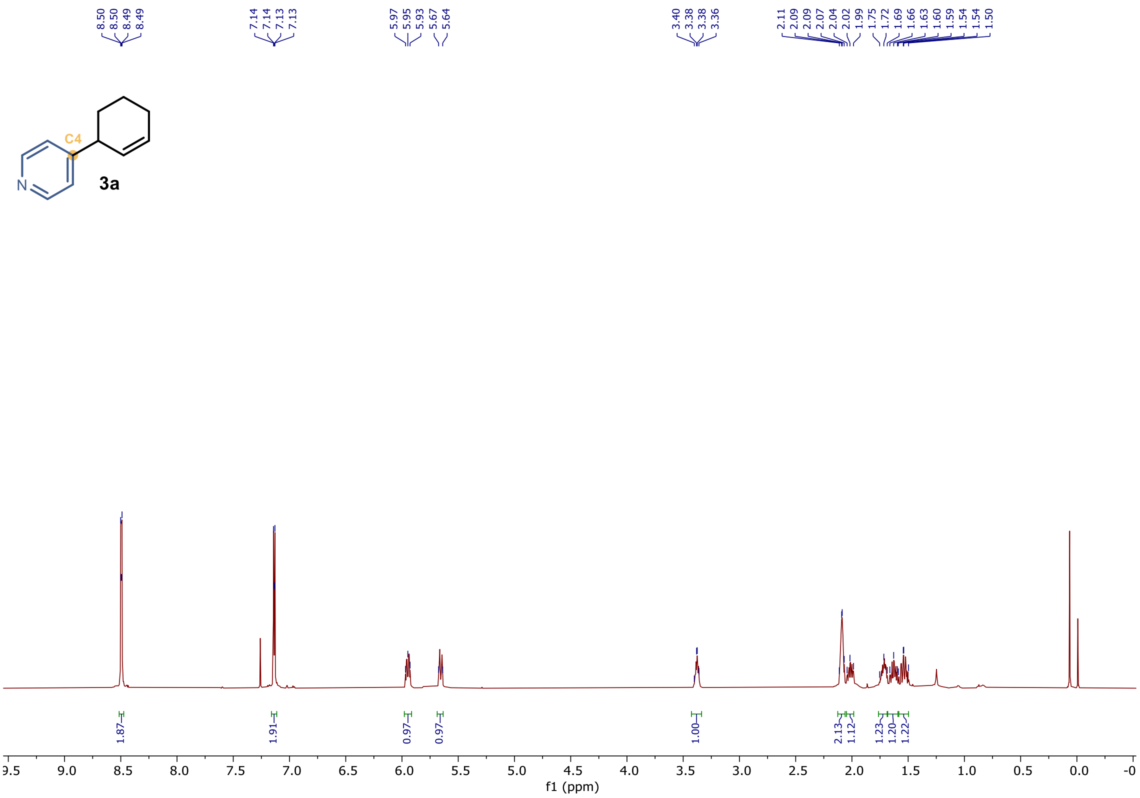

$^{13}$C NMR (126 MHz, $CDCl_3$) of **3a**

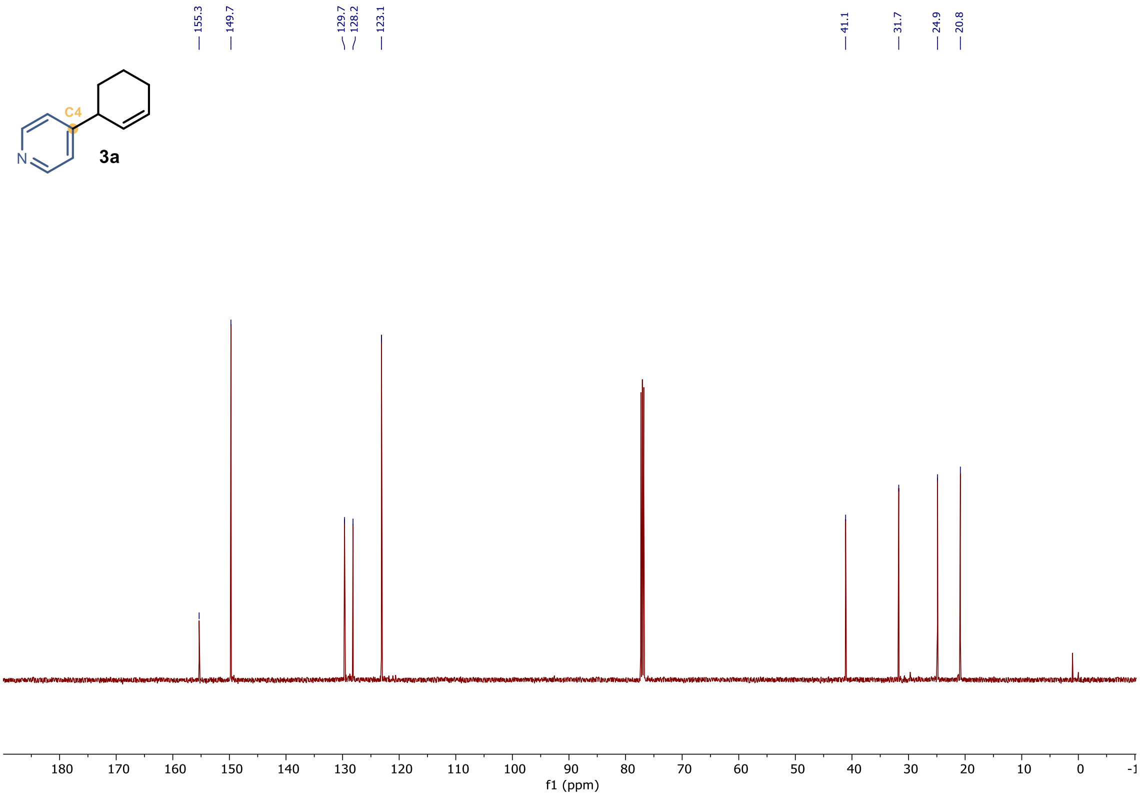

$^1$H NMR (500 MHz, $CDCl_3$) of **3b**

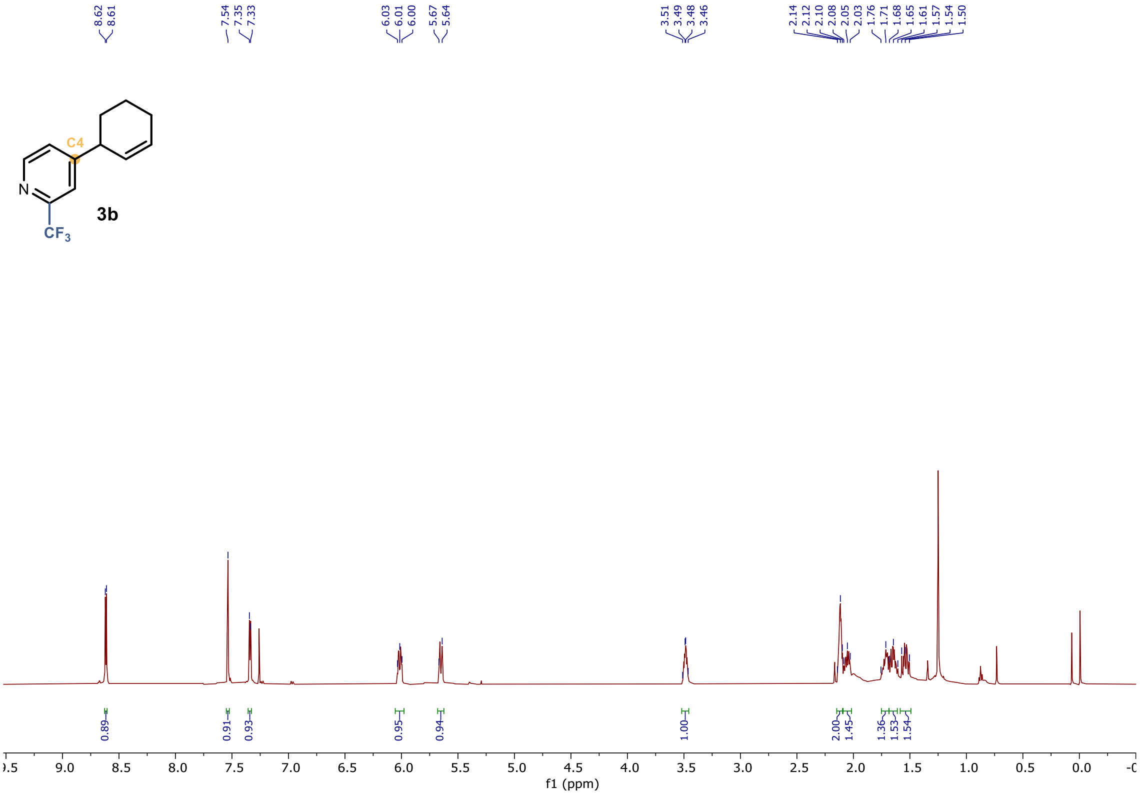


$^{13}$C NMR (126 MHz, $CDCl_3$) of **3b**

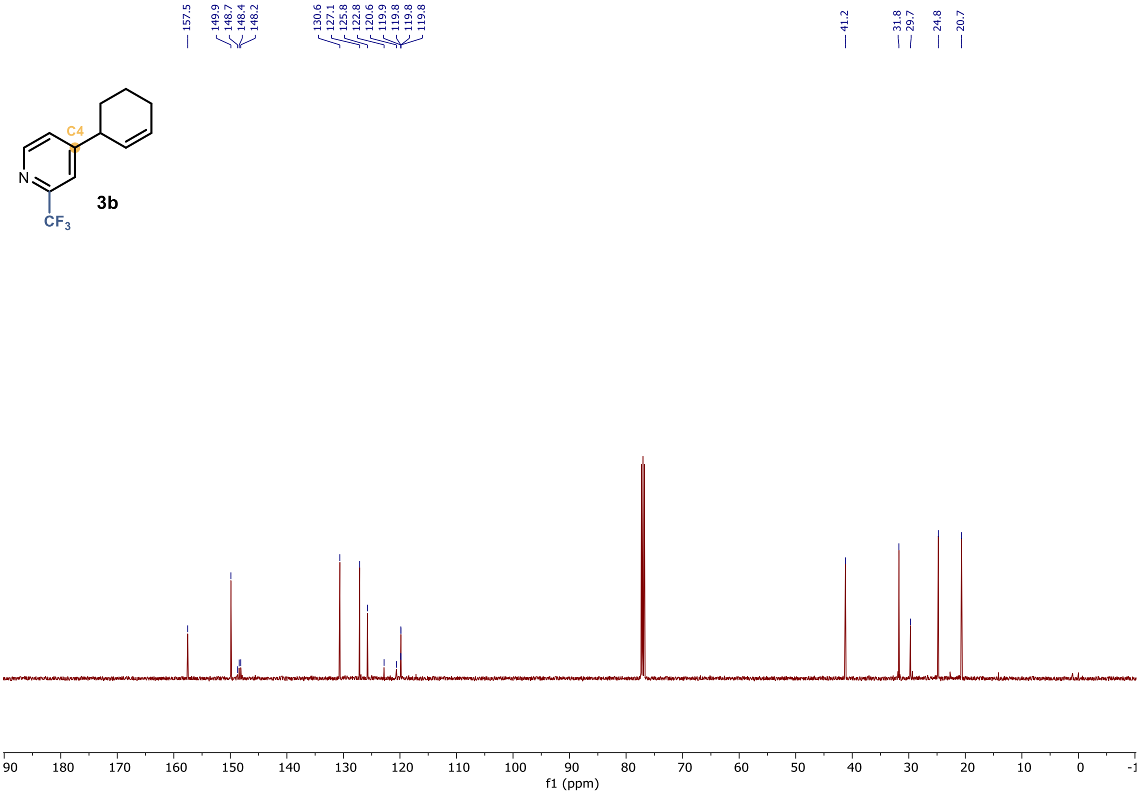

$^{19}$F NMR (471 MHz, $CDCl_3$) of **3b**

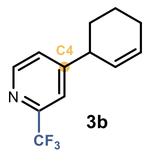


3b

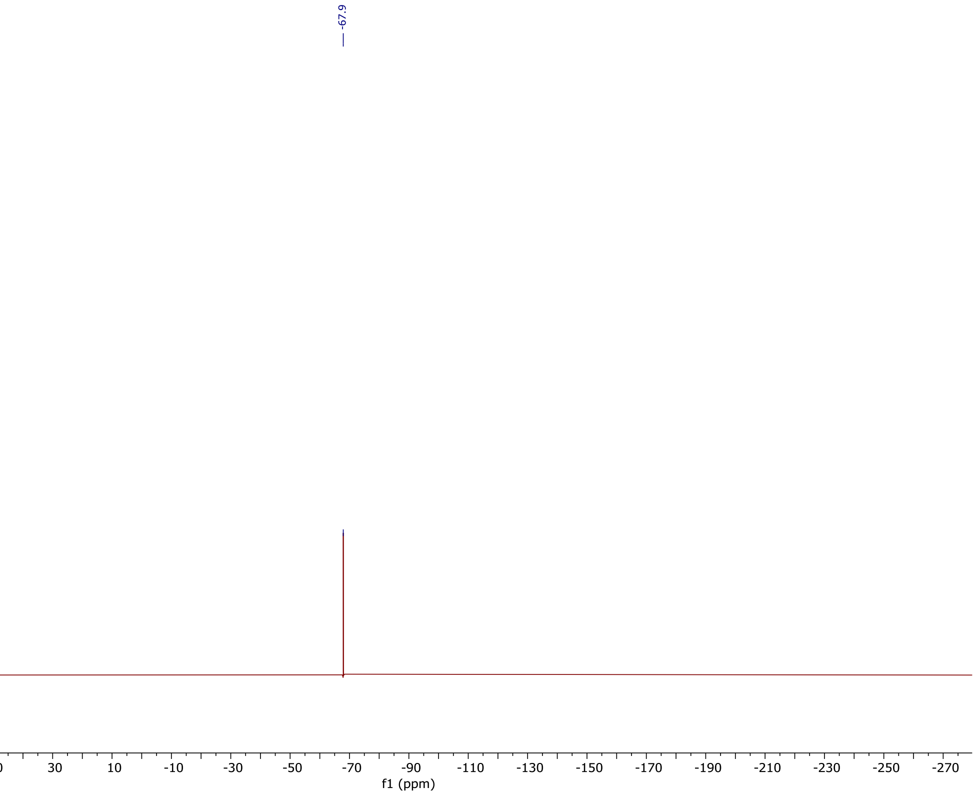

$^{1}H$ NMR (500 MHz, $CDCl_3$) of **3c**

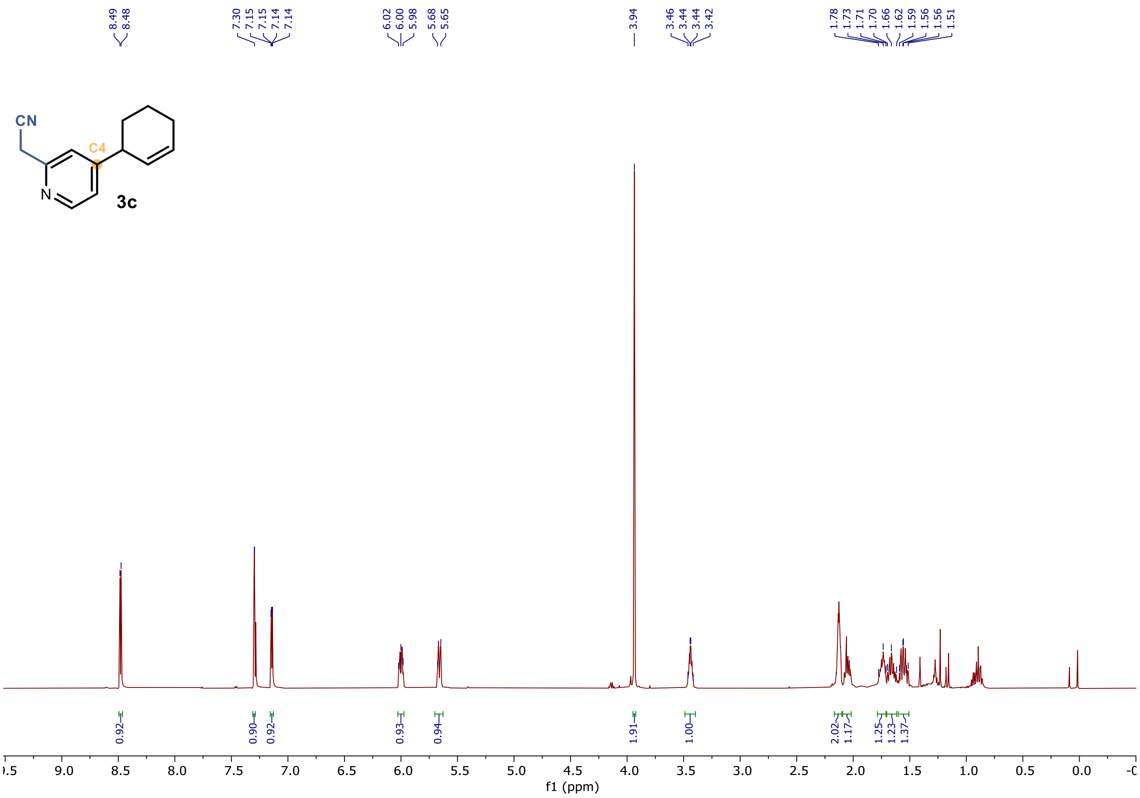

$^{13}C$ NMR (126 MHz, $CDCl_3$) of **3c**

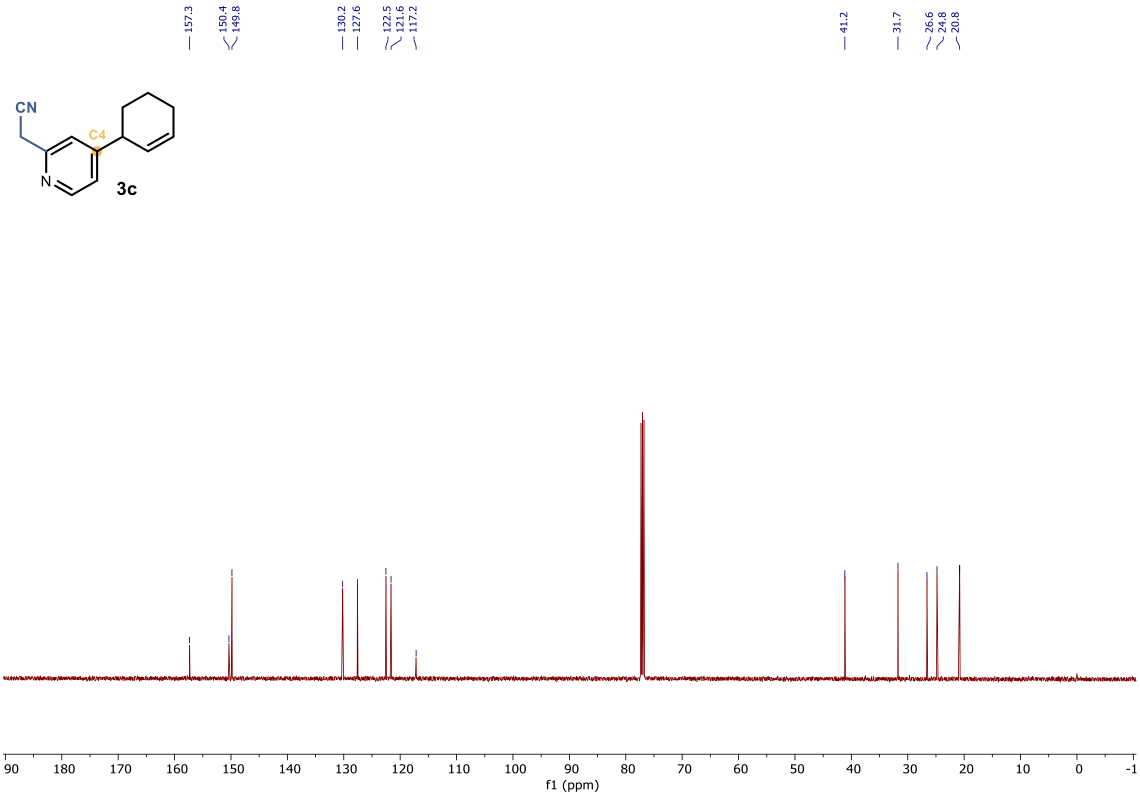

$^{1}$H NMR (500 MHz, $CDCl_3$) of **3d**

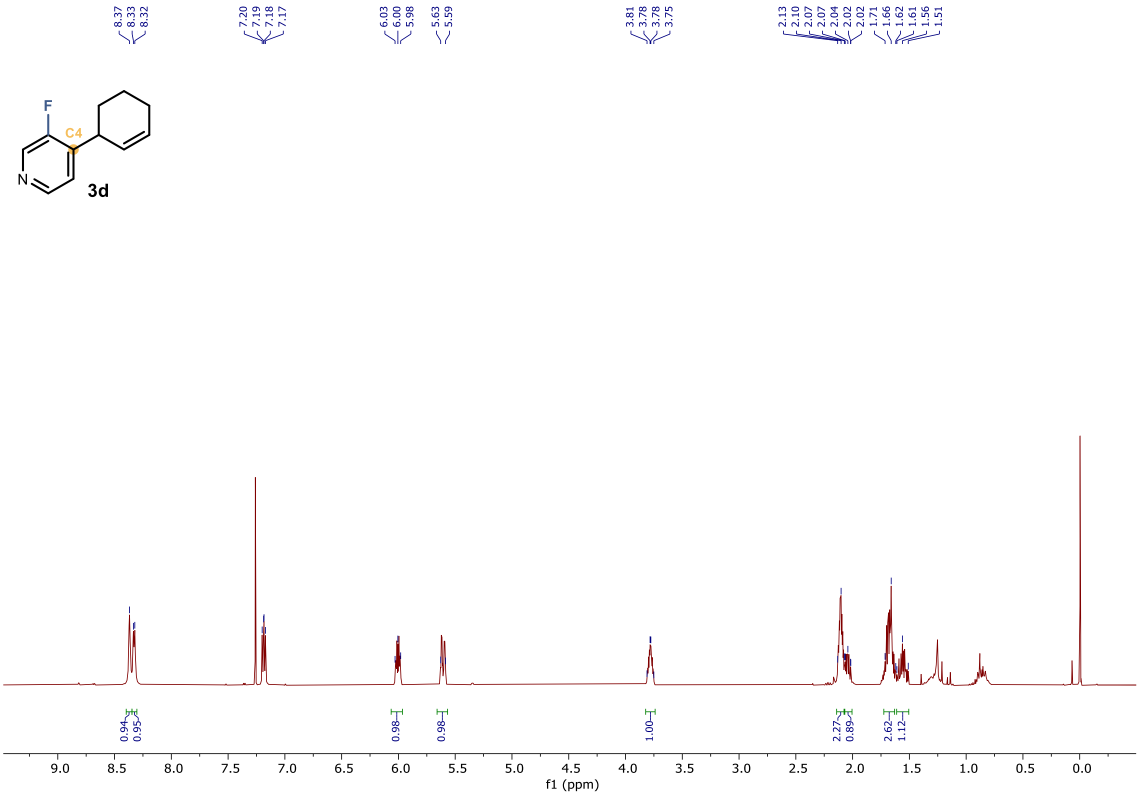


$^{13}$C NMR (126 MHz, $CDCl_3$) of **3d**

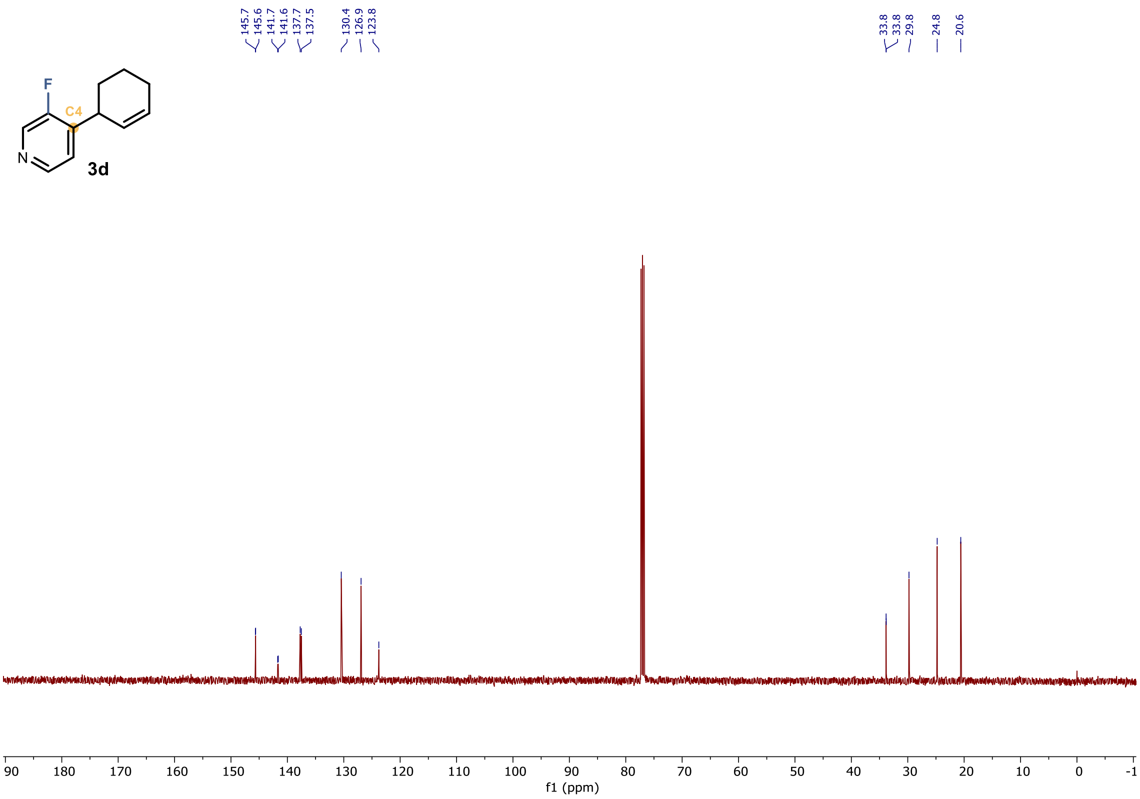

$^{19}F\{^{1}H\}$ NMR (471 MHz, $CDCl_3$) of **3d**

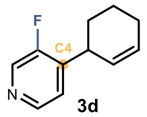


**3d**

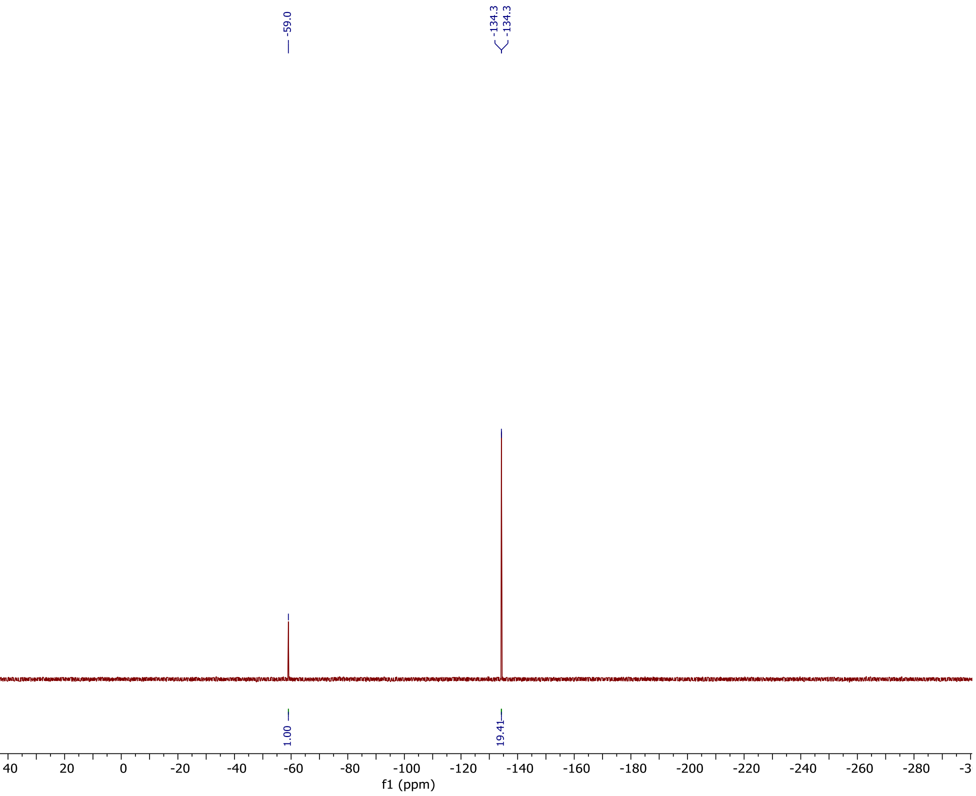

$^{1}$H NMR (400 MHz, $CDCl_3$) of **3e +3e'**

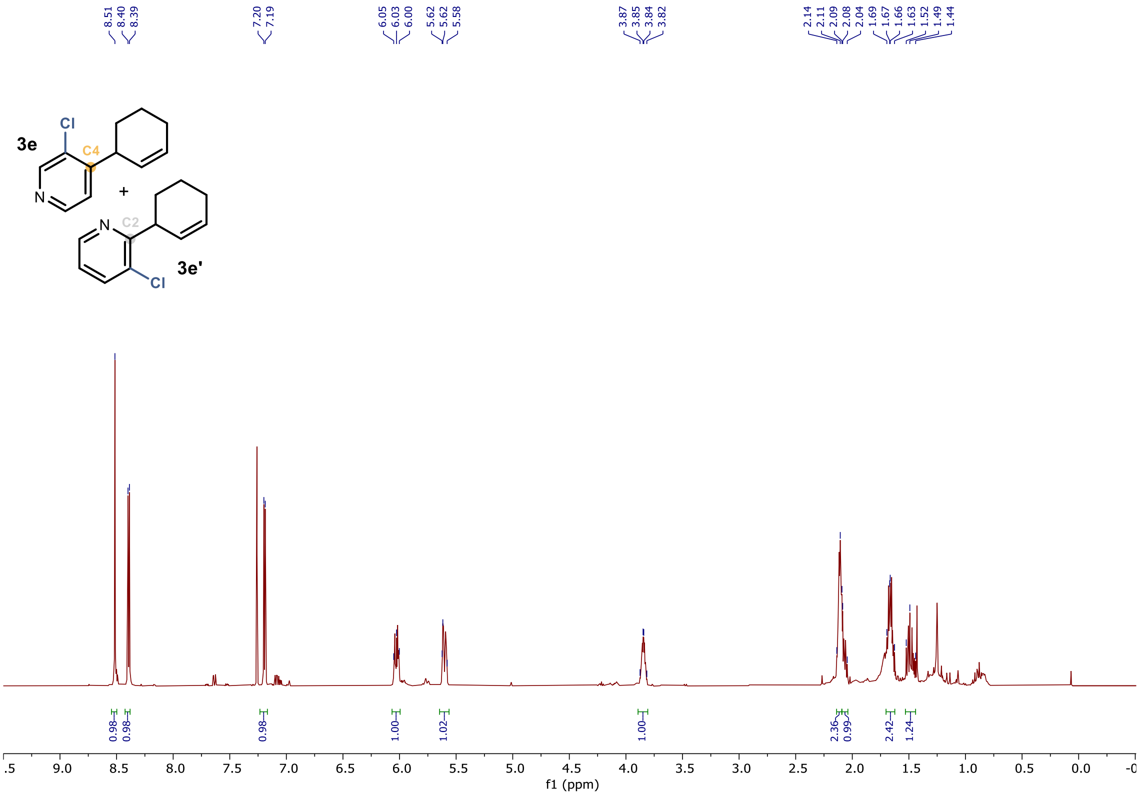


$^{13}$C NMR (101 MHz, $CDCl_3$) of **3e +3e'**

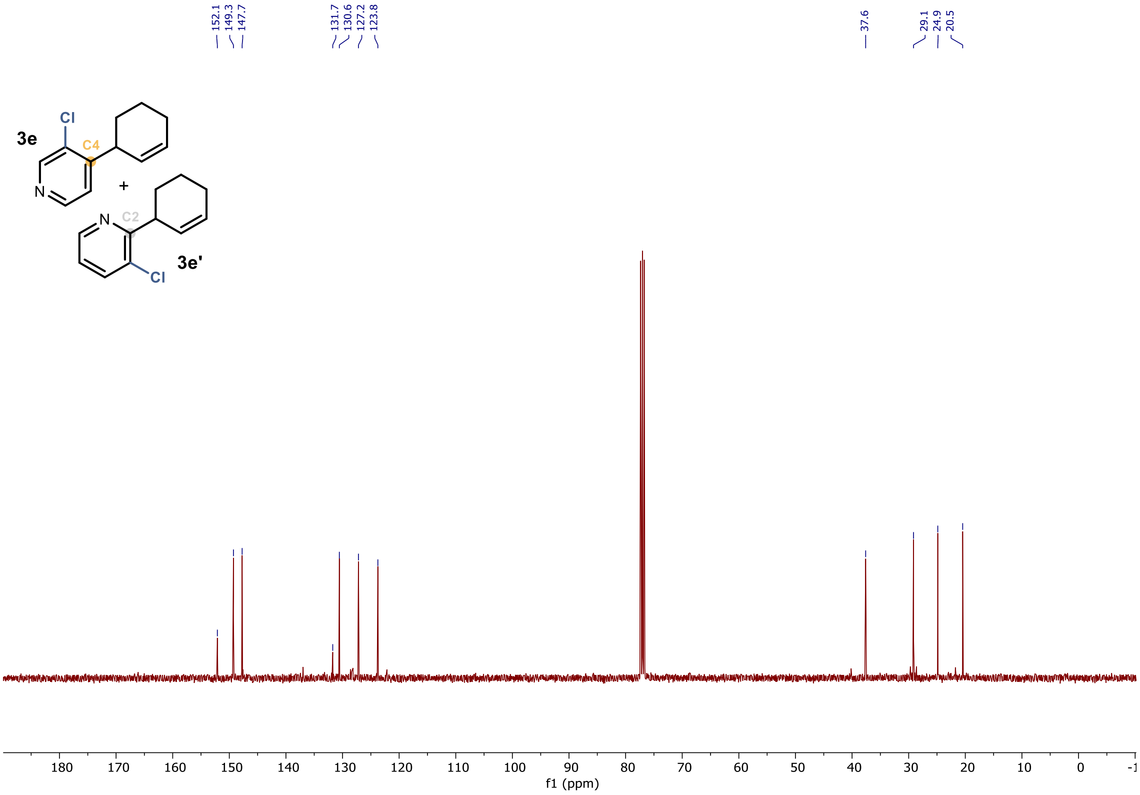

$^{1}$H NMR (500 MHz, $CDCl_3$) of **3f**

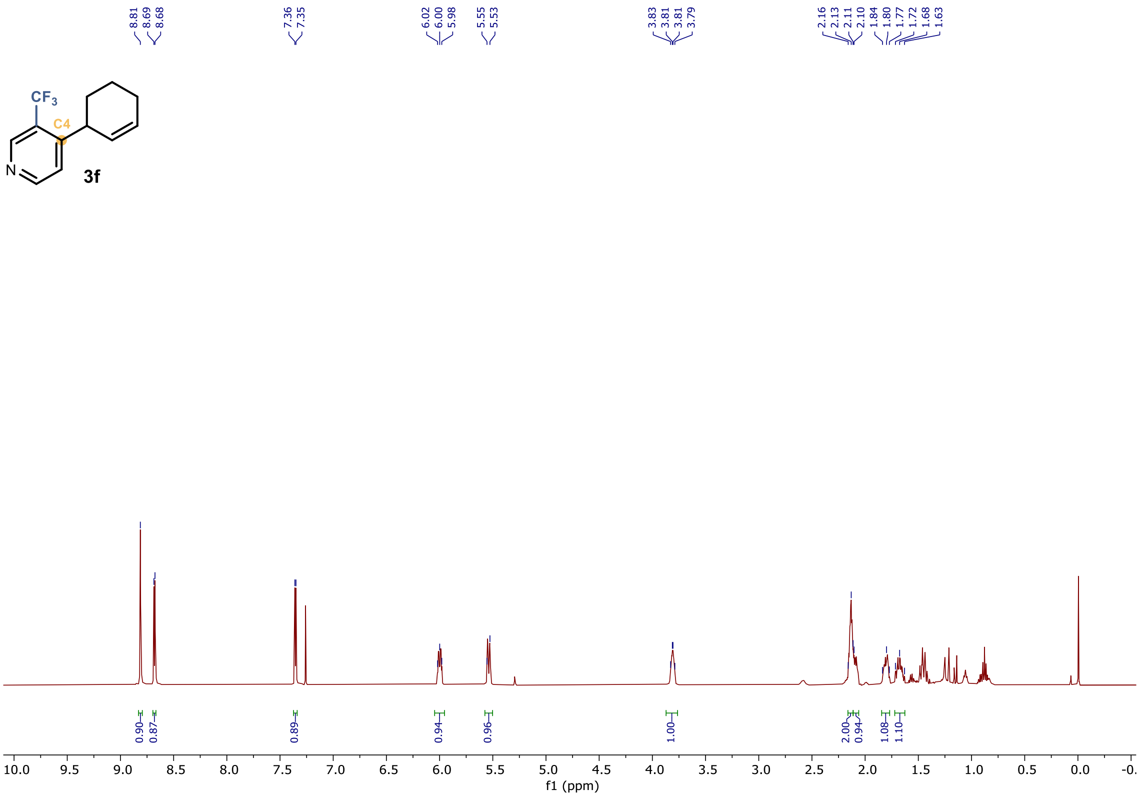


$^{13}$C NMR (126 MHz, $CDCl_3$) of **3f**

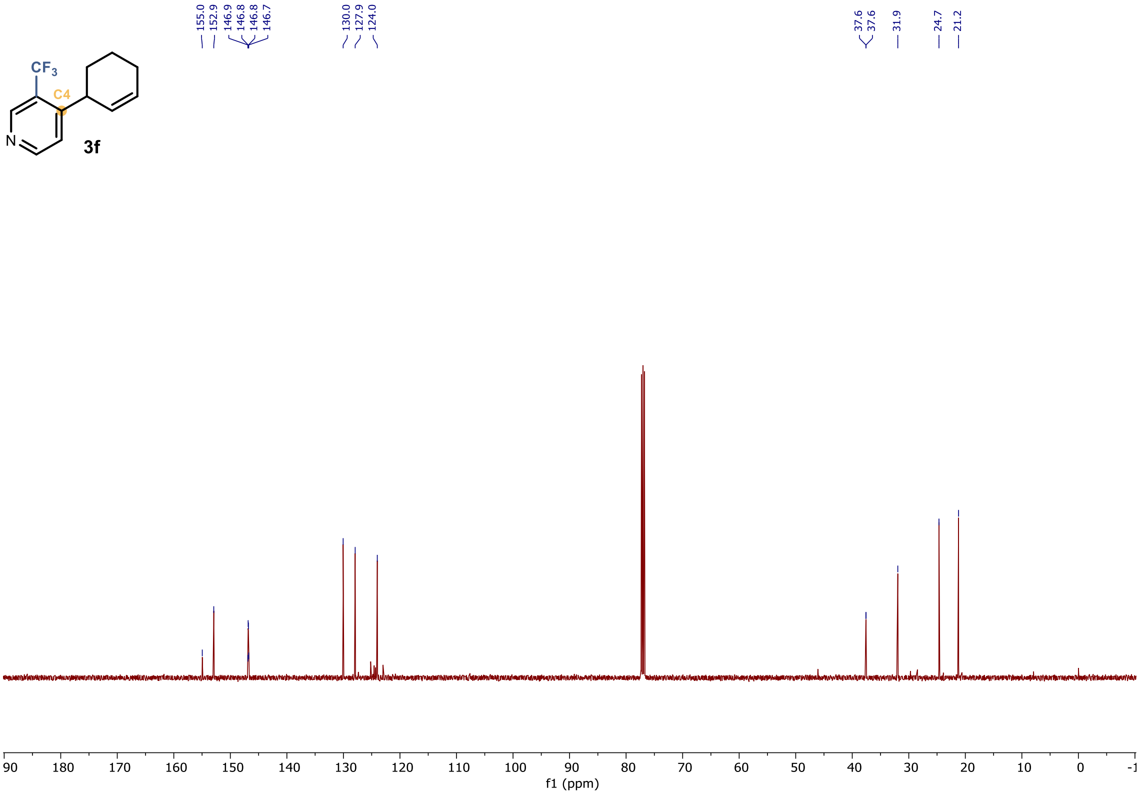

$^{19}F\{^{1}H\}$ NMR (471 MHz, $CDCl_3$) of **3f**

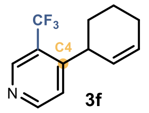

**3f**

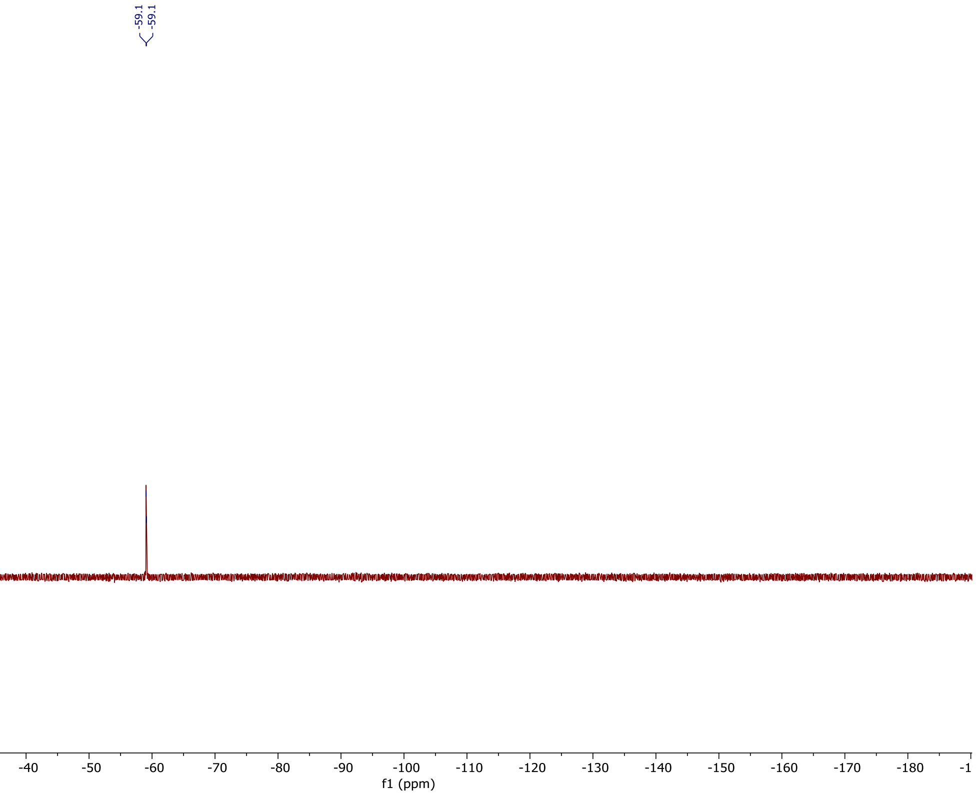

$^{1}$H NMR (400 MHz, $CDCl_3$) of **3g**

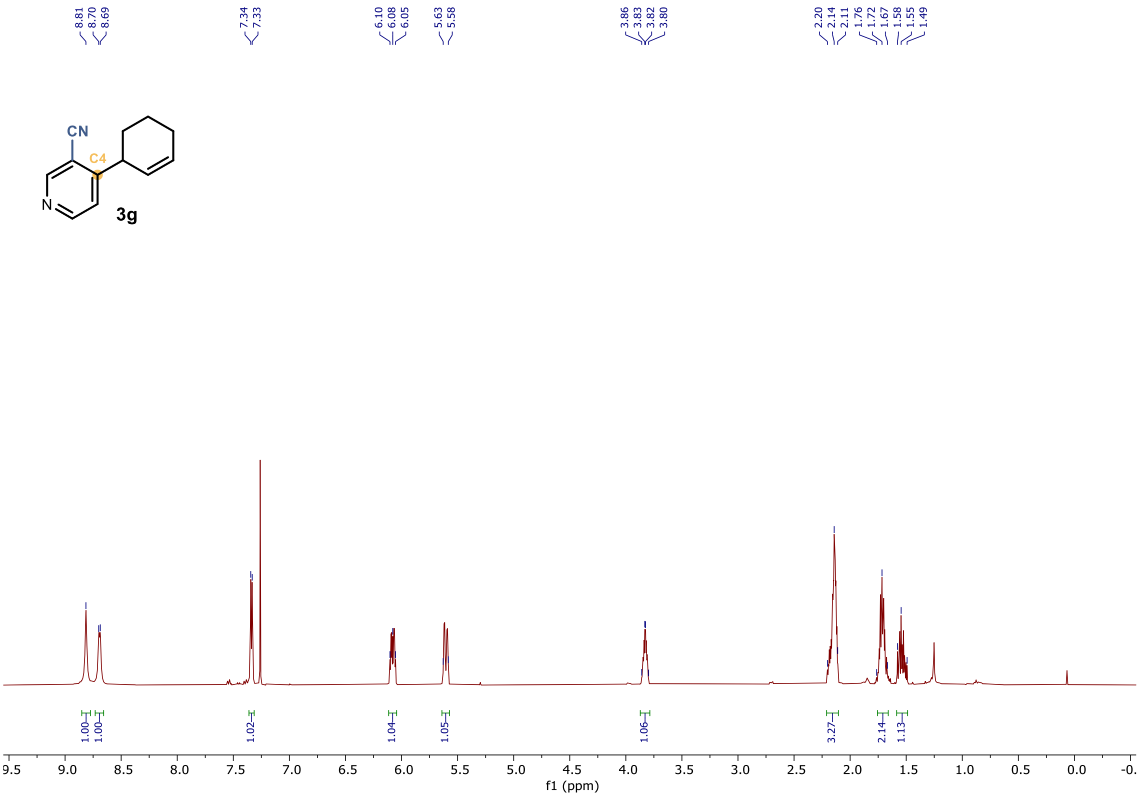

$^{13}$C NMR (101 MHz, $CDCl_3$) of **3g**

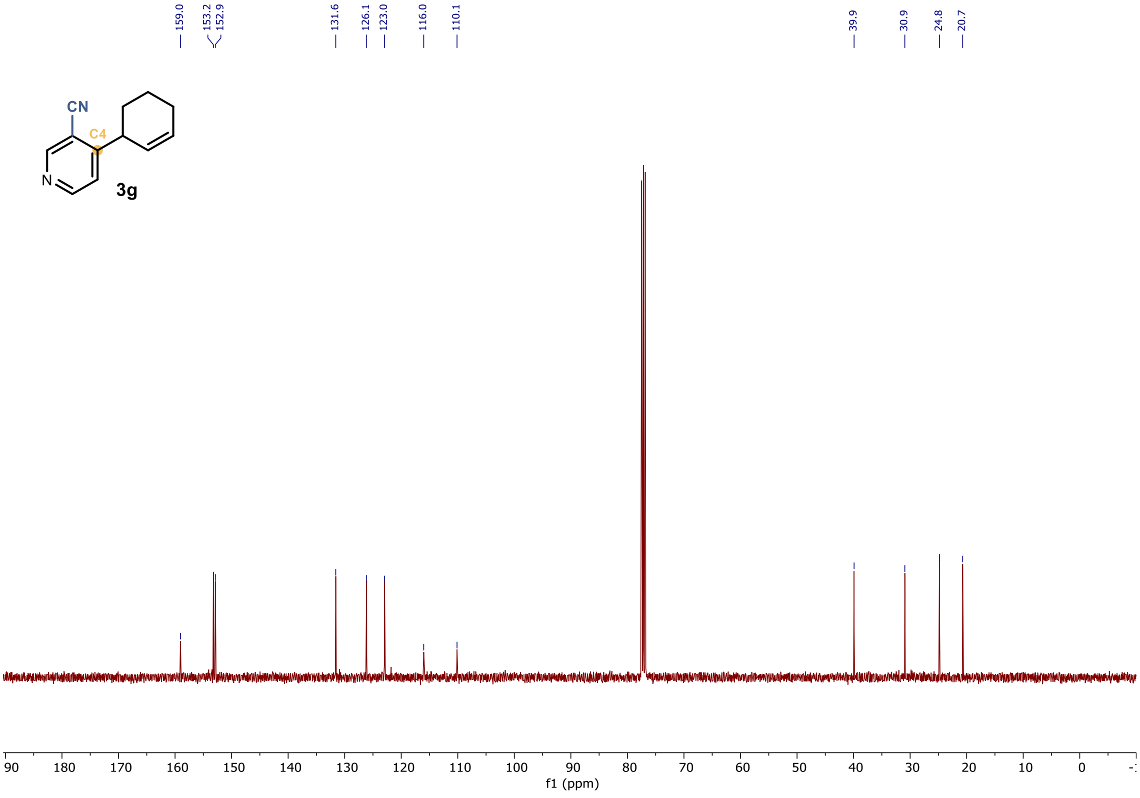

$^{1}H$ NMR (400 MHz, $CDCl_3$) of **3h**

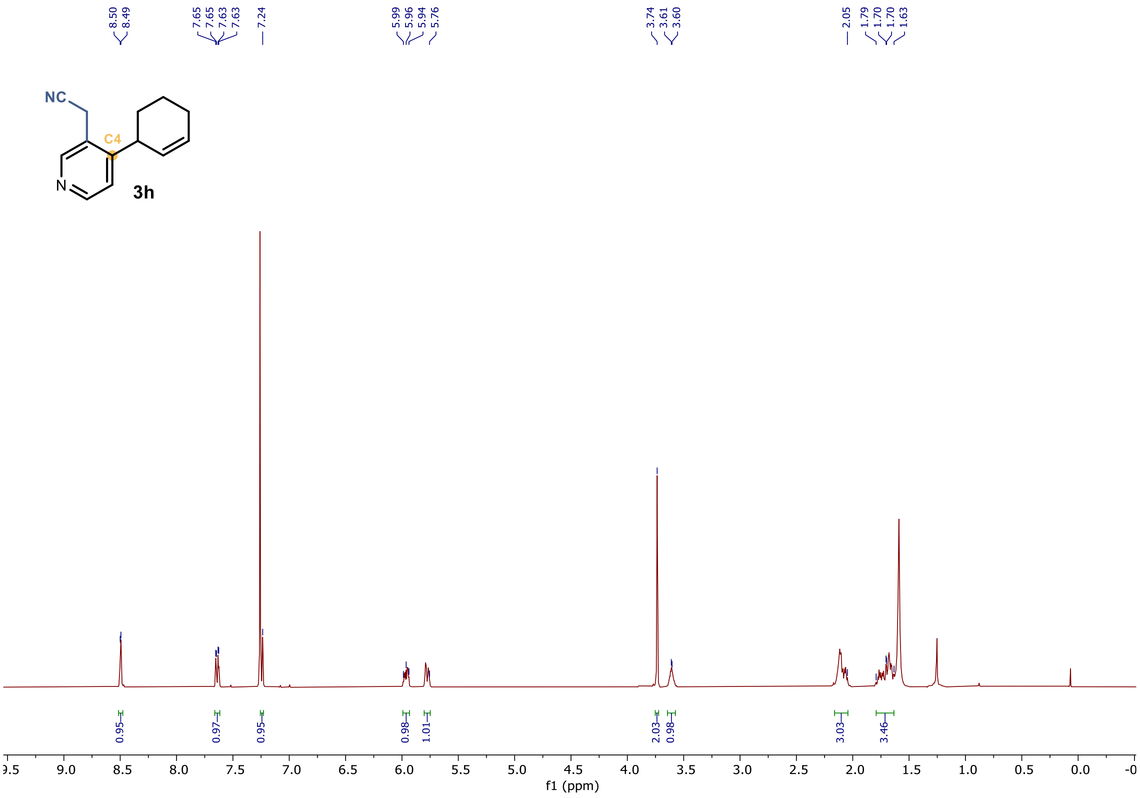


$^{13}C$ NMR (101 MHz, $CDCl_3$) of **3h**

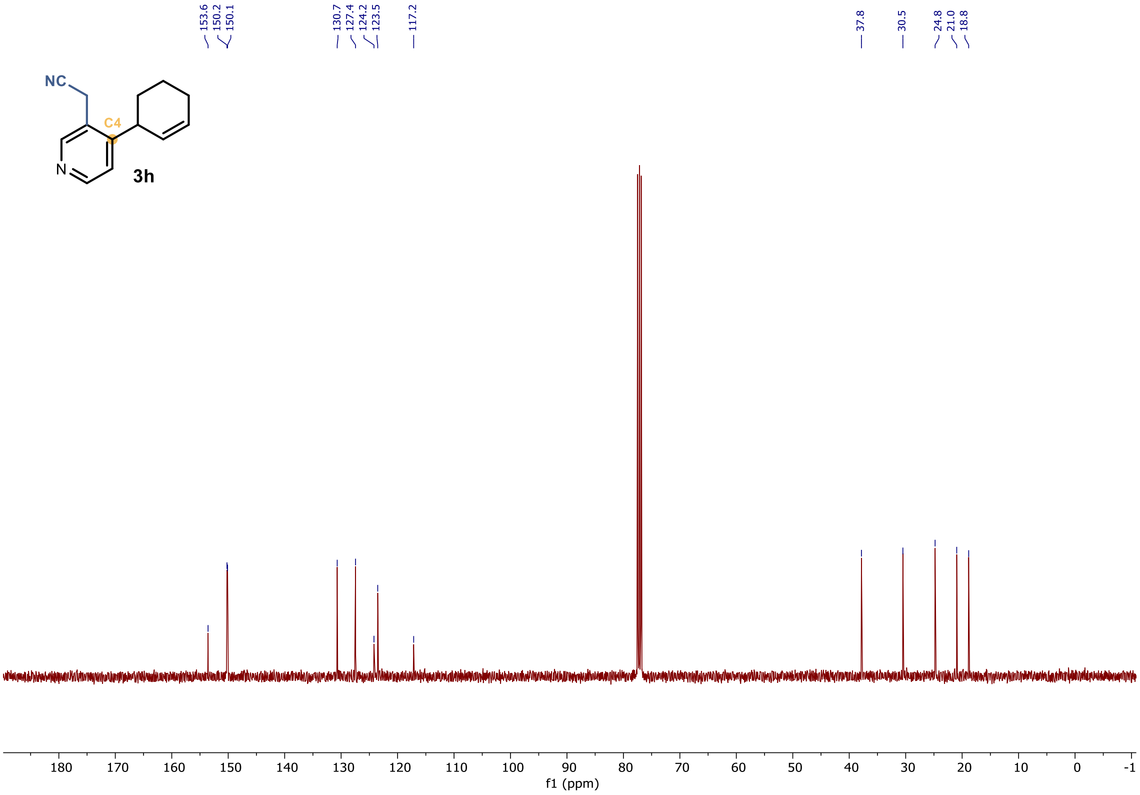

$^{1}$H NMR (400 MHz, $CDCl_3$) of **3i**

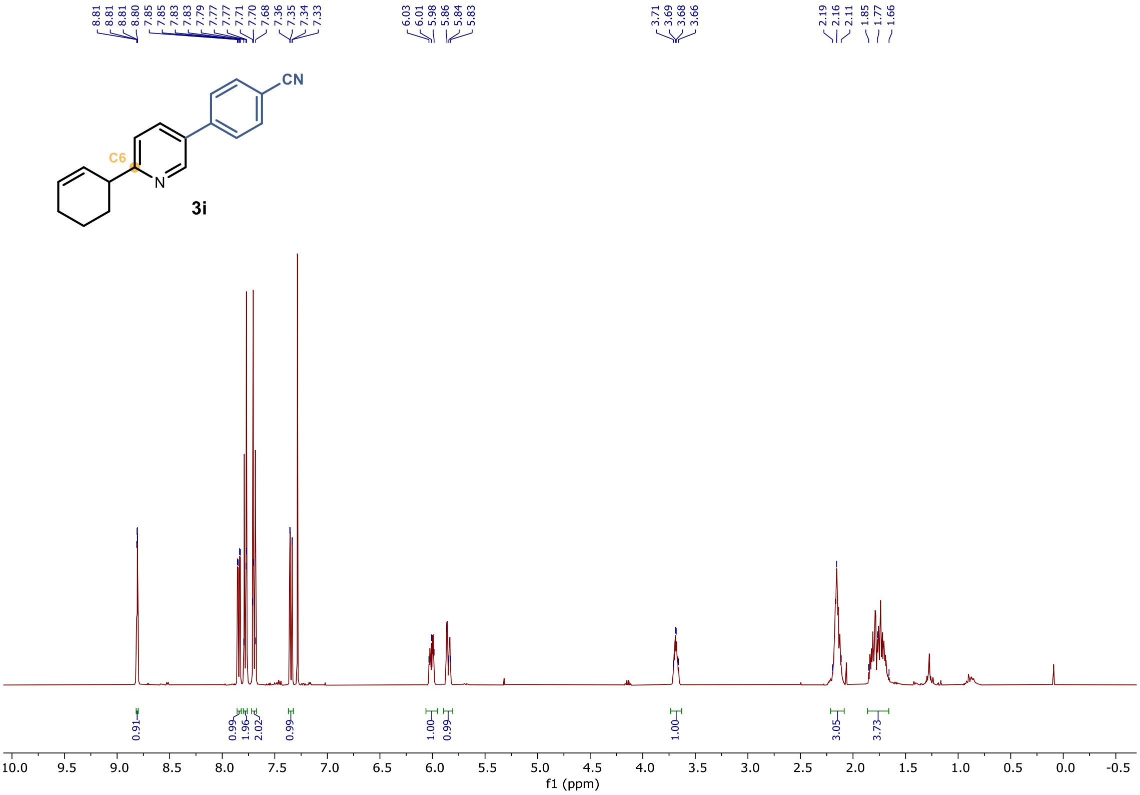


$^{13}$C NMR (101 MHz, $CDCl_3$) of **3i**

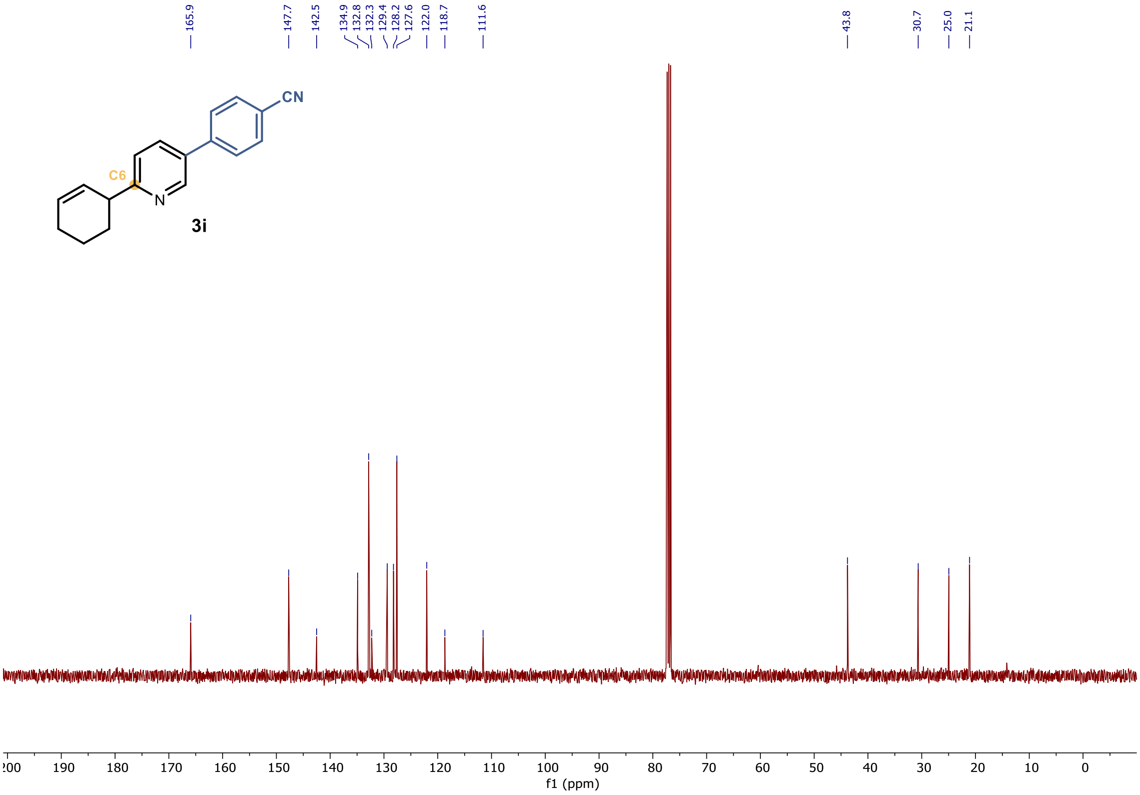

$^{1}$H NMR (400 MHz, MeOD) of **3j**

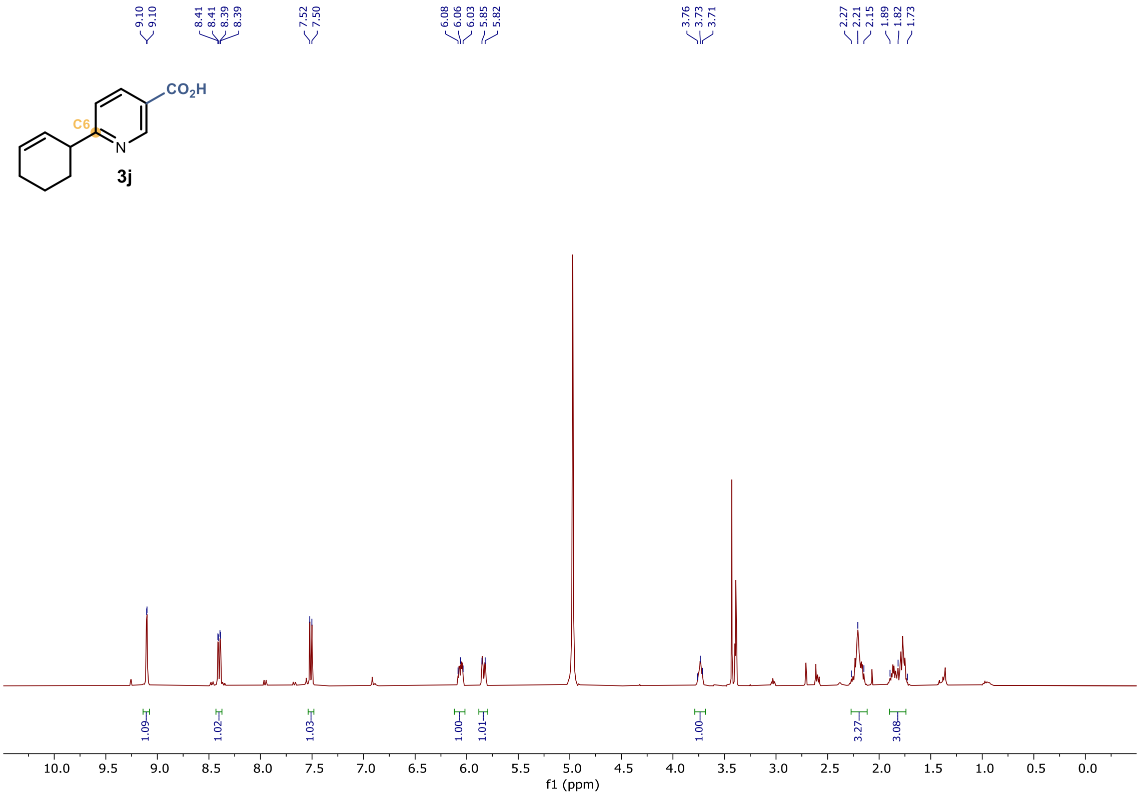


$^{13}$C NMR (101 MHz, MeOD) of **3j**

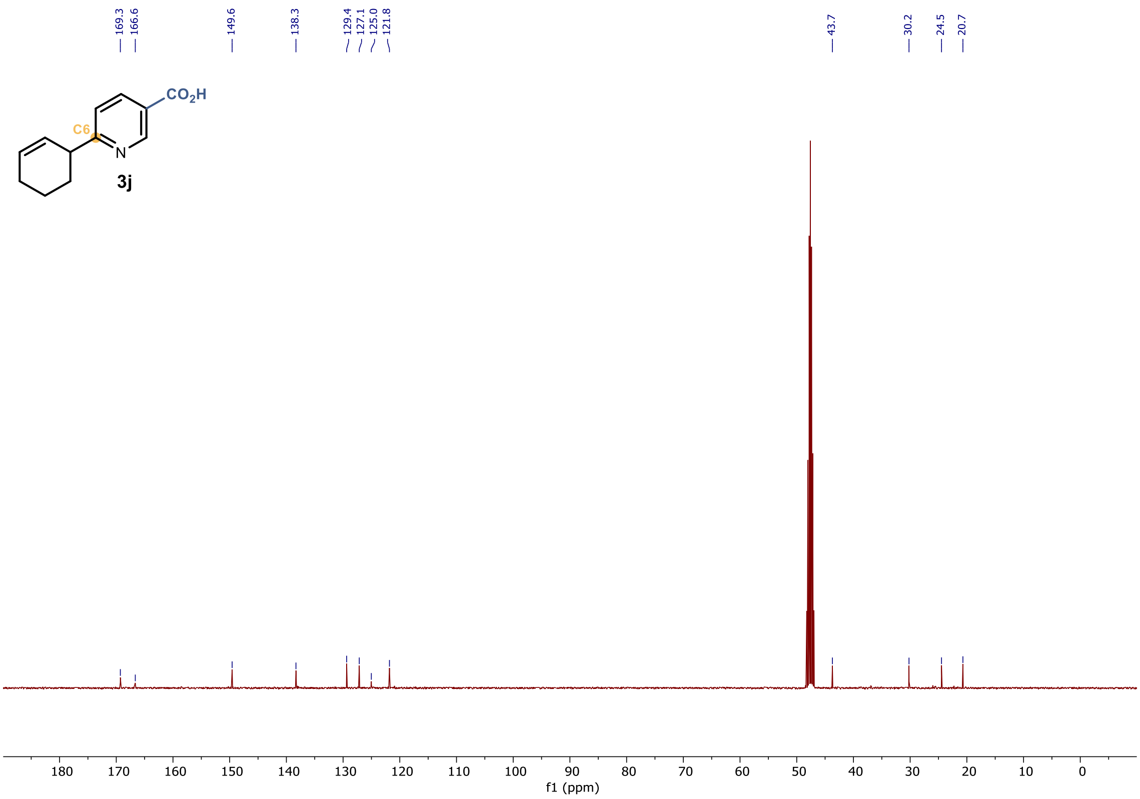

$^{1}$H NMR (400 MHz, MeOD) of **3k**

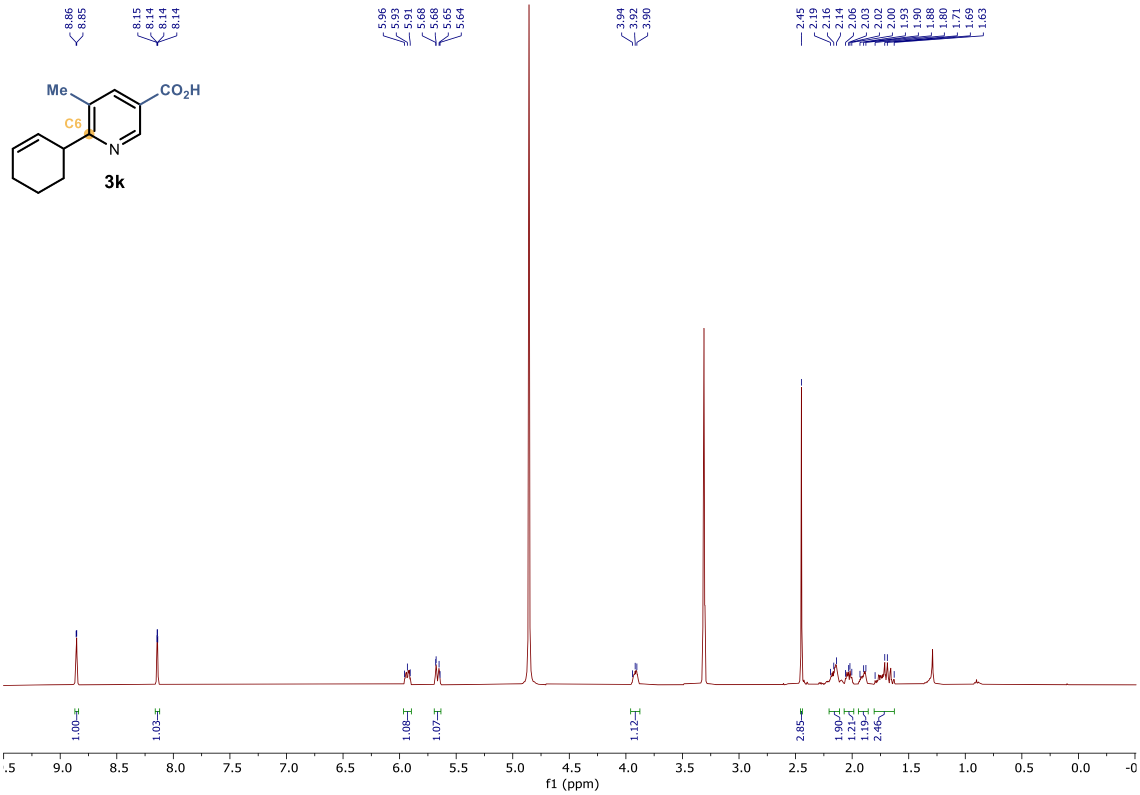

$^{13}$C NMR (101 MHz, MeOD) of **3k**

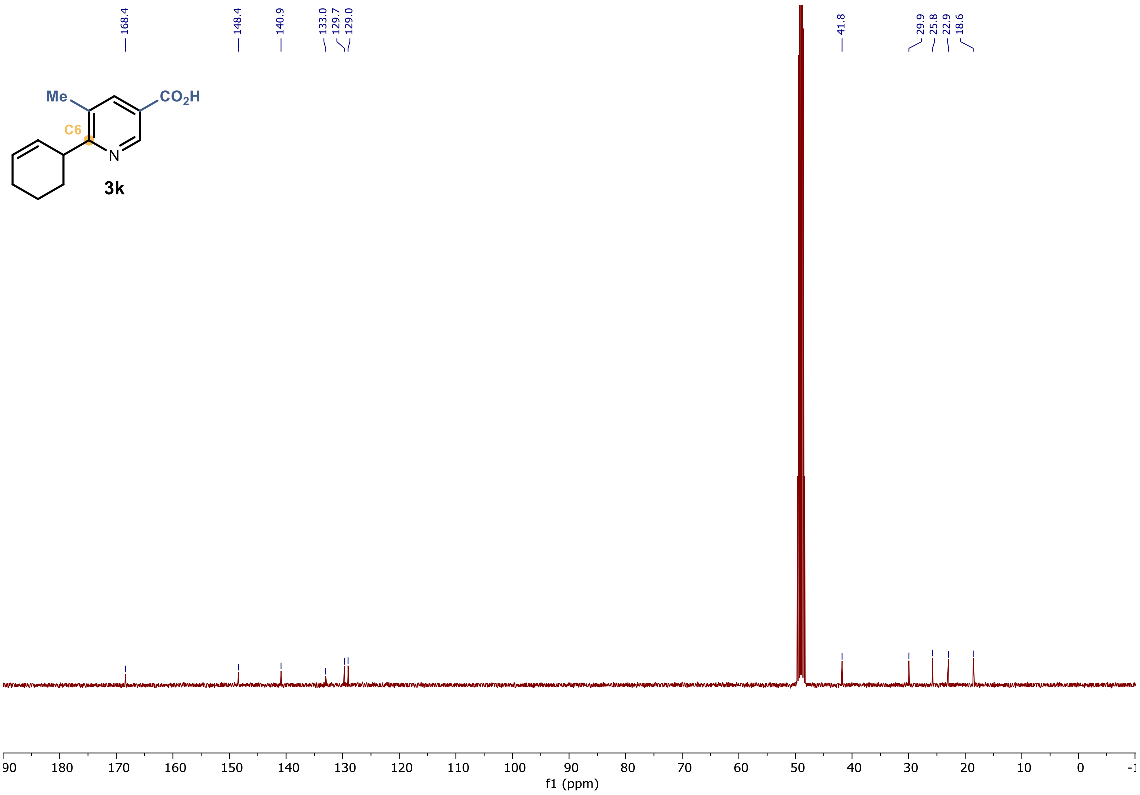

$^{1}$H NMR (400 MHz, $CDCl_3$) of **3l**

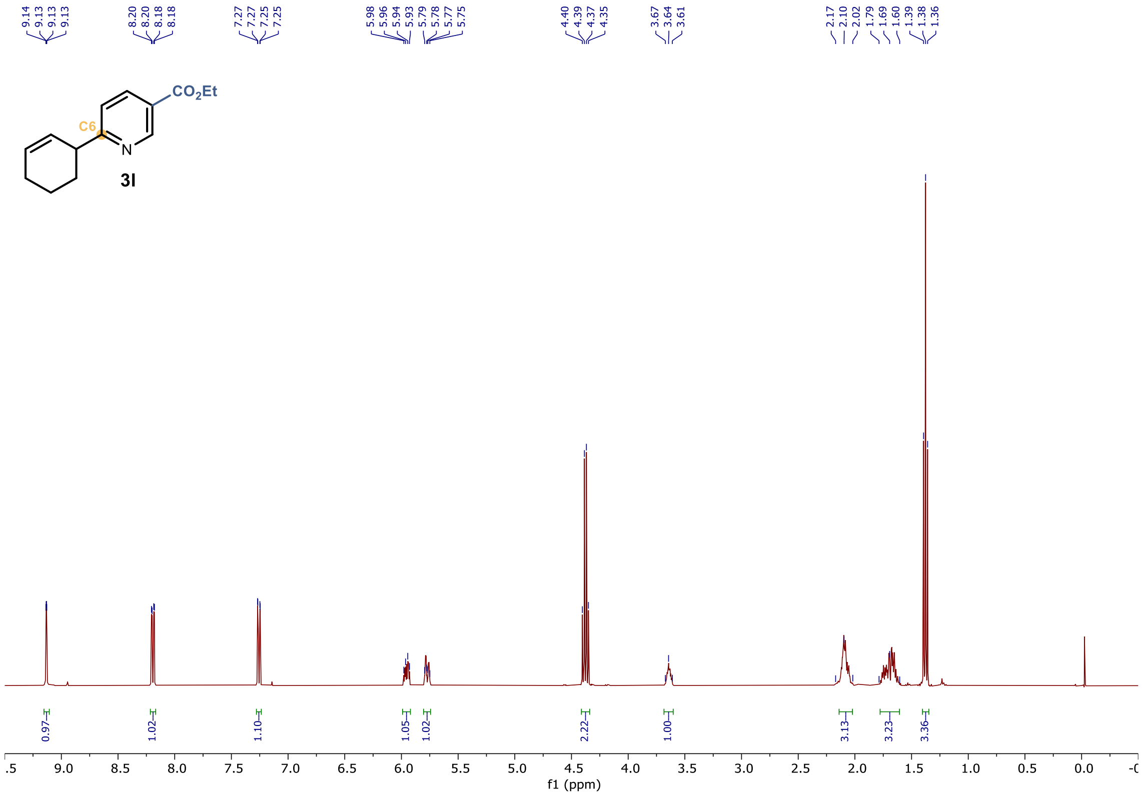


$^{13}$C NMR (101 MHz, $CDCl_3$) of **3l**

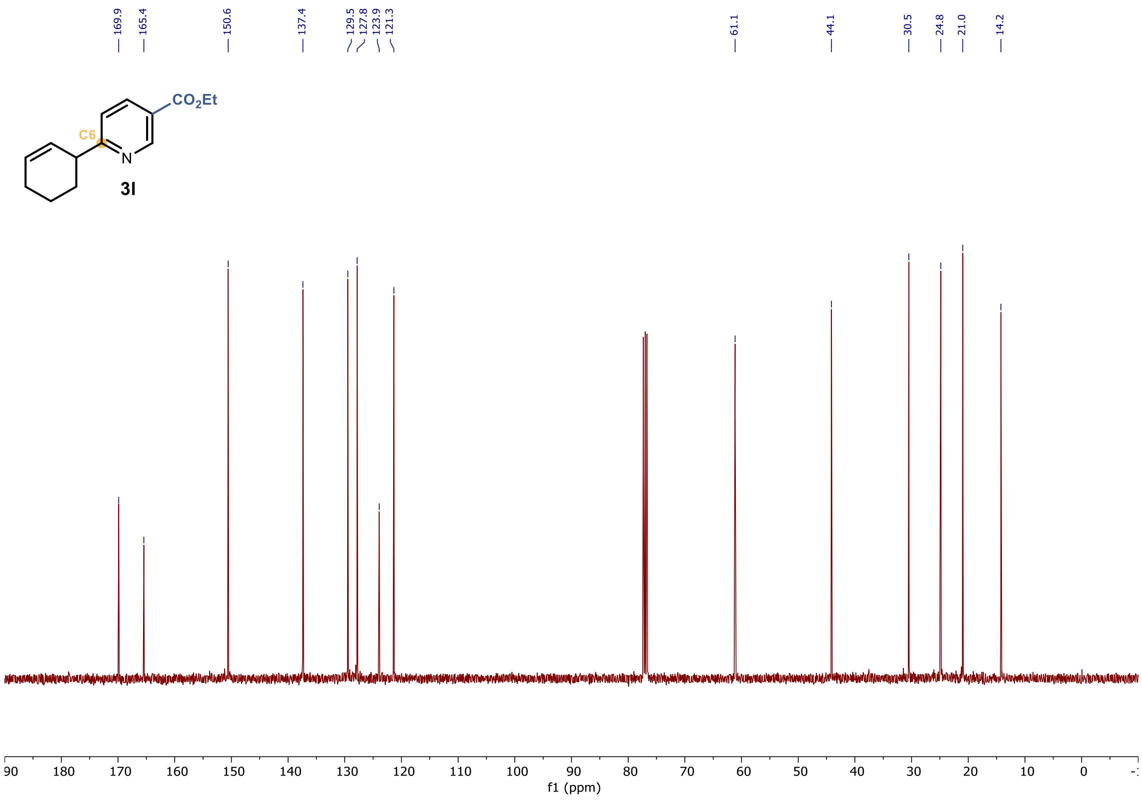

$^1$H NMR (400 MHz, $CDCl_3$) of **3m**

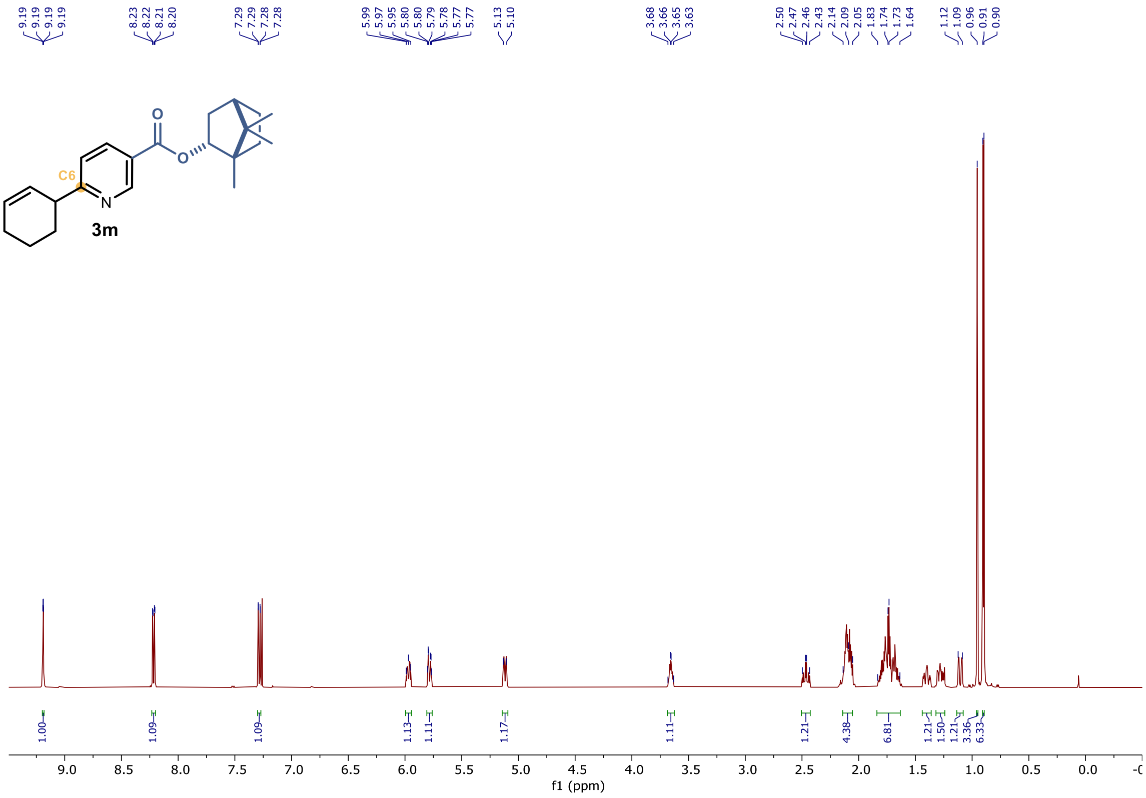

$^{13}$C NMR (101 MHz, $CDCl_3$) of **3m**

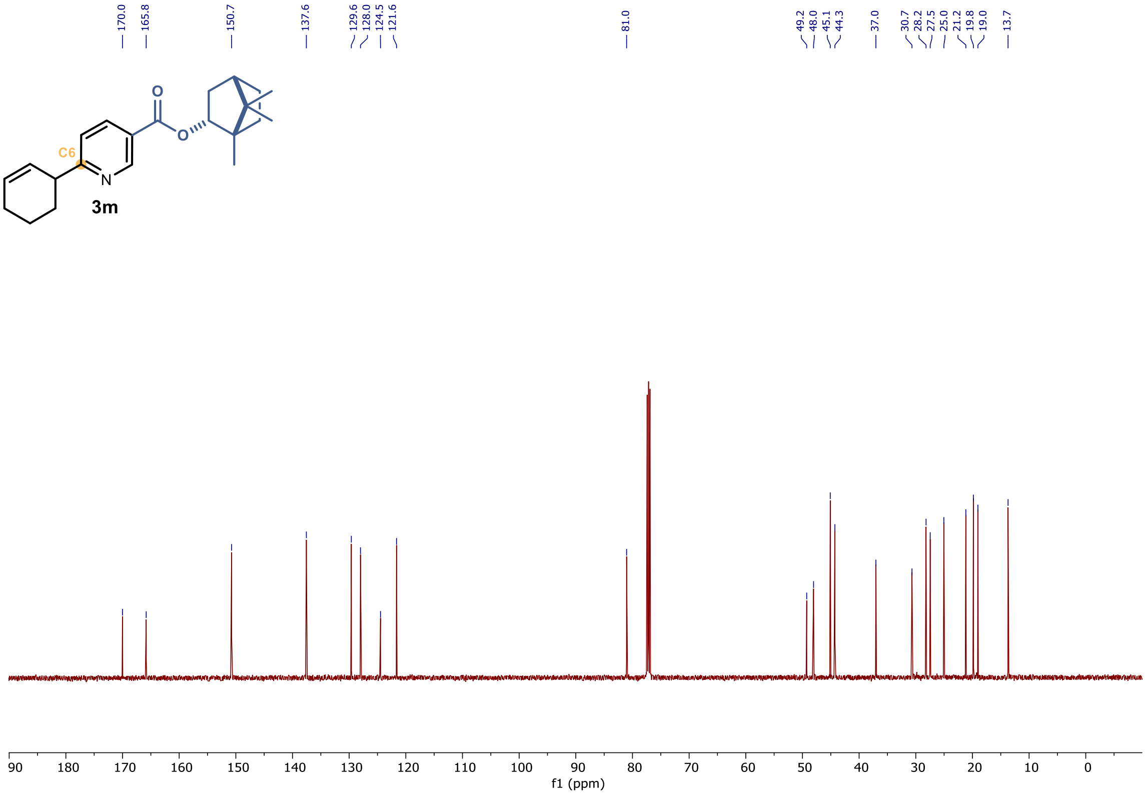

$^{1}H$ NMR (400 MHz, $CDCl_3$) of **3n**

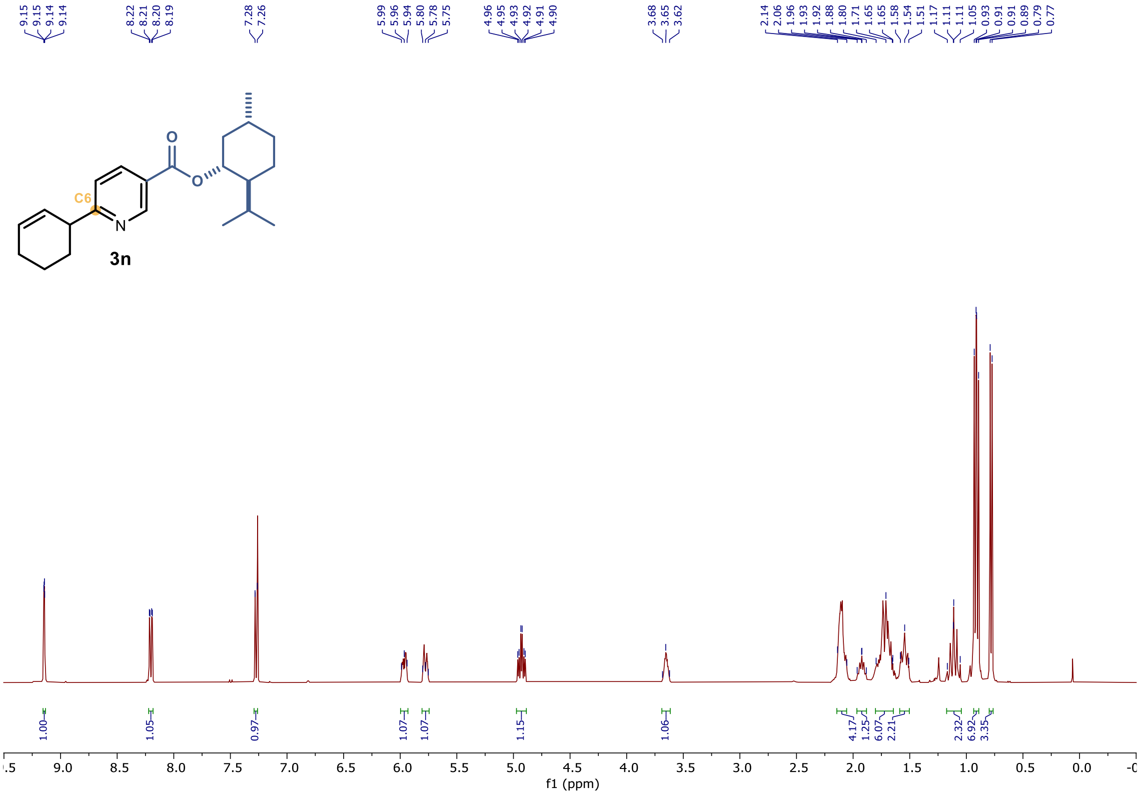


$^{13}C$ NMR (101 MHz, $CDCl_3$) of **3n**

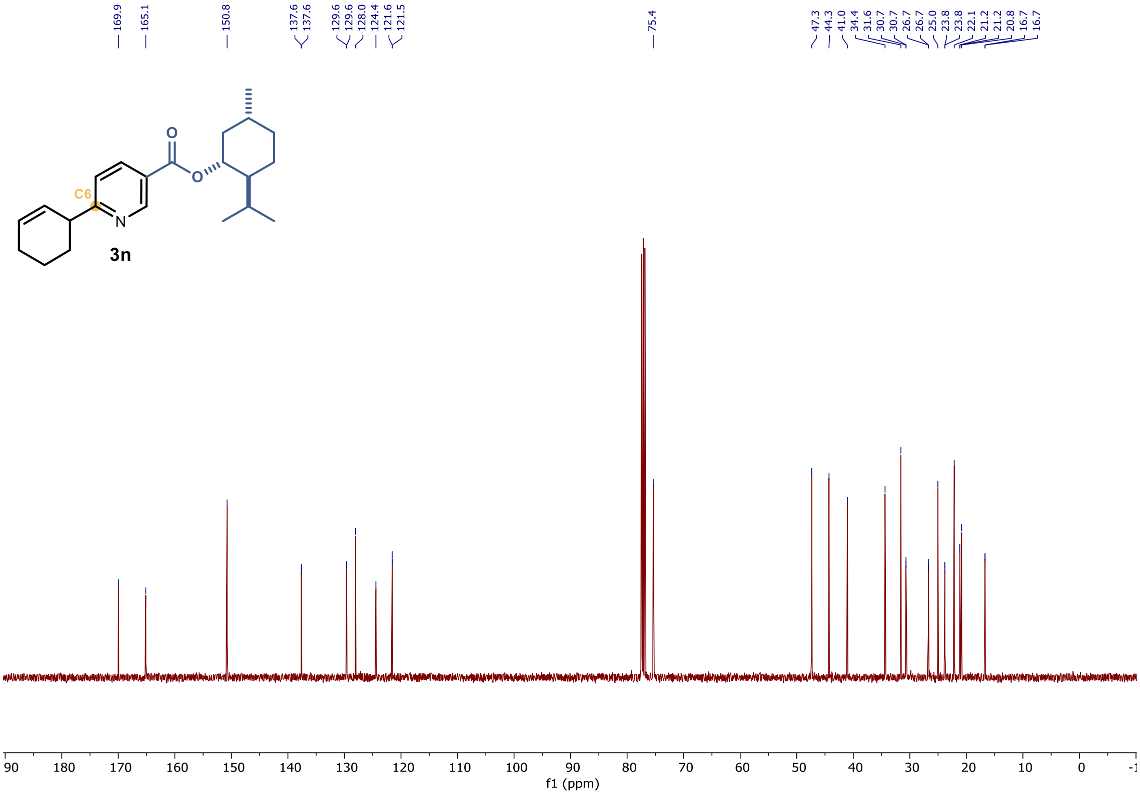

## $^1$H NMR (400 MHz, $CDCl_3$) of **3o**

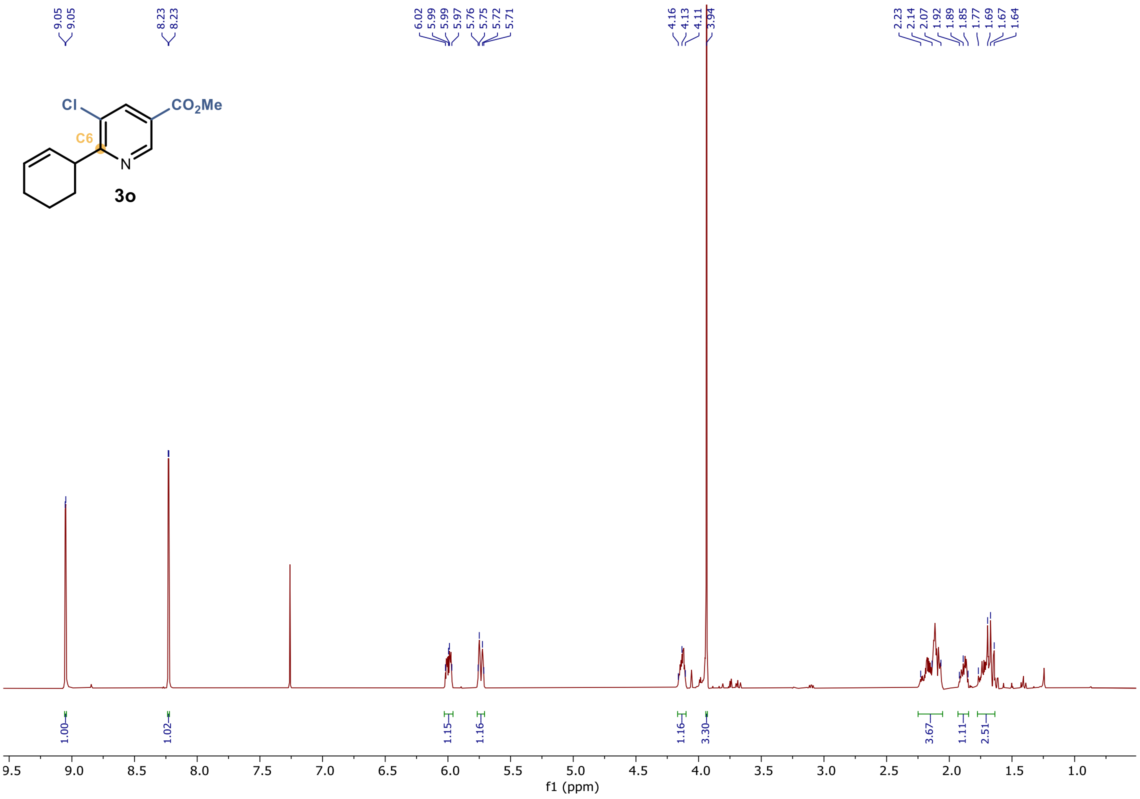


## $^{13}$C NMR (101 MHz, $CDCl_3$) of **3o**

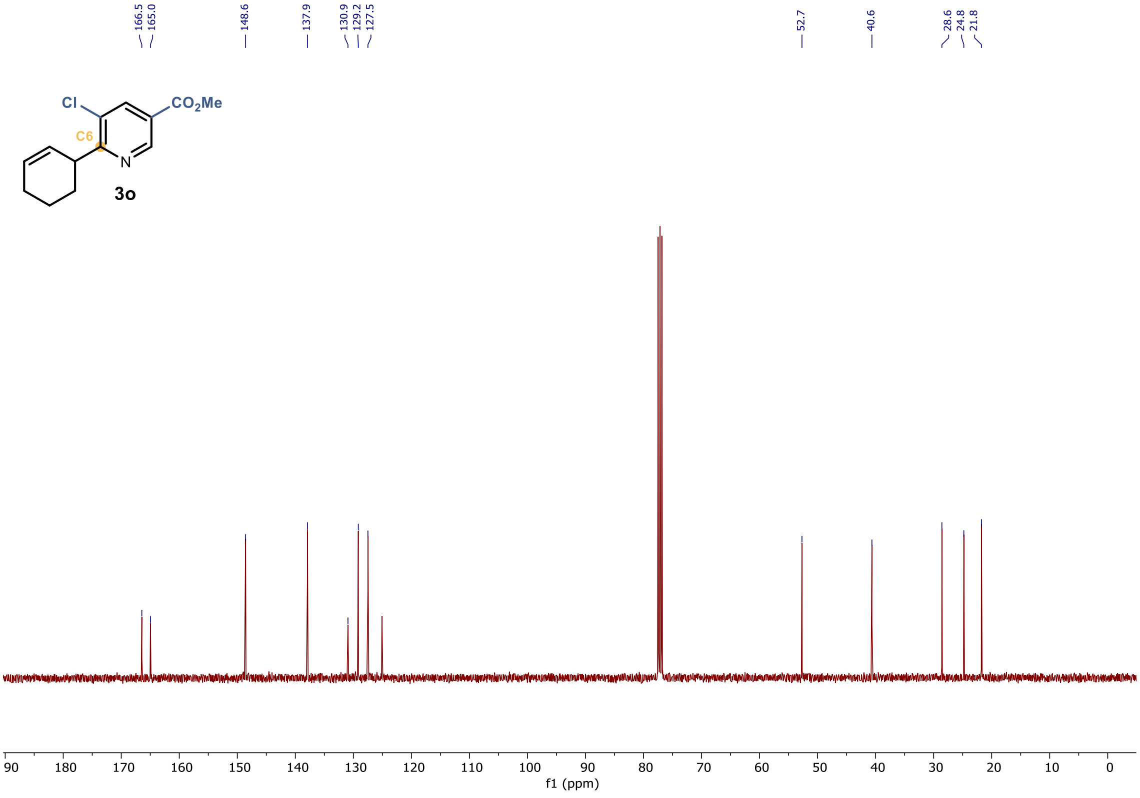

$^{1}$H NMR (400 MHz, CDCl$_3$) of **3p**

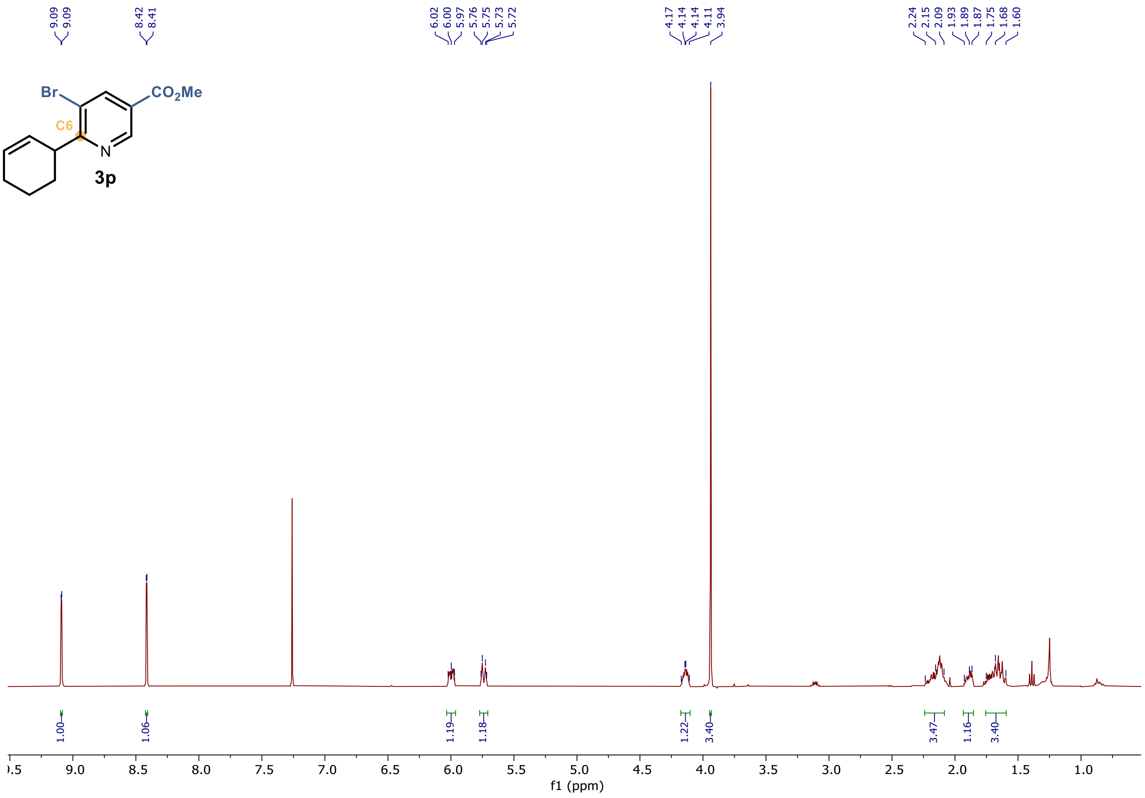


$^{13}$C NMR (101 MHz, CDCl$_3$) of **3p**

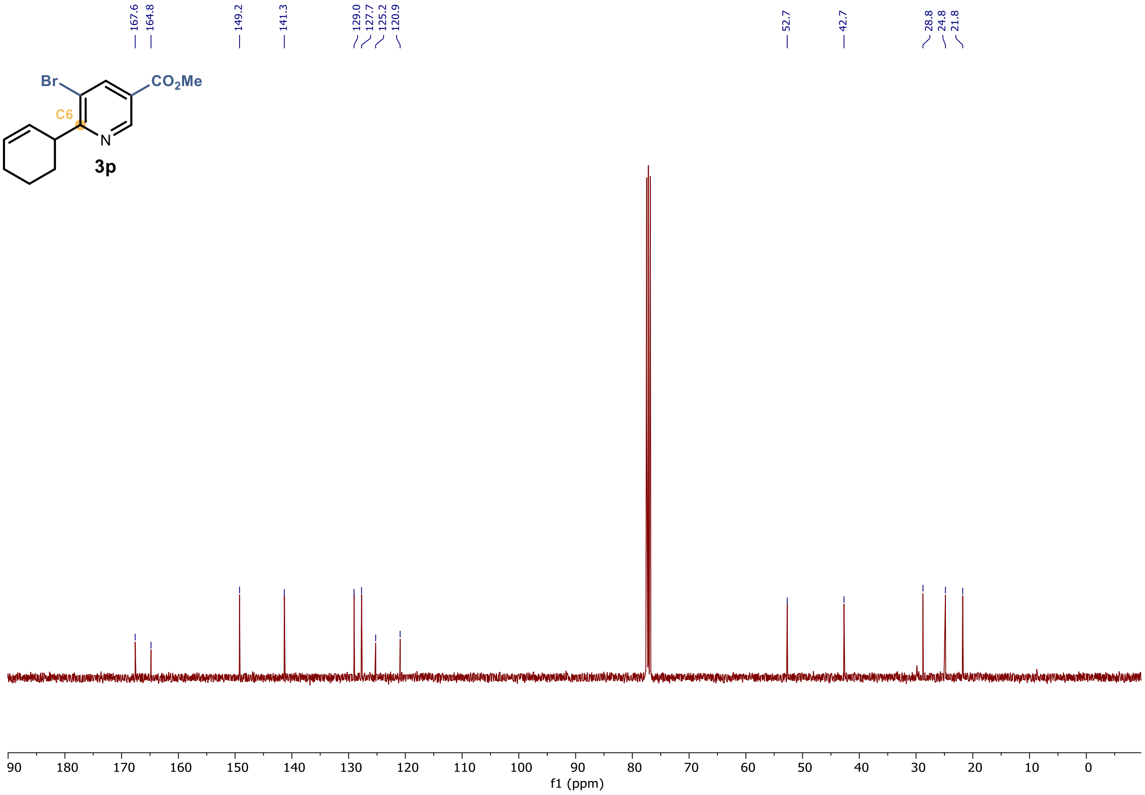

## $^{1}$H NMR (500 MHz, $CDCl_3$) of **3q**

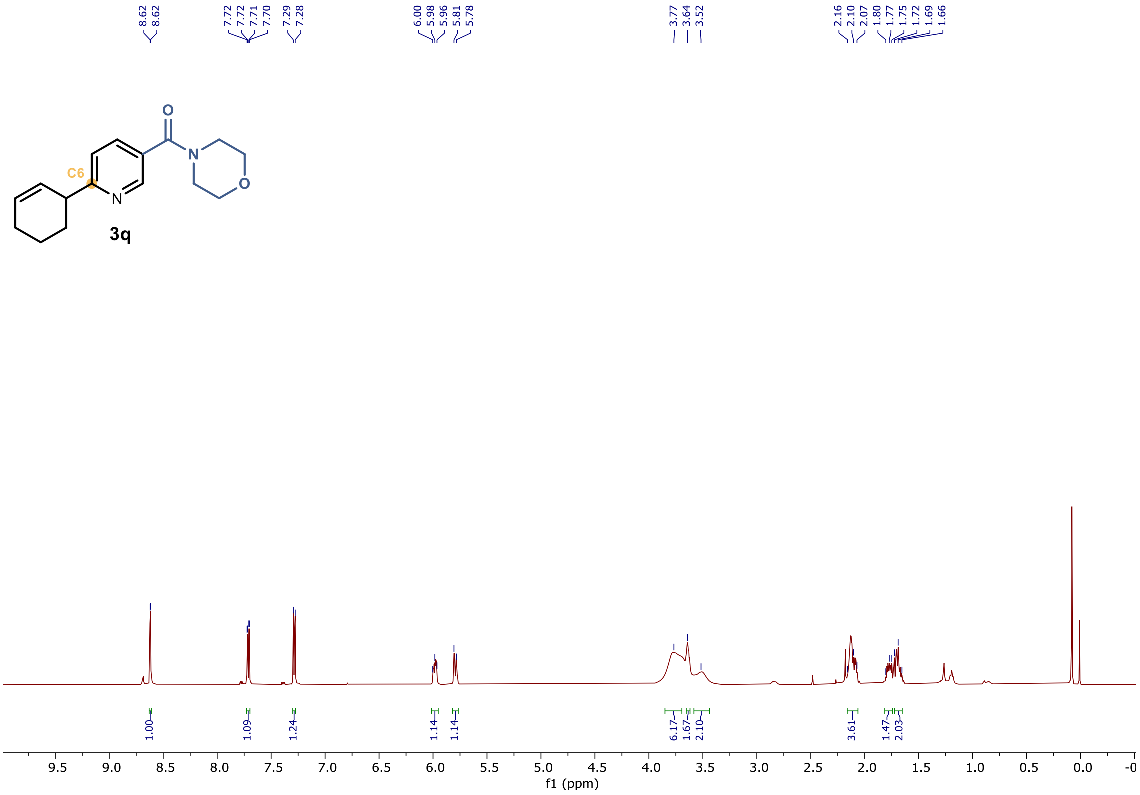


## $^{13}$C NMR (126 MHz, $CDCl_3$) of **3q**

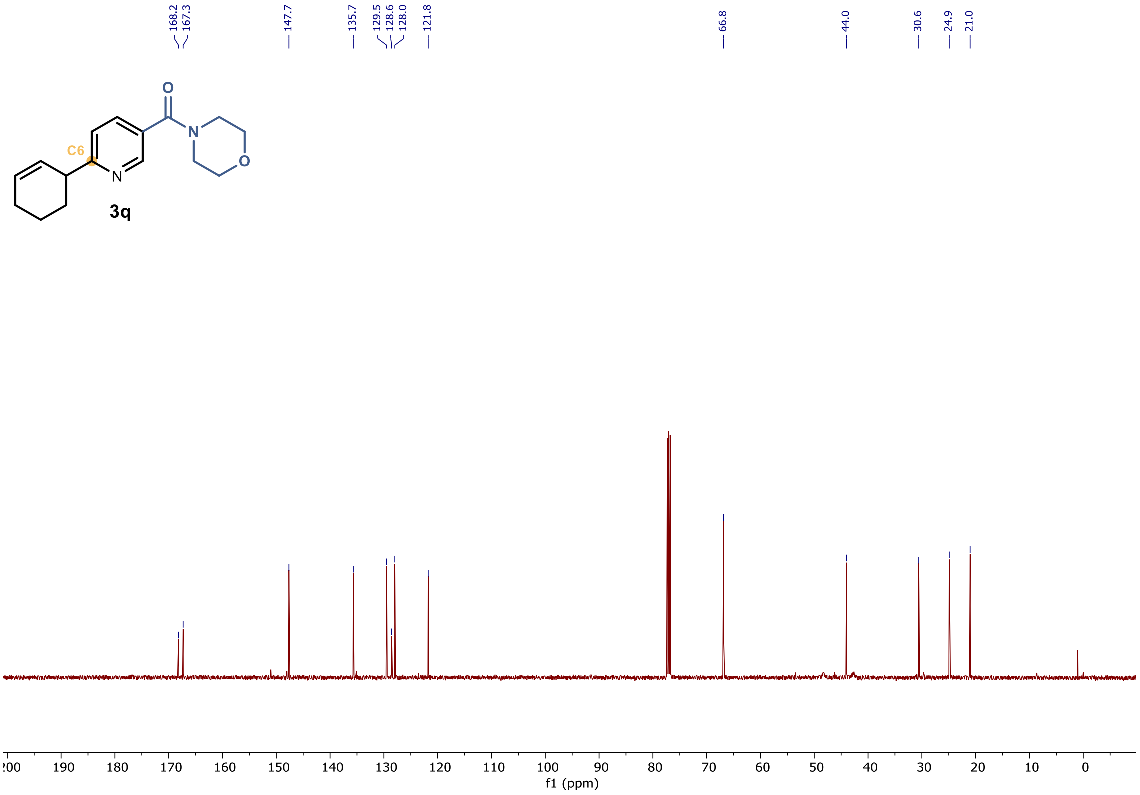

$^{1}$H NMR (400 MHz, $CDCl_3$) of **3r**

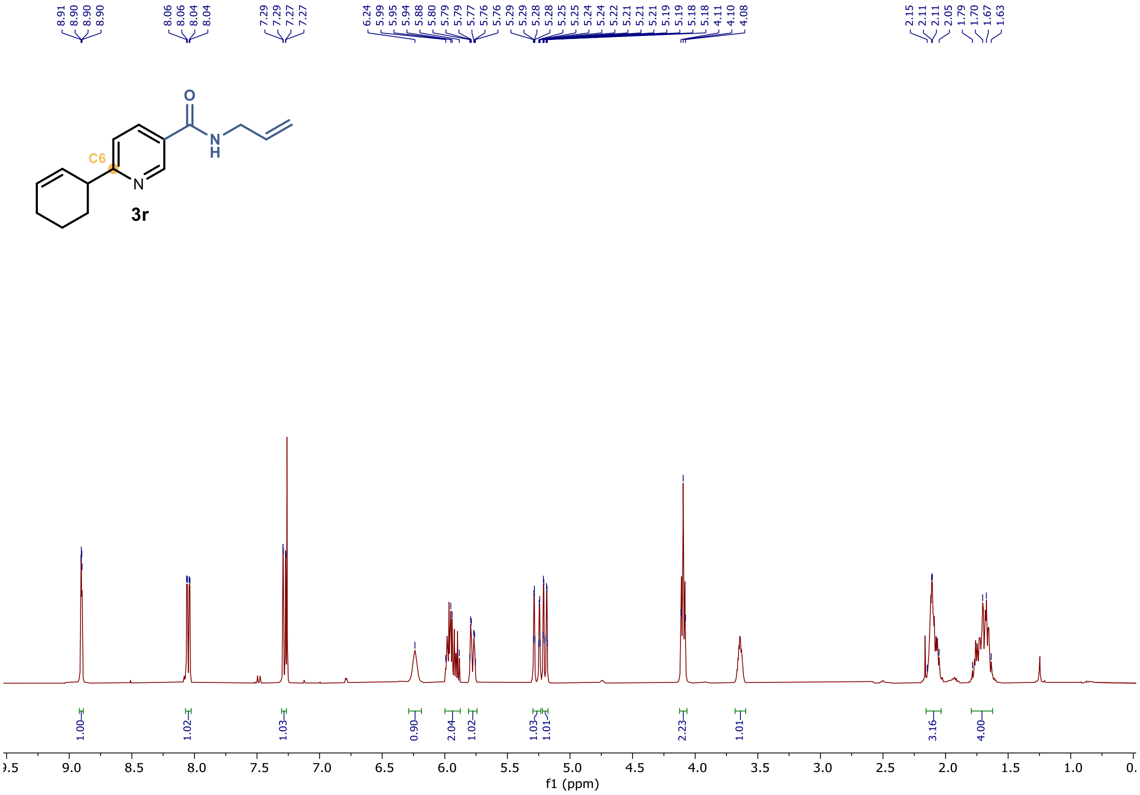


$^{13}$C NMR (101 MHz, $CDCl_3$) of **3r**

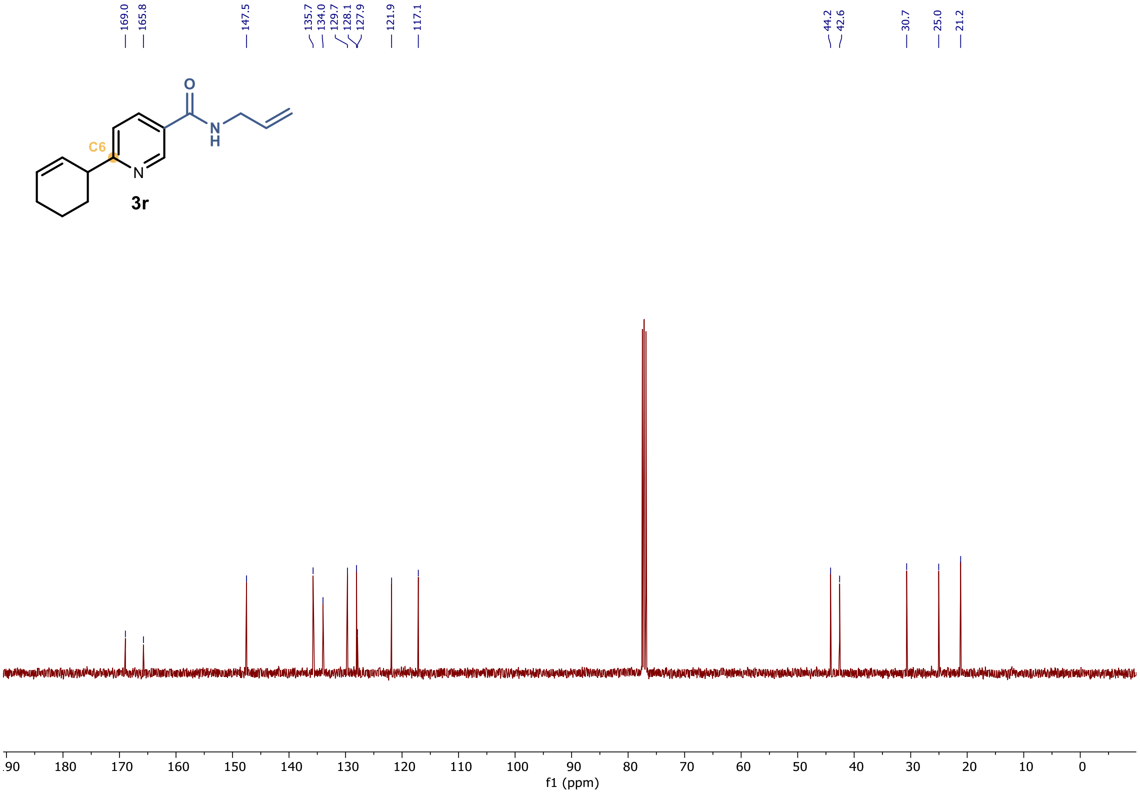

$^{1}$H NMR (400 MHz, $CDCl_3$) of **3s**

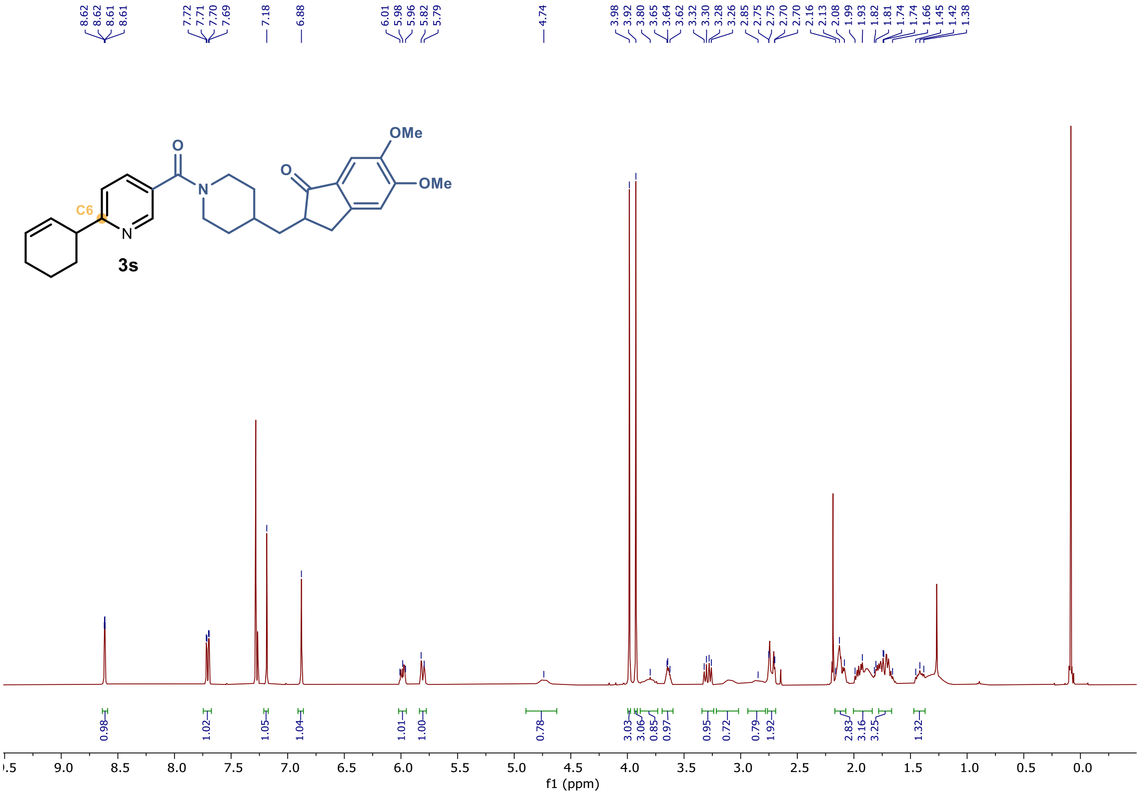

$^{13}$C NMR (101 MHz, $CDCl_3$) of **3s**

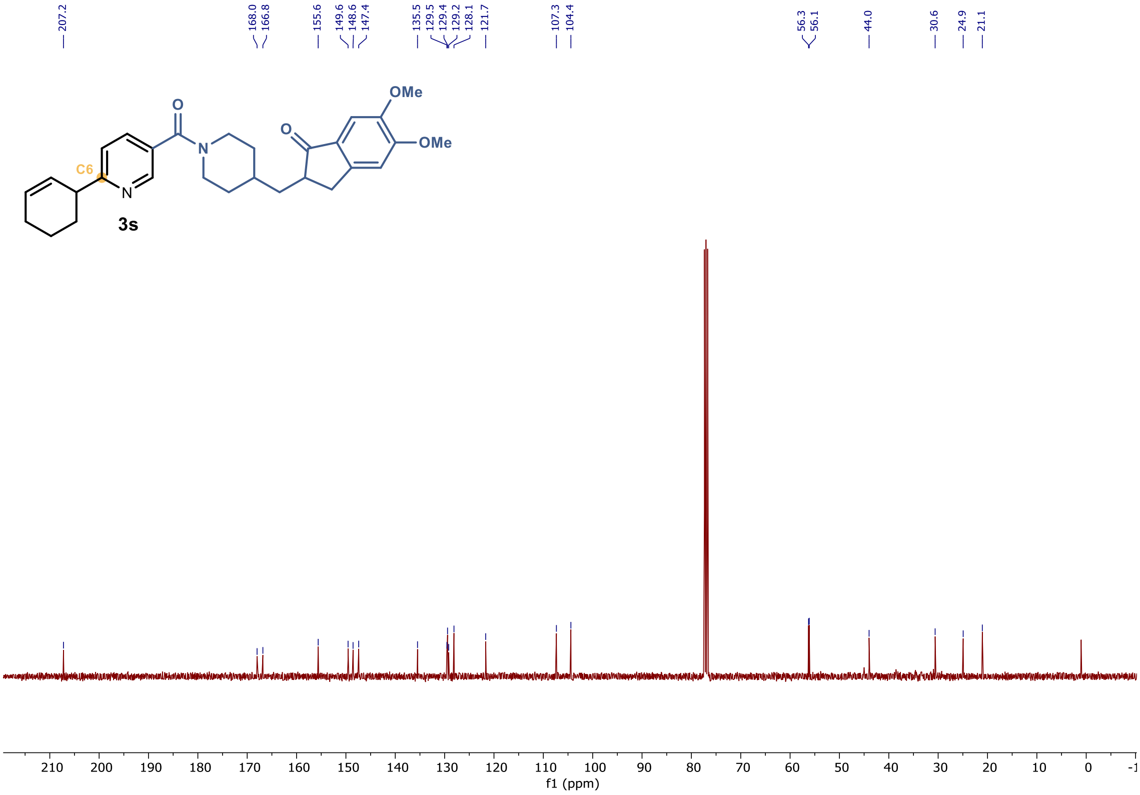

$^{1}$H NMR (400 MHz, $CDCl_3$) of **3t**

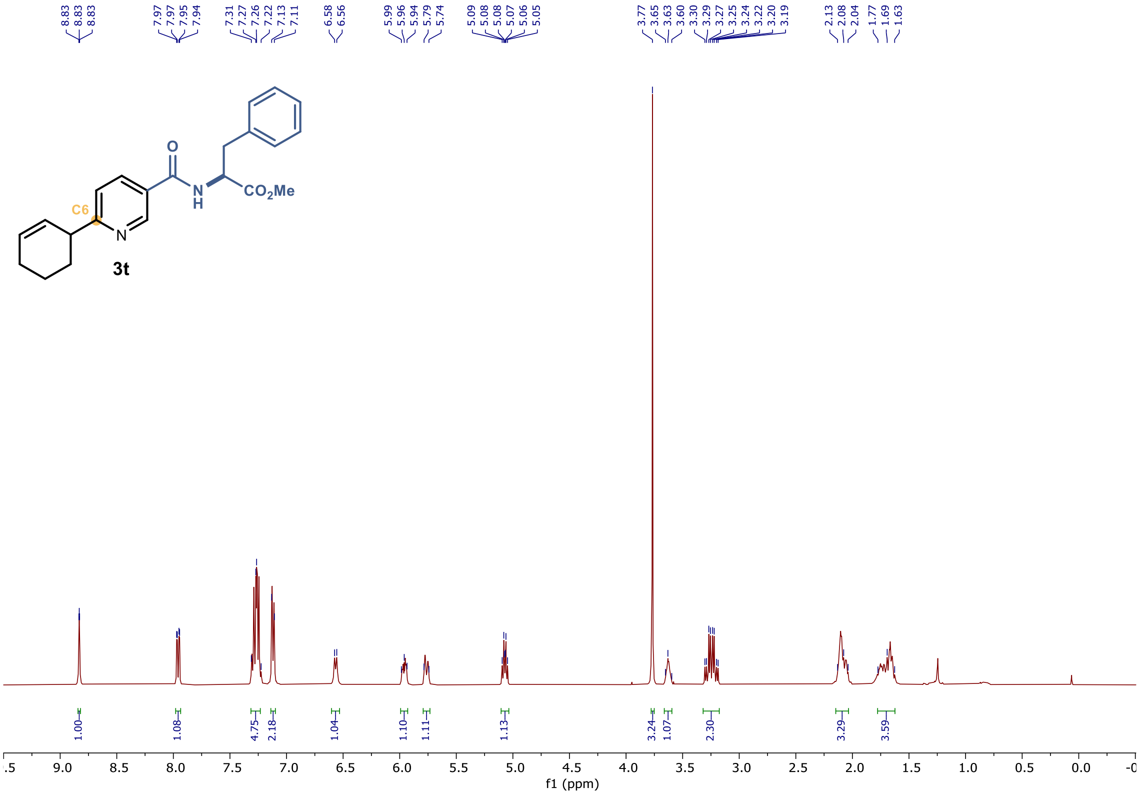

$^{13}$C NMR (101 MHz, $CDCl_3$) of **3t**

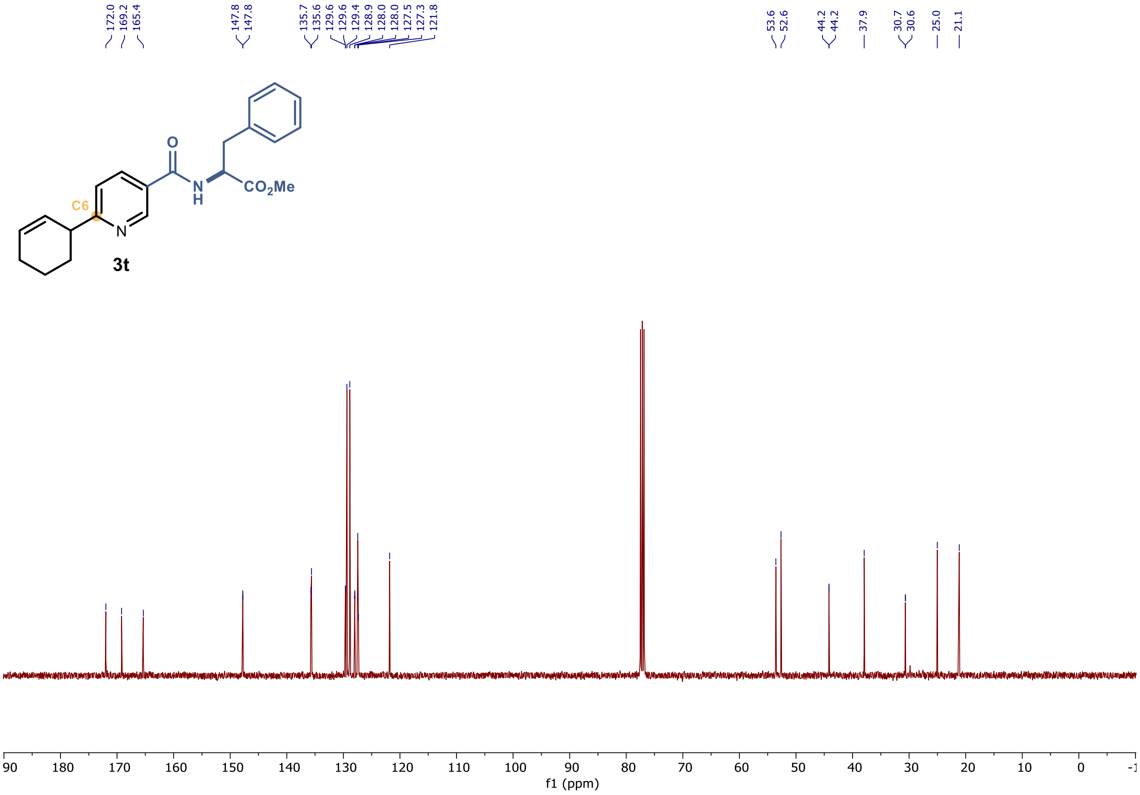

$^{1}$H NMR (400 MHz, $CDCl_3$) of **3u**

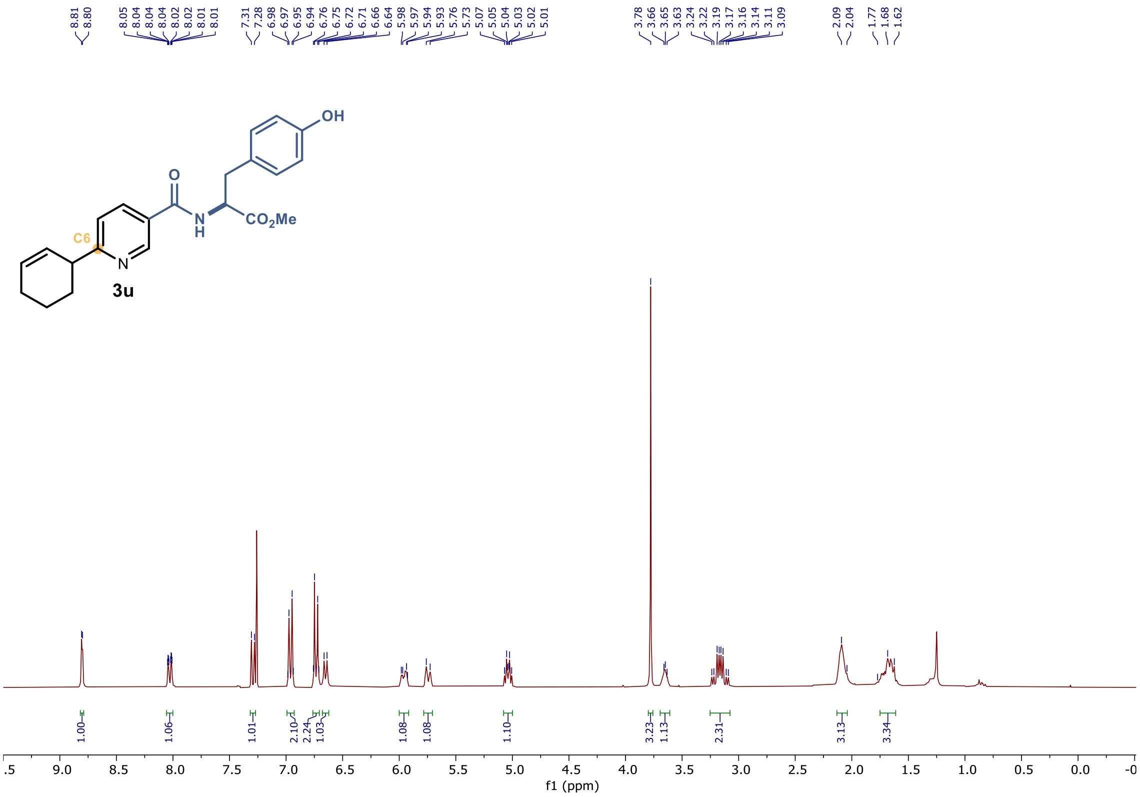

$^{13}$C NMR (101 MHz, $CDCl_3$) of **3u**

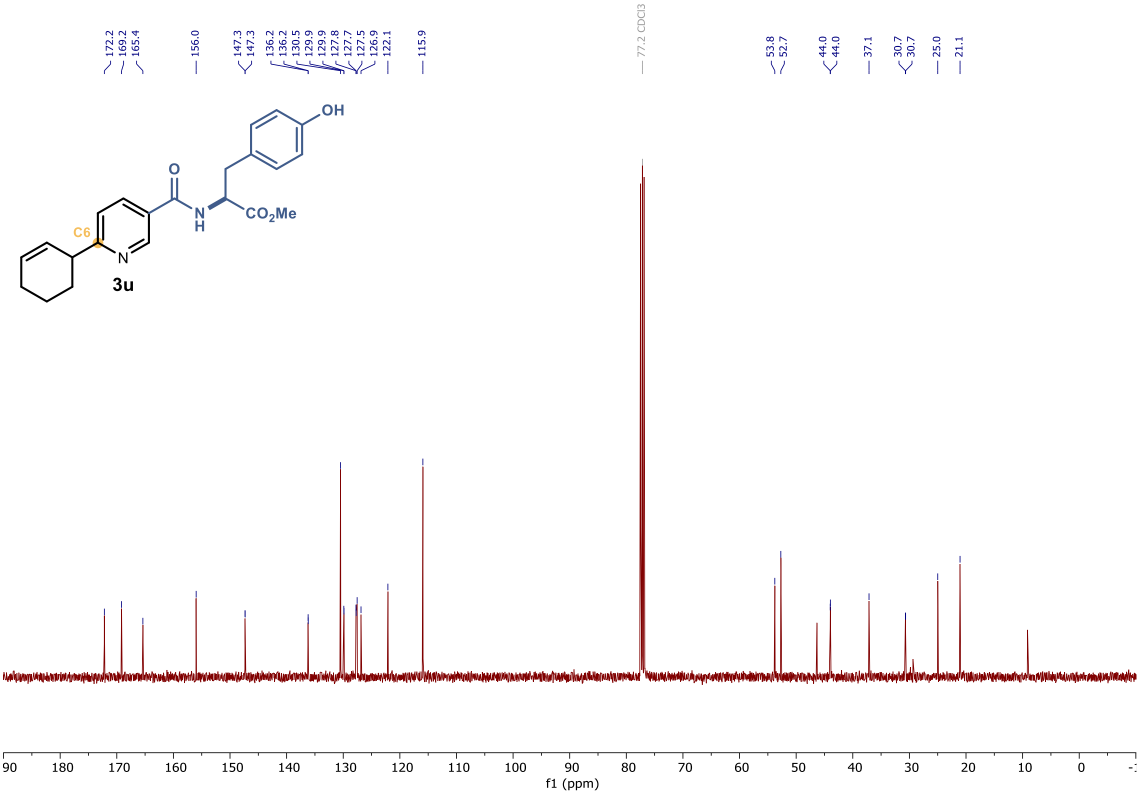

$^{1}$H NMR (400 MHz, $CDCl_3$) of **3v**

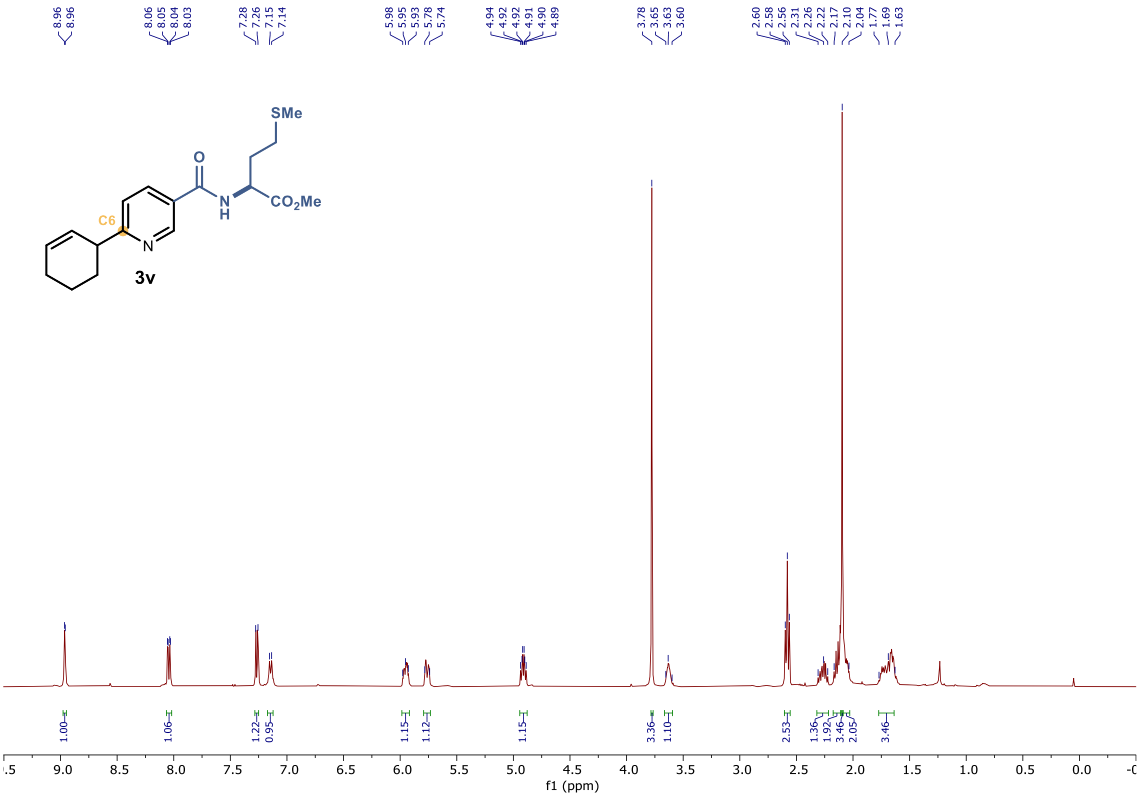

$^{13}$C NMR (101 MHz, $CDCl_3$) of **3v**

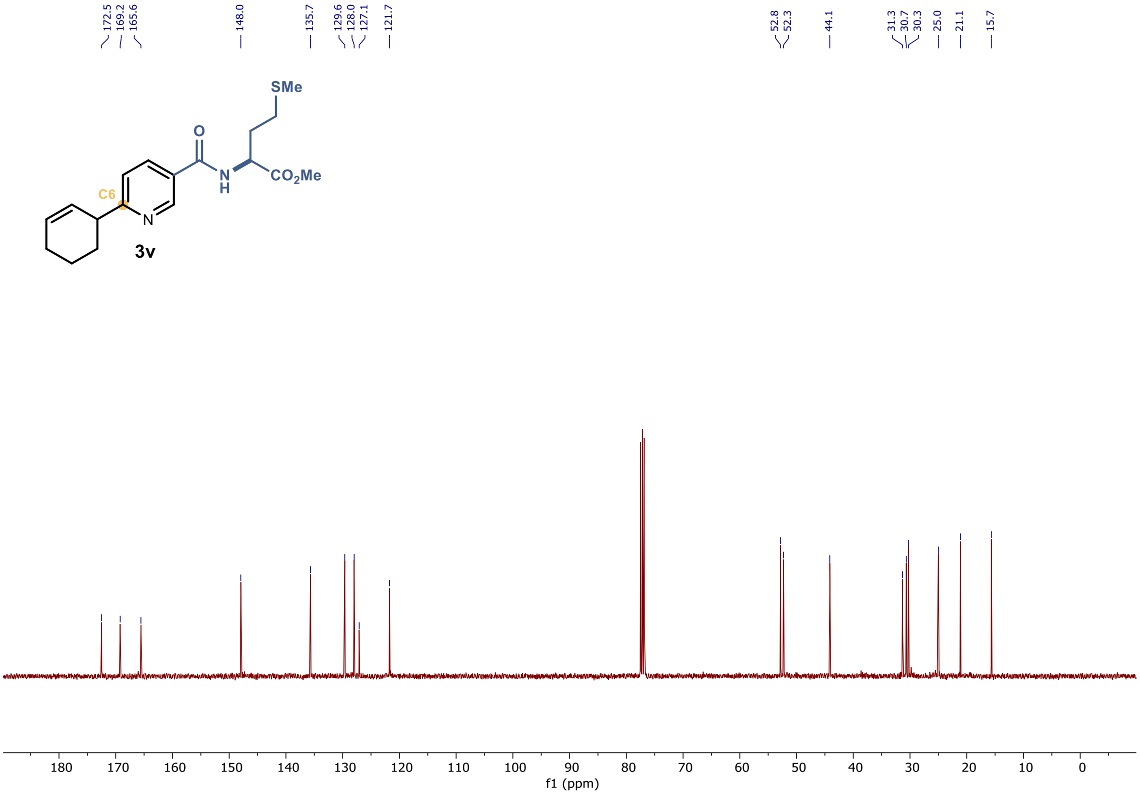

$^{1}$H NMR (400 MHz, $CDCl_3$) of **3w**

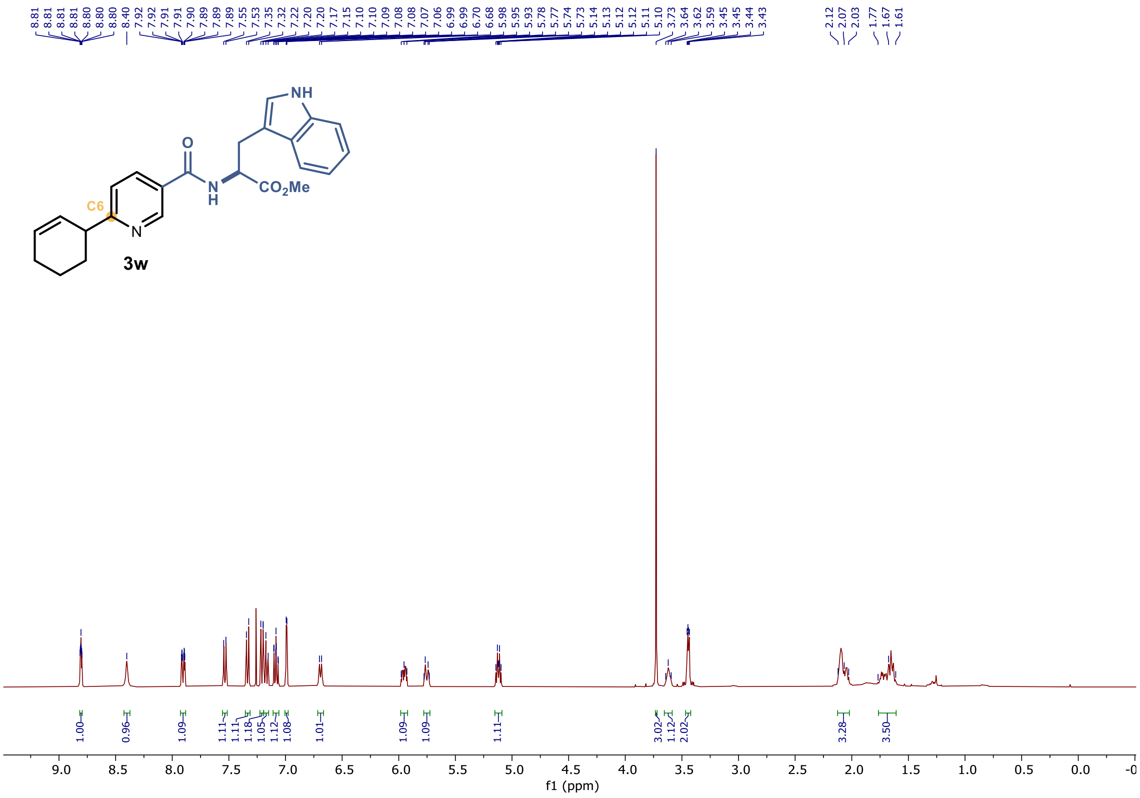

$^{13}$C NMR (101 MHz, $CDCl_3$) of **3w**

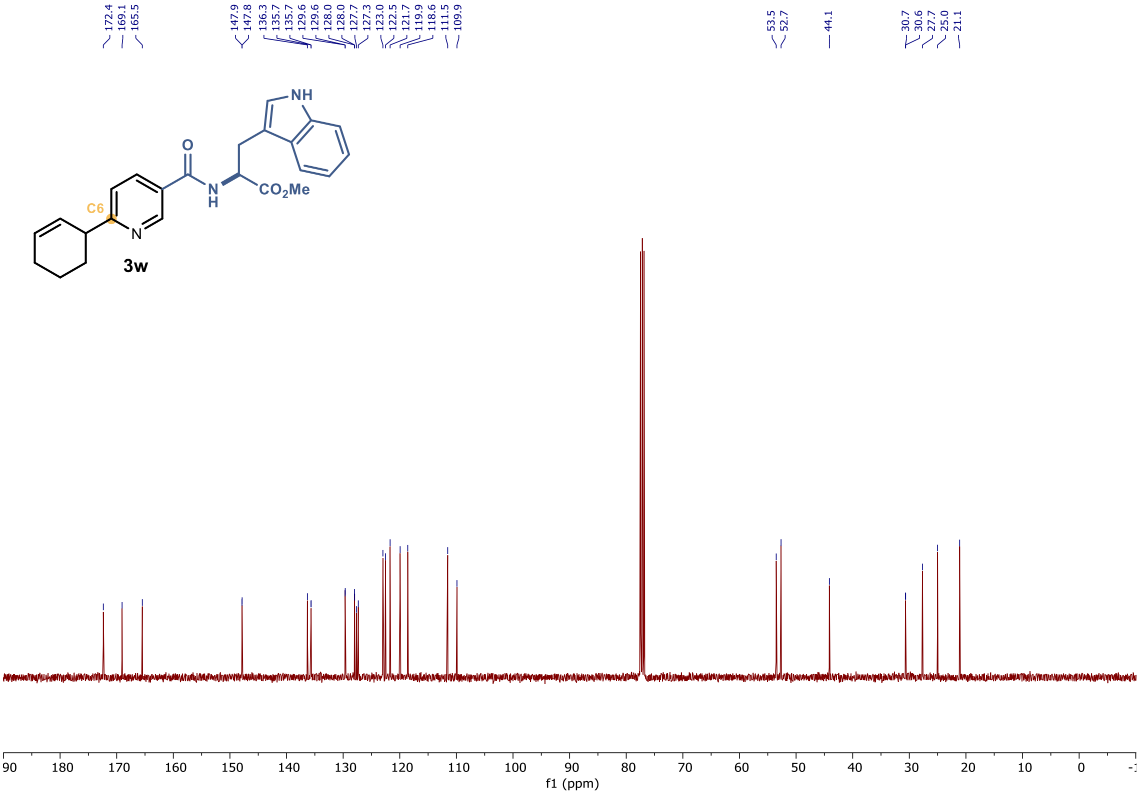

$^1$H NMR (500 MHz, $CDCl_3$) of **3x**

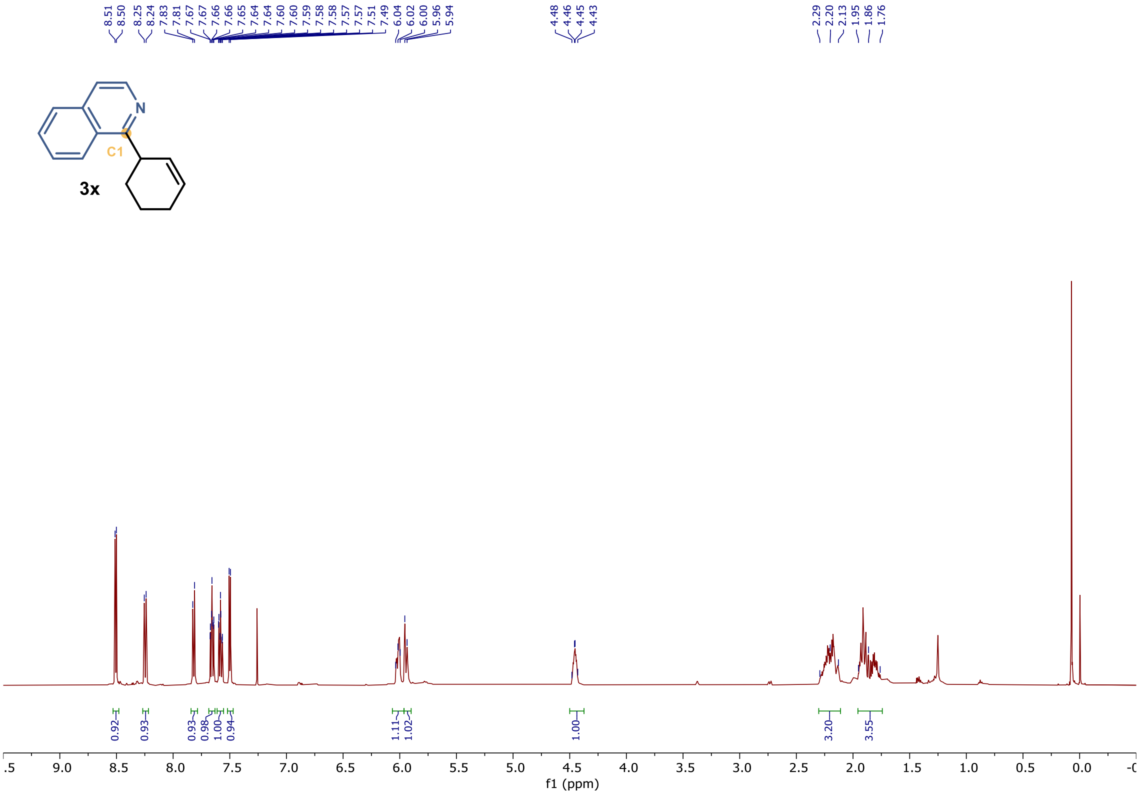

$^{13}$C NMR (126 MHz, $CDCl_3$) of **3x**

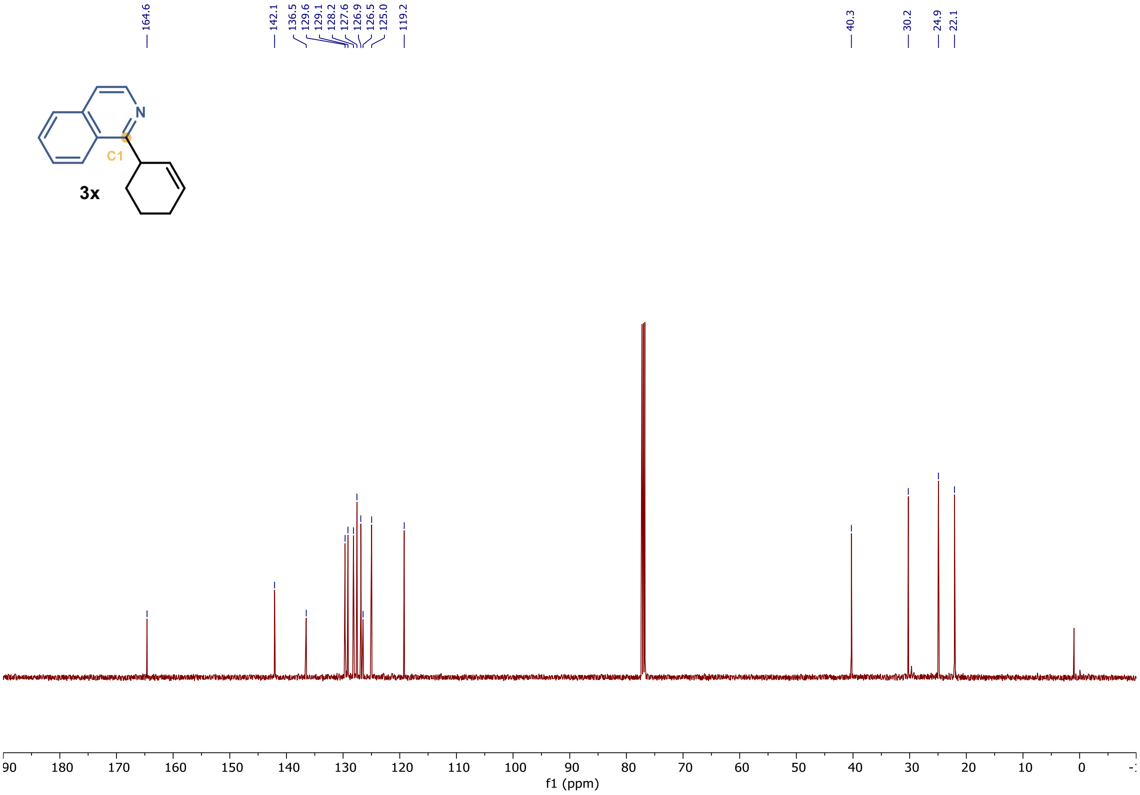

$^{1}$H NMR (400 MHz, $CDCl_3$) of **3y**

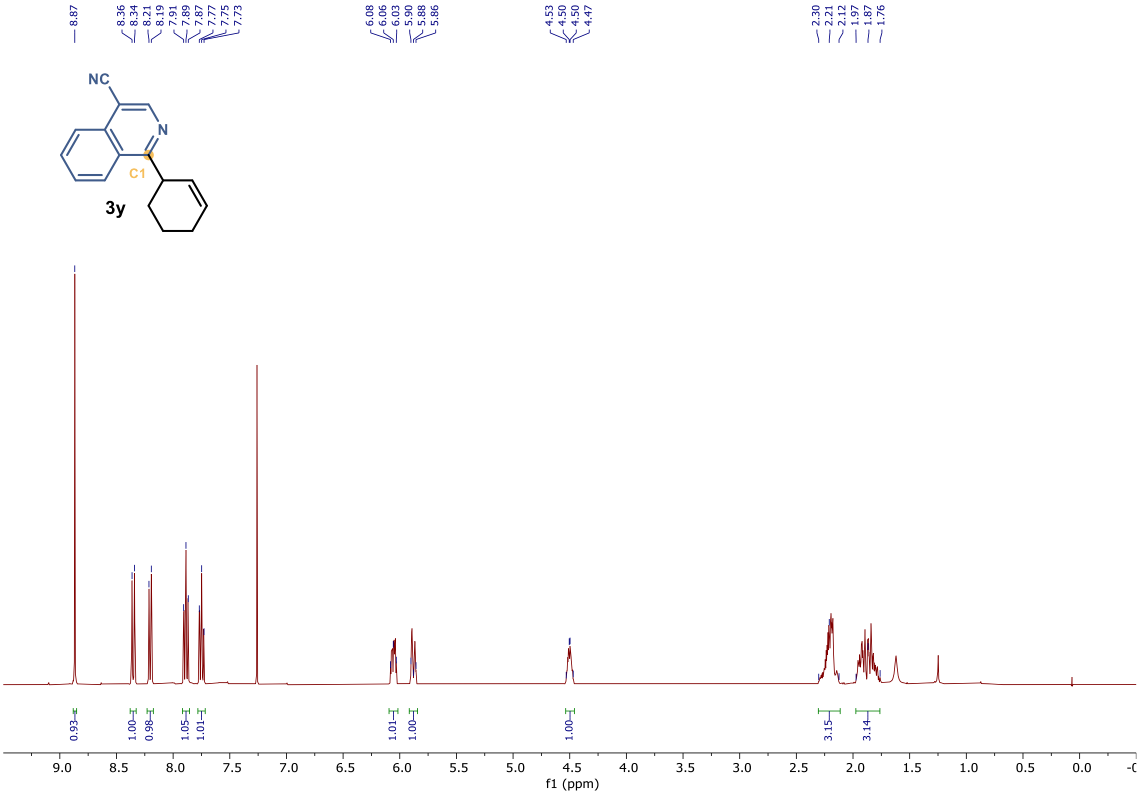


$^{13}$C NMR (101 MHz, $CDCl_3$) of **3y**

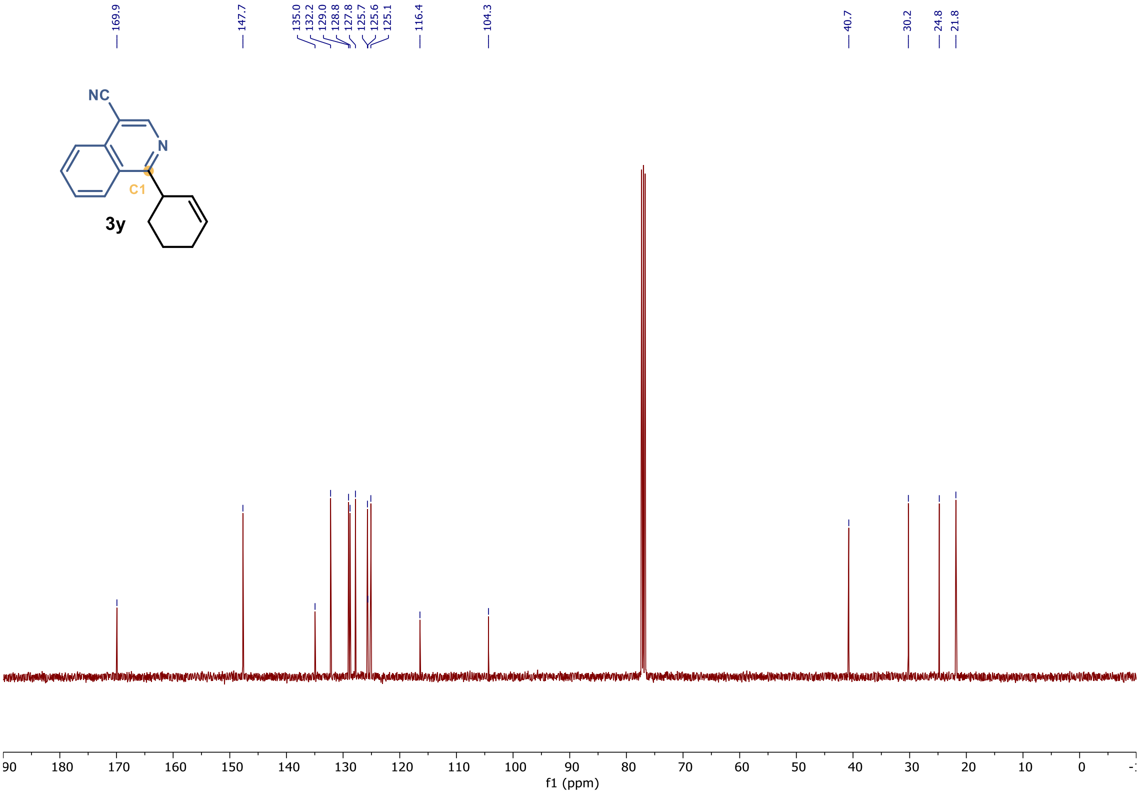

$^{1}$H NMR (400 MHz, $CDCl_3$) of **3z**

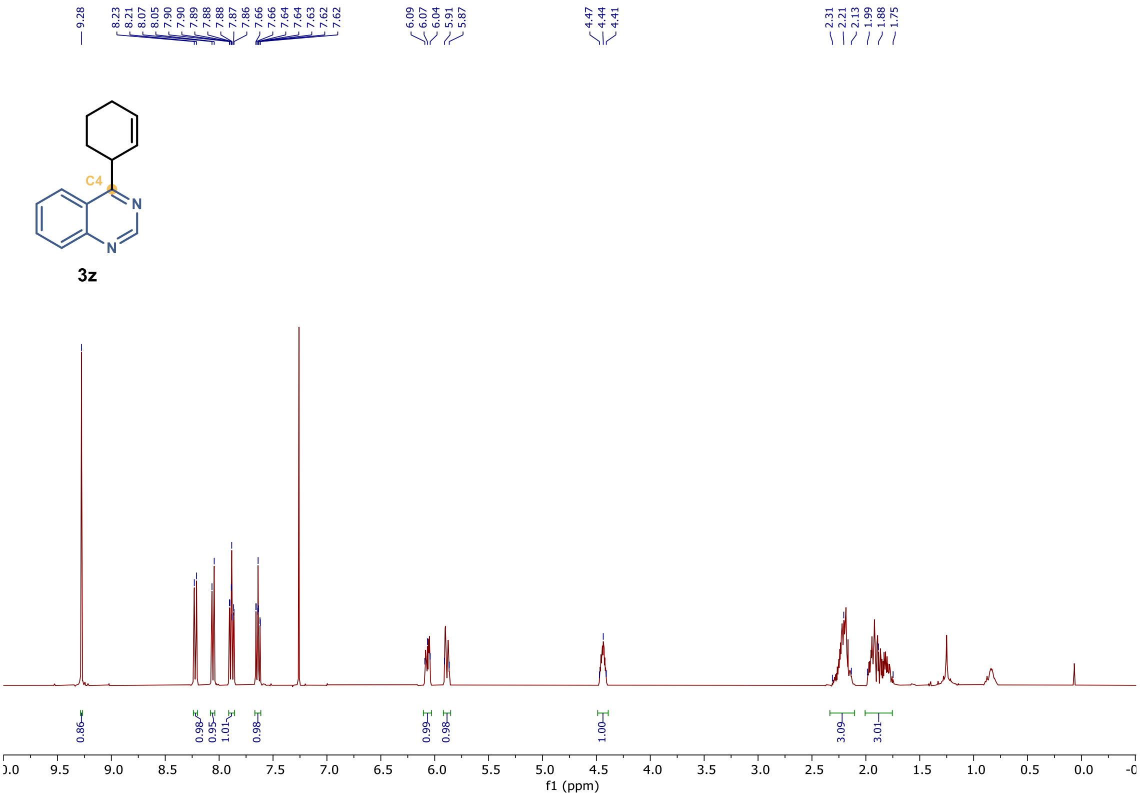

$^{13}$C NMR (101 MHz, $CDCl_3$) of **3z**

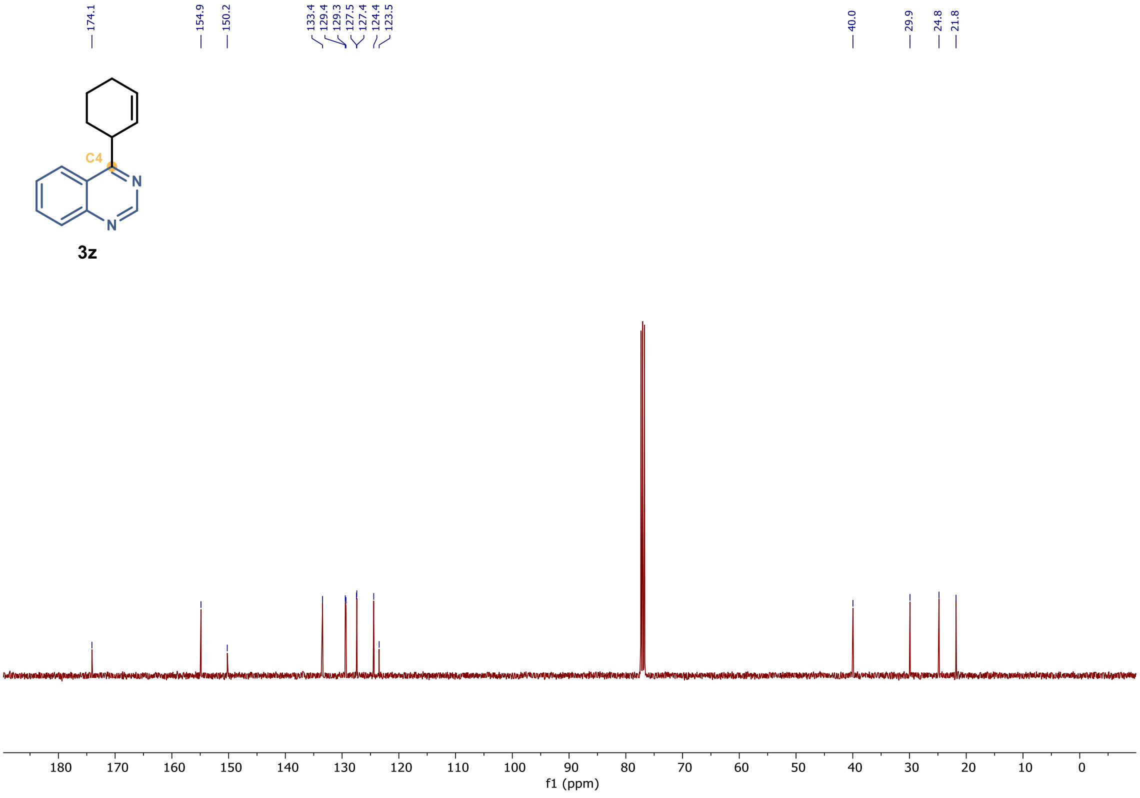

## $^{1}H$ NMR (500 MHz, $CDCl_3$) of **3aa**

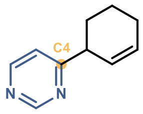


**3aa**

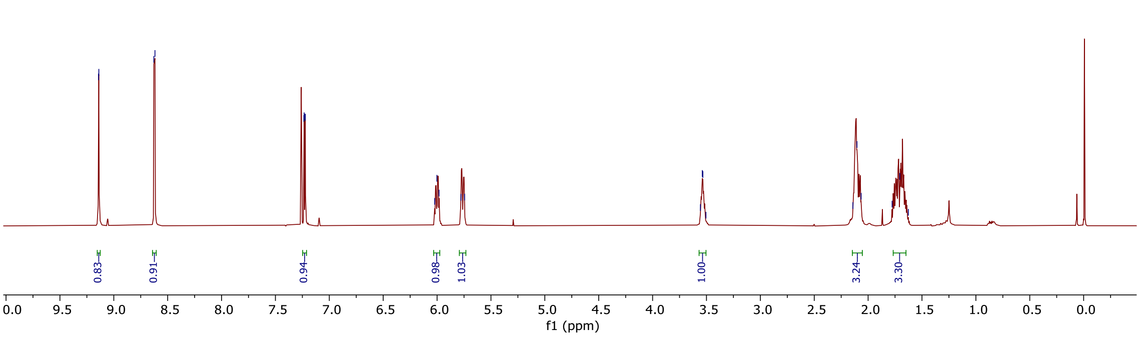


## $^{13}C$ NMR (126 MHz, $CDCl_3$) of **3aa**

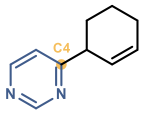


**3aa**

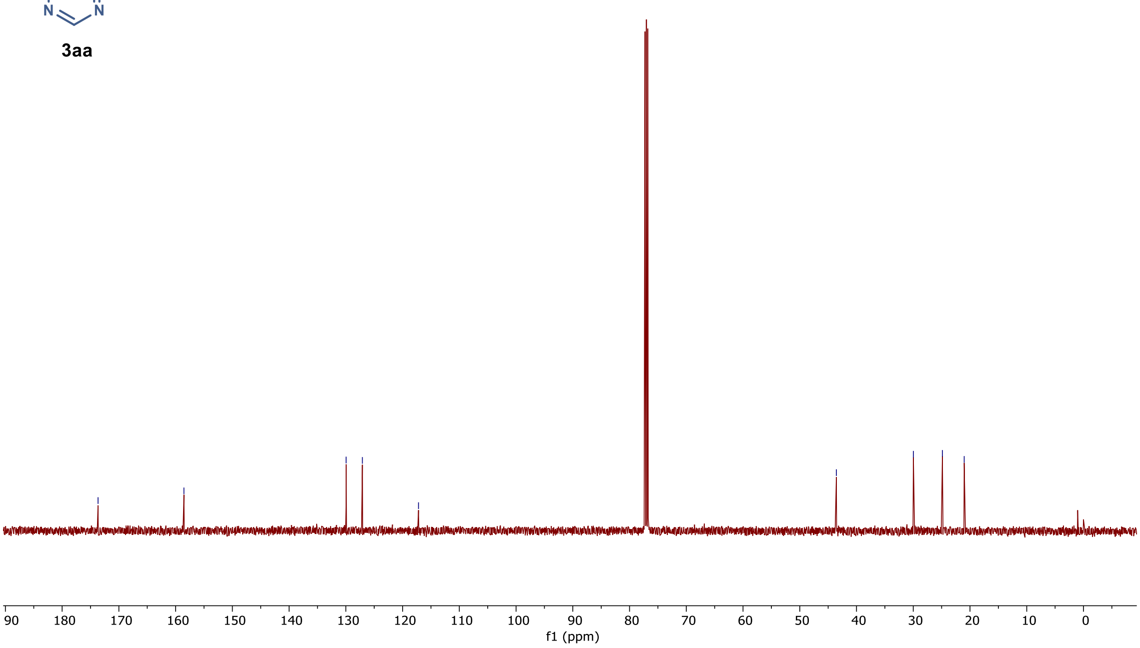

$^{1}$H NMR (500 MHz, $CDCl_3$) of **3aa’**

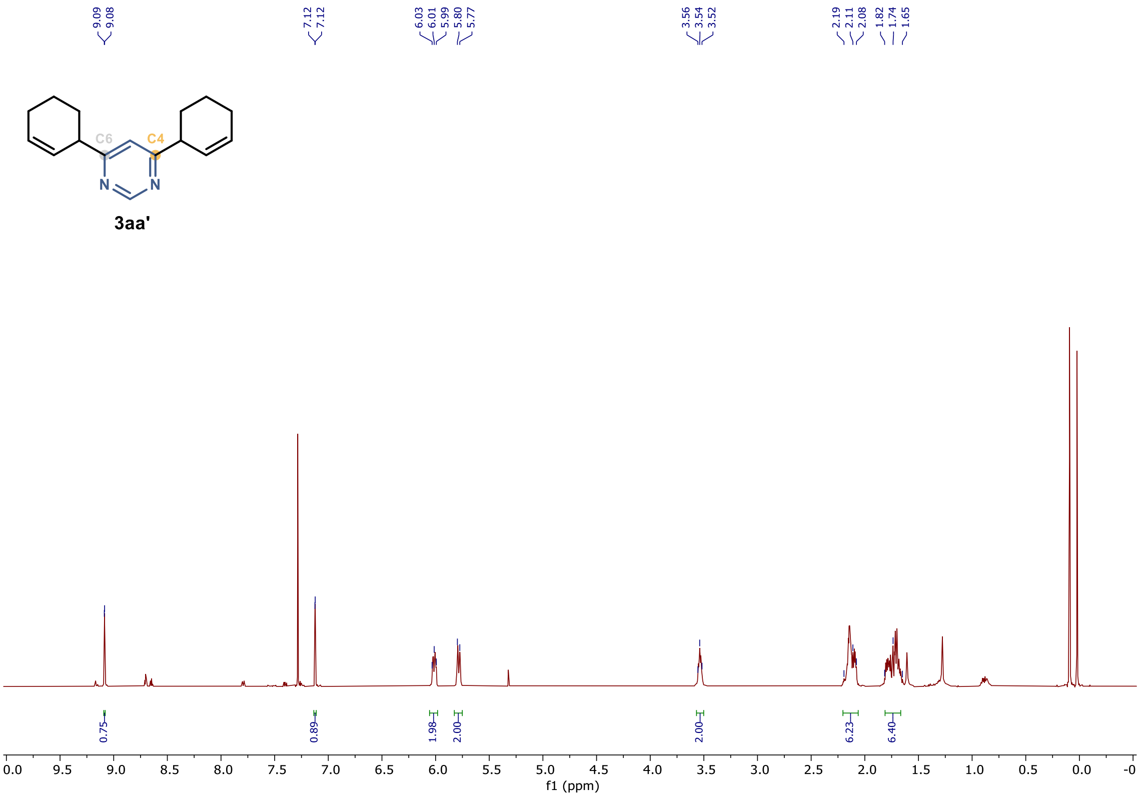


$^{13}$C NMR (126 MHz, $CDCl_3$) of **3aa’**

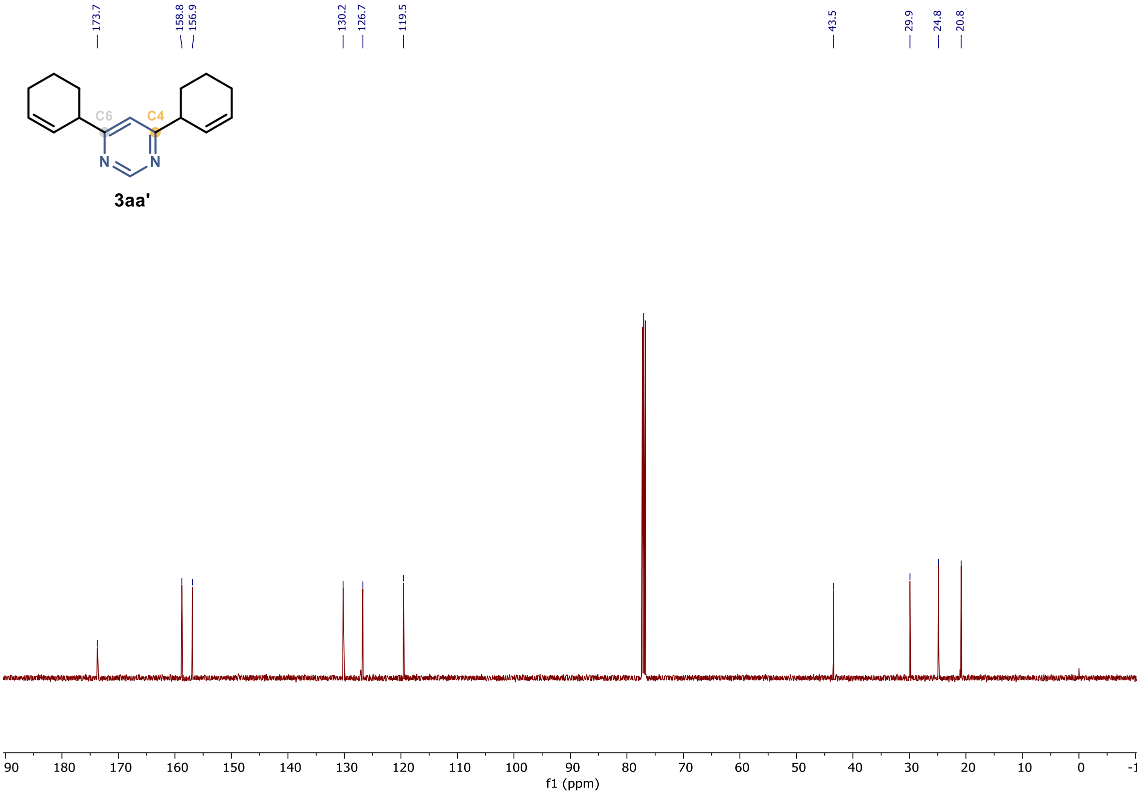

$^{1}$H NMR (400 MHz, $CDCl_3$) of **3bb**

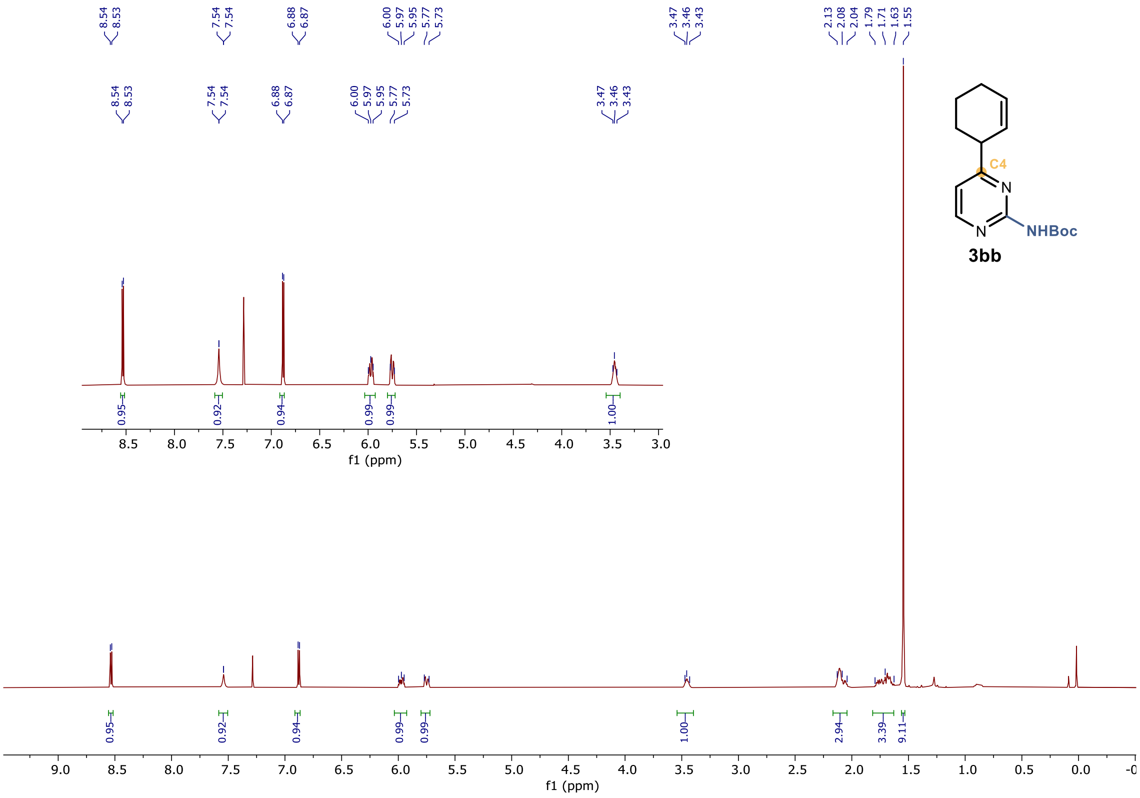


$^{13}$C NMR (101 MHz, $CDCl_3$) of **3bb**

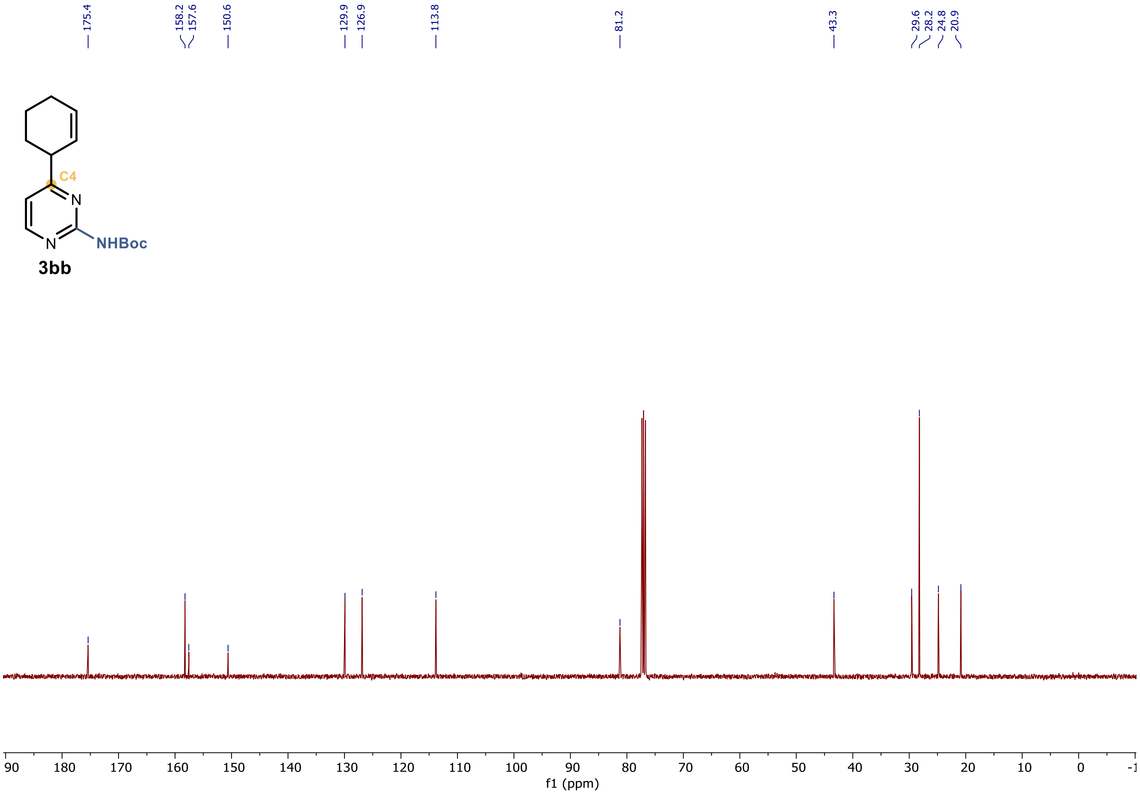

$^{1}$H NMR (400 MHz, $CDCl_3$) of **3cc**

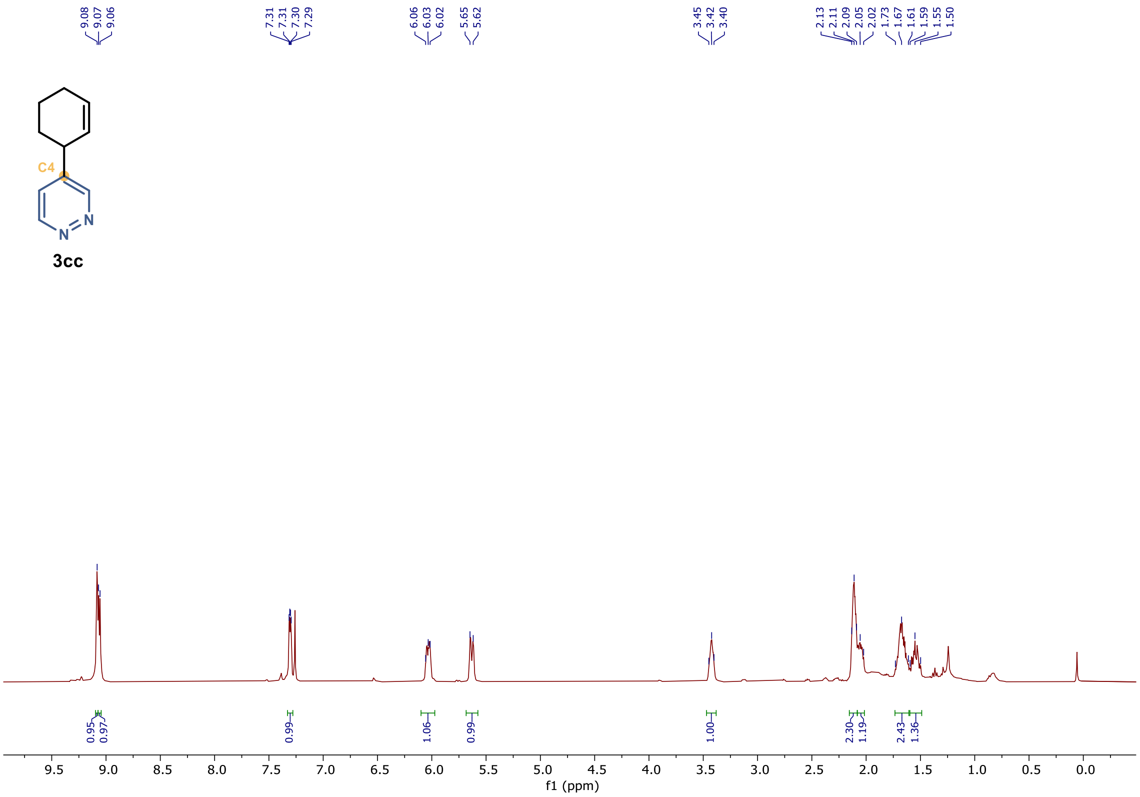

$^{13}$C NMR (101 MHz, $CDCl_3$) of **3cc**

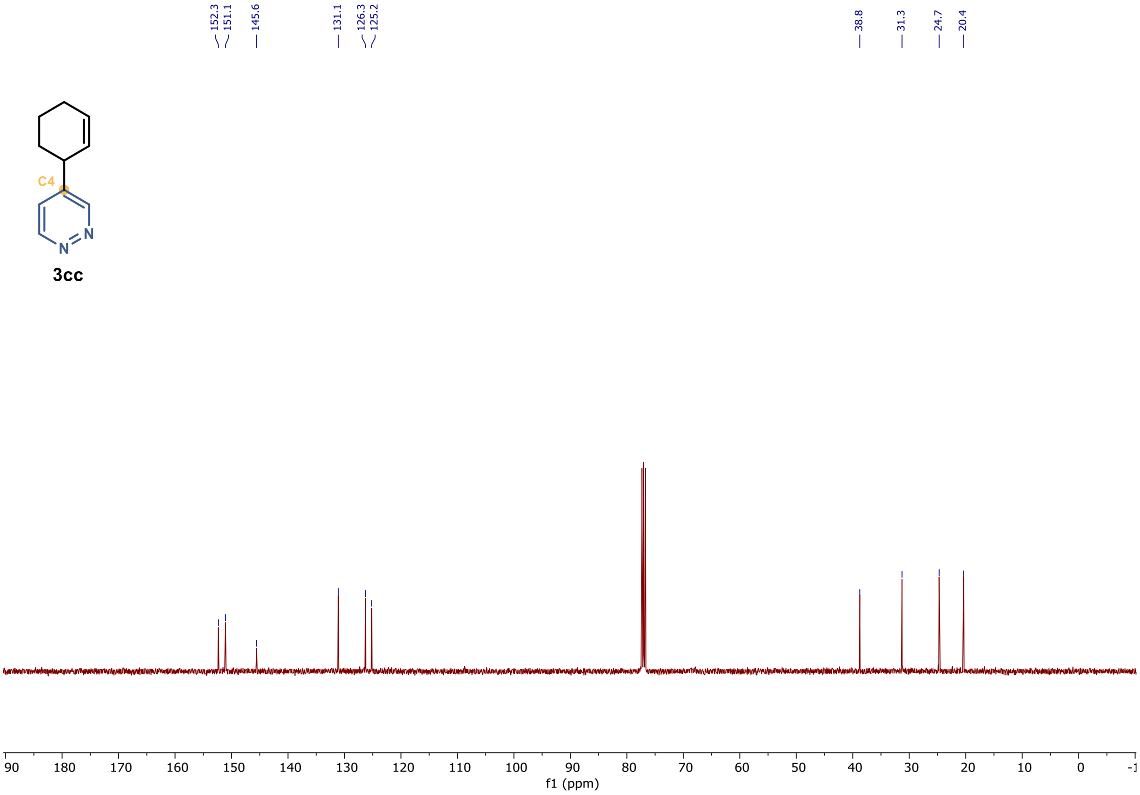

$^{1}$H NMR (400 MHz, $CDCl_3$) of **3dd**

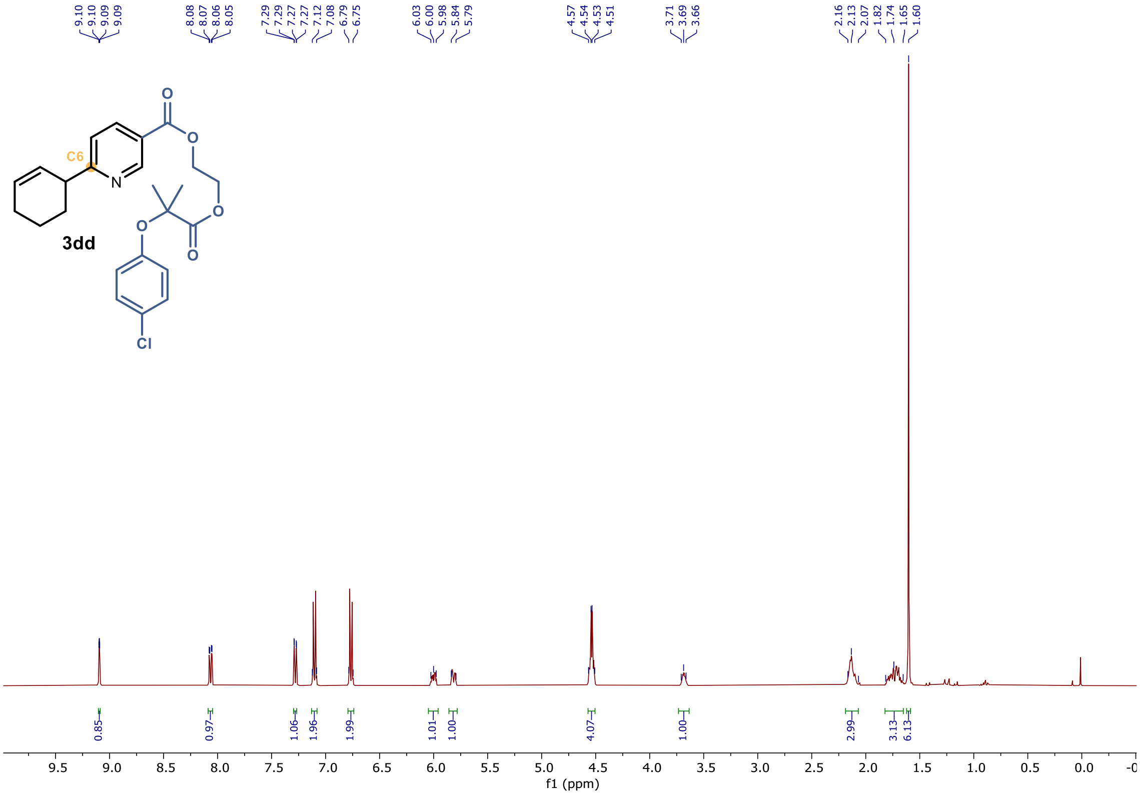


$^{13}$C NMR (101 MHz, $CDCl_3$) of **3dd**

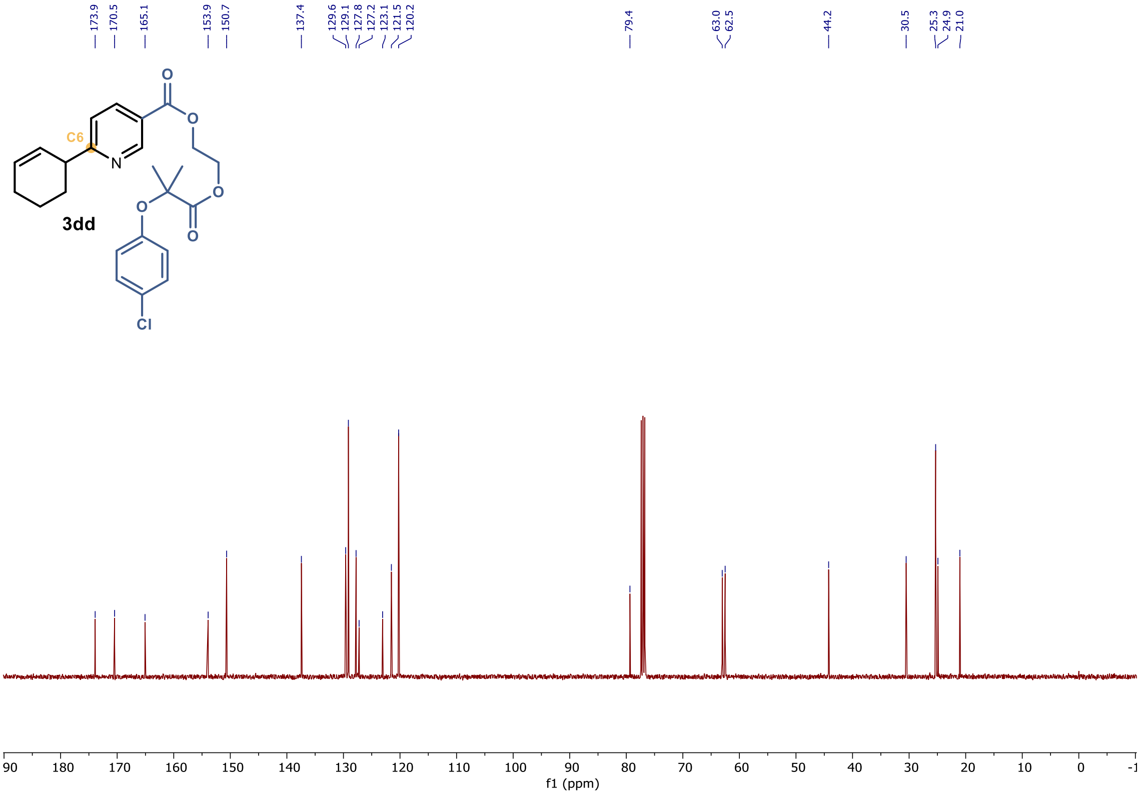

## $^{1}$H NMR (500 MHz, $CDCl_3$) of **3ee**

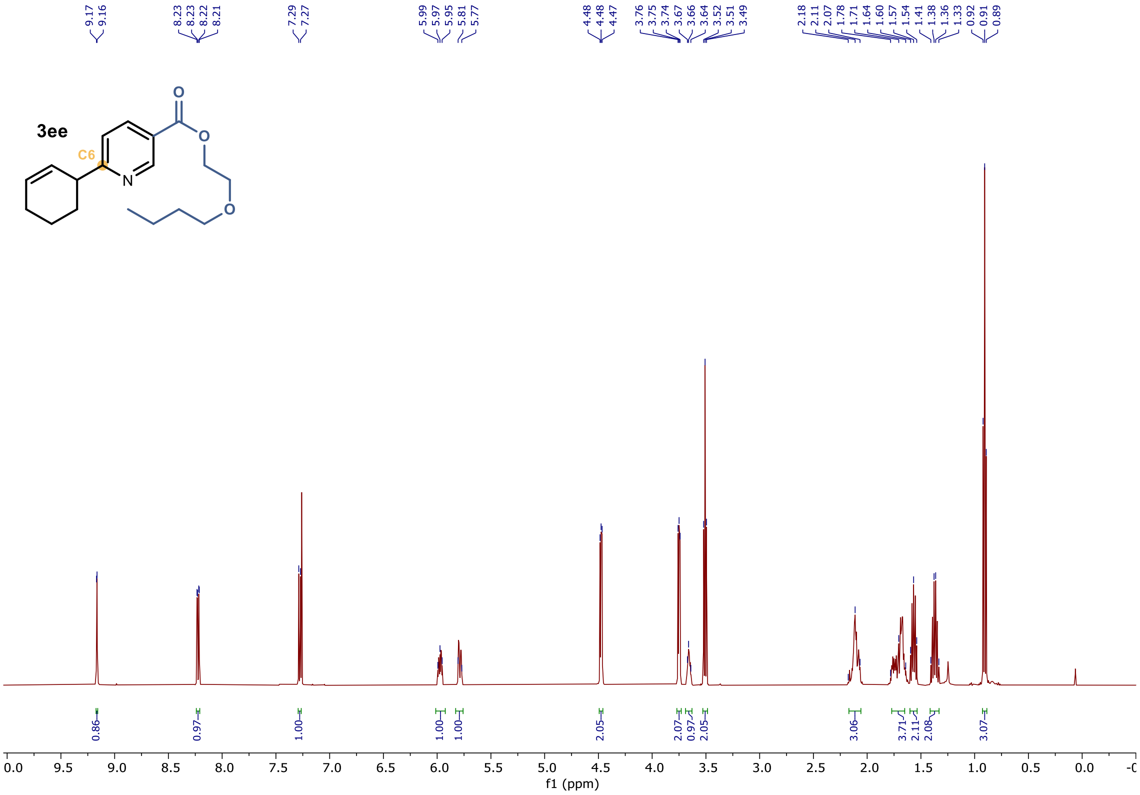


## $^{13}$C NMR (126 MHz, $CDCl_3$) of **3ee**

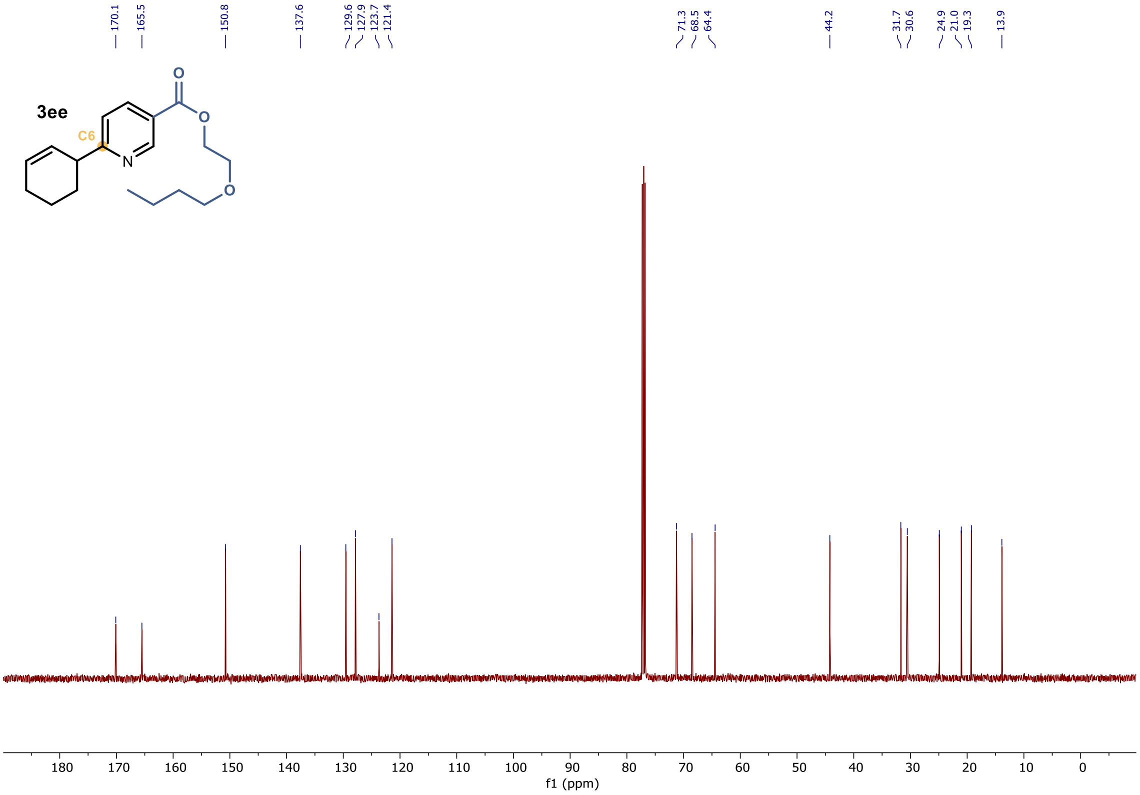

$^{1}$H NMR (400 MHz, Acetone) of **3ff**

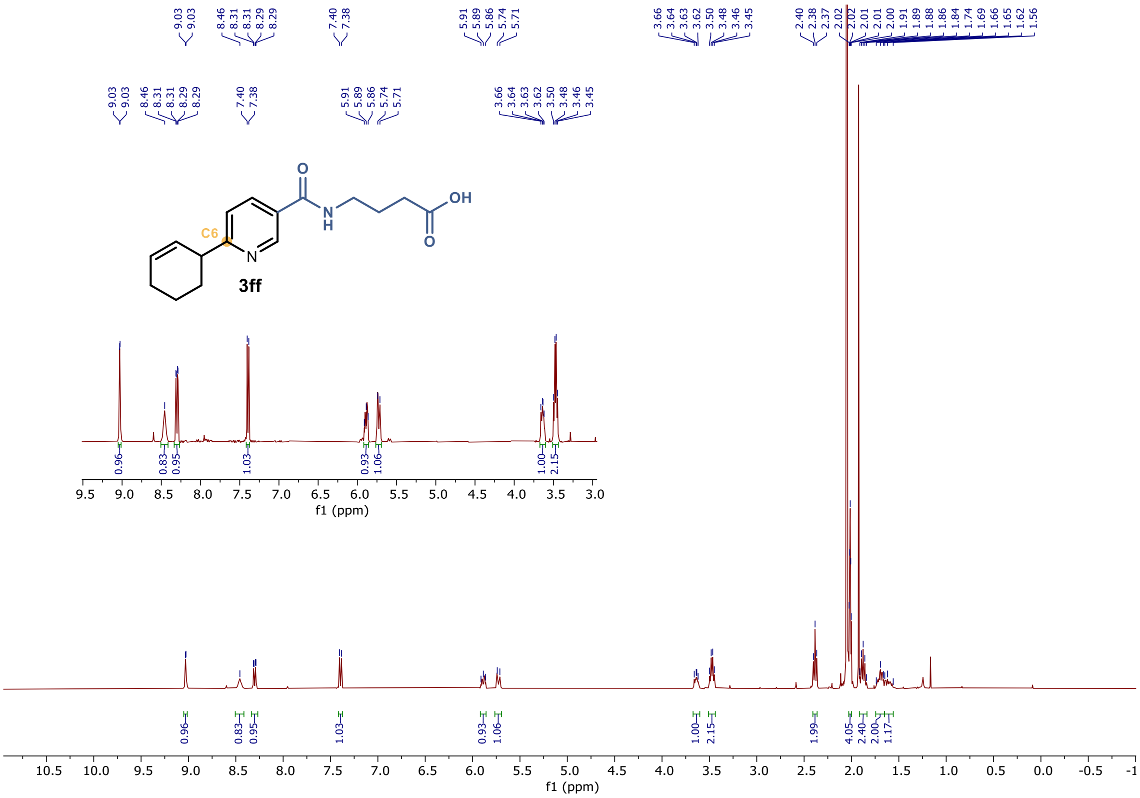

$^{13}$C NMR (101 MHz, Acetone) of **3ff**

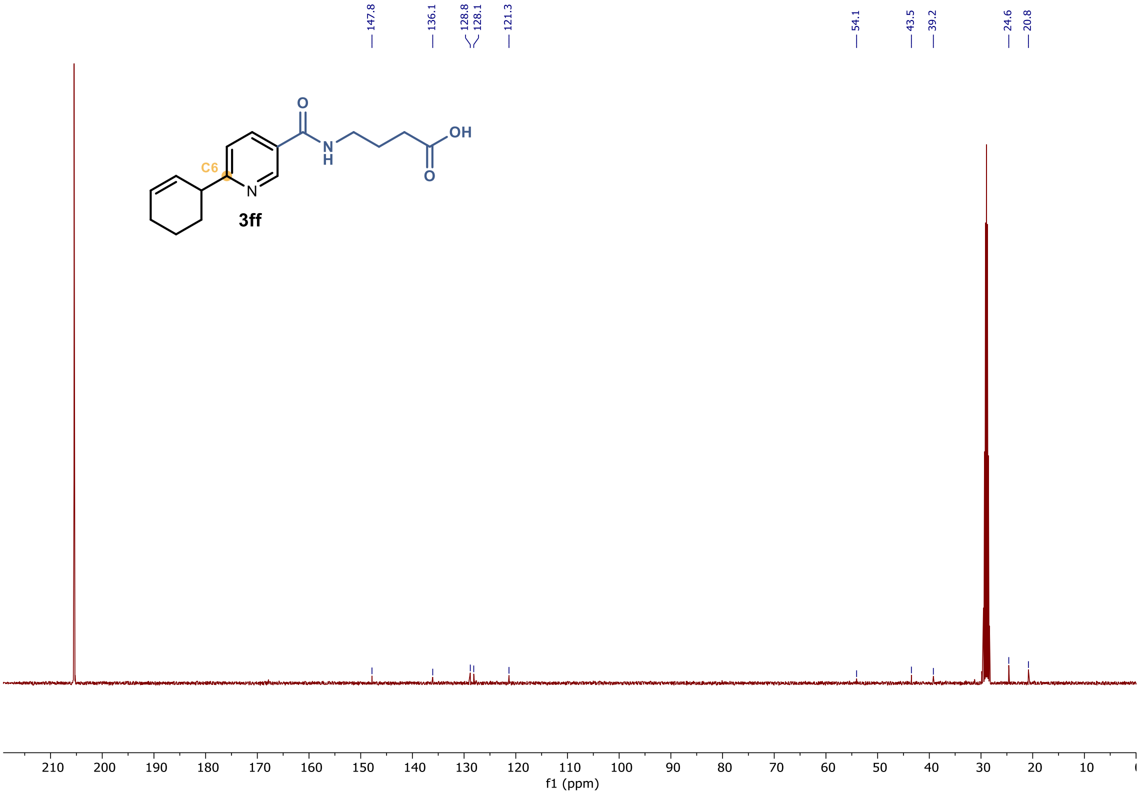

$^{1}$H NMR (400 MHz, $CDCl_3$) of **3gg**

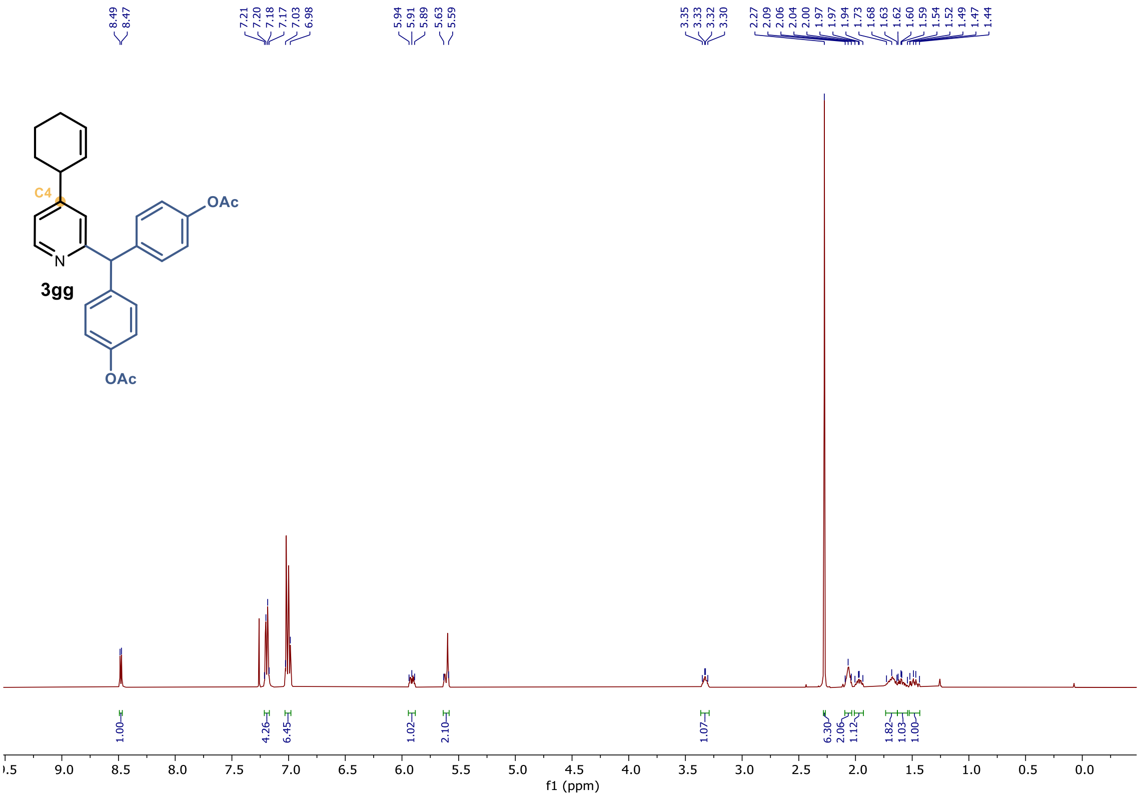


$^{13}$C NMR (101 MHz, $CDCl_3$) of **3gg**

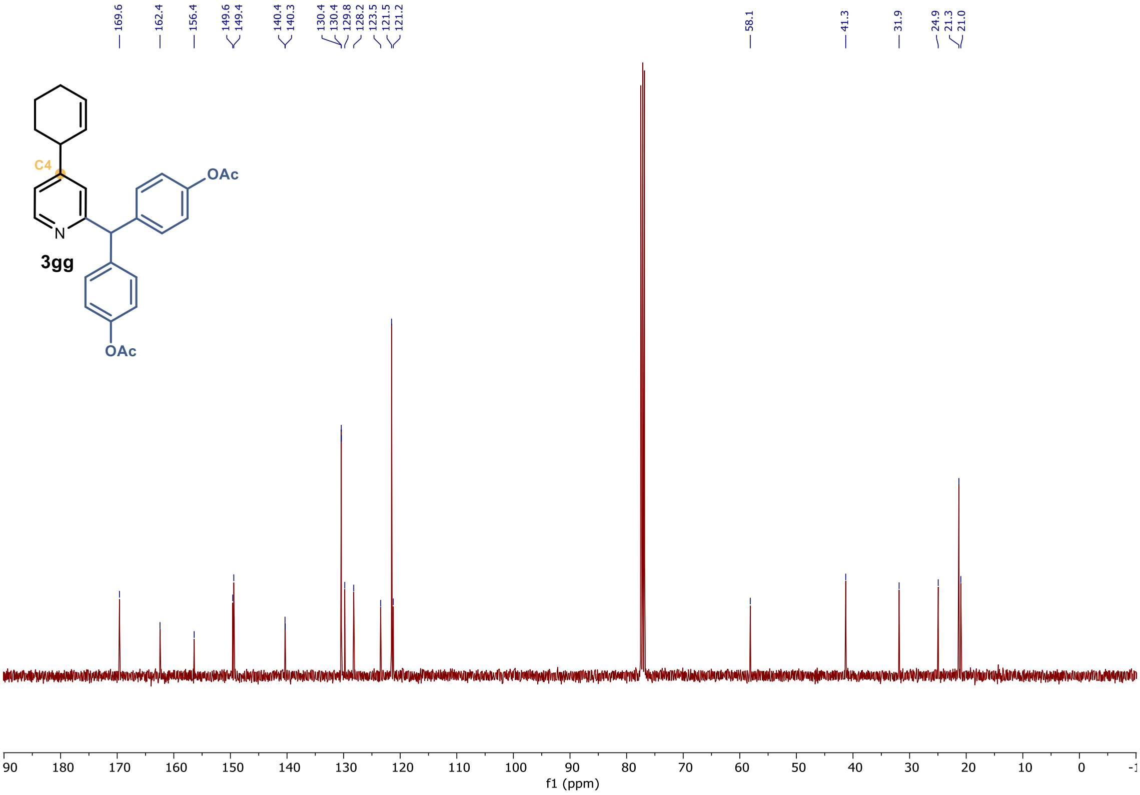

$^{1}$H NMR (400 MHz, CDCl$_3$) of **3hh**

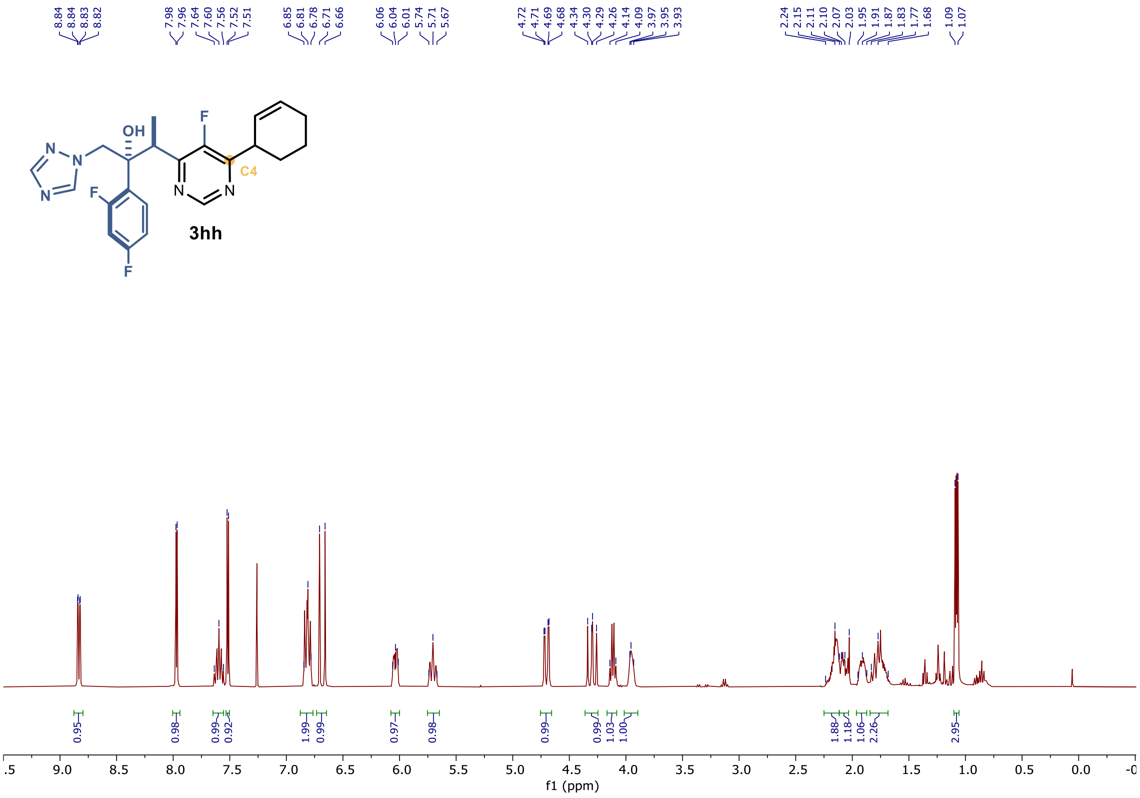


$^{13}$C NMR (101 MHz, CDCl$_3$) of **3hh**

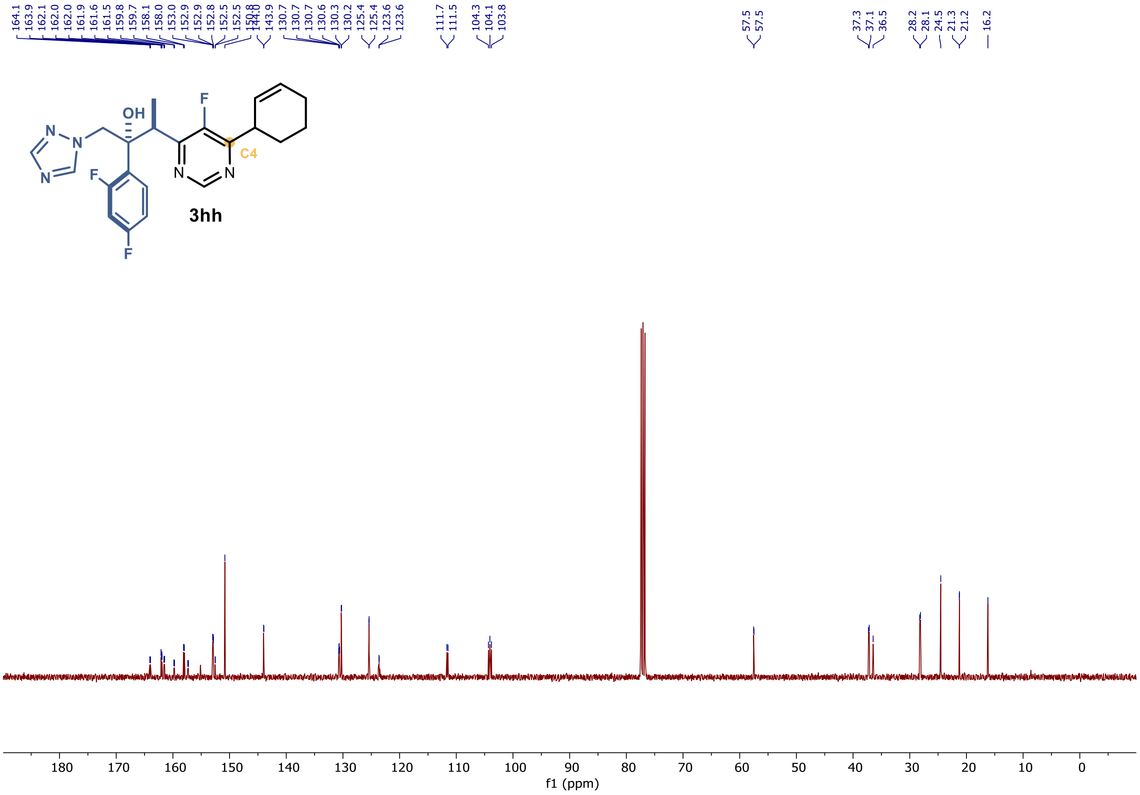

$^{19}F\{^{1}H\}$ NMR (376 MHz, $CDCl_3$) of **3hh**

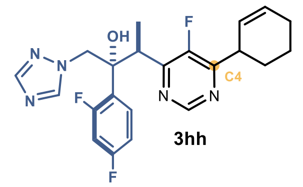


**3hh**

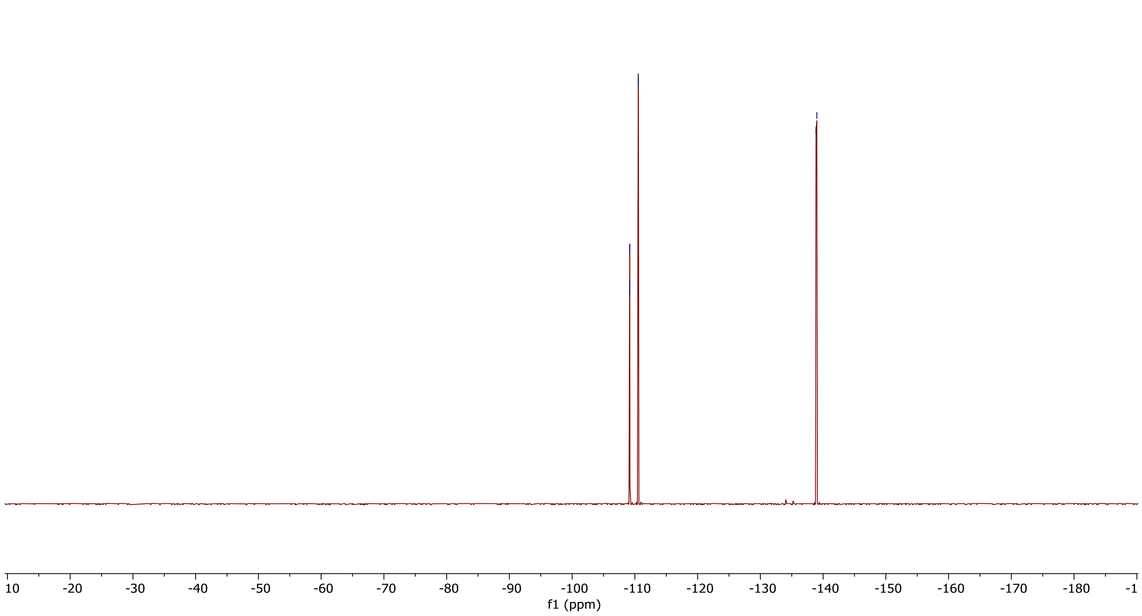

$^{1}H$ NMR (400 MHz, $CDCl_3$) of **3ii**

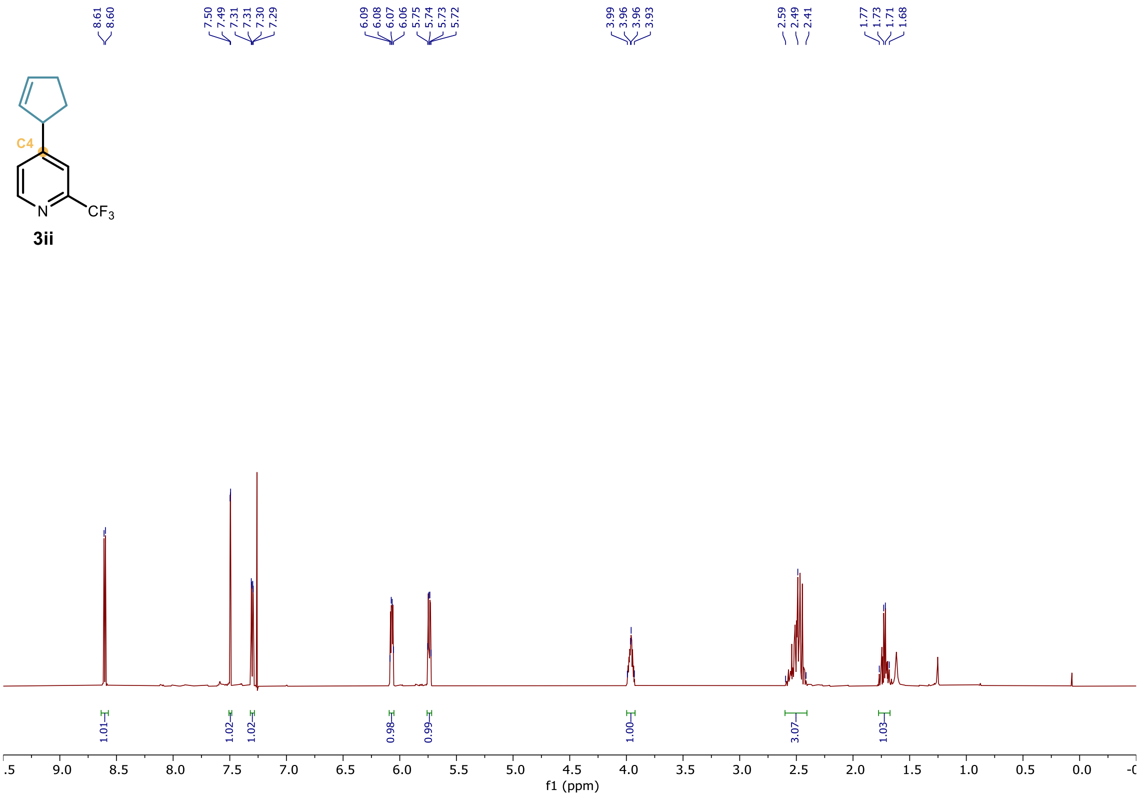


$^{13}C$ NMR (101 MHz, $CDCl_3$) of **3ii**

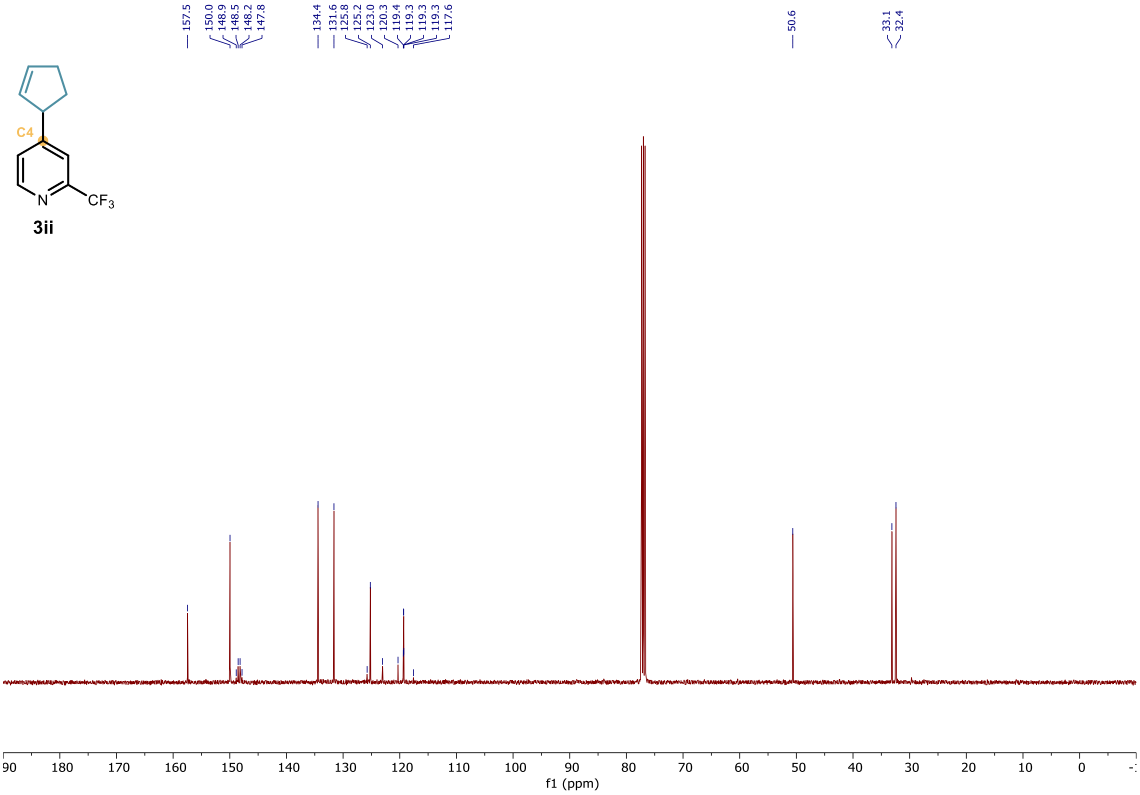

$^{19}F\{^{1}H\}$ NMR (376 MHz, $CDCl_3$) of **3ii**

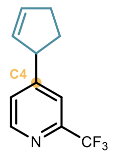


**3ii**

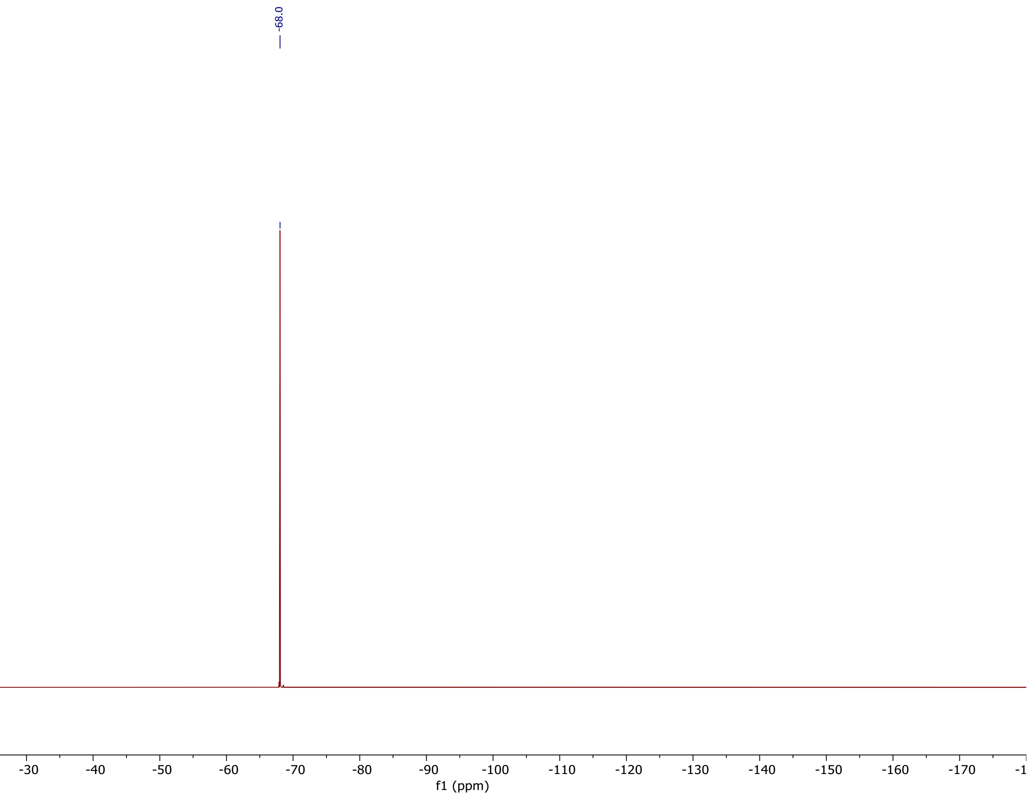

$^{1}$H NMR (400 MHz, $CDCl_3$) of **3jj**

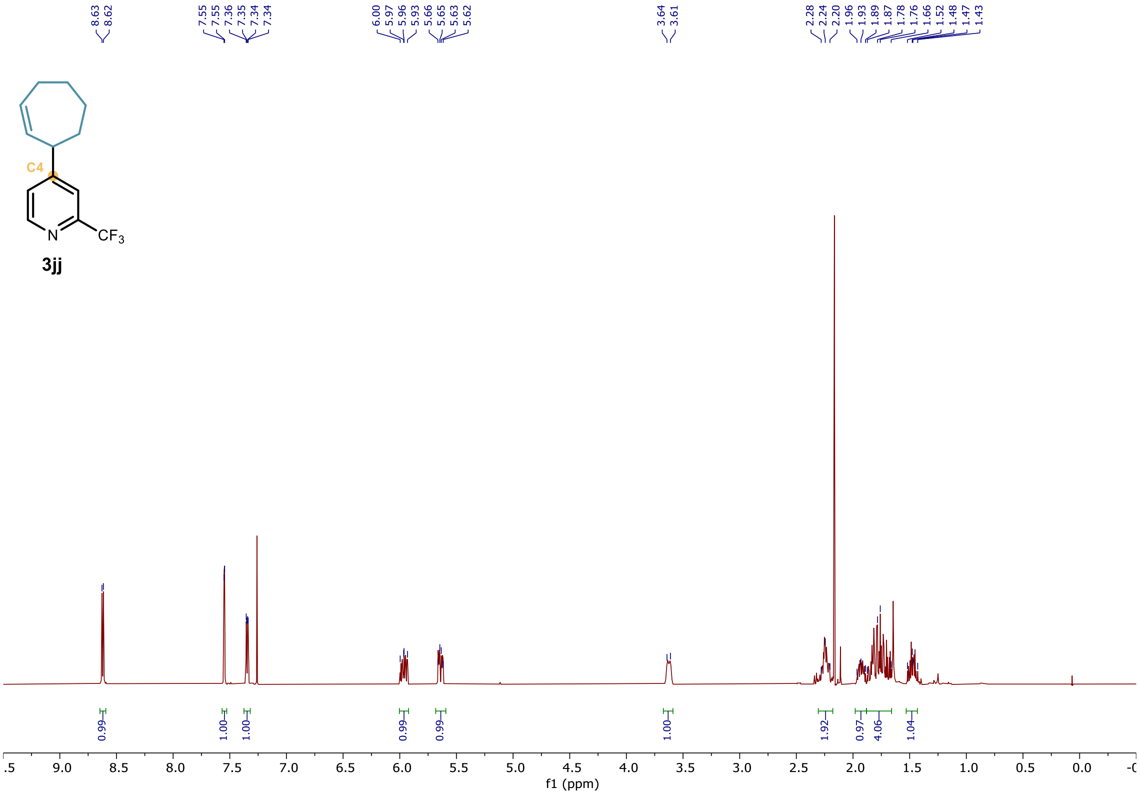


$^{13}$C NMR (101 MHz, $CDCl_3$) of **3jj**

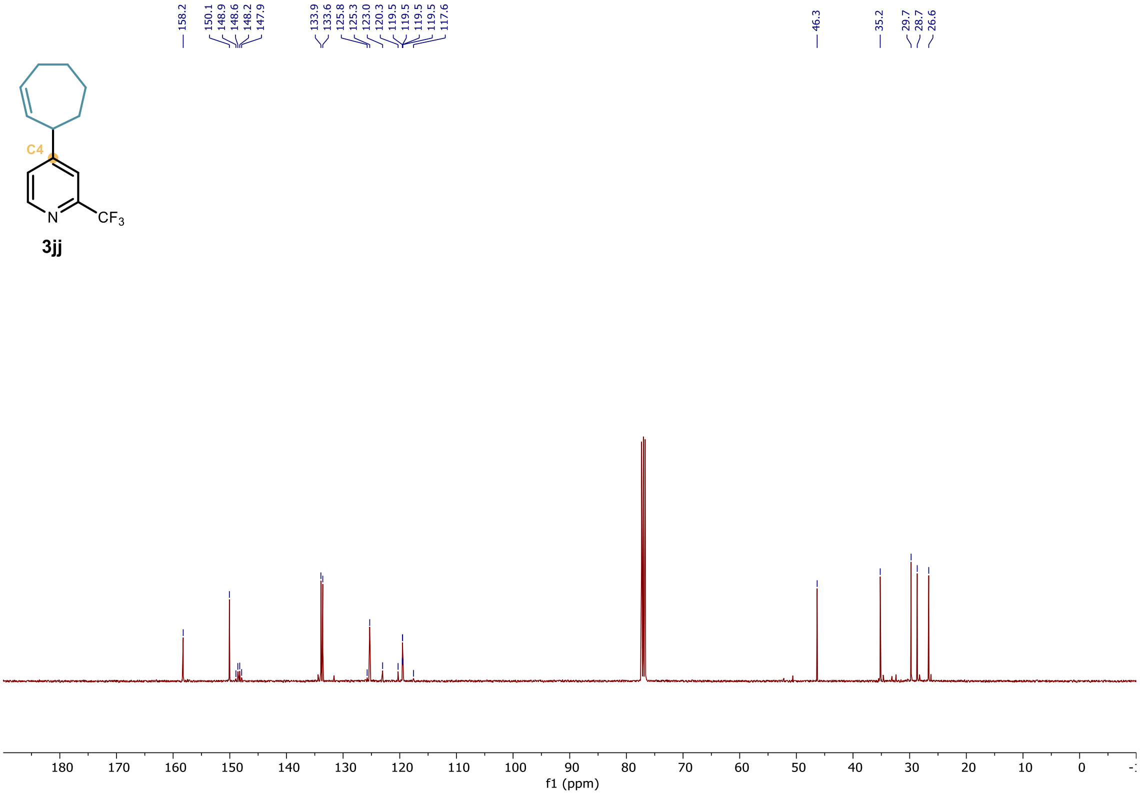

$^{19}F\{^1H\}$ NMR (376 MHz, $CDCl_3$) of **3jj**

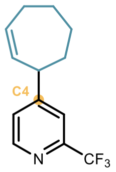


3jj

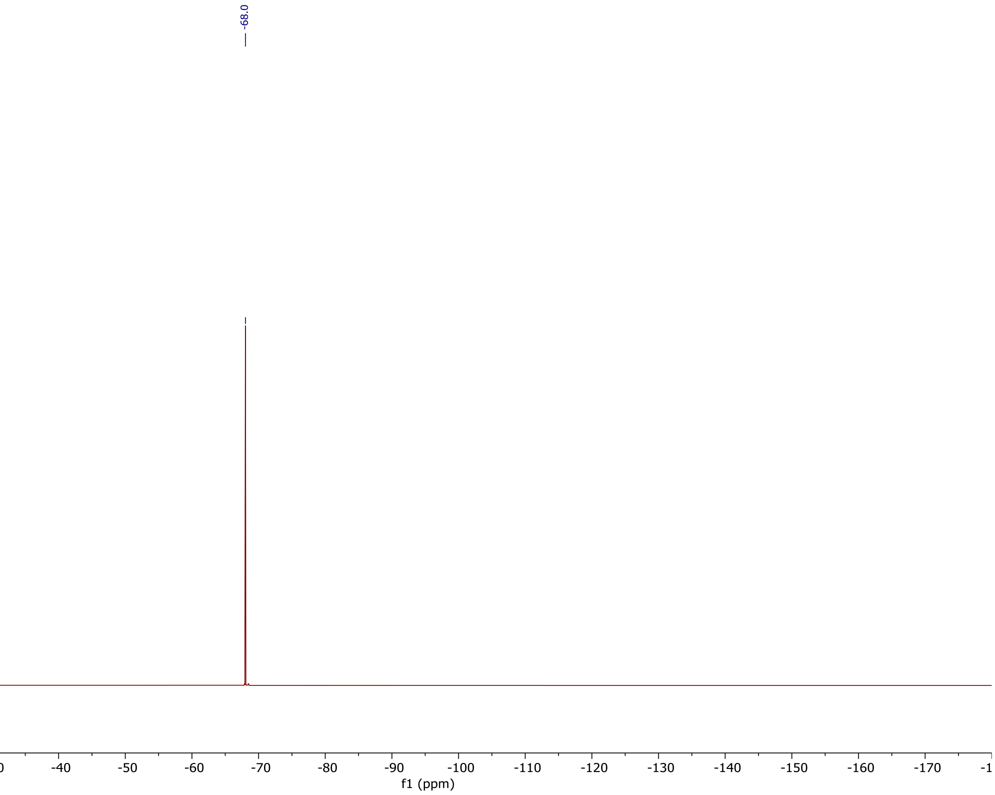

<sup></sup>$^{1}$H NMR (400 MHz, $CDCl_3$) of **3kk + 3kk' + 3kk''**

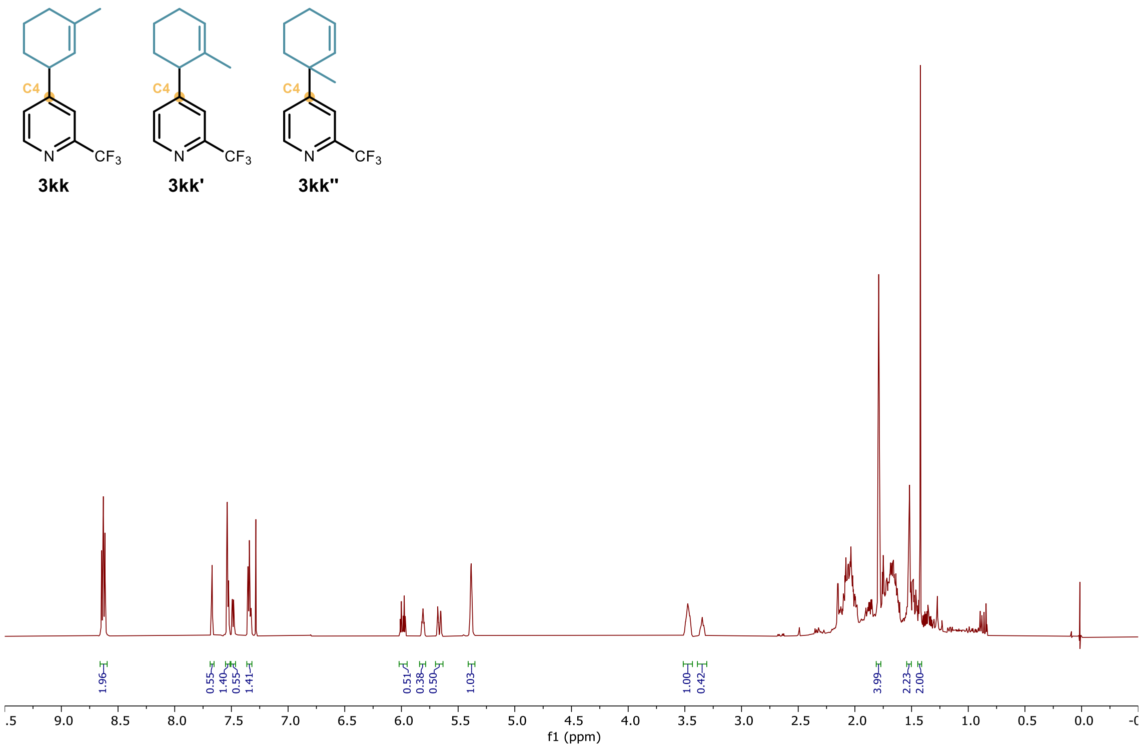

$^{13}$C NMR (101 MHz, $CDCl_3$) of **3kk + 3kk' + 3kk''**

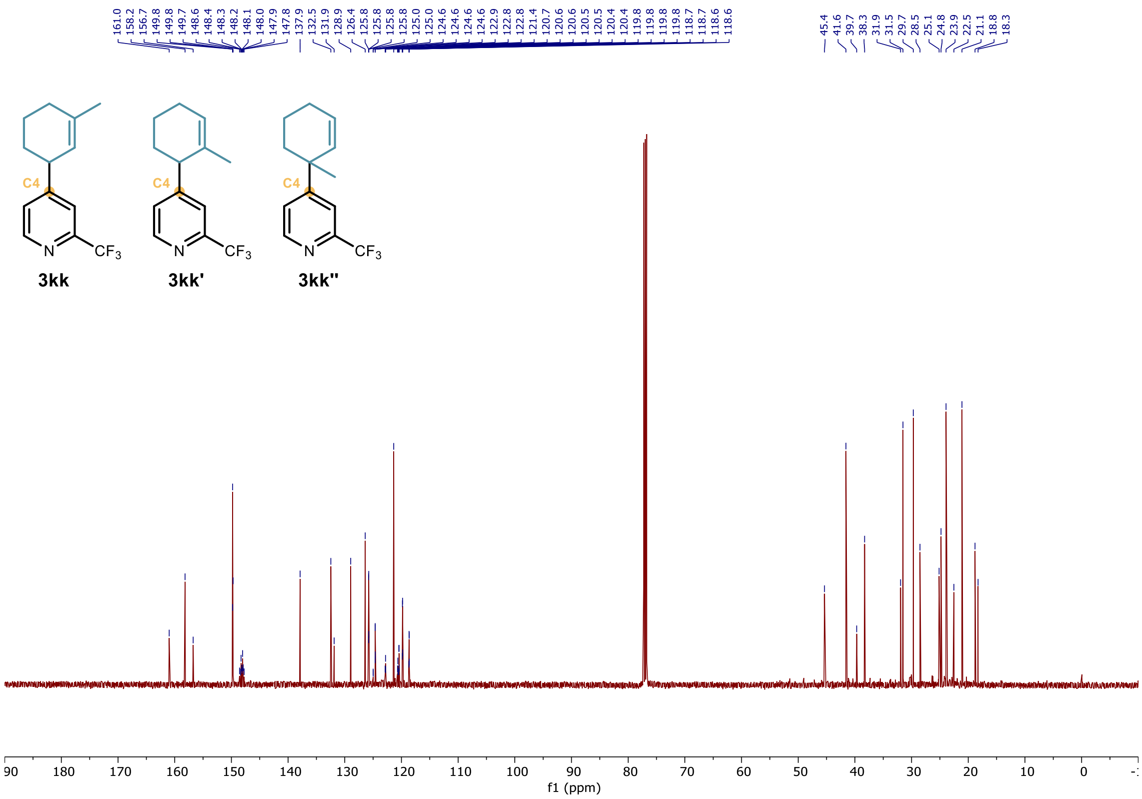

$^{19}F\{^{1}H\}$ NMR (376 MHz, $CDCl_3$) of **3kk + 3kk’ + 3kk’’**

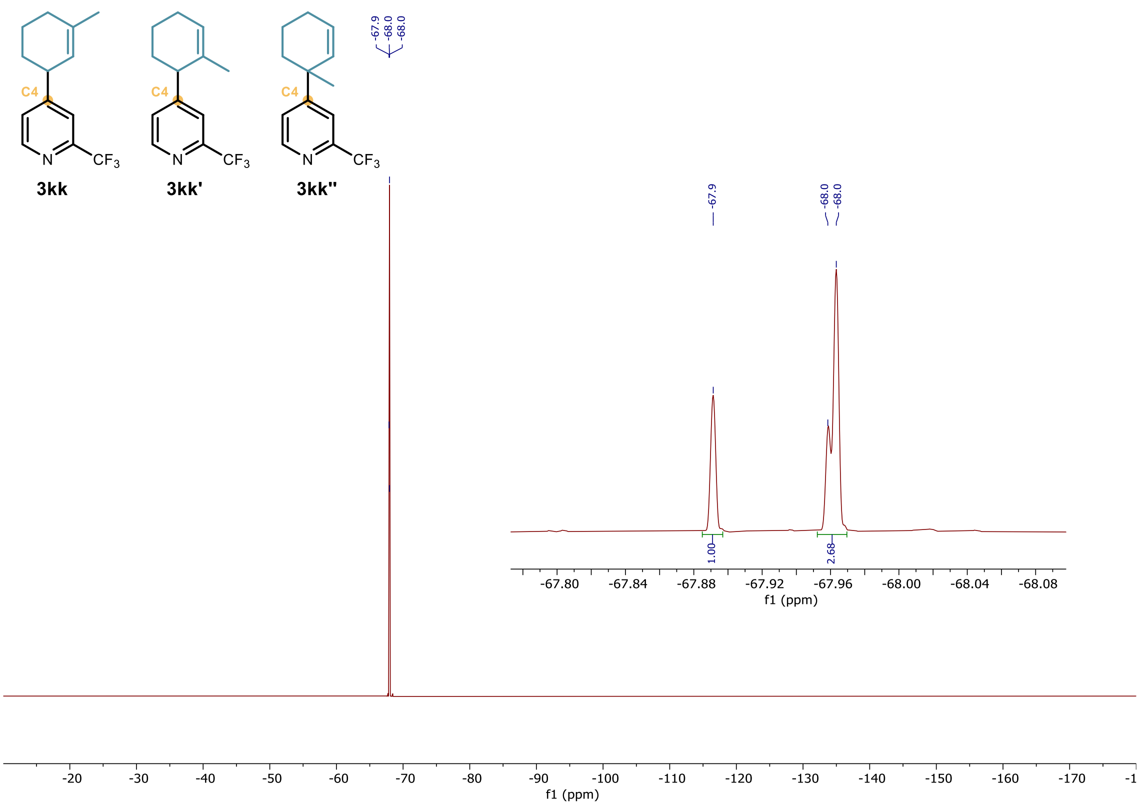

$^{1}H$ NMR (400 MHz, $CDCl_3$) of **3ll + 3ll’**

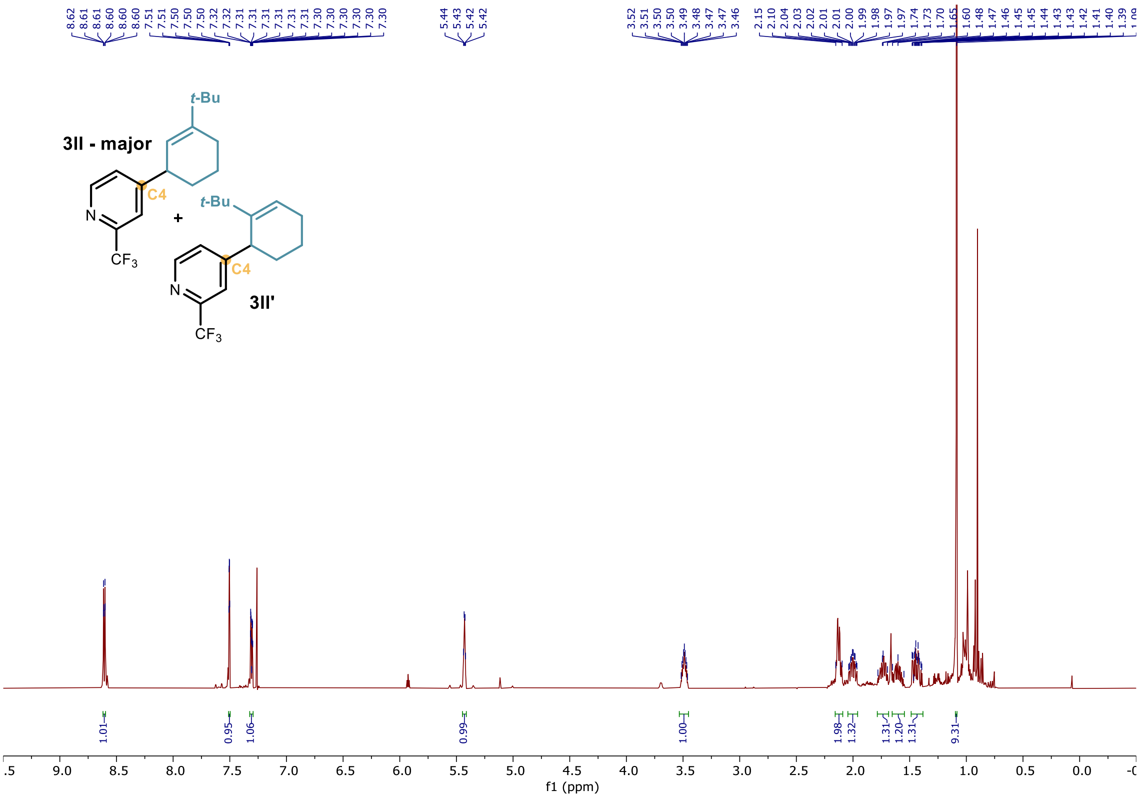


$^{13}C$ NMR (101 MHz, $CDCl_3$) of **3ll + 3ll’**

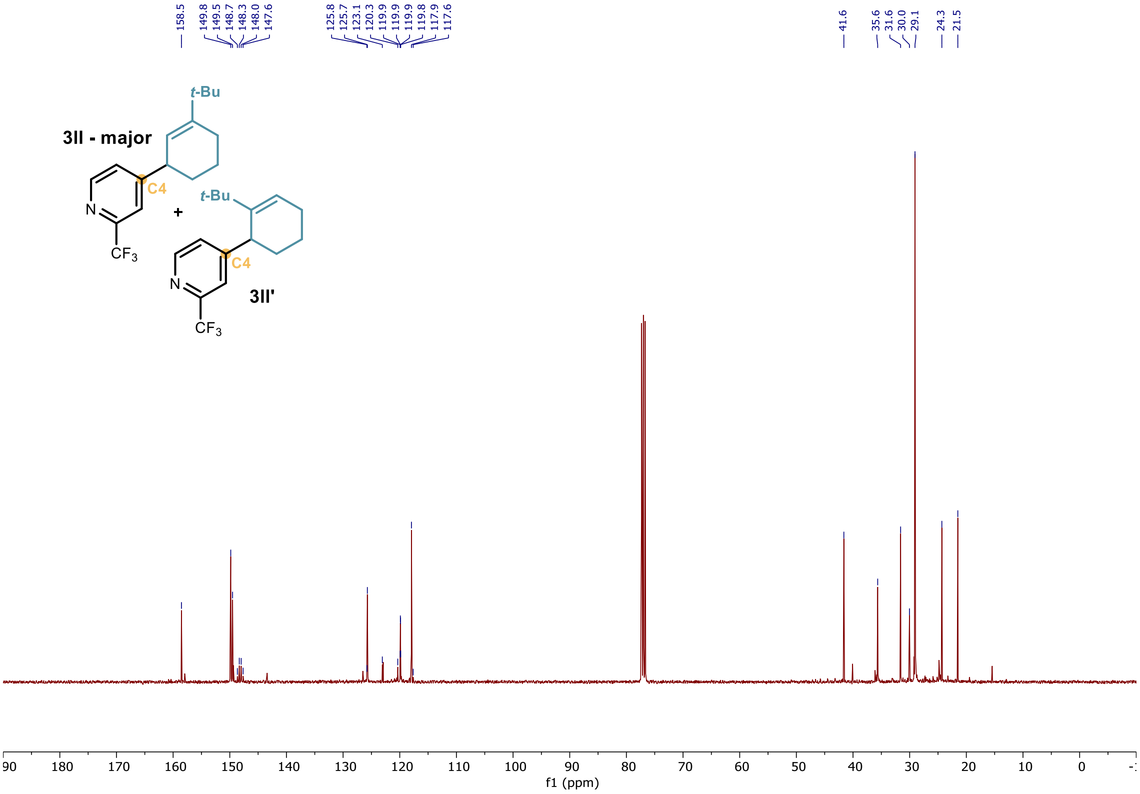

$^{19}F\{^{1}H\}$ NMR (376 MHz, $CDCl_3$) of **3ll + 3ll'**

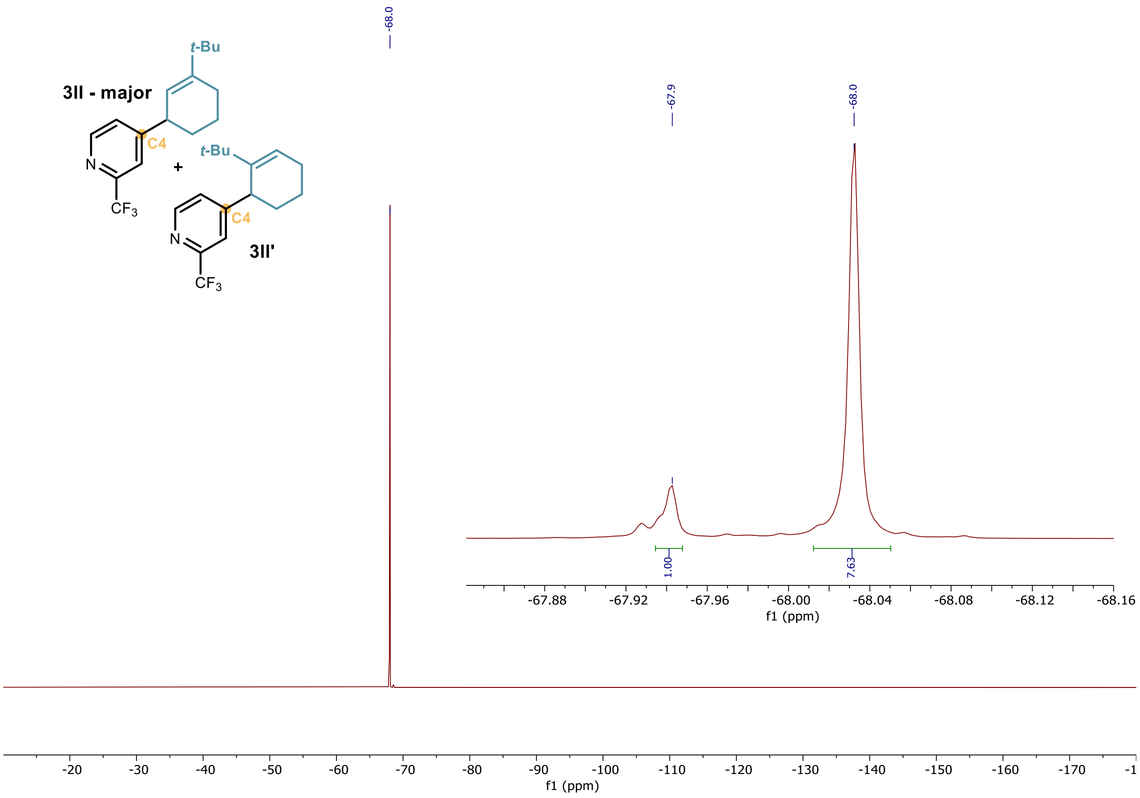

$^{1}$H NMR (400 MHz, $CDCl_3$) of **3mm**

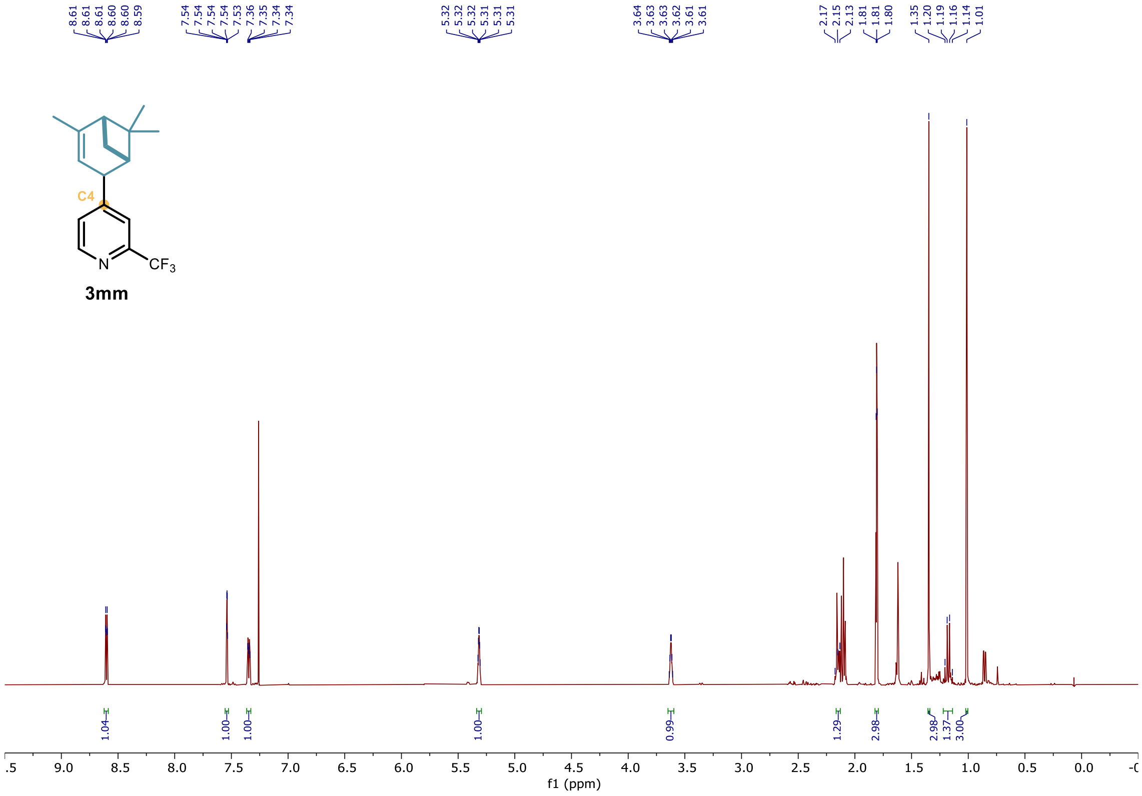

$^{13}$C NMR (101 MHz, $CDCl_3$) of **3mm**

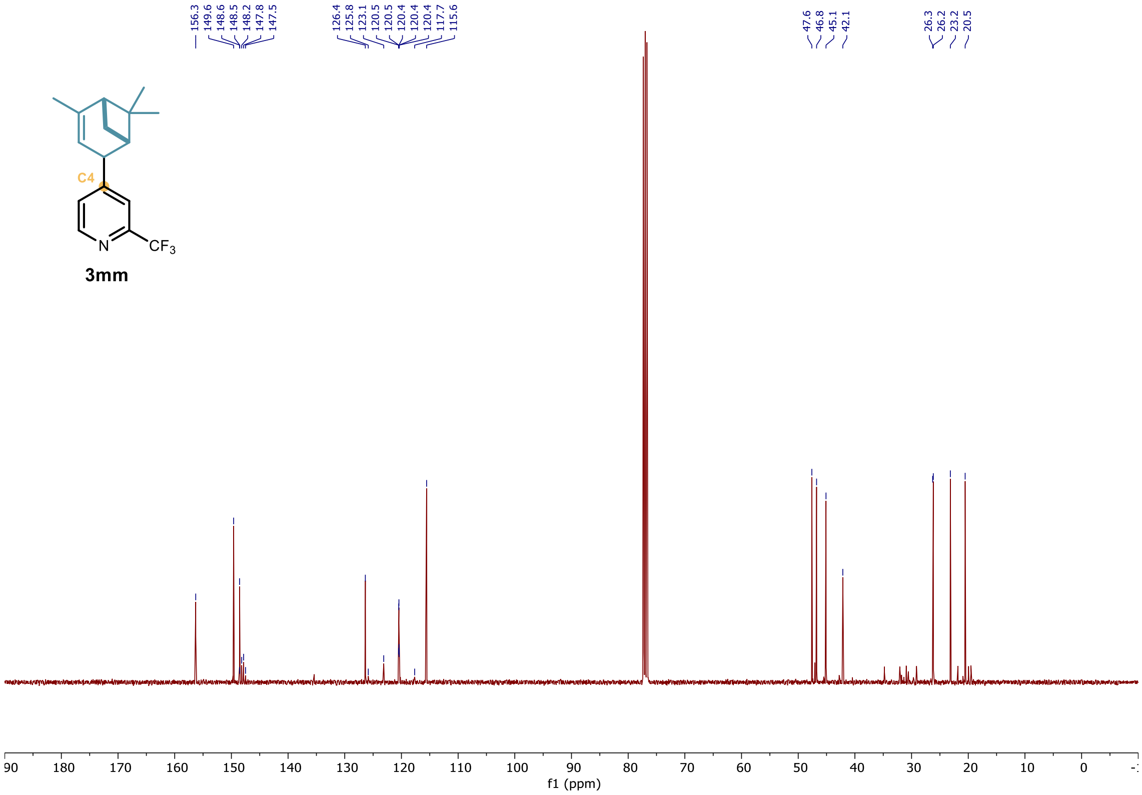

$^{19}F\{^{1}H\}$ NMR (376 MHz, $CDCl_3$) of **3mm**

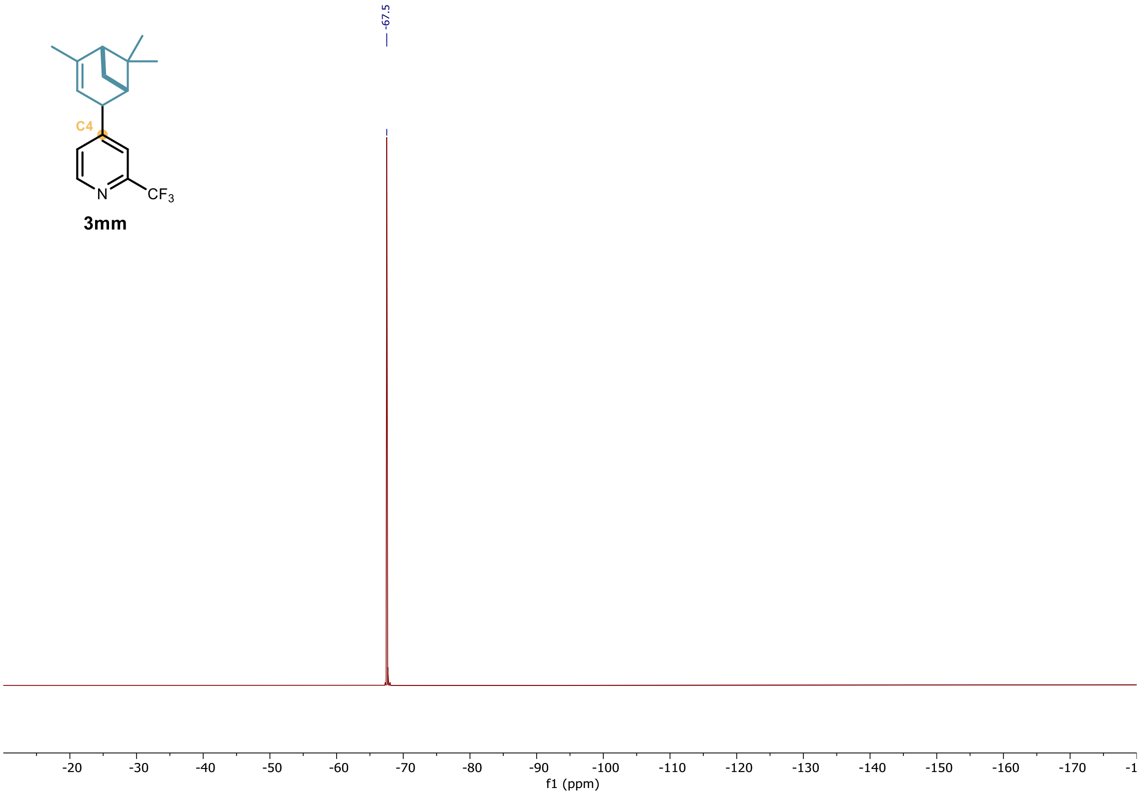

$^{1}$H NMR (400 MHz, $CDCl_3$) of **3nn + 3nn'**

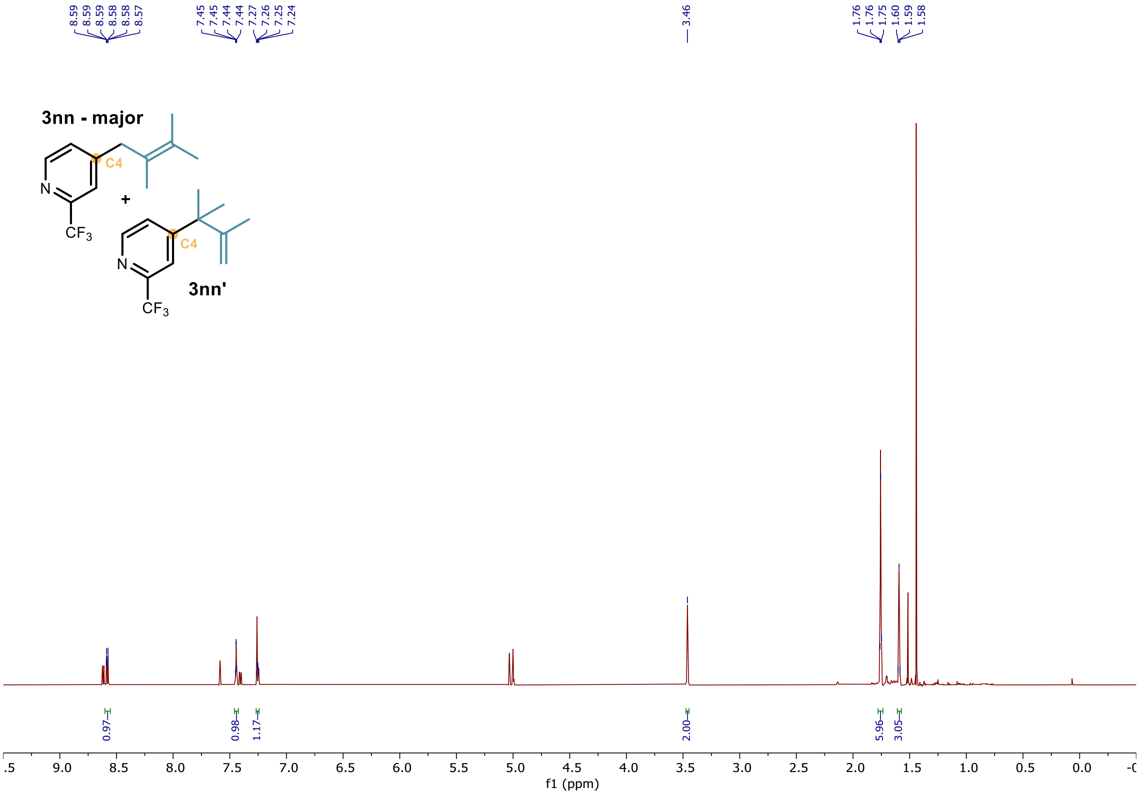


$^{13}$C NMR (101 MHz, $CDCl_3$) of **3nn + 3nn'**

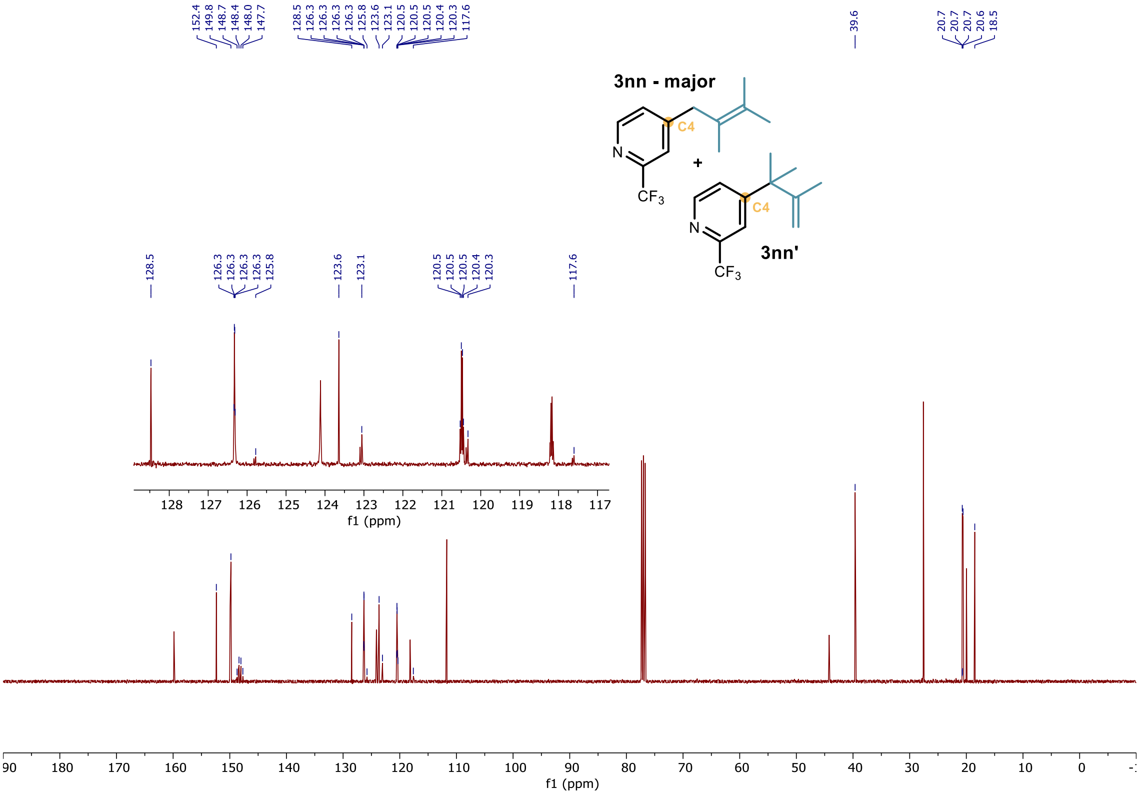

$^{1}$H NMR (400 MHz, $CDCl_3$) of **3nn + 3nn'**

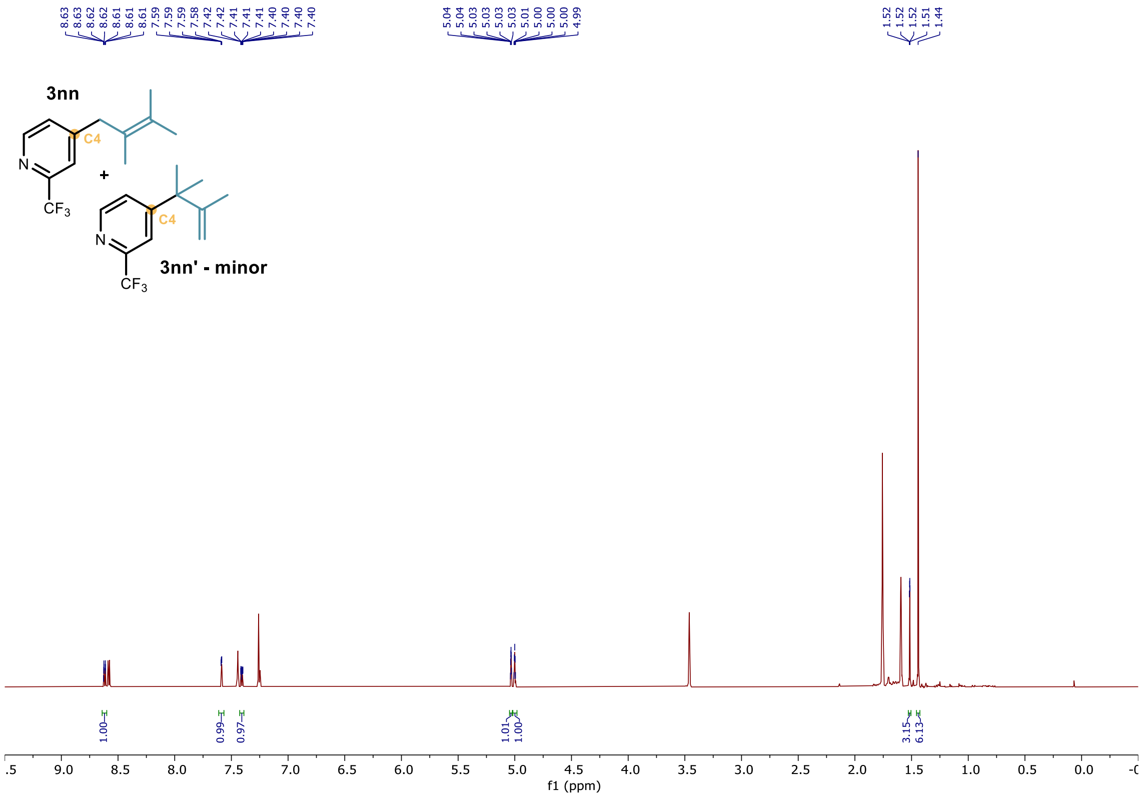

$^{13}$C NMR (101 MHz, $CDCl_3$) of **3nn + 3nn'**

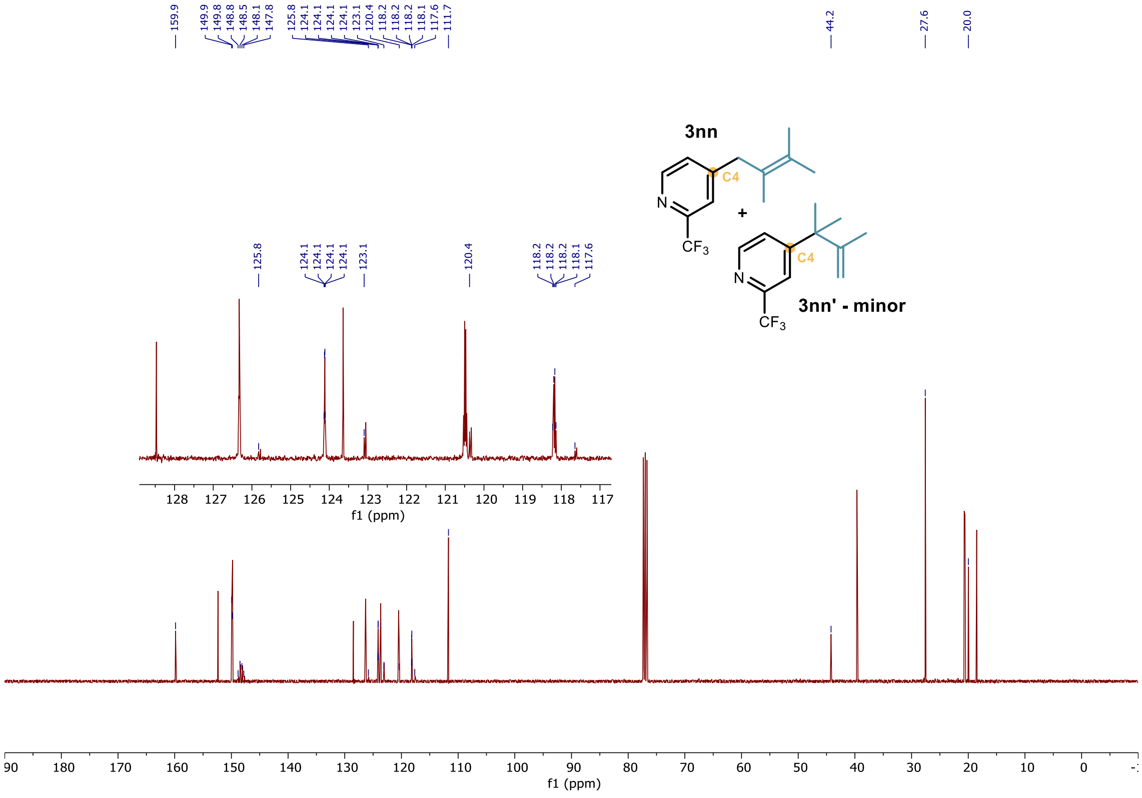

$^{19}F\{^{1}H\}$ NMR (376 MHz, $CDCl_3$) of **3nn + 3nn'**

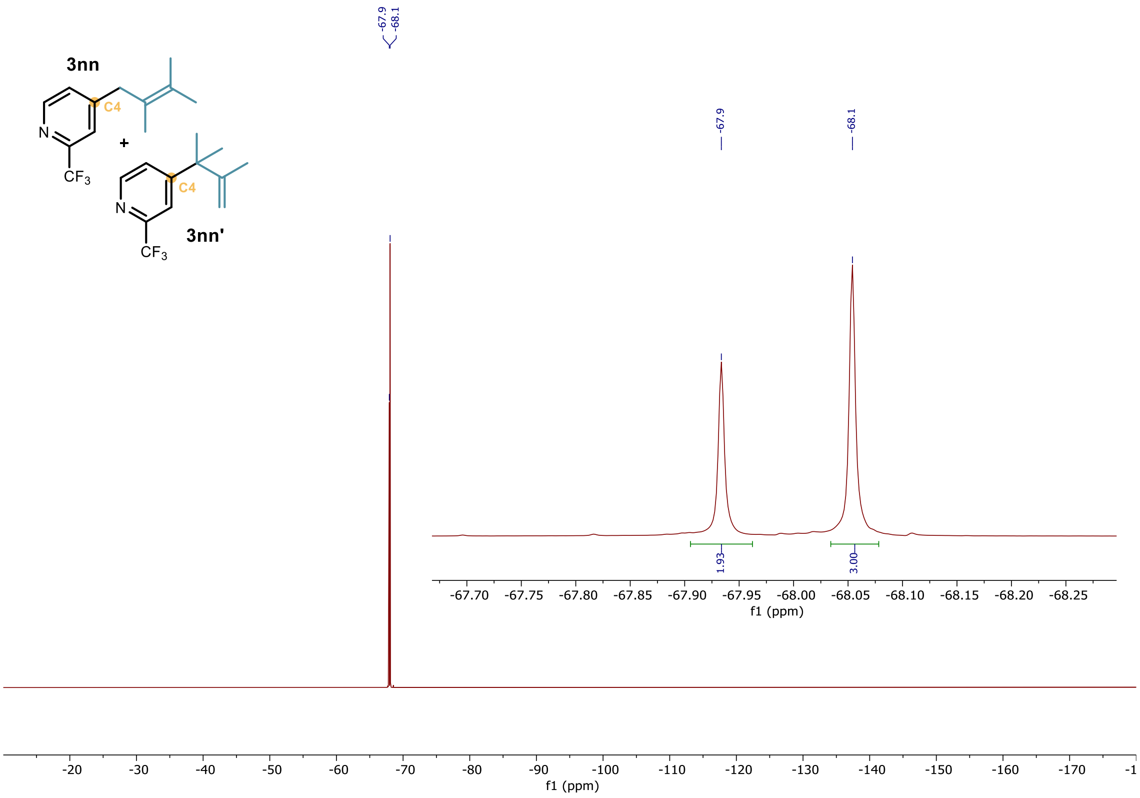

$^{1}$H NMR (400 MHz, $CDCl_3$) of **3oo + 3oo'**

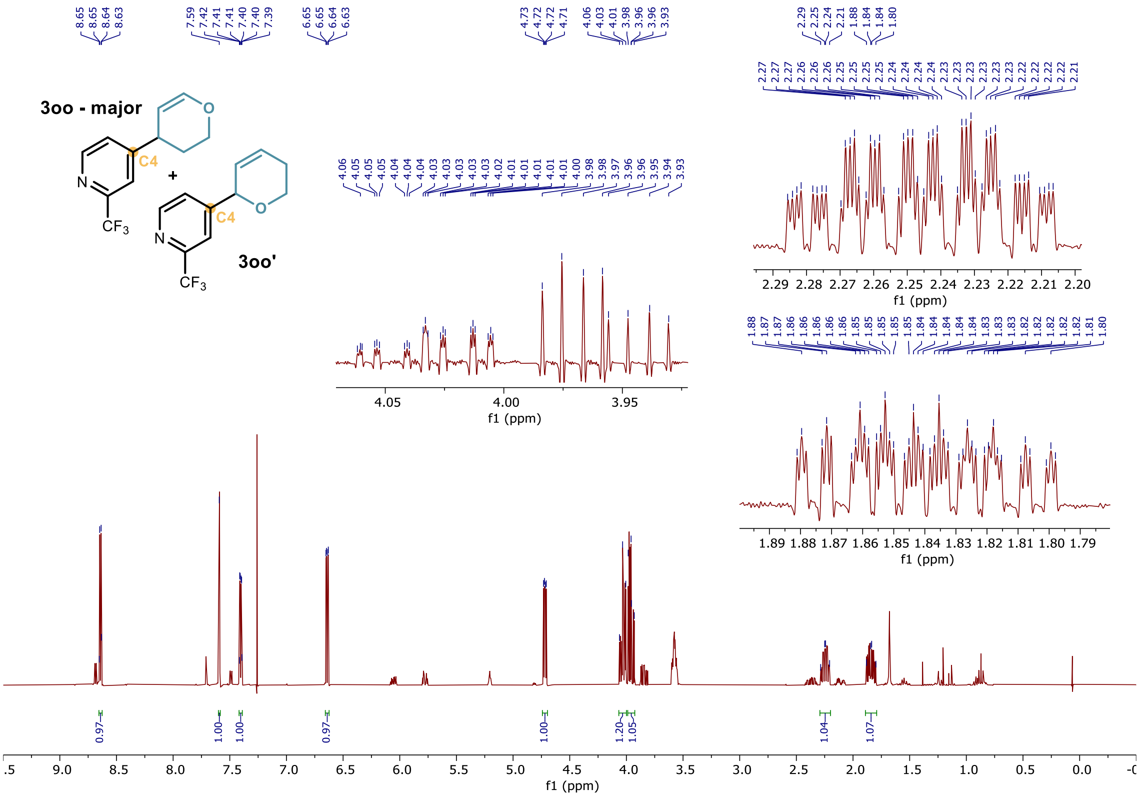


$^{13}$C NMR (101 MHz, $CDCl_3$) of **3oo + 3oo'**

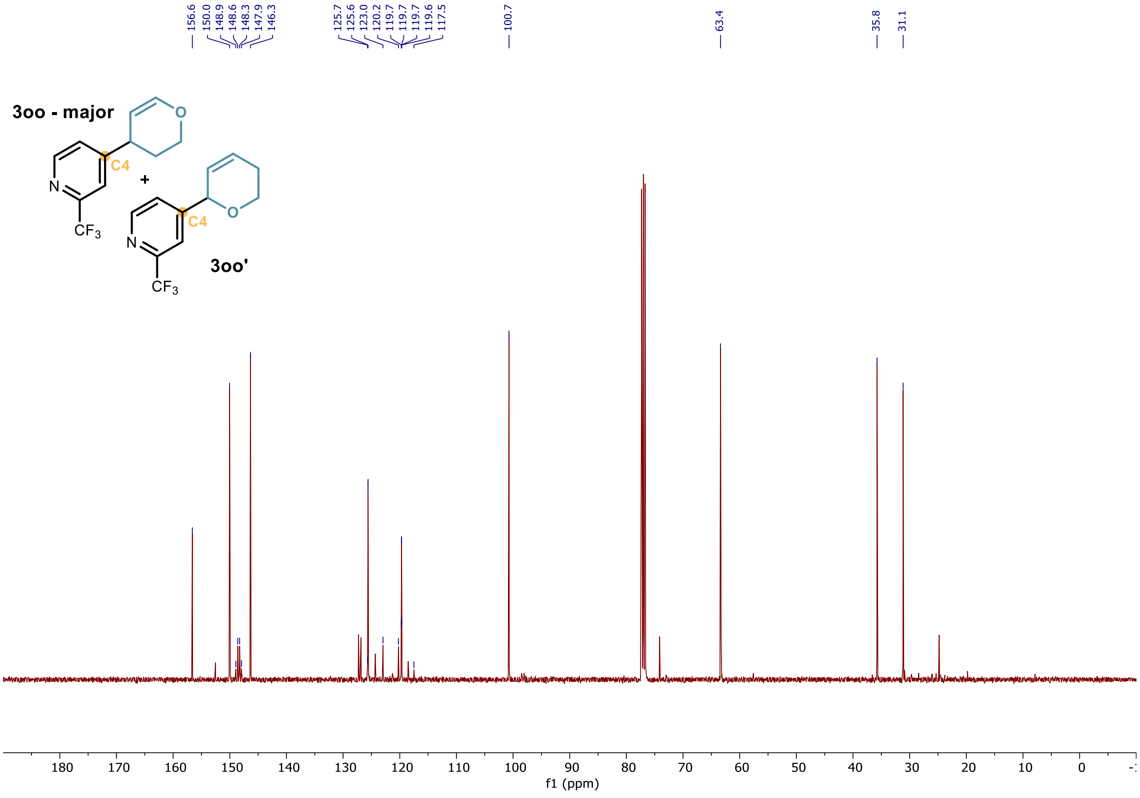

$^{19}F\{^1H\}$ NMR (376 MHz, $CDCl_3$) of **3oo + 3oo'**

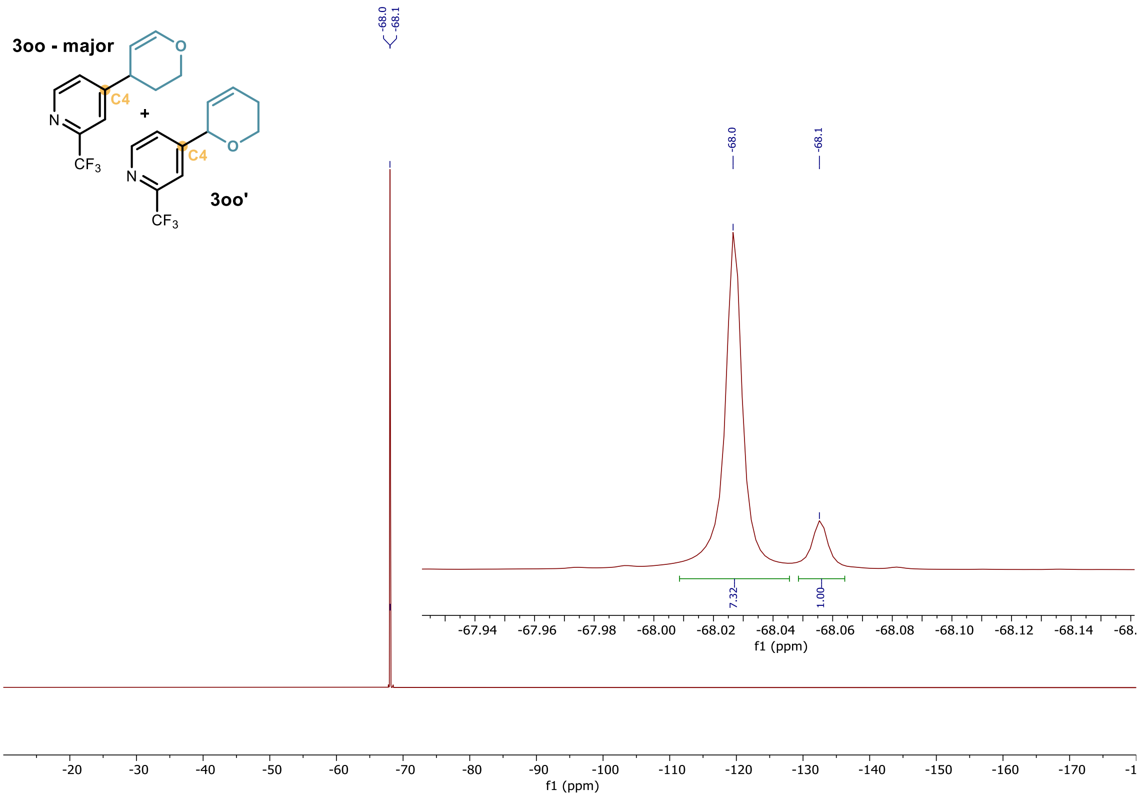

$^{1}H$ NMR (500 MHz, $CDCl_3$, 328K) of **3pp**

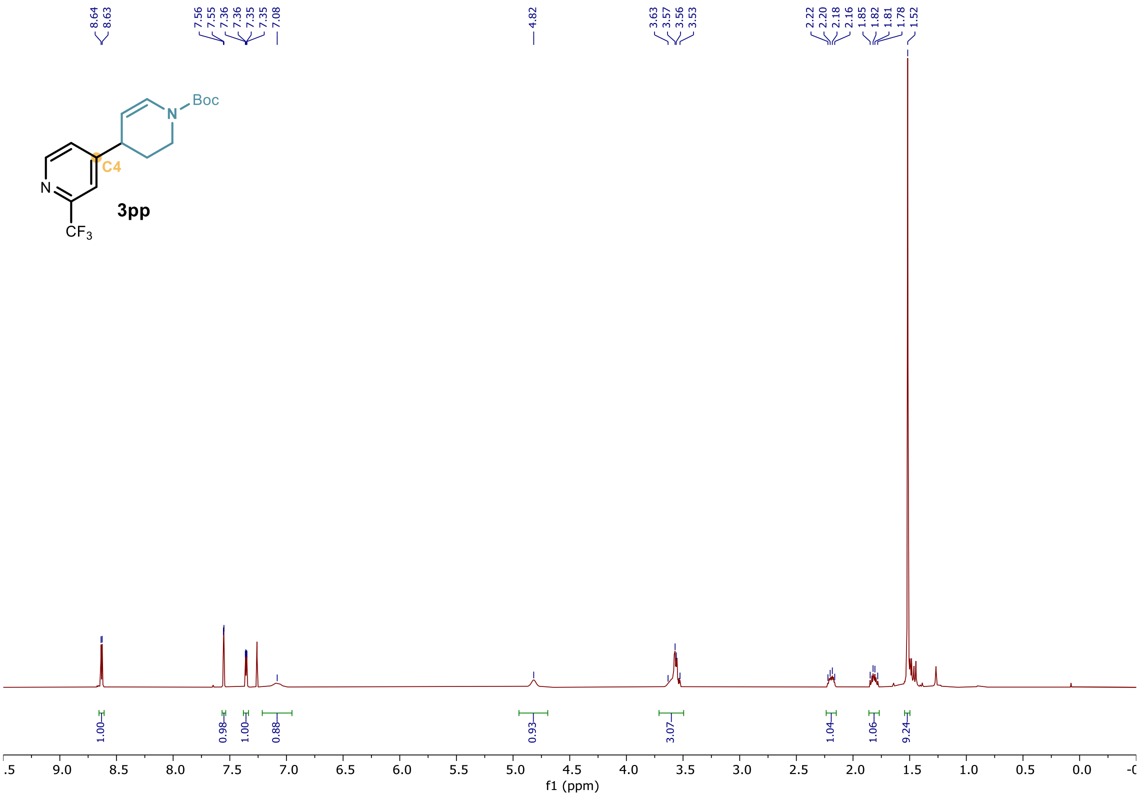


$^{13}C$ NMR (126 MHz, $CDCl_3$, 328K) of **3pp**

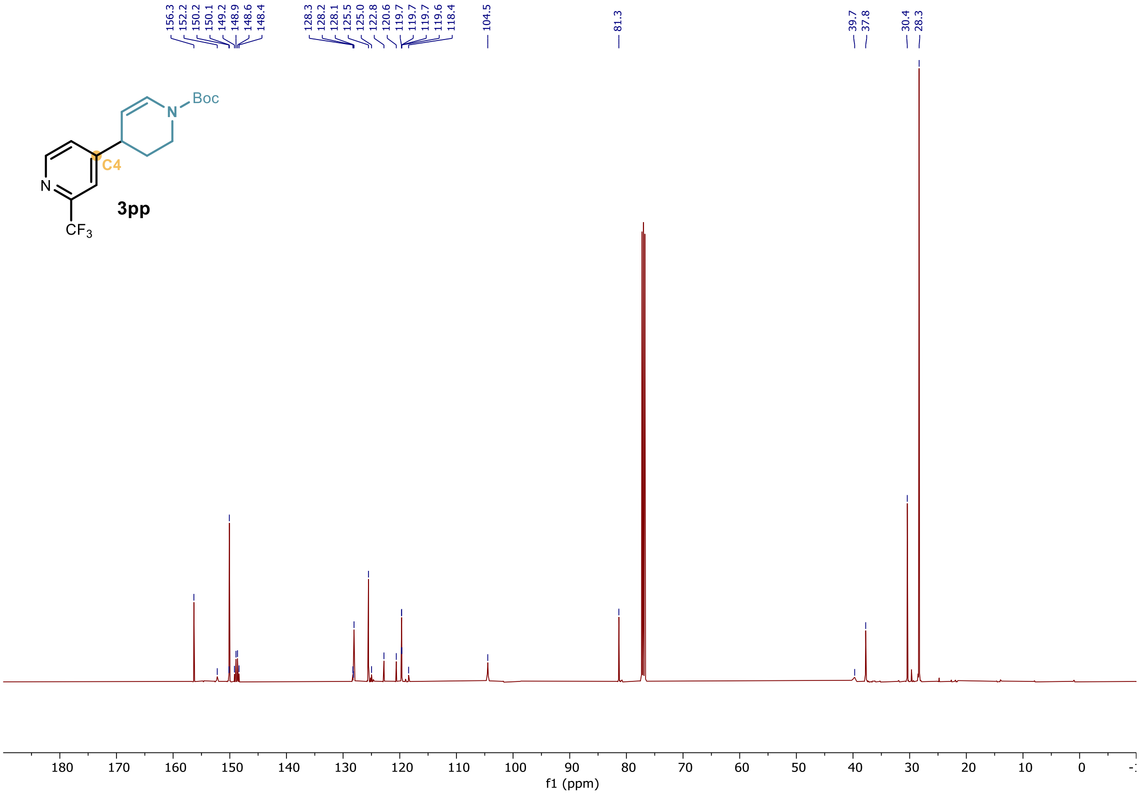

$^{19}F\{^{1}H\}$ NMR (376 MHz, $CDCl_3$) of **3pp**

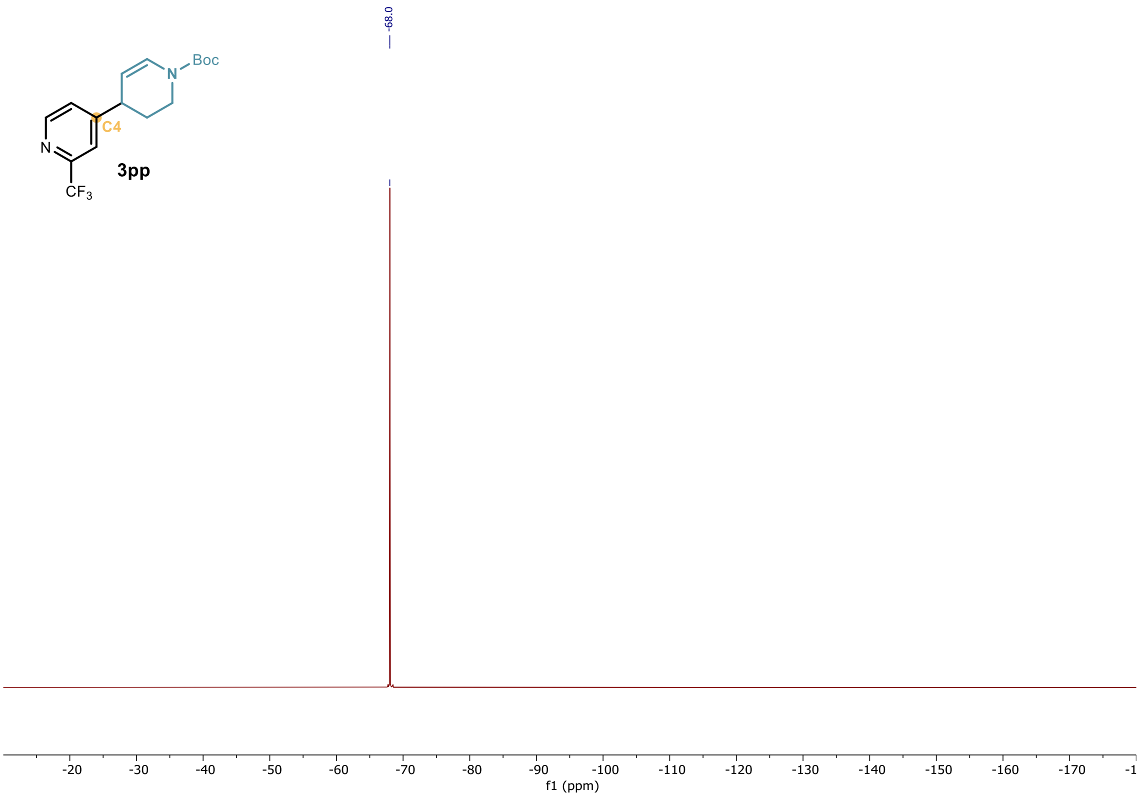

$^{1}$H NMR (400 MHz, $CDCl_3$) of **3qq**

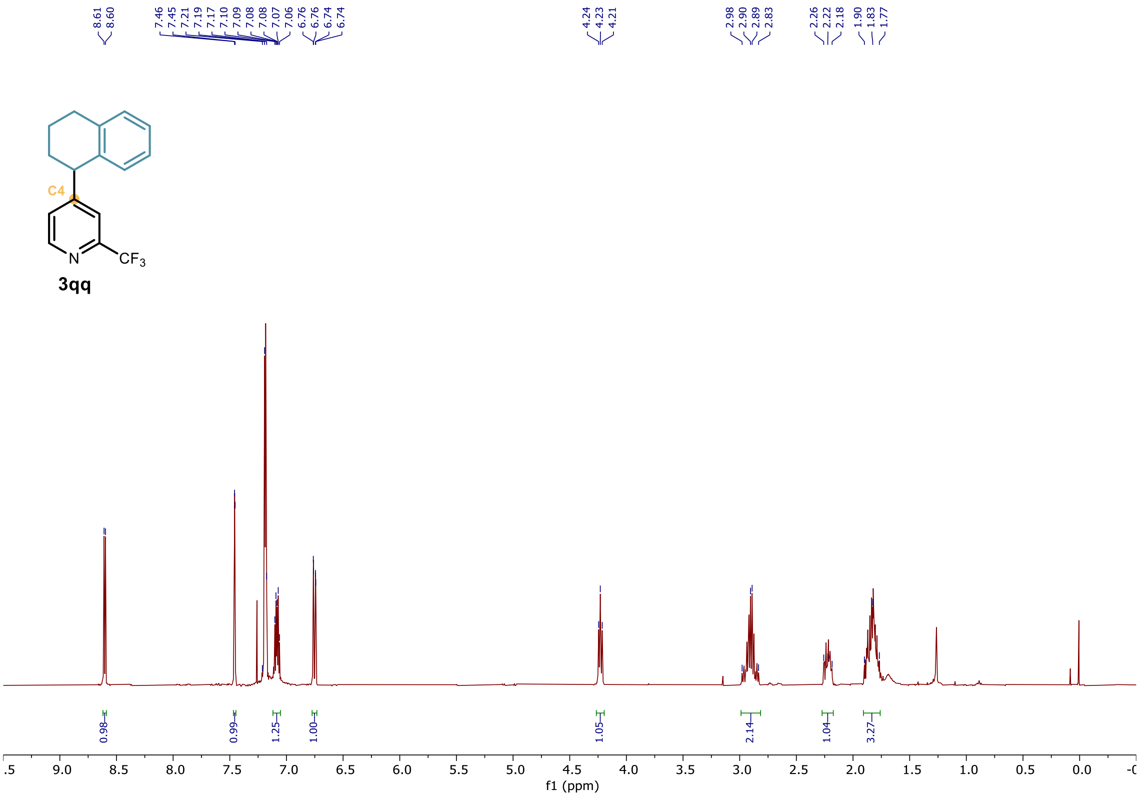


$^{13}$C NMR (101 MHz, $CDCl_3$) of **3qq**

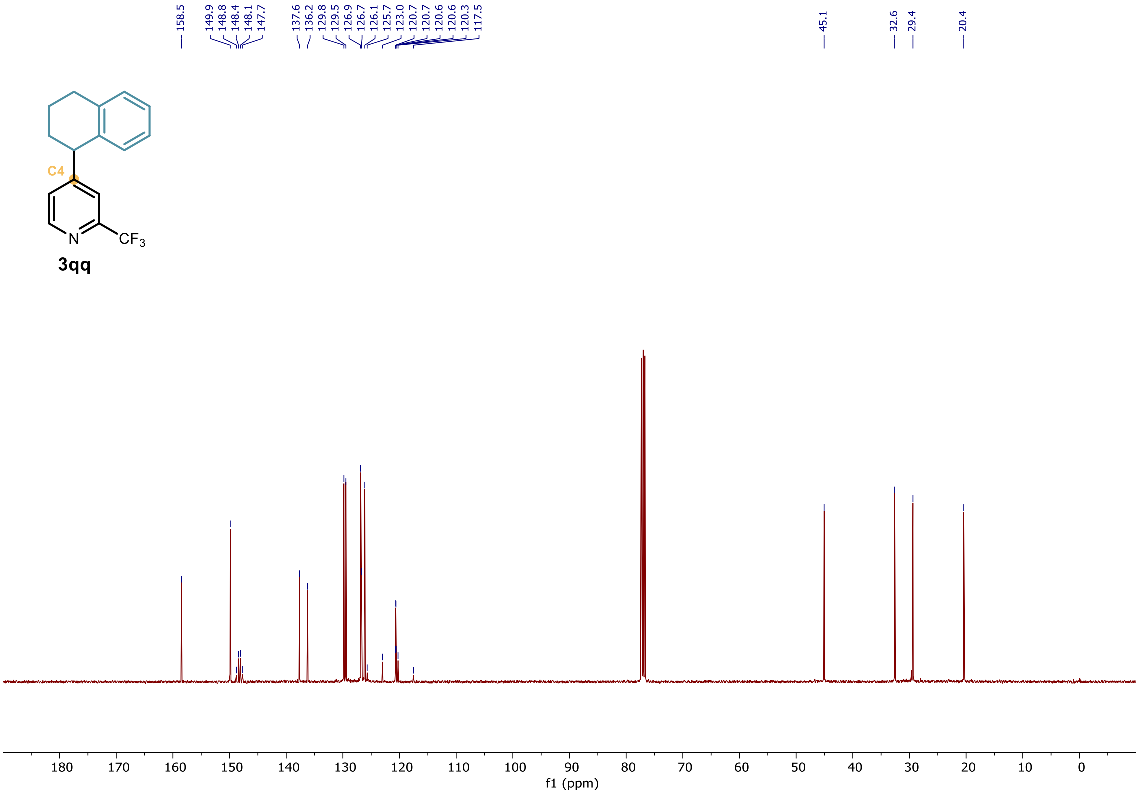

$^{19}F\{^{1}H\}$ NMR (376 MHz, $CDCl_3$) of **3qq**

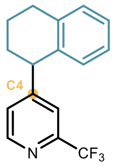


**3qq**

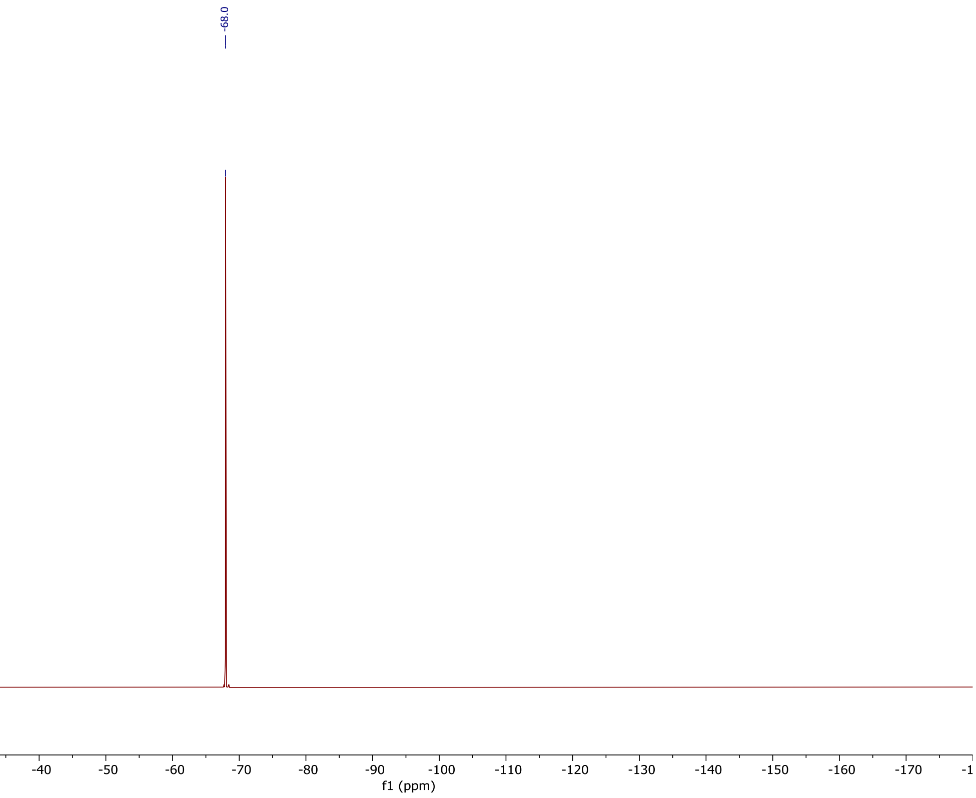

$^{1}$H NMR (400 MHz, $CDCl_3$) of **3rr**

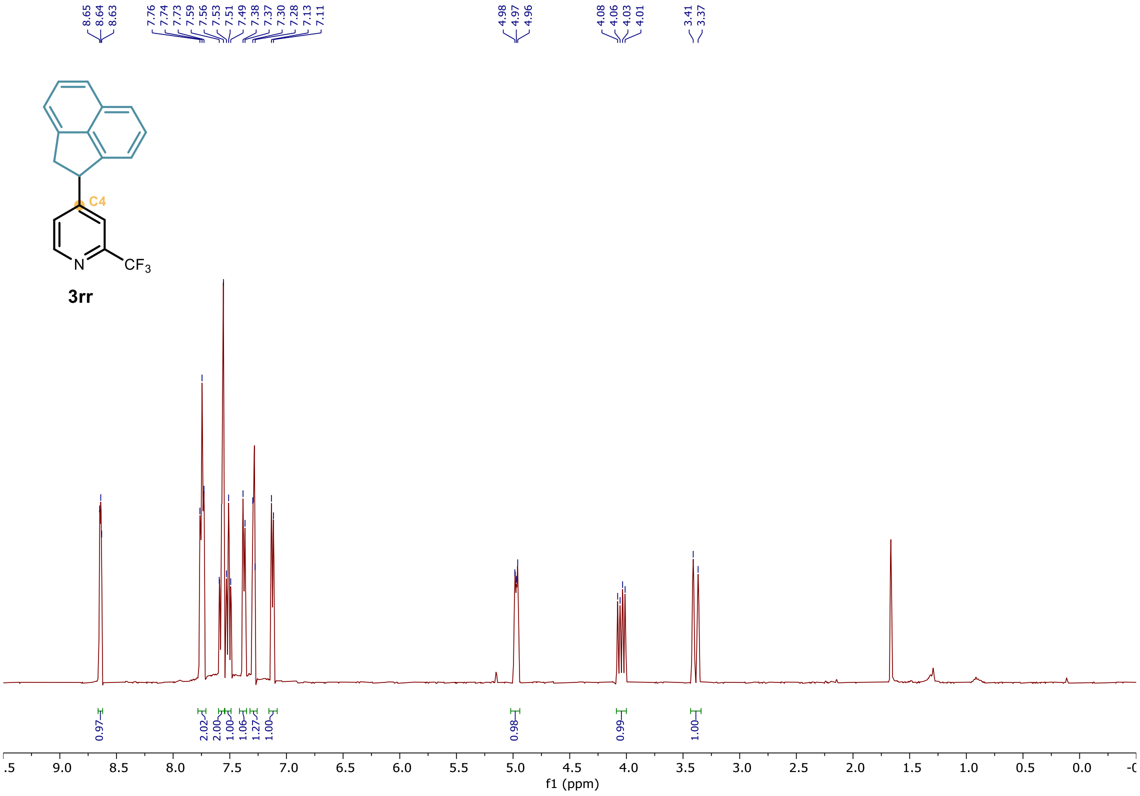


$^{13}$C NMR (101 MHz, $CDCl_3$) of **3rr**

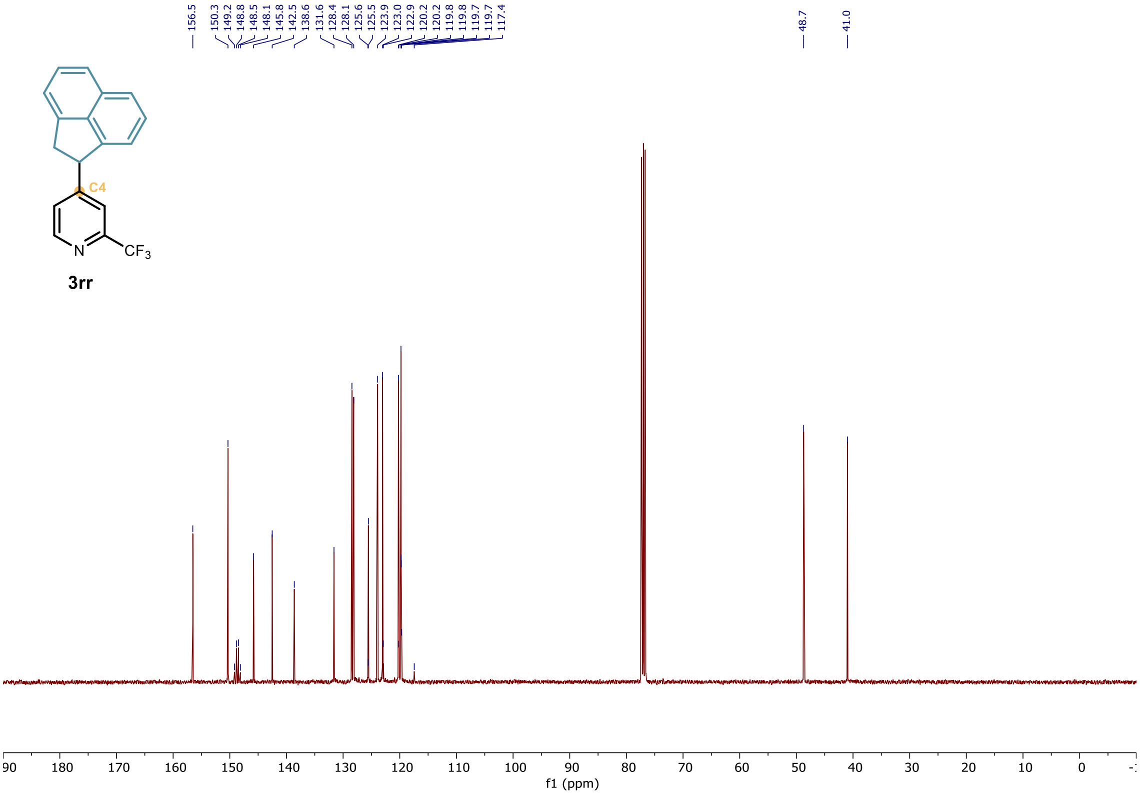

$^{19}F\{^{1}H\}$ NMR (376 MHz, $CDCl_3$) of **3rr**

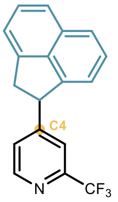


**3rr**

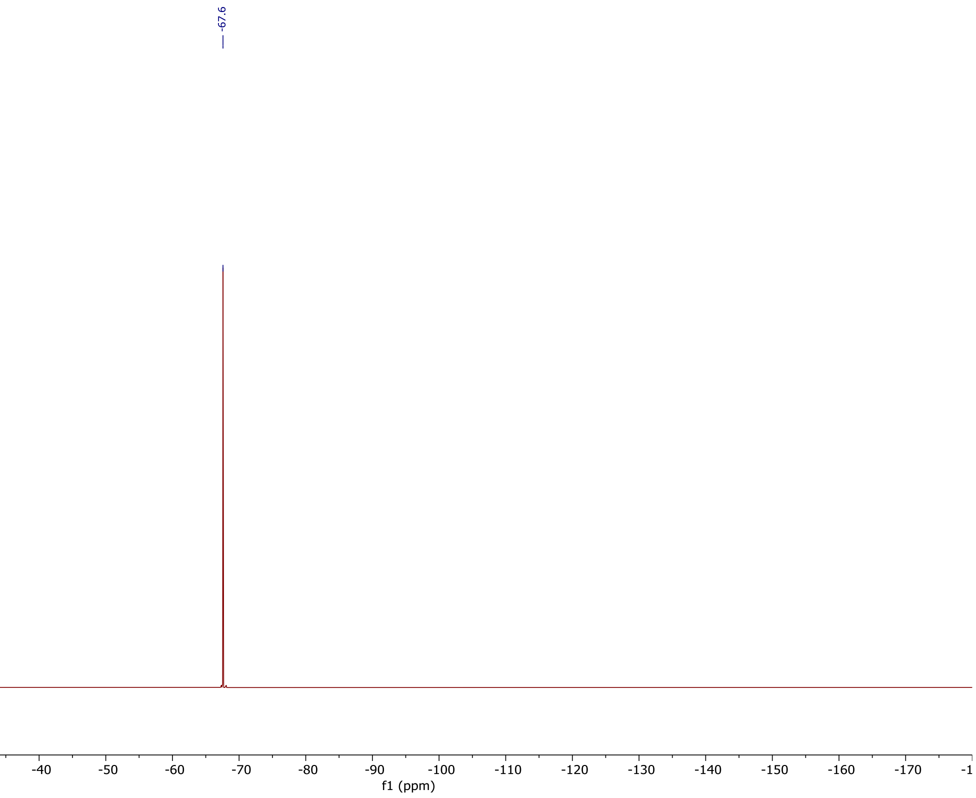

$^{1}H$ NMR (400 MHz, $CDCl_3$) of **5a**

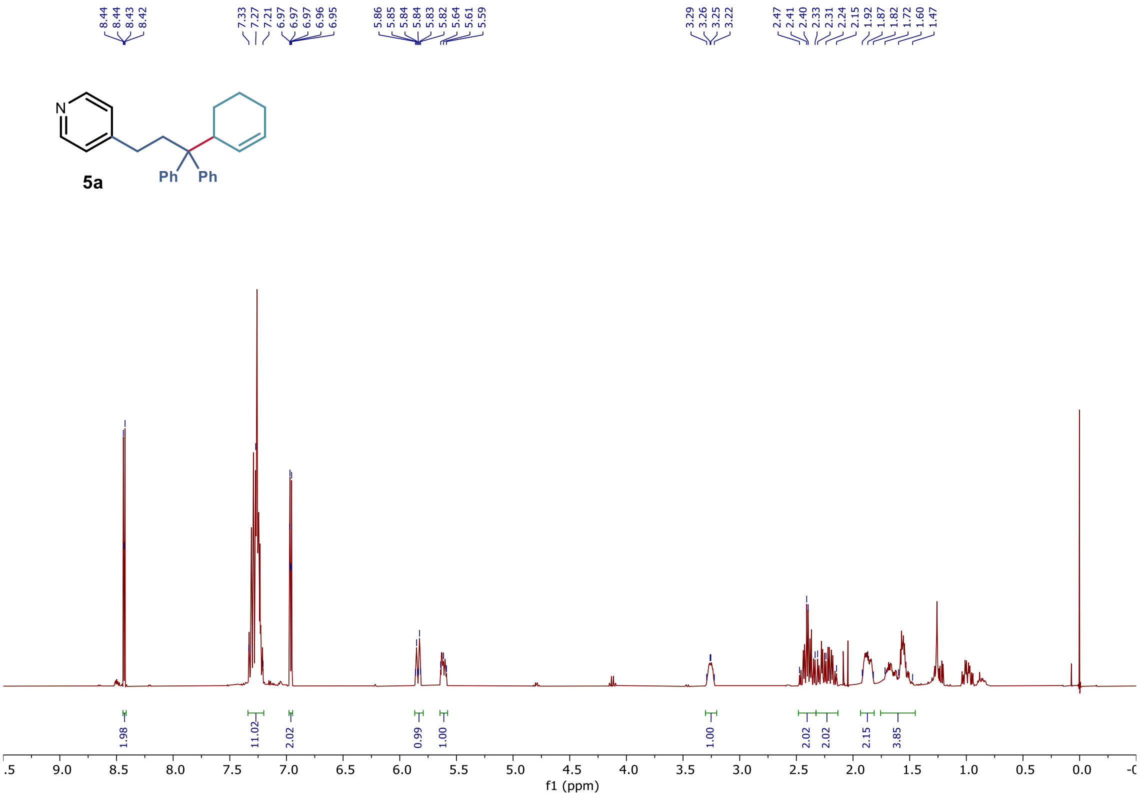

$^{13}C$ NMR (101 MHz, $CDCl_3$) of **5a**

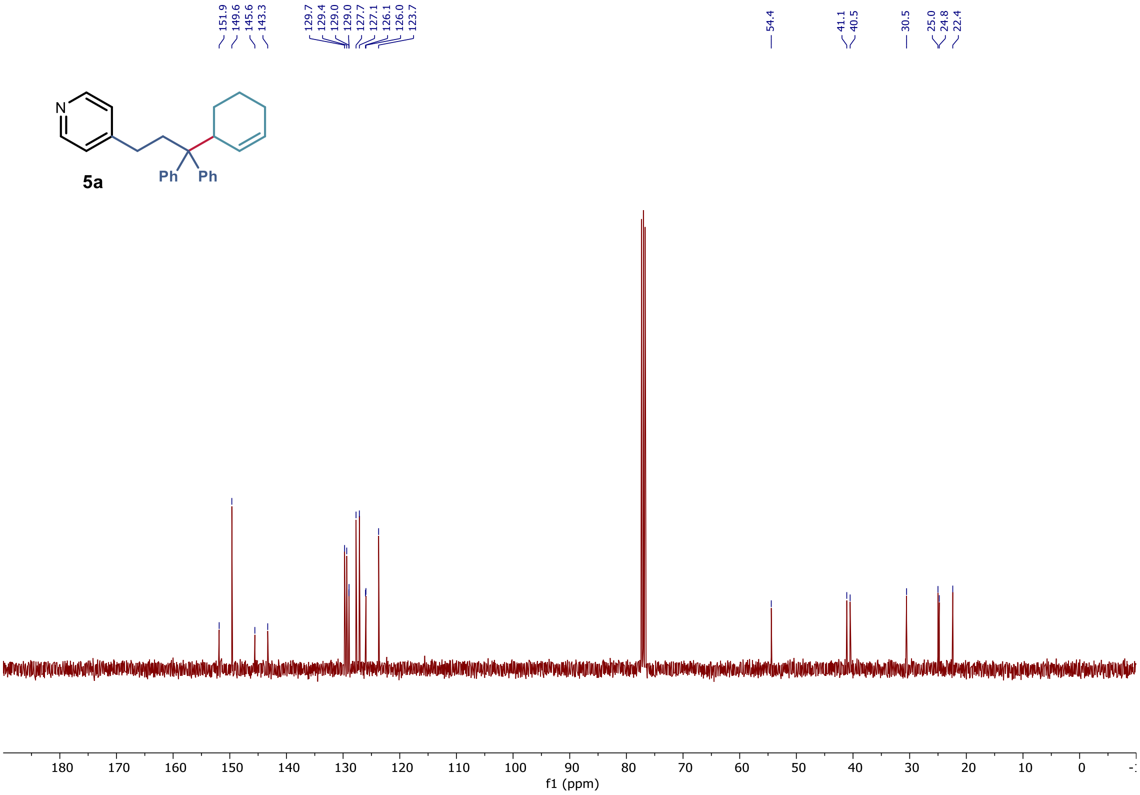

$^{1}$H NMR (400 MHz, $CDCl_3$) of **5b**

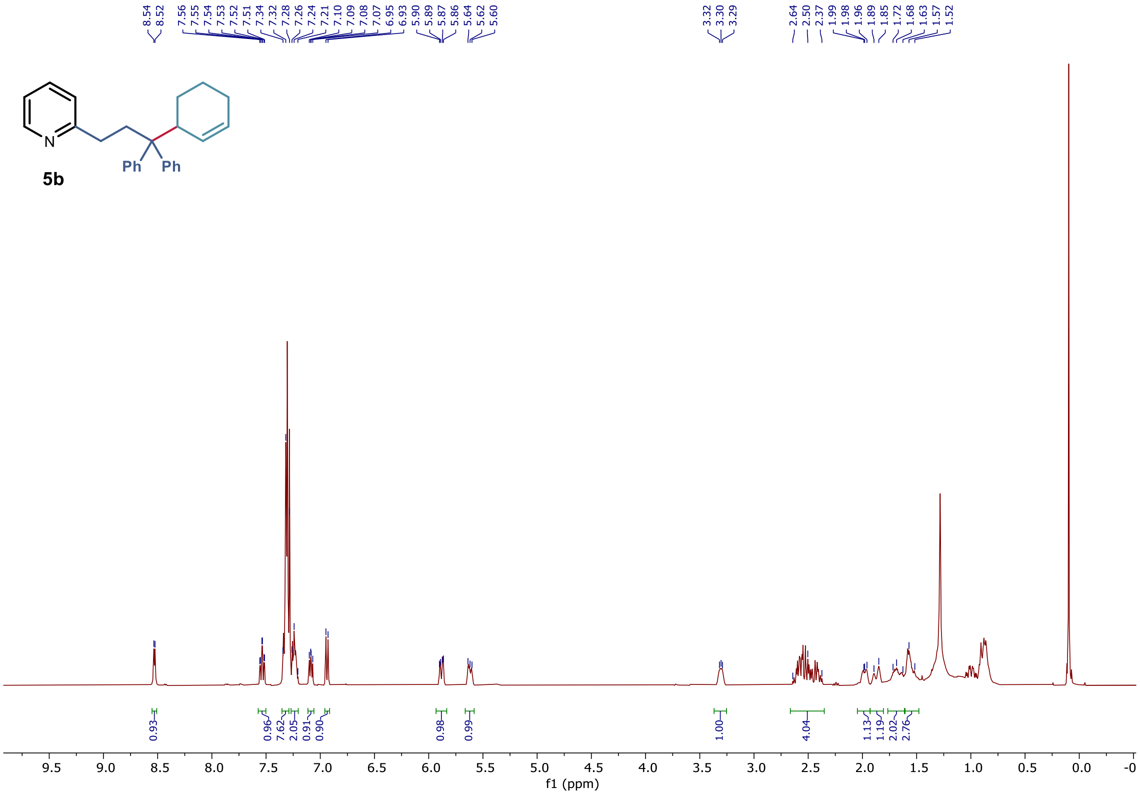


$^{13}$C NMR (101 MHz, $CDCl_3$) of **5b**

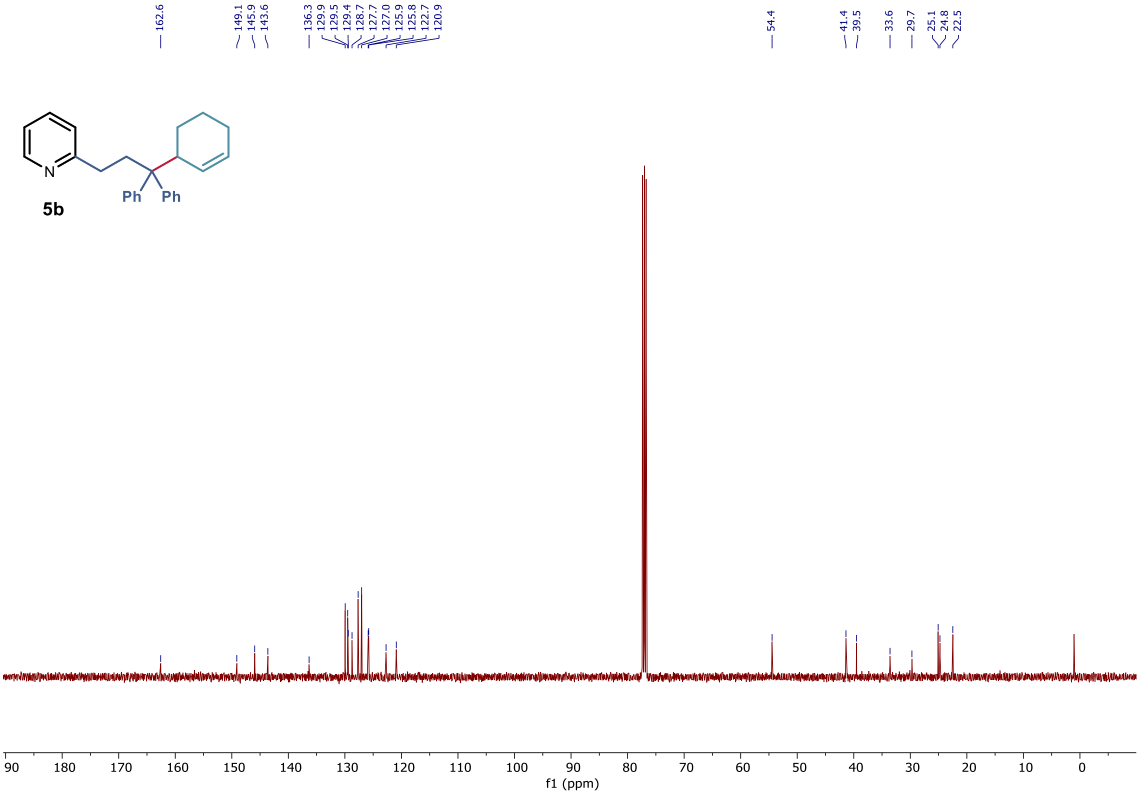

## J. UPC$^2$ traces

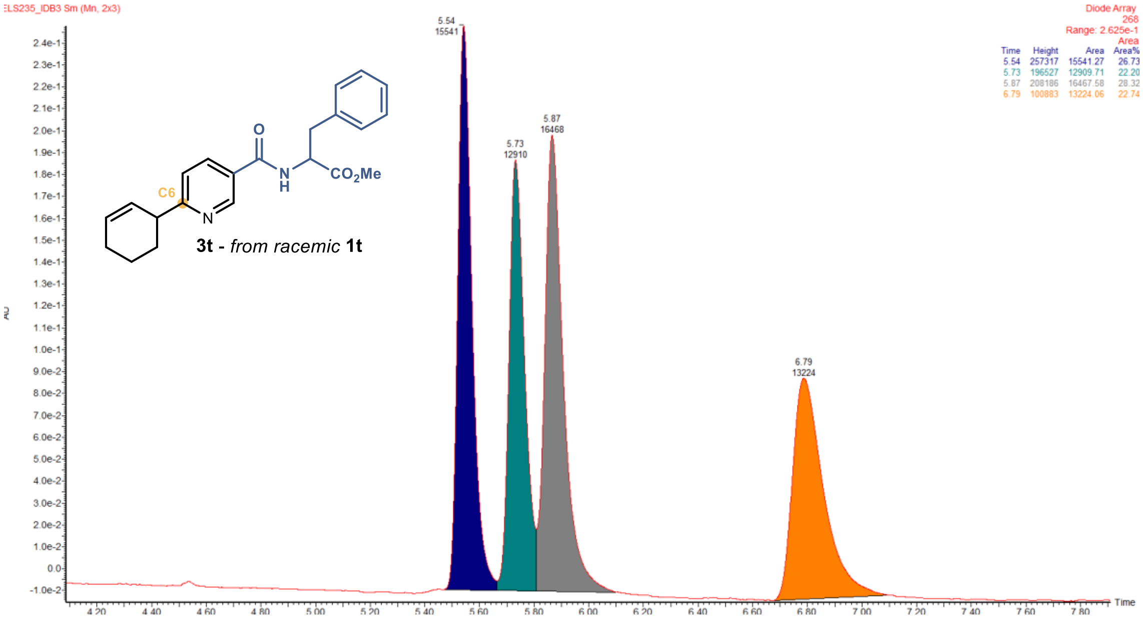


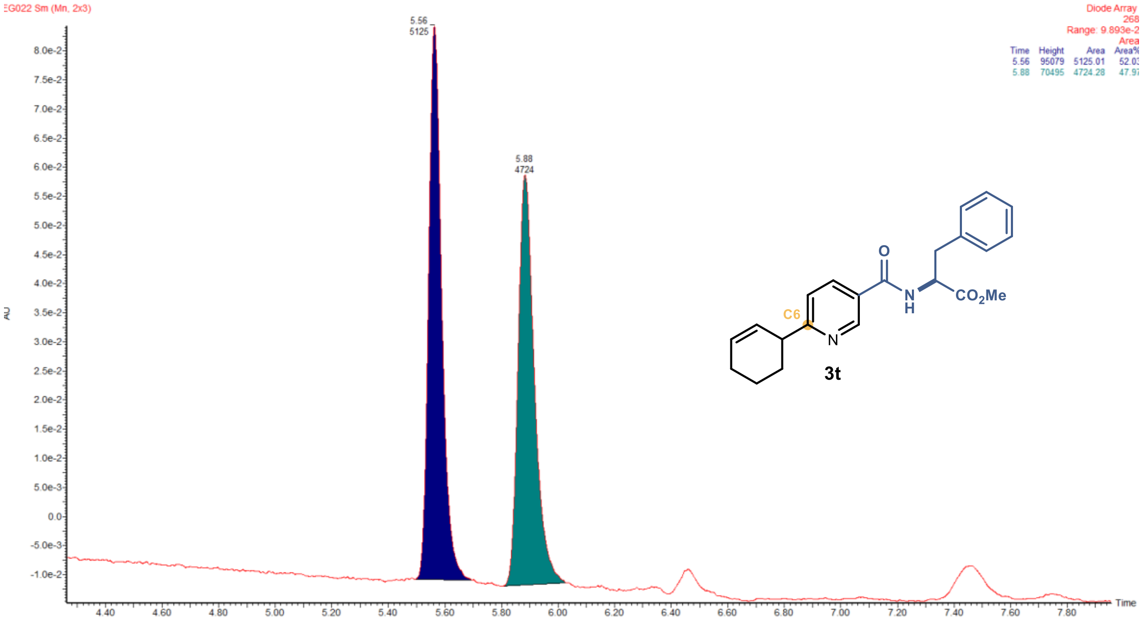

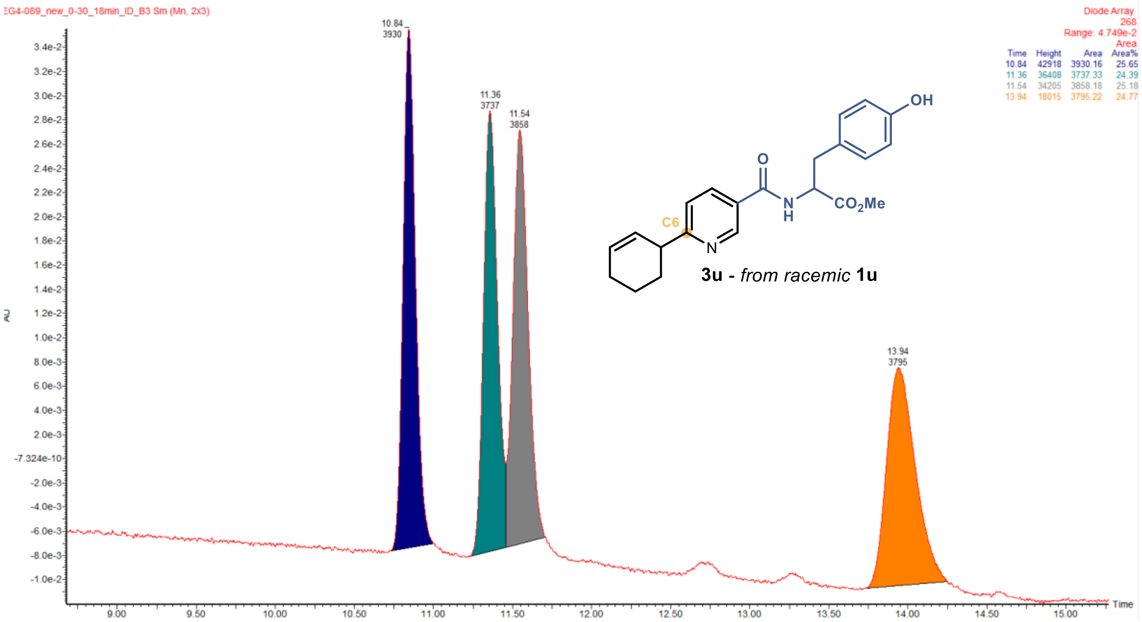
EG4-089_new_0-30_18min_ID_B3 Sm (Mn, 2x3)
Diode Array
268
Range: 4.749e-2
Area
Time Height Area Area%
10.84 42918 3930.16 25.65
11.36 36408 3737.33 24.39
11.54 34205 3858.18 25.18
13.94 18015 3795.22 24.77
3u - from racemic 1u
AU
Time

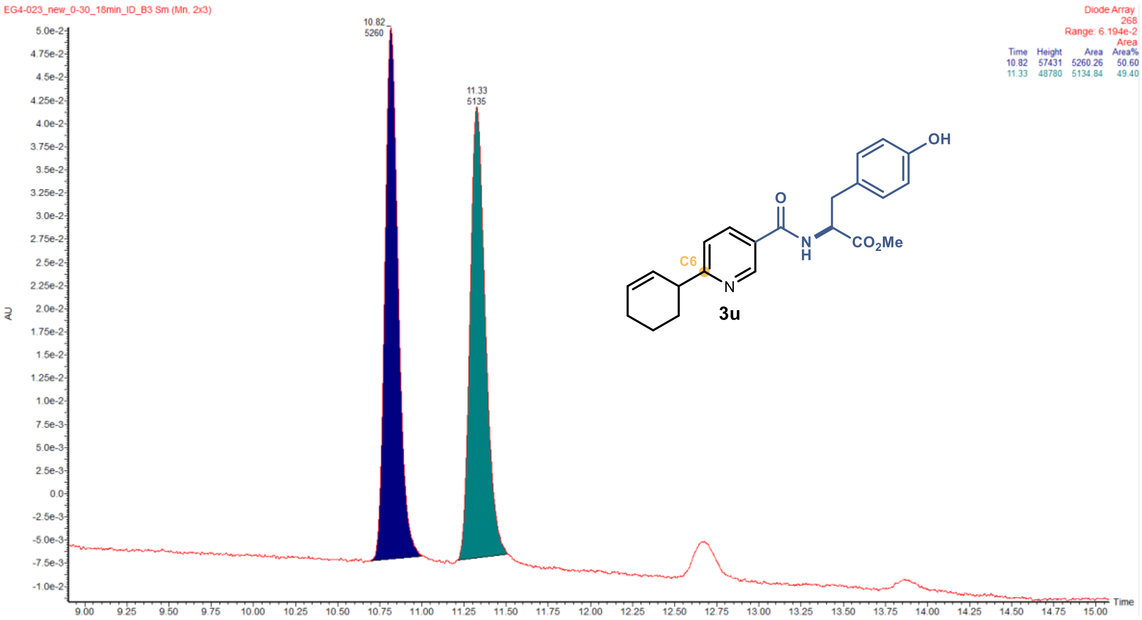
EG4-023_new_0-30_18min_ID_B3 Sm (Mn, 2x3)
Diode Array
268
Range: 6.194e-2
Area
Time Height Area Area%
10.82 57431 5260.26 50.60
11.33 48780 5134.84 49.40
3u
AU
Time

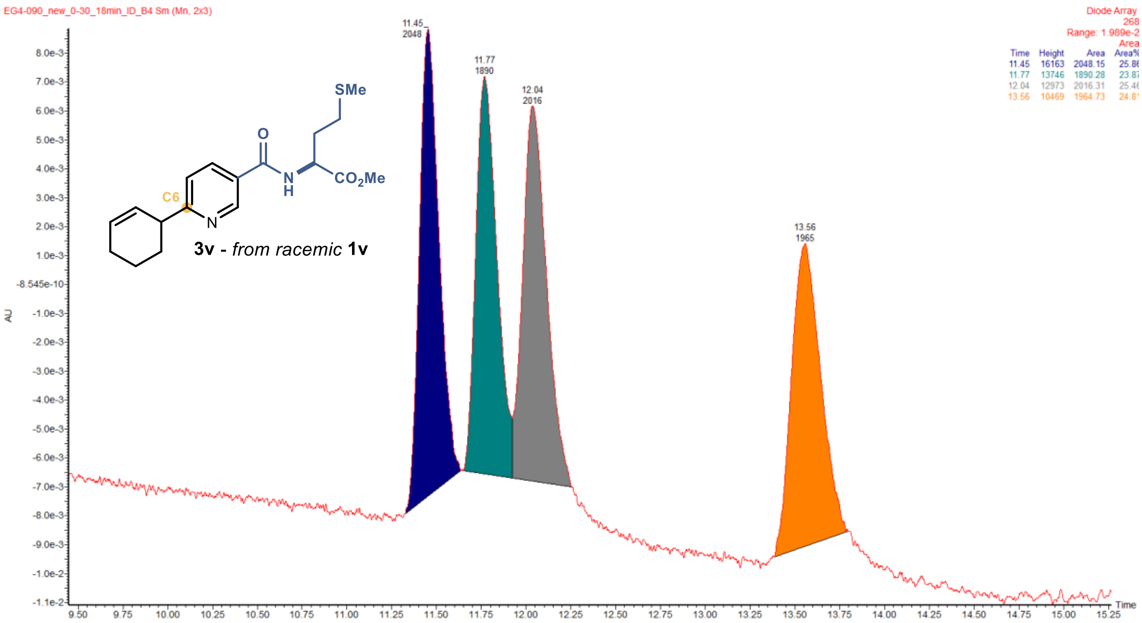
EG4-090_new_0-30_18min_ID_B4 Sm (Mn, 2x3)
Diode Array
268
Range: 1.989e-2
Time Height Area Area%
11.45 16163 2048.15
11.77 13746 1890.28
12.04 12973 2016.31
13.56 10469 1964.73
11.45
2048
11.77
1890
12.04
2016
13.56
1965
AU
Time
SMe
O
N
H
CO2Me
C6
N
3v - from racemic 1v

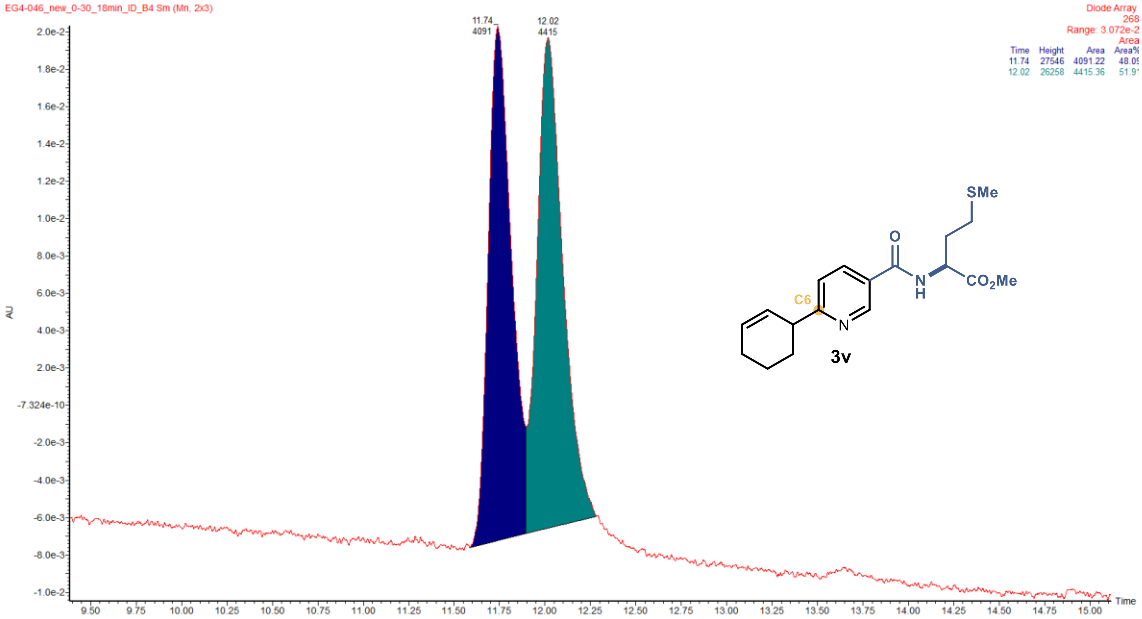
EG4-046_new_0-30_18min_ID_B4 Sm (Mn, 2x3)
Diode Array
268
Range: 3.072e-2
Time Height Area Area%
11.74 27546 4091.22
12.02 26258 4415.36
11.74
4091
12.02
4415
AU
Time
SMe
O
N
H
CO2Me
C6
N
3v

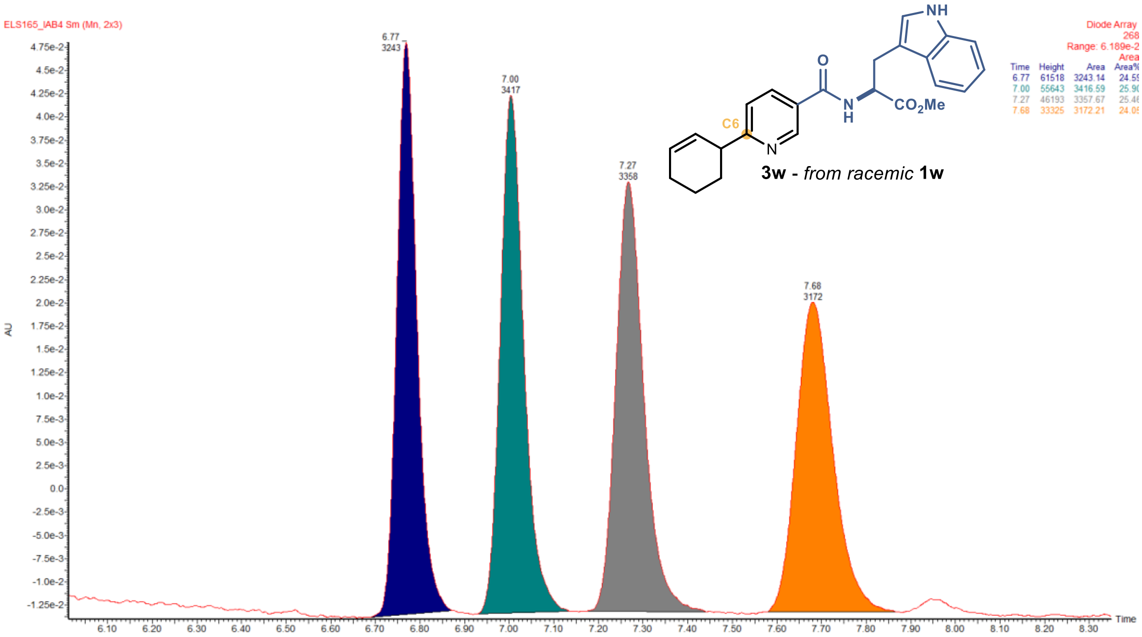
ELS165_IAB4 Sm (Mn, 2x3)
Diode Array
268
Range: 6.189e-2
Area
Time Height Area Area%
6.77 61518 3243.14 24.5
7.00 55643 3416.59 25.9
7.27 46193 3357.67 25.4
7.68 33325 3172.21 24.0
6.77
3243
7.00
3417
7.27
3358
7.68
3172
AU
Time
C6
NH
O
N
H
CO2Me
3w - from racemic 1w

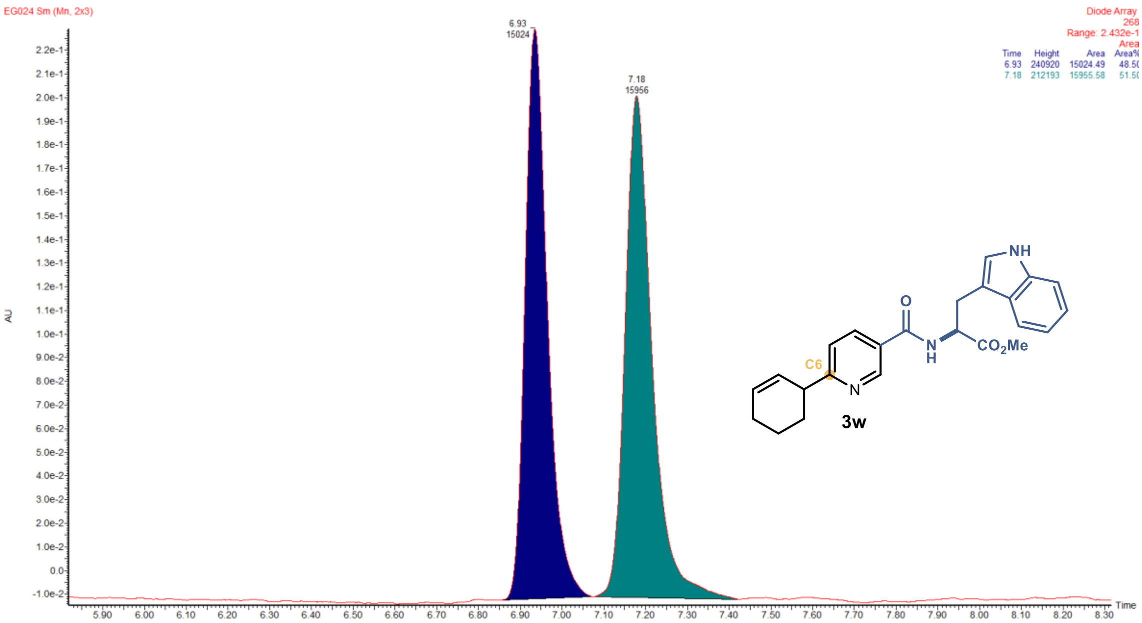
EG024 Sm (Mn, 2x3)
Diode Array
268
Range: 2.432e-1
Area
Time Height Area Area%
6.93 240920 15024.49 48.5
7.18 212193 15955.58 51.5
6.93
15024
7.18
15956
AU
Time
C6
NH
O
N
H
CO2Me
3w
